# Whole-genome resequencing to investigate the genetic diversity and mechanisms of plateau adaptation in Tibetan sheep

**DOI:** 10.1186/s40104-024-01125-1

**Published:** 2024-12-06

**Authors:** Xue Li, Buying Han, Dehui Liu, Song Wang, Lei Wang, Quanbang Pei, Zian Zhang, Jincai Zhao, Bin Huang, Fuqiang Zhang, Kai Zhao, Dehong Tian

**Affiliations:** 1 Key Laboratory of Adaptation and Evolution of Plateau Biota, Qinghai Provincial Key Laboratory of Animal Ecological Genomics, Northwest Institute of Plateau Biology, Chinese Academy of Sciences, Xining, Qinghai, 810008 China; 2 University of Chinese Academy of Sciences, Beijing, 100049 China; 3 Qinghai Sheep Breeding and Promotion Service Center, Gangcha, Qinghai, 812300 China

**Keywords:** Altitude adaptation, Domestication, Genetic selection, Population genetic structure, Tibetan sheep, Whole-genome sequencing

## Abstract

**Introduction:**

Tibetan sheep, economically important animals on the Qinghai–Tibet Plateau, have diversified into numerous local breeds with unique characteristics through prolonged environmental adaptation and selective breeding. However, most current research focuses on one or two breeds, and lacks a comprehensive representation of the genetic diversity across multiple Tibetan sheep breeds. This study aims to fill this gap by investigating the genetic structure, diversity and high-altitude adaptation of 6 Tibetan sheep breeds using whole-genome resequencing data.

**Results:**

Six Tibetan sheep breeds were investigated in this study, and whole-genome resequencing data were used to investigate their genetic structure and population diversity. The results showed that the 6 Tibetan sheep breeds exhibited distinct separation in the phylogenetic tree; however, the levels of differentiation among the breeds were minimal, with extensive gene flow observed. Population structure analysis broadly categorized the 6 breeds into 3 distinct ecological types: plateau-type, valley-type and Euler-type. Analysis of unique single-nucleotide polymorphisms (SNPs) and selective sweeps between Argali and Tibetan sheep revealed that Tibetan sheep domestication was associated primarily with sensory and signal transduction, nutrient absorption and metabolism, and growth and reproductive characteristics. Finally, comprehensive analysis of selective sweep and transcriptome data suggested that Tibetan sheep breeds inhabiting different altitudes on the Qinghai–Tibet Plateau adapt by enhancing cardiopulmonary function, regulating body fluid balance through renal reabsorption, and modifying nutrient digestion and absorption pathways.

**Conclusion:**

In this study, we investigated the genetic diversity and population structure of 6 Tibetan sheep breeds in Qinghai Province, China. Additionally, we analyzed the domestication traits and investigated the unique adaptation mechanisms residing varying altitudes in the plateau region of Tibetan sheep. This study provides valuable insights into the evolutionary processes of Tibetan sheep in extreme environments. These findings will also contribute to the preservation of genetic diversity and offer a foundation for Tibetan sheep diversity preservation and plateau animal environmental adaptation mechanisms.

**Supplementary Information:**

The online version contains supplementary material available at 10.1186/s40104-024-01125-1.

## Introduction

Small ruminants, particularly native breeds, are crucial to the livelihoods of a substantial portion of the human population from a socioeconomic perspective [1–4]. Thus, integrated trials focusing on management practices and genetic advancements to increase animal productivity are highly important [4–8]. Tibetan sheep, as small ruminants formed by long-term natural and artificial selection in the environment of the Qinghai–Tibet Plateau, exhibit notable traits such as cold resistance, tolerance to rough feeding conditions, disease resistance, and adaptation to high altitudes. These characteristics are essential to the livelihoods of pastoral communities throughout the Tibetan Plateau [9]. Currently, the population of Tibetan sheep is approximately 30 million, with approximately 17 indigenous sheep breeds [10]. Based on the ecological conditions of the production area and considering production and economic characteristics, Tibetan sheep can be divided into 3 groups: plateau-type (grassland-type), valley-type, and Euler-type [11]. However, the unique environmental conditions of the plateau, characterized by high altitude, low oxygen levels, intense radiation, and low temperatures, combined with the grazing-based feeding practices and traditional breeding methods, have resulted in low reproductive rates, slow growth, small body size in Tibetan sheep [12]. Furthermore, the harsh plateau environment poses substantial challenges to the introduction of other sheep breeds [13].

Indeed, comprehending the population structure and evolutionary relationships among species is essential for elucidating evolutionary processes, understanding the mechanisms underlying the development of unique traits, and informing the management and conservation of species resources [14]. Additionally, understanding domestication is pivotal for explaining the phenotypic, morphological, reproductive, and behavioral characteristics of domesticated animals [15]. These phenotypic modifications include economically significant traits such as fecundity and body structure, which are fundamental to modern sheep husbandry [16]. In recent years, numerous studies have examined the population structure and domestication process of sheep globally and within China, elucidating the sheep origins and migration patterns of sheep [17, 18]. However, these investigations has often focused on a limited number of Tibetan sheep breeds, or select only one breed of Tibetan sheep as a representative, resulting in an incomplete understanding of the phylogenetic evolution, phylogeography, and domestication of Tibetan sheep populations [19–21]. This study addresses this gap by investigating 6 distinct Tibetan sheep breeds in Qinghai Province, China, using whole-genome resequencing, providing a more detailed understanding of their population diversity, genetic structure, and domestication.

Another research focus for animals is understanding their adaptation to extreme environments [22]. The Tibetan Plateau, with an average elevation exceeding 4,000 m, represents a unique core area in Asia characterized by low temperatures, low oxygen availability, and strong ultraviolet radiation [23]. Recent studies have increasingly employed molecular evolution theory and techniques from molecular biology to elucidate the link between genes and adaptive traits [24, 25]. By analyzing extensive omics data, researchers have identified several candidate genes potentially involved in this process within the HIF hypoxia-inducible pathway [26], including endothelial PAS domain protein 1 (*EPAS1*) [27], egl-9 family hypoxia inducible factor 1 (*EGLN1)* [28], and vascular endothelial growth factor (*VEGF*) [29]. However, previous research on plateau adaptation has typically compared the adaptation mechanisms of animals in lowland and high-altitude regions (2,000–4,000 m). In contrast, we used whole-genome resequencing and transcriptomic analysis of Zhashijia sheep (ZS) residing at an altitude of 4,300 m and Valley Tibetan sheep (VT) residing at an altitude of 1,800 m to investigate the plateau adaptation mechanism of Tibetan sheep residing at different altitudes in the plateau region.

With the rapid development of genomics and bioinformatics, comparing genomic data across breeds, conducting functional annotations, and analyzing mutations can provide deep insights into the mechanisms of trait differentiation and species domestication [29–32]. By integrating genomic and phenotypic data, researchers can identify regions of the genome shaped by natural and artificial selection, helping to reveal the molecular foundations of species adaptation to various environments and production demands [33–37]. In this study, we used whole-genome resequencing technology to analyze, for the first time, the genetic structure, diversity, and domestication of 6 Tibetan sheep breeds from Qinghai Province, China. Additionally, we combined whole-genome resequencing with transcriptomics to explore the plateau adaptation mechanisms of Tibetan sheep residing at different altitudes in the plateau region. This study provides significant insights into the genetic basis of adaptation and domestication in Tibetan sheep, contributing to the conservation of genetic diversity among plateau species and enhancing our understanding of environmental adaptation in these unique ecosystems.

## Methods

### Sample collection and data downloads

The study included 6 breeds of Tibetan sheep: Plateau Tibetan sheep (PT), Valley-type Tibetan sheep (VT), and Euler-type Tibetan sheep (EL), Zhashijia sheep (ZS), Zeku sheep (ZK), and Guinan black fur sheep (GB). Each breed was represented by 20 adult sheep (10 males and 10 females), which were selected based on specific breed characteristics. Ear tip tissue samples were collected from each sheep and preserved in 95% alcohol. Additionally, Argali sheep was selected as the wild ancestor of domestic sheep owing to its close phylogenetic relationship and similar living environment. The sequencing data from 10 Argali samples sourced from NCBI (SRX2728088, SRX2728091, SRX2728092, SRX2728094, SRX2728095, SRX2728097, SRX2729596–SRX2729599) were integrated into the analysis.

Four adult individuals (2 males and 2 females) from each of the ZS and VT groups were administered deep anesthesia with 3% pentobarbital sodium intravenously according to relevant institutional guidelines/ethical regulations for animal research. Following anesthesia, the animals were euthanized through exsanguination during the healthy physiological stage. The blood present on the surface of the heart and lungs was rinsed with phosphate-buffered saline (PBS). Subsequently, 1 cm^3^ of heart and lung tissues from the same part of each sheep were promptly frozen in liquid nitrogen and stored at −80 °C for subsequent analysis. The animal experiments were approved by the Animal Ethics Welfare Committee of the Northwest Institute of Plateau Biology, Chinese Academy of Sciences (Approval No. NWIPB2023015). All experiments and methods were performed according to the relevant guidelines and regulations.

### DNA extraction and whole‑genome sequencing

High-quality DNA was extracted using a TIANGEN^®^ TIANamp Genomic DNA Kit (Tiangen, Beijing, China). DNA purity and integrity were subsequently evaluated by 1% agarose gel electrophoresis and a spectrophotometer. The DNA concentration was quantified using a fluorometer-based assay. Sequencing was performed by an external company (Personal Biotechnology Co., Ltd., Shanghai, China) on the Illumina NovaSeq 6000 platform. The sequencing generated 150 bp paired-end reads with a target depth of 10-fold coverage per genome.

### Variant calling

The raw read quality was assessed using FastQC (v0.21) with the default settings [38]. High-quality paired-end reads (either 150–100 bp) were then mapped to the sheep reference genome (ARS–UI_Ramb_v2.0, https://www.ncbi.nlm.nih.gov/assembly/GCF_016772045.1) using the Burrows–Wheeler Aligner (BWA v.0.7.12) [39]. The resulting SAM files were sorted and converted to BAM format using Picard software (v1.107, http://www.psc.edu/index.php/user-resources/software/picard) with the “FixMateInformation” command to ensure consistency. Because library preparation steps can introduce duplicate reads, Picard’s “MarkDuplicates” function was employed to eliminate these duplicates. Only reads with the highest mapping scores were retained for further analysis. Single-nucleotide polymorphism (SNP) and population insertion/deletion (InDel) variant sites were called using the Genome Analysis Toolkit (GATK v3.8) UnifiedGenotyper with stringent quality control parameters (stand_call_conf ≥ 30; stand_emit_conf ≥ 10) [40]. InDel variants were further filtered based on established quality criteria (FS ≤ 200, DP > 4, QD ≥ 2, ReadPosRankSum ≥ −20) and missing sites were removed. Finally, ANNOVAR software was used to annotate the identified SNPs and InDels [41].

### Population genetics analysis

Pairwise identical-by-state (IBS) distance matrices were computed using PLINK v1.90 to assess genetic relatedness among individuals [42]. Principal component analysis (PCA) was performed on the SNP data (excluding minor allele frequency [MAF] < 0.05) using GCTA software (http://cnsgenomics.com/software/gcta). A maximum likelihood phylogenetic tree was then constructed with FastTree software (http://www.microbesonline.org/fasttree/), using Argali sheep as an outgroup. The reliability of the tree branches was assessed through bootstrapping with 1,000 replications.

Population structure analysis was conducted using ADMIXTURE software (http://dalexander.github.io/admixture) based on SNP information. The hybrid model was employed with a range of K values (2–10) representing the number of ancestral populations. Default settings were used for other parameters. The optimal K value closest to the true underlying structure was determined based on cross-validation error.

Treemix v1.13 was employed to analyze gene flow patterns using genome-wide allele frequency data [43]. This software leverages population divergence and admixture events to construct a maximum likelihood tree. Following tree construction, migration events were inferred and depicted as directional arrows on the tree, visually representing the inferred gene flow between populations.

### Genetic diversity

A filtered set of 48.8 million high-quality SNPs was employed to estimate genetic diversity. The software package Stacks (http://creskolab.uoregon.edu/stacks/) and its Populations program were used to calculate genetic parameters. These parameters included observed heterozygosity (Ho), expected heterozygosity (He), nucleotide diversity (Pi), and inbreeding coefficients (*F*_IS_), as defined by Catchen et al. [44]. This comprehensive approach allowed for a detailed assessment of genetic variation within the studied Tibetan sheep populations.

Population differentiation (*F*_ST_) between breeds was assessed using VCFtools and GCTA software with linkage disequilibrium filtering (“-indep -pairwise 50 5 0.2”) [45].

Finally, linkage disequilibrium across the genome was estimated using Haploview software [46]. Here, 500,000 SNPs were randomly selected and analyzed with parameters set to minimize missing data and focus on common variants (“-missingCutoff 0.2 -dprime -minMAF 0.1”). Linkage disequilibrium (LD) decay patterns were explored by grouping SNP pairs based on physical distance and calculating the average *r*-squared (mean *r*^2^) value for each group.

### Unique SNP analysis

STRUCTURE software (v2.3.4) was used to analyze genotype frequencies across populations to identify SNPs unique to Tibetan and Argali sheep populations [47]. Each SNP has 4 possible genotypes: homozygous reference (0/0), heterozygous (0/1), homozygous alternative (1/1), and missing data (./.). By comparing genotype frequencies between populations, unique SNPs can be identified. If a specific genotype has frequencies of 1 (all individuals) in population A and 0 (no individuals) in population B, it is considered unique to population A. Conversely, genotypes with equal frequencies in both populations are considered shared.

### Detection of selective signatures

#### Selective signatures in Tibetan sheep during domestication

To identify the genomic signatures of selection in Tibetan sheep, 6 breeds were combined and compared to Argali sheep. The cross-population composite likelihood ratio test (XR-CLR) method implemented in XP-CLR v1.0 was used to identify selection signals and determine the sweep likelihood. Only SNPs with less than 10% missing data were included (“-max-missing 0.9”) [48]. XP-CLR scores were calculated with a 2 kb grid spacing, a maximum of 200 SNPs per 0.5 cM window, and a downweighting of highly correlated SNPs (*r*^2^ > 0.95). The top 1% of regions based on XP-CLR scores were considered candidate selection sweeps.

#### Selection for high-altitude adaptation

To identify genomic regions potentially linked to high-altitude adaptation, we performed a targeted scan using the XP-CLR test. The experimental group, consisting of 20 high-altitude Tibetan sheep populations (ZS) residing at an elevation of 4,300 m, was compared with a control group of 20 low-altitude populations (VT) living at an elevation of 1,800 m. The top 1% of the genomic regions with the highest regional-level XP-CLR scores were designated candidate selective sweep regions.

#### Gene annotation, GO, and KEGG analyses

Genes within the selected regions were annotated using the sheep reference assembly ARS-UI_Ramb_v2.0. Functional enrichment analysis was then conducted using the Gene Ontology (GO) database (http://geneontology.org) and the Kyoto Encyclopedia of Genes and Genomes (KEGG) database (http://www.genome.ad.jp/kegg/) [49]. We identified significantly enriched GO terms and KEGG pathways based on a *P* value threshold of 0.05.

### RNA-seq analysis

RNA extraction, sequencing, and transcriptome profiling were conducted on four biological replicates of hearts and lungs from ZS and VT. Total RNA was extracted from each sample using TRIzol (Invitrogen, Carlsbad, CA, USA), followed by assessments of degradation and contamination on 1% agarose gels and verification of purity using a Nanodrop^®^ spectrophotometer (Thermo, Carlsbad, CA, USA). RNA concentration was quantified using a Qubit^®^ RNA Assay Kit and Qubit^®^ 2.0 Fluorometer (Life Technologies, Carlsbad, CA, USA). The RNA integrity was evaluated using an RNA Nano 6000 Assay Kit on an Agilent Bioanalyzer 2100 system (Agilent Technologies, Santa Clara, CA, USA). Each sample utilized 1.5 µg of RNA as input material for RNA sample preparation. Subsequently, sequencing libraries were produced with the MGIEasy RNA Library Prep Kit (BGI Co., Ltd., Shenzhen, Guangdong, China) according to the manufacturer’s instructions. The resulting library was sequenced on the DNBSEQ-T7 platform, generating 100 bp paired-end reads (BGI Co., Ltd., Shenzhen, Guangdong, China).

SAMtools filtering was employed with the parameters “-l 15 -q 0.2 -n 0.05” for quality control of the read data [50]. A reference genome index was subsequently constructed, and high-quality RNA-seq reads were subsequently aligned to the reference genome using HISAT2 (v2.0.4) with the parameters “--sensitive --no-discordant --no-mixed -I 1 -X 1,000 -p 8 --rna-strandness RF” [51]. Differential expression analysis was carried out using the R package DESeq2 (v1.38.3) based on read count numbers [52]. The statistical significance of variations in gene expression was assessed through the Wald test, with a threshold of *P* < 0.05 deemed significant. Enrichment analyses of GO and KEGG pathways, as well as the construction of a PPI network for genes displaying a |log_2_fold-change| ≥ 2, were conducted using the online platform Dr. Tom, which is made available by BGI (http://biosys.bgi.com).

## Results

### Whole-genome sequencing and variation

**Fig. 1:**
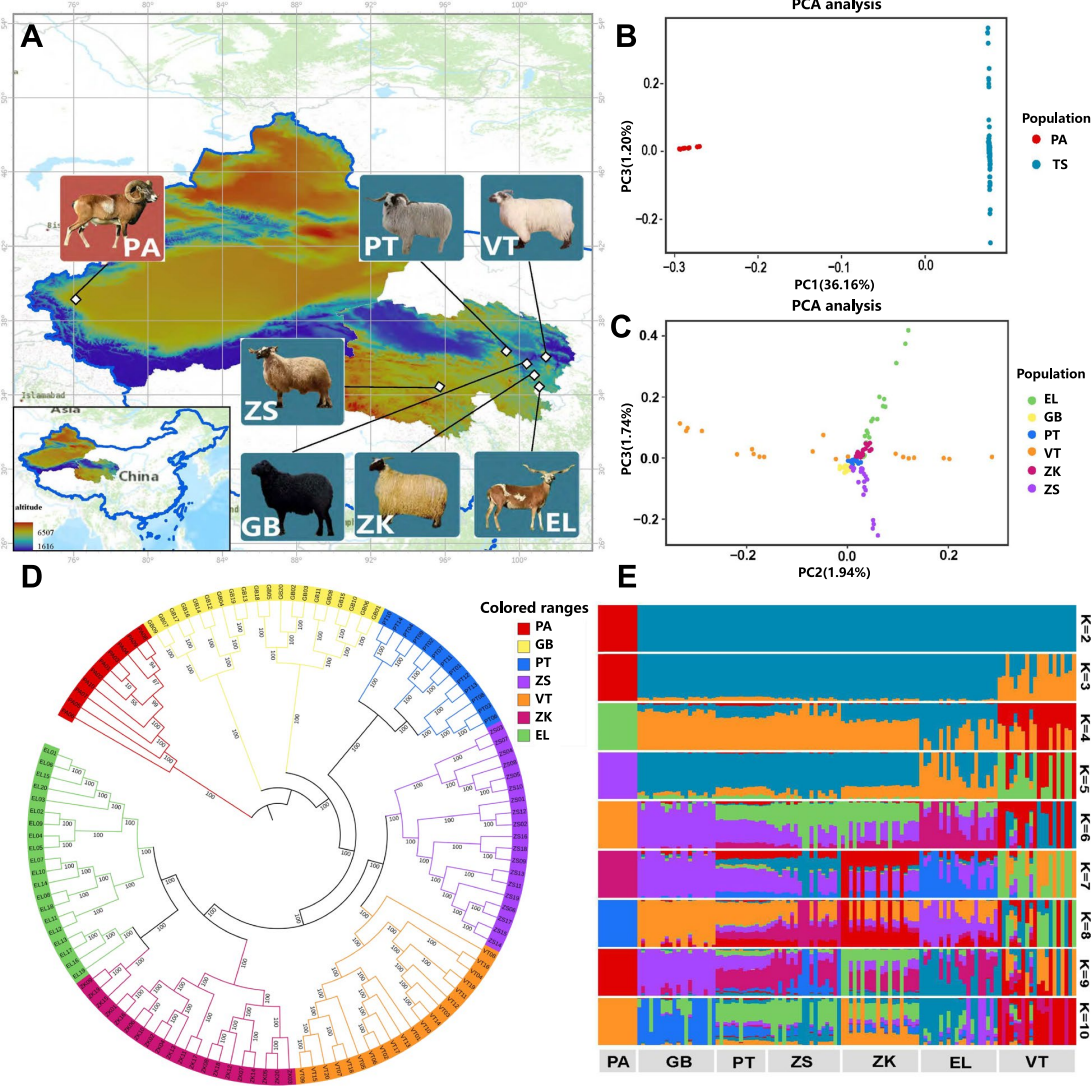
Phylogenetic relationships and population structure of Tibetan sheep. **A** Geographical distribution map of sample collection. **B** PCA of Pan sheep and Tibetan sheep. **C** PCA of the 6 Tibetan sheep breeds. **D** Utilizing the maximum likelihood algorithm in fast tree software, a phylogenetic tree of the 6 Tibetan sheep breeds was constructed using Argali sheep as the outgroup. **E** Based on whole-genome SNP data, the population genetic structure of the 6 breeds of Argali and Tibetan sheep were inferred using admixture software (set K = 2–10). PA: Argali sheep; TS: Tibetan sheep; GB: Guinan Black Fur sheep; PT: Plateau Tibetan sheep; ZS: Zhashijia sheep; VT: Valley Tibetan sheep; ZK: Zeku sheep; EL: Euler Tibetan sheep. Each column in the image represents an individual, and the length of each different colored segment indicates the proportion of an ancestor in the individual’s genome. The labels on the image denote the groups

For this study, sequencing data were obtained for a total of 130 sheep, consisting of 120 Tibetan sheep from 6 different breeds (EL, GB, PT, VT, ZS, ZK), and sequencing data from 10 Argali sheep were sourced from NCBI. The geographical distribution of the samples is presented in Fig. 1A and detailed in Table S1.

Assessment of the sequencing data quality revealed 0% fuzzy bases, a mean GC content of 45.12%, and averages of 97.23% and 92.82% of the bases with base recognition accuracies exceeding 99% (Q20) and 99.9% (Q30), respectively (Table S2). These values met the stipulated criteria of Q20 > 90%, Q30 > 85%, and a GC content of approximately 50%, confirming the adequacy of the sequencing data quality.

The sequencing data contained low-quality reads, such as adapter dimers, which could hinder subsequent information analysis. To ensure the quality of the ensuing data analysis, additional filtering of the raw sequencing data was necessary to generate high-quality data. Details of the data filtering process are provided in Table S3. Following filtration, 23.95 billion high-quality reads (HQ reads) were preserved, resulting in a total data size of approximately 3.54 TB. Furthermore, the mean genome coverage achieved was 99.68%, with a range of 86.79%–99.94% (Table S4). Therefore, the sequencing quality and choice of reference genomes aligned with the requirements for subsequent analysis.

### Characterization of the variants

Analysis of detected and filtered SNPs revealed an average of 33.46 million SNPs per individual. Of these, 7.94 million were heterozygous, and 6.36 million were homozygous. Additionally, 10.07 million SNPs were categorized as purine transitions (Ts), and 4.23 million SNPs were categorized as purine‒pyrimidine transversions (Tv). These findings are detailed in Table S5. A comprehensive annotation of approximately 48.76 million SNPs across 130 individuals was performed. Most SNPs were located in intergenic regions (60.4%), followed by introns (35.9%), exons (1.33%), synonymous single-nucleotide variants (SNVs) (0.86%), 3′UTRs (0.78%), and nonsynonymous SNVs (0.43%), with additional categories detailed in Table S6.

To identify the target trait, each group of trait-related samples, collectively referred to as InDels, was filtered to determine the final number of InDels in each sample (Table S7). On average, individuals contained approximately 3.10 million InDels, with 0.92 million being heterozygous and 0.93 million being homozygous. Of these, 0.84 million were insertions, and 1.00 million were deletions. A comprehensive annotation of approximately 5.14 million InDels revealed that the majority were in intergenic regions (59.72%), followed by introns (37.32%), 3′UTRs (0.92%), downstream regions (0.75%), upstream regions (0.73%), 5′UTRs (0.34%), exons (0.16%), and other regions (Table S8).

### Phylogenetic relationships and population structure

Our analysis of genetic variations (IBS) revealed significant associations, as detailed in Table S9. The genetic distances ranged from 0.042 to 0.352 across the entire population. Specifically, the average genetic distance between Argali and Tibetan sheep was 0.351. The greatest genetic distance was observed between EL individuals and Argali individuals, suggesting potential subgroup differentiation or distant kinship between Argali and Tibetan sheep. Conversely, the Argali population presented the smallest genetic distance, indicating a close genetic relationship and high genetic similarity within the population. In contrast, the genetic distance observed within the Tibetan sheep population exceeded that of the Argali population, suggesting greater genetic diversity among individual Tibetan sheep.

To investigate the relationships among the seven sheep breeds, PCA was using two separate approaches. First, a comparative analysis was performed that included all 6 Tibetan sheep breeds collectively with Argali sheep. PCA focused specifically on the 6 Tibetan sheep breeds. The results of these analyses are presented in Fig. 1B and C. The results revealed that Argali and Tibetan sheep were distinguishable, as well as the 6 individual Tibetan breeds, although some overlaps were observed in population of EL, ZK, GB and ZS. Moreover, PCA was used to identify and remove outlier samples, resulting in a final selection of 122 individuals out of the initial 130 (GB = 20; ZS = 19; EL = 20; PA = 10; VT = 20; PT = 13; ZK = 20).

As demonstrated in Fig. 1D, the phylogenetic tree using Argali sheep as an outgroup revealed that samples from the same breed clustered closely together, indicating clear genetic differentiation within the breed populations. The divergence of Argali and Tibetan sheep occurred simultaneously, with the following order of differentiation observed: GB first, followed by PT, then ZS, VT, ZK, and EL.

To validate the previously observed genetic differentiation between the 6 Tibetan sheep breeds and Argali sheep, STRUCTURE software was used to assess the proportions of the shared ancestry among the seven breeds. An unsupervised clustering model was utilized to investigate the population structures for different numbers of ancestral groups (K = 2–10). The results presented in Fig. 1E suggest that at K = 2, Argali and Tibetan sheep could be traced back to separate ancestral lineages. At K = 4, the genetic data indicated that Tibetan sheep had genetic contributions from multiple ancestral groups. Most of the genetic information for GB, PT, ZS, and ZK originated from a common ancestral group (yellow), with EL and VT showing predominant genetic contributions from blue and red ancestral groups, respectively.

**Fig. 2:**
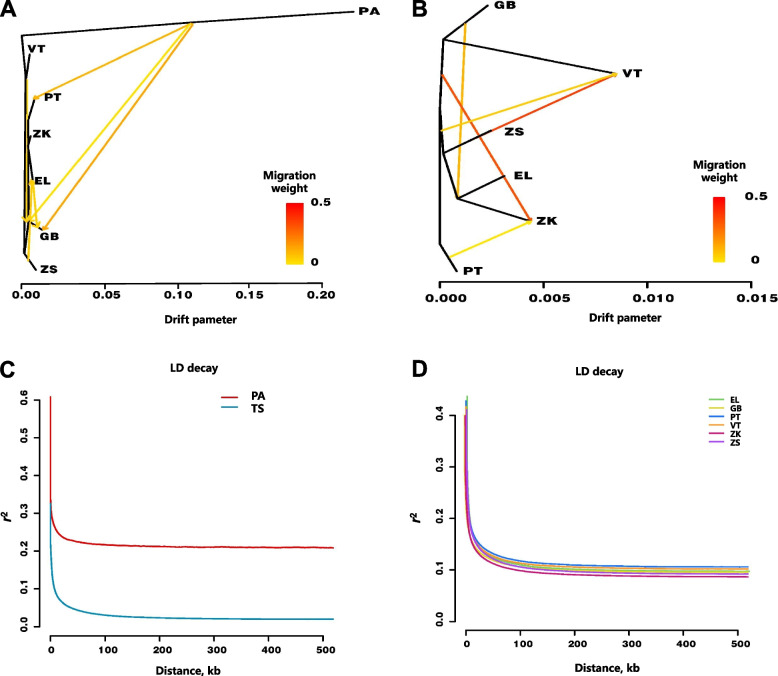
Gene flow and LD analysis of the 6 breeds of Tibetan and Argali sheep. **A** Gene flow diagram between Argali sheep and the 6 Tibetan sheep breeds. The arrow direction in the figure corresponds to the direction of gene drift. The *x*-axis represents the 10 times average standard deviation of the elements in the sample covariance matrix. **B** Gene flow diagram of the 6 Tibetan sheep breeds. The arrow direction in the figure corresponds to the direction of gene drift. The *x*-axis represents the 10 times average standard deviation of the elements in the sample covariance matrix. **C** Distribution map of LD in Argali and Tibetan sheep. The horizontal axis represents the distance between SNPs, and the vertical axis represents the *r*^2^ value. **D** Distribution map of LD among the 6 Tibetan sheep breeds. PA: Argali sheep; TS: Tibetan sheep; GB: Guinan Black Fur sheep; PT: Plateau Tibetan sheep; ZS: Zhashijia sheep; VT: Valley Tibetan sheep; ZK: Zeku sheep; EL: Euler Tibetan sheep. The horizontal axis represents the distance between SNPs, and the vertical axis represents the *r*^2^ value

A maximum likelihood tree was constructed using genome-wide allele frequency data. Migration events were then inferred on the phylogenetic tree, with arrows indicating the direction of gene flow (Fig. 2A and B). The results revealed gene flow from Argali sheep to the PT and GB breeds. Additionally, gene flow events were observed between Tibetan sheep breeds, including ZS to VT, PT to ZK, VT and a shared ancestor of GB to ZK, GB to a shared ancestor of EL and ZK, and a shared ancestor of ZS, EL, and ZK to VT.

### Genomic diversity

This study utilized 6 genetic indicators (listed in Table S10) to estimate the levels of genetic diversity within the populations. The findings revealed that Tibetan and Argali sheep presented Ho, He, Pi, and *F*_IS_ values ranging from 0.054 to 0.246, 0.044 to 0.259, 0.267 to 0.046, and 0.075 to −0.007, respectively. In addition, the Ho and Pi of the Argali population were relatively low, whereas the two indices of the 6 Tibetan sheep populations ranged from 0.23 to 0.27, indicating that the population diversity was moderate. Generally, *F*_IS_ values are expected to be positive, yet the *F*_IS_ value for the Argali population examined was determined to be −0.007 in this study, suggesting a potential deviation from Hardy–Weinberg equilibrium and implying constraints on the population capacity for self-breeding [53].

The population differentiation index (*F*_ST_) quantifies the level of genetic differentiation among subpopulations within a larger population, with values typically ranging from 0 to 1 [54]. The results of this study showed that the *F*_ST_ values between Argali sheep and 6 breeds of Tibetan sheep were all greater than 0.25, indicating that there were obvious genetic differentiation. However, the *F*_ST_ values among the 6 breeds of Tibetan sheep breeds were consistently less than 0.05, indicating that the levels of differentiation were very small (Table S11).

The study investigated the decay of LD as quantified by the squared correlation coefficient (*r*²) in the seven distinct sheep breeds at different physical distances. To provide a clearer representation of the decay rate across the seven sheep breeds, LD decay was analyzed in two ways: first, by treating the 6 Tibetan sheep breeds as a whole (TS) compared with Argali sheep, and second, by focusing solely on the six Tibetan sheep breeds. The findings presented in Fig. 2C and D revealed that TS exhibited a faster decay rate than Argali sheep. Among the 6 Tibetan sheep breeds, the PT subtype displayed the slowest decay rate, whereas the ZK subtype exhibited the fastest decay rate. These results suggested that the ZK population has undergone the highest degree of domestication and experienced the most intense artificial selection. Conversely, the PT subtype presented the highest group diversity and the lowest intensity of artificial selection.

### Unique SNPs in Argali and Tibetan sheep

Despite the absence of breed-specific SNPs within the Tibetan sheep populations, this study conducted a comparative analysis of SNPs between the genomes of Argali sheep and TS. The primary goal was to identify unique SNPs associated with specific biological processes and functions in TS with their wild ancestor, Argali sheep.

**Fig. 3:**
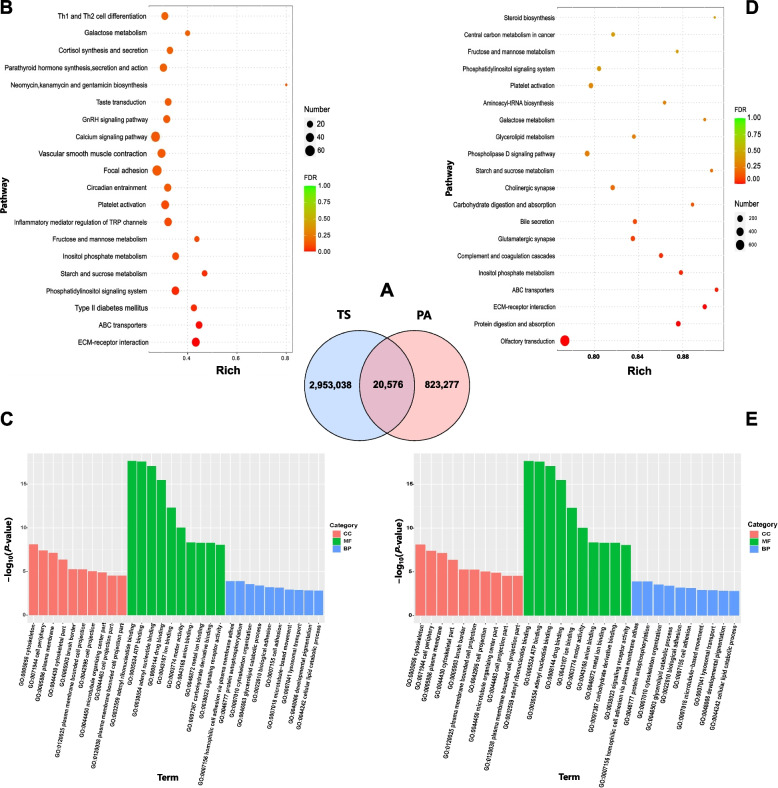
Analysis of SNPs unique to Argali and Tibetan sheep. **A** Venn diagram of unique and shared SNPs in Argali and Tibetan sheep. PA: Argali sheep; TS: Tibetan sheep. **B** KEGG enrichment analysis of Argali-specific SNPs. **C** GO enrichment analysis of Argali-specific SNPs. **D** Enrichment analysis of KEGG pathways for Tibetan sheep-specific SNPs. **E** Enrichment analysis of GO terms for Tibetan sheep-specific SNPs

As illustrated in Fig. 3A and detailed in Table S12, Argali and Tibetan sheep presented 2,953,038 and 823,277 unique SNPs, respectively, with 20,576 shared SNPs. The annotation results for SNPs unique to Argali and Tibetan sheep are presented in Tables S13 and S14, respectively.

The KEGG pathway enrichment analysis of unique SNPs in Argali sheep revealed significant enrichment in various biological processes, including nutrient metabolism (including starch, sucrose, inositol phosphoric acid, fructose, mannose, and galactose), gastric acid secretion, substance transshipment (ABC transporters), hormone synthesis and secretion (including thyroid hormone, insulin, growth hormone, aldosterone, and cortisol.), and signal transduction pathways (including taste transduction, phosphatidylinositol signaling system, axon guidance, and ECM-receptor interaction), as depicted in Fig. 3B and Table S15. The findings from the GO analysis of unique SNPs in Argali sheep predominantly revealed enrichment in material binding categories such as ATP binding, drug binding, ion binding, carbohydrate derivative binding, and nucleotide binding, along with ATPase activity, cytoskeleton organization, basement membrane composition, and other cellular structures (Fig. 3C and Table S16).

In particular, the results of the KEGG and GO enrichment analyses of unique SNPs in Tibetan sheep closely resembled those observed in Argali sheep. The enriched KEGG pathways were predominantly associated with processes such as nutrient digestion, hormone synthesis and secretion, digestive juice secretion, taste signal transduction, and immune-related pathways (Fig. 3D and Table S17). Similarly, GO-enriched pathways were linked to material binding, ATPase activity pathways, and cellular structures (Fig. 3E and Table S18).

### Detection of selective signatures

#### Selective signatures in Tibetan sheep during domestication

Our findings revealed that although the 6 Tibetan sheep breeds could be distinguished, they presented a high degree of genetic similarity (*F*_ST_ < 0.05). Consequently, the 6 breeds were collectively treated as the experimental group, with the wild Argali sheep serving as the control group. The genomic regions of Tibetan sheep that underwent natural selection and artificial domestication during domestication were identified through the XR-CLR.

**Fig. 4:**
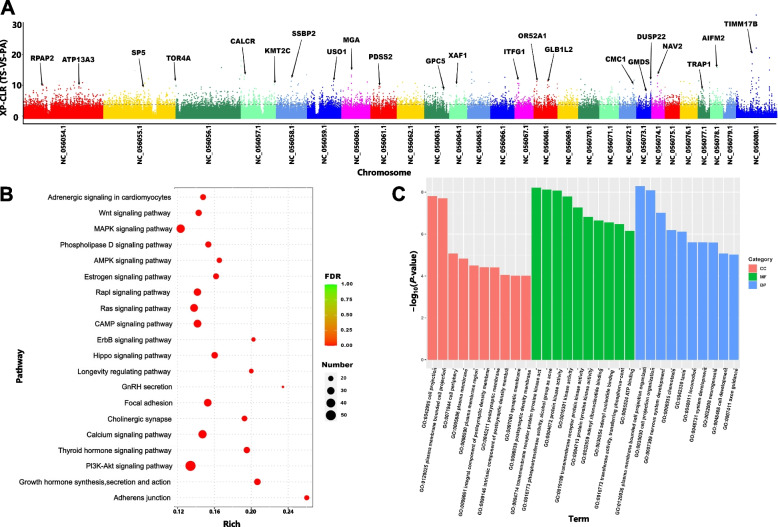
Selective signatures of Tibetan sheep during the introduction of improvements. **A** Manhattan plot of genome-wide selective sweep signals of Argali and Tibetan sheep. The horizontal axis represents the chromosomes, and the vertical axis represents the xpclr_norm values. Taking the first 1% of the xpclr_norm values (pclr_norm ≥ 3.726865) as the selection criterion, the value is represented by a dashed line, which indicates the selective elimination region during the domestication process of Tibetan sheep. The text in the figure indicates genes related to domestication with significant selection intensity. **B** KEGG functional enrichment of selectively eliminated genes. **C** GO functional enrichment of selectively downregulated genes

The study findings revealed that 2,698 selective elimination regions presented extreme values exceeding a predefined threshold in the XR-CLR test (top 1%, xpclr_norm ≥ 3.726865). These regions were associated with a total of 1,470 genes (Table S19). Based on the xpclr_norm values and gene functions, genes related to domestication and significant selection were annotated in the genome-wide selection signal map (Fig. 4A), and the functions of these genes are detailed in Table S20. These functions were primarily categorized into mitochondrial function and cellular metabolism (*AIFM2*, *TIMM17B*, *CMC1*), apoptosis and cell proliferation (*XAF1*,* GPC5*, *MGA*, *PDSS2*), neurological development and signal transduction (*NAV2*, *DUSP22*,* OR52A1*), nutrient absorption (*CALCR*, *USO1*, *GLBIL2*, *TOR4A*, *ATP13A2*), transcription and expression regulation (*RPAP2*, *KMT2C*, *SP5*), immune function and stress response (*TRAP1*, *ITFG1*), and DNA repair (*SSBP2*).

Functional enrichment analysis of genes within the selected regions revealed significant pathways based on both KEGG (Fig. 4B and Table S21) and GO enrichment (Fig. 4C and Table S22). KEGG pathway analysis highlighted pathways involved in signal transduction, including the Ras signaling pathway, the PI3K/Akt signaling pathway, and the calcium signaling pathway. Additionally, pathways related to hormone function, such as growth hormone synthesis, secretion, and action, and adrenergic signaling in cardiomyocytes, were enriched. GO analysis revealed processes critical for development and cellular function, including nervous system development, ATP binding, cell development, protein kinase activity, and cell morphogenesis.

#### Selection for high-altitude adaptation

**Fig. 5:**
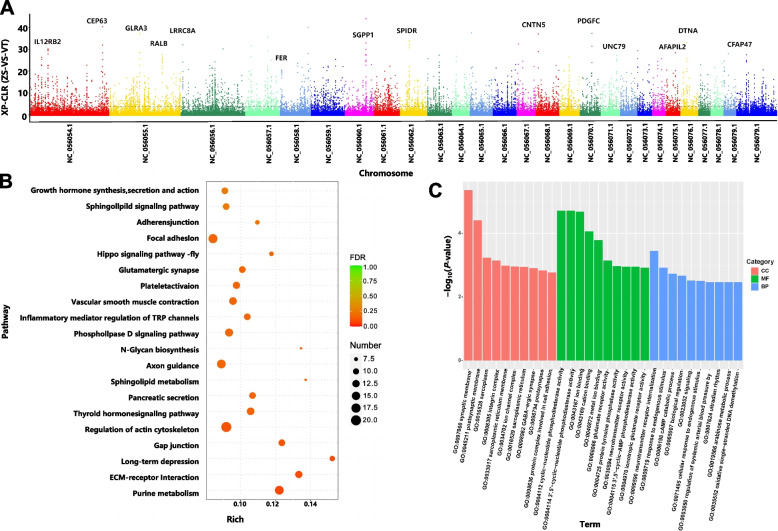
Selective elimination analysis of adaptation to Tibetan sheep. **A** Manhattan plot of genome-wide selective sweep signals in Zhashijia sheep (ZS) and Valley Tibetan sheep (VT). ZS distributed at an altitude of 4,300 m were used as the experimental group, and VT distributed at an altitude of 1,800 m were used as the control group. The horizontal axis represents the chromosomes, and the vertical axis represents the xpclr_norm values. Taking the first 1% of the xpclr_norm values (xpclr_norm ≥ 3.842473) as the selection criterion, the value is represented by a dashed line, which indicates the selective elimination region during the domestication process of Tibetan sheep. The text in the figure indicates genes associated with plateau adaptation with significant selection intensity. **B** KEGG functional enrichment of selectively eliminated genes. **C** GO functional enrichment of selectively downregulated genes

ZS, which inhabits the high-altitude (4,300 m) Qinghai‒Tibet Plateau region, was designated as the experimental group for genome-wide selective sweep analysis using the XPCLR method, with VT from the lower valley area (1,800 m) serving as the control group. XPCLR identified 5,788 selective elimination regions exceeding a predefined threshold (top 1%, xpclr_norm ≥ 3.842473), encompassing a total of 1,174 genes associated with altitude adaptation (Table S23). Based on the xpclr_norm values and gene functions, genes related to altitude adaptation and significant selection were annotated in the genome-wide selection signal map (Fig. 5A), which primarily included genes related to nervous system development (*GLRA3*,* CNTN5*, and *CEAP47*), cell division and cycle regulation (*CEP63*,* RALB*, and *SGPP1*), cell volume regulation (*LRRC8A*), immune function (*IL12RB2* and *FER*), angiogenesis (*PDGFC*), and DNA repair (*SPIDR*) (Table S24).

Functional enrichment analysis of genes identified in the altitude adaptation selective sweep regions revealed significant pathways based on both KEGG (Fig. 5B and Table S25) and GO analyses (Fig. 5C and Table S26). KEGG enrichment highlighted pathways involved in purine metabolism, ECM-receptor interactions, vascular smooth muscle contraction, platelet activation, and the thyroid hormone signaling pathway. GO enrichment analysis revealed processes relevant to adaptation, including oxidative RNA demethylase activity, biological regulation, the cAMP catabolic process, protein tyrosine phosphatase activity, and neurotransmitter receptor activity.

#### Joint analysis of unique SNPs and selective signals in Tibetan sheep

A joint analysis of unique SNPs and selective sweep results between Tibetan and Argali sheep was performed to elucidate the pivotal genes and pathways involved in Tibetan sheep domestication, which revealed a total of 429 genes (Table S27), 9 KEGG enrichment pathways (Table S28, *P* < 0.05), and 107 overlapping GO enrichment pathways (Table S29, *P* < 0.05). These findings highlighted selection in biological processes and signaling pathways, including signal transduction, carbohydrate digestion and absorption, estrogen secretion, platelet activation, and insulin secretion, during Tibetan sheep domestication and adaptation. These changes suggest that Tibetan sheep have modified traits related to signal sensing and transmission, nutrient digestion and absorption, and reproductive characteristics to suit the environmental conditions and meet the production requirements of the local population.

### RNA-seq analysis

Tibetan sheep have adapted to the hypoxic environment of high-altitude plateaus, with organs such as the heart and lungs playing critical roles in this adaptation [55, 56]. To investigate the underlying mechanisms of this adaptation, we conducted a transcriptomic analysis of heart and lung samples from four individuals each of two Tibetan sheep breeds, ZS and VT, which inhabit habitats with significant altitude differences. The average data yield per sample was 6.64 GB (SRR29500183–SRR29500198), with an average alignment rate of 97.43% with the reference genome (Table S30).

**Fig. 6:**
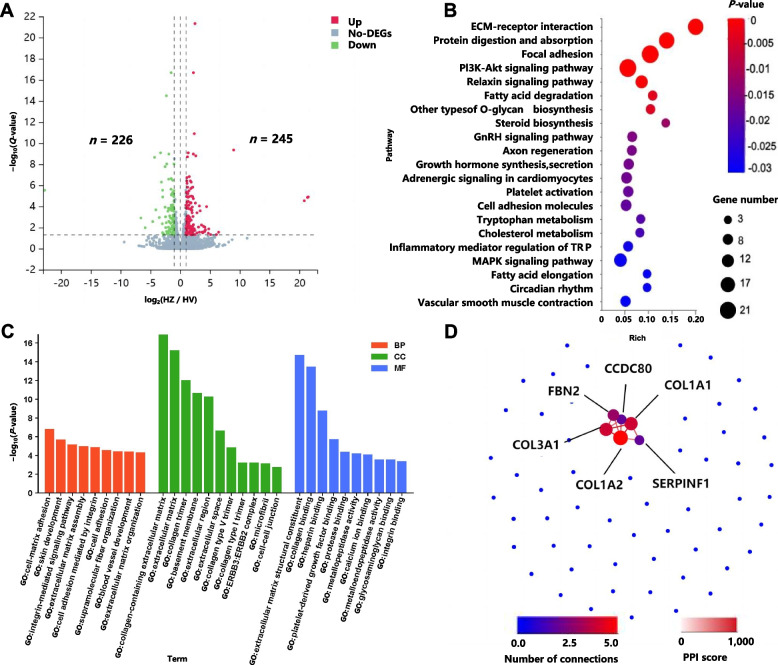
Heart transcriptome results in the high- and low-altitude groups Heart transcriptome results for the high-altitude (Zhashijia sheep, ZS) and low-altitude (Valley Tibetan sheep, VT) groups. **A** Volcano map of DEGs. HZ: Heart transcriptome of Zhashijia sheep; HV: Heart transcriptome of Valley Tibetan sheep. The *X*-axis represents the fold change in the difference after conversion to log_2_, and the *Y*-axis represents the significance value after conversion to log_10_. Red represents upregulated DEGs, blue represents downregulated DEGs, and gray represents non-DEGs. **B** Bubble map of enriched KEGG pathways. **C** Bar graph graphs of enriched GO pathways. **D** PPI network of DEGs (|log_2_fold-change| ≥ 2). The color of the circles represents the number of node connections, with redder colors indicating higher numbers of node connections. The color of the connection lines represents the interaction score, with redder colors indicating higher scores

Compared with the low-altitude VT group, the high-altitude ZS group presented 245 upregulated and 226 downregulated genes in the heart (Fig. 6A). Thse differentially expressed genes (DEGs) were enriched primarily in pathways associated with extracellular matrix–receptor interactions, protein digestion and absorption, signal transduction (PI3K/Akt signaling pathway and adrenergic signaling in cardiomyocytes), vascular smooth muscle contraction, and other processes (Fig. 6B and Table S31). GO analysis revealed significant enrichment in pathways related to blood vessel development, platelet-derived growth factor binding, protease binding, and basement membrane formation (Fig. 6C and Table S32). Seventy-two DEGs with an absolute log_2_ fold-change (|log_2_fold-change|) ≥ 2 (Table S33) were selected for protein‒protein interaction (PPI) network analysis. The results revealed a single interaction module consisting of 6 genes (*COL1A2*, *COL3A1*, *COL1A1*, *SERPINF1*,* FBN2*, and *CCDC80*) (Fig. 6D). Notably, *COL1A2*, *COL1A1*, and *COL3A1*, all members of the collagen gene family, displayed high connectivity within the network, suggesting a potentially crucial regulatory role within this module.

**Fig. 7:**
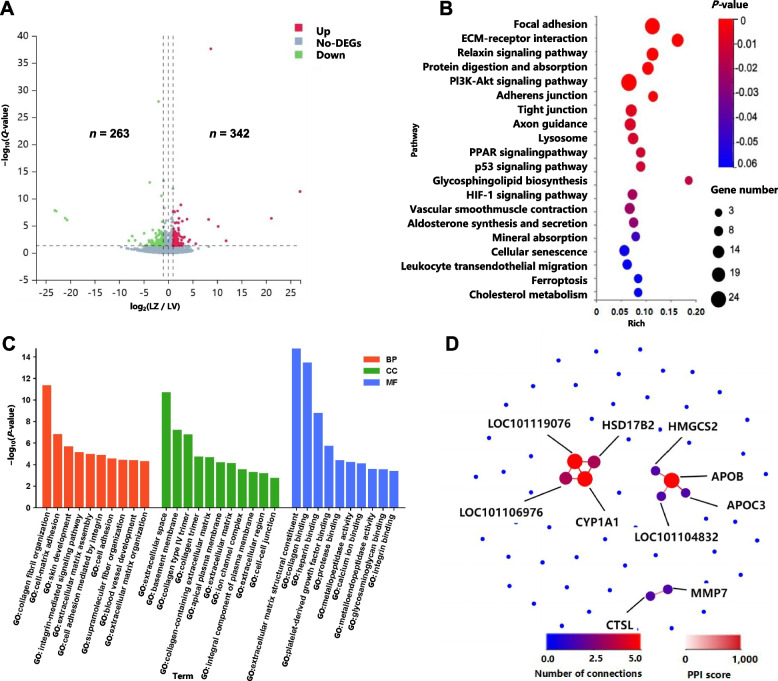
Lung transcriptome results in the high- and low-altitude groups. **A** Volcano plot of DEGs. LZ: Lung transcriptome of Zhashijia sheep; HV: Lung transcriptome of Valley Tibetan sheep.The *X*-axis represents the fold change in the difference after conversion to log_2_, and the *Y*-axis represents the significance value after conversion to log_10_. Red represents upregulated DEGs, blue represents downregulated DEGs, and gray represents non-DEGs. **B** Bubble map of enriched KEGG pathways. **C** Bar graph of enriched GO pathways. **D** PPI network of DEGs (|log_2_fold-change| ≥ 2). The color of the circles represents the number of node connections, with redder colors indicating higher numbers of node connections. The color of the connection lines represents the interaction score, with redder colors indicating higher scores

The lung transcriptome analysis of ZS revealed 605 DEGs compared with those of VT, with 342 upregulated and 263 downregulated genes (Fig. 7A). KEGG pathway enrichment of these DEGs highlighted pathways associated with extracellular matrix‒receptor interactions, vascular smooth muscle contraction, the PI3K/Akt signaling pathway, and protein digestion and absorption, among others (Fig. 7B and Table S34). Similarly, GO analysis (Fig. 7C and Table S35) revealed enrichment in processes such as platelet-derived growth factor binding, lung lobe morphogenesis, and calcium ion binding. Seventy DEGs with an absolute log_2_ fold-change (|log_2_fold-change|) ≥ 2 (Table S36) were selected for PPI network analysis. This analysis identified 3 distinct interaction modules (Fig. 7D). Notably, within one module, *L0C101119076*, *CYP1A1*, and *APOB* displayed high connectivity, suggesting a potentially significant regulatory role in this biological process.

### Combined analysis of the transcriptome and selective sweep in plateau adaptation

**Fig. 8:**
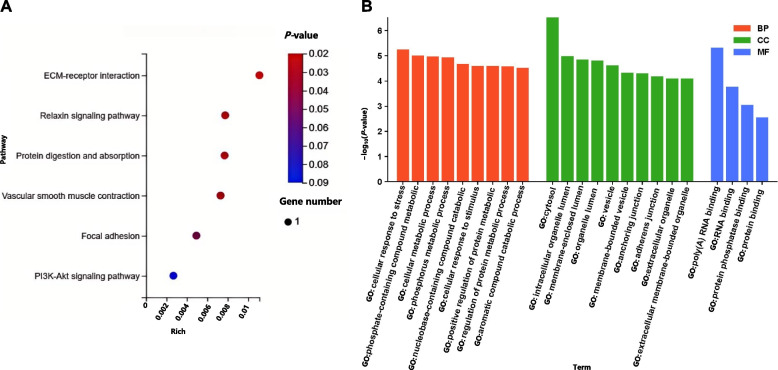
Functional enrichment analysis of common differentially expressed genes in selecte sweep regions, the heart transcriptome, and the lung transcriptome. **A** Bubble map of enriched KEGG pathways. **B** Bar graphs of enriched GO terms

Five genes (*RAMP1*, *PTPN3*, *COL4A2*, *MXRA5*, and *ADAMTS2*) were consistently differentially expressed across the selected sweep regions and the heart, and lung transcriptome data in the ZS vs. VT comparison (Table S37). These genes are potential candidate genes for adaptation to high altitudes. KEGG enrichment analysis of these genes revealed pathways associated with G protein-coupled receptor signaling, relaxin signaling, protein digestion and absorption, vascular smooth muscle contraction, and focal adhesion (Fig. 8A and Table S37). GO enrichment analysis highlighted pathways involved in the cellular response to stress, cellular metabolic processes, response to stimuli, and positive regulation of protein metabolism (Fig. 8B and Table S38). These pathways likely represent crucial regulatory mechanisms by which Tibetan sheep adapt to the high-altitude plateau environments.

## Discussion

### Structure and genetic relationship analysis of Tibetan sheep

The scholarly work ‘Chinese Livestock and Poultry Genetic Resources (2011–2020)’ proposes that EL sheep originated from a hybridization event between Argali and Tibetan sheep during the Yuan Dynasty [57]. Notably, Argali and EL sheep share significant morphological similarities in terms of horn shape, fur length, coat color, and body size. Given these similarities and the proposed ancestry, Argali sheep were chosen as the outgroup to investigate the genetic relationships between 6 Tibetan sheep breeds (PT, VT, EL, ZS, ZK, and GB), with emphasis on the genetic and evolutionary connections between EL and Argali sheep. In this study, phylogenetic tree analysis, genetic structure analysis, PCA, and *F*_ST_ values (*F*_ST_ > 0.25) collectively revealed a significant genetic differentiation between Argali and Tibetan sheep, which was consistent with the results of Li et al. [58]. Additionally, IBS analysis indicated that the genetic distance between EL and PA sheep was the greatest (IBS = 0.351), suggesting that Argali sheep might not be the sole direct ancestor of EL sheep.

PCA analysis showed that ZK, PT, GB, VT populations could not be clearly distinguished, and the *F*_ST_ values between the populations were all below 0.05. However, the 6 Tibetan sheep breeds exhibited clear segregation in the phylogenetic tree. The results indicated that the genetic relationships of the 6 Tibetan sheep breeds are relatively close and could not be clearly distinguished, but there was a trend of differentiation. Furthermore, genetic structures analysis revealed that when the ancestral ratio K = 4, the 6 Tibetan sheep breeds could be broadly grouped into 3 categories: GB, PT, ZS, and ZK sheep predominantly originated from a shared ancestral group (yellow), whereas EL and VT sheep primarily traced back to the blue and red ancestral groups, respectively. The results are consistent with previous studies that Tibetan sheep can be divided into 3 different ecological types: plateau type, valley type, and Euler type [23]. In particular, the GB, PT, ZS and ZK sheep all classified as plateau-type Tibetan sheep, but the genetic relationships among them are not obvious. These phenomena may be caused by the brief period of differentiation, or increase in hybridization resulting from geographical proximity, and this study have also found that there are extensive gene flows in these populations [59].

In conclusion, this study showed that Tibetan sheep and Argali sheep were differentiated at the same time, and the genetic relationships among the 6 Tibetan sheep were very close, with evidence of gene flow, although clear segregation trends were observed in the phylogenetic tree. In addition, the 6 Tibetan sheep breeds can be divided into 3 categories: plateau type, valley type, and Euler type, which is consistent with the ecological type classification of Tibetan sheep. This study provides an important scientific basis for understanding the genetic background and evolution of Tibetan sheep.

### Artificial selection of Tibetan sheep

Sheep (*Ovis aries*) were the first documented domesticated grazing animal [60]. Since their domestication, sheep have played vital roles in human societies and are valued for their wool and meat production, and their economic and cultural significance extends worldwide [61, 62]. On the Qinghai‒Tibet Plateau, Tibetan sheep constitute a cornerstone of the local economy, and studying the mechanisms behind their domestication can provide valuable empirical evidence. This knowledge can enhance our understanding of domestication selection patterns in Tibetan sheep, elucidate the genetic basis for breed-specific characteristics, and ultimately inform the development of molecular breeding strategies to improve economically important traits in sheep.

In this study, compared with those in Argali (wild sheep), the unique SNPs and selective sweep regions in Tibetan sheep were enriched in taste and signal transduction pathways (olfactory transduction, taste transduction, gustatory transduction, and cholinergic synapses), and the signal transduction-related genes *NAV2* and *DUSP22* and the taste-related gene *OR52A1* were strongly selected [63–65]. It has been shown that domestic sheep have a 28% reduction in olfactory-related brain regions and a 40% reduction in the limbic system compared to their wild ancestors [66]. These results suggest that domestication frequently leads to alterations in the sensory functions of domesticated animals. This phenomenon can be attributed to the provision of protection and sustenance by humans, which diminishes the necessity for acute sensory abilities to locate food and detect predators [67]. Furthermore, these changes conducive to human proximity and domestication. Additionally, the reduction in sensory system energy expenditure may allow for the reallocation of energy towards growth and reproductive processes in domesticated species [68].

Tibetan sheep also exhibited enrichment in pathways related to carbohydrate digestion and absorption, adipocytokine signaling, insulin secretion, gastric acid secretion, and other metabolic processes within their unique SNPs and selective sweep regions. Notably, these selective regions harbored genes under strong selection pressure that were involved in calcium absorption regulation (*CALCR*), protein transport (*USO1*), glucose metabolism (*GLBIL2*), energy and protein metabolism (*TOR4A*) [69–72]. This enrichment was likely attributable to the dietary transition from wild grasses to human-provided fodder, such as cereals and hay, during the domestication process [73, 74].

Furthermore, the enrichment of pathways involved in the secretion and synthesis of growth hormone, thyroid hormone, parathyroid hormone, and estrogen during the domestication of Tibetan sheep implies that these traits were deliberately selected for their advantageous growth and reproductive features. The selection for increased reproductive and growth output is also widely recognized as a key objective and outcome of livestock animal domestication [75].

In conclusion, this study demonstrated that the sensory function, nervous system, digestive system, metabolic ability, growth and development of Tibetan sheep were significantly changed compared with those of wild sheep during the domestication process. These changes reflect the tendency of farmers to select traits that can improve the adaptability and growth rate, and are more suitable for human feeding of Tibetan sheep. In addition, these genetic changes not only reflect the demand for production performance and environmental adaptability, but also reflect the pursuit of economic value and feeding convenience of Tibetan sheep.

### Molecular basis of high-altitude adaptation in Tibetan sheep

High-altitude adaptation is a growing field of research aimed at understanding the molecular mechanisms underlying this unique physiological response. Studies have shown that hemoglobin concentrations in Tibetans increase only at altitudes surpassing 4,000 m above sea level. Furthermore, Tibetans living at 3,900 m have hemoglobin concentrations approximately 21% lower than those of Andeans, despite both populations residing in plateau regions [76]. These findings suggest that Tibetans residing at various altitudes on the plateau have different mechanisms of adaptation to high-altitude environments. In animal research, studies on high-altitude adaptation typically compare species living at 2,000–3,000 m with their sea-level counterparts. However, research on the adaptation mechanisms of animals at varying altitudes on the plateau plateaus was scarce. This study addresses this gap by employing Tibetan sheep as a model. We utilized two populations: a high-altitude group (ZS) residing at 4,300 m and a low-altitude group (VT) residing at 1,800 m. This design allowed us to explore the high-altitude adaptation mechanisms of Tibetan sheep across this significant altitude range within the plateau environment.

Selective sweep analysis of Tibetan sheep from the high-altitude (ZS) and low-altitude (VT) groups revealed enrichment of genes involved in sphingolipid metabolism and phospholipase D signaling pathways were strongly selected. Notably, the differentially expressed genes identified in the lung and heart transcriptomes were enriched in pathways related to fat metabolism. *APOB* is the central gene in the PPI network module and downregulated in the lung tissue of high-altitude Tibetan sheep, and this gene involved primarily in lipid metabolism and plays an significant role in cholesterol metabolism and cardiovascular health [77]. These results suggest that the regulation of lipid digestion and metabolism plays a crucial role in Tibetan sheep adaptation to the plateau environment. This downregulated of lipid metabolism may be ascribed to a preferential shift towards more efficient energy pathways at high altitude environment, such as glycolysis and oxidative phosphorylation [78, 79]. This shift allows Tibetan sheep to increase their overall energy utilization efficiency while potentially reducing their reliance on lipids. Furthermore, lipid metabolism results in increased levels of oxidative byproducts, which can exacerbate oxidative stress under hypoxic conditions. Therefore, the inhibition of this pathway may serve as a protective mechanism [80]. *CYPA1*, another hub gene in the PPI network of DEGs in the lung transcriptome, mediates the production of reactive oxygen species (ROS) during its enzymatic processes. The downregulation of *CYPA1* expression can reduce ROS production, thereby mitigating oxidative stress [81].

In addition to metabolic regulation, humoral regulation is another significant factor in altitude adaptation. Selective sweep analysis comparing sheep residing at low altitudes (VT) with those residing at high altitudes (ZS) revealed significant enrichment of strongly selected genes in the aldosterone and renin secretion pathways. Additionally, the *LRRC8A* gene, which plays a crucial role in the regulation of cell volume, is also subject to strong selective pressures [82]. Furthermore, enrichment analysis of DEGs in the lung and cardiac transcriptomes revealed significant involvement of the relaxin signaling pathway, which is implicated in humoral regulation through the modulation of the glomerular filtration rate [83]. Studies have shown that adjustments in kidney function at high altitudes are crucial for maintaining fluid balance [84]. Upon initial exposure, the body rapidly reduces its plasma volume as an acclimatization response to hypoxia [85]. As adaptation progresses, the kidneys take over, regulating fluid balance by controlling sodium and water excretion. These adjustments involve changes in the glomerular filtration rate (GFR) and the regulation of hormone levels, including aldosterone, angiotensin, and antidiuretic hormone [86]. These findings suggest that humoral regulation, which involves complex renal functions and hormonal interactions, plays a critical role in the physiological adaptations of sheep to varying altitudes.

Cardiovascular function and angiogenesis are significant factors in acclimatization to high altitudes [87]. The results shown that the genes in the selective sweep region were significantly enriched in the vascular smooth muscle contraction pathway, and *FDGFC*, a regulator of angiogenesis, was also strongly selected [88]. Additionally, DEGs in the heart transcriptome were enriched in the relaxin signaling and adrenergic signaling pathways in cardiocytes. Furthermore, *COL1A2*, *COL1A1*, and *COL3A1* genes, which are members of the collagen family and serve as key component of connective tissue, shown upregulated expression [89]. Connective tissue plays a vital role in preserving the structural and mechanical integrity of cardiac tissue, thereby ensuring stable and powerful contraction and relaxation to promote oxygen delivery in the hypoxic environment [90]. Interestingly, *RAMP1*, *COL4A2*, and *ADAMTS2*, which were all differentially expressed in the selective sweep analysis of the lung and heart transcriptomes, were related to vascular function and connective tissue [89, 91, 92]. These results suggested that Tibetan sheep residing at high altitudes exhibit increased oxygen transport under hypoxic conditions through increased vascular smooth muscle function and cardiac contractility, which was consistent with previous studies that shown that the body increases blood pressure and increases oxygen supply through vascular smooth muscle [93]. This vasoconstrictive response is primarily mediated by the activation of the renin‒angiotensin‒aldosterone system (RAAS) and the sympathetic nervous system [94]. The low-oxygen environment also prompts the body to stimulate angiogenesis, leading to the formation of new blood vessels for improved oxygen delivery [95]. These results suggest that Tibetan sheep living at higher altitudes adapt to the plateau environment by increasing vascular smooth muscle function and cardiac contractility to increase oxygen transport compared to Tibetan sheep living at lower altitudes of the plateau. There are different from the plateau adaptation mechanism of plateau animals and lowland animals, which focuses on increasing the level of hemoglobin for oxygen delivery to adapt to the plateau environment [15].

In addition to cardiovascular function and angiogenesis, immune function, the stress response, cell division, and protein absorption and metabolism were also identified as significant factors influencing Tibetan sheep adaptation to high-altitude environments. In conclusion, compared with their counterparts at lower altitudes, Tibetan sheep inhabiting high-altitude regions exhibit adaptations that prioritize enhancing cardiopulmonary function, regulating body fluid balance through kidney reabsorption, and altering nutrient digestion and absorption pathways to meet the challenges of the plateau environment.

## Conclusion

In this study, we analysed the genomic variations, population structures, differentiation, genetic distance, and gene flow in 6 Tibetan sheep breeds provided a comprehensive understanding of their phylogenetic relationships. Additionally, this study demonstrated that significant changes in the sensory function, nervous system, digestive system, metabolic capacity, and growth of Tibetan sheep compared to wild sheep during domestication. Notably, our research revealed that Tibetan sheep adapt to high-altitude environments by enhancing cardiopulmonary function, regulating fluid balance through renal reabsorption, and modifying nutrient digestion and absorption pathways. This research provides valuable insights into the evolutionary processes that have enabled Tibetan sheep to thrive in extreme environments. The findings will also contribute to the preservation of genetic diversity and offer a foundation for future studies on environmental adaptation and biodiversity conservation in high-altitude species.

## Supplementary Information

Additional file 1. Whole genome and transcriptome sequencing and analysis results. **Table S1.** The information of sequencing sample and download data. **Table S2.** The statistical results of of sequencing data results. **Table S3.** The statistical results of high-quality sequencing data. **Table S4.** The results of the read mapping. **Table S5.** The statistical results of population SNPs in each sample. **Table S6.** The results of SNPs annotation. **Table S7.** The statistical results of population InDels in each sample. **Table S8.** The results of InDels annotation. **Table S9.** The results of IBS similarity value. **Table S10.** The results of genetic diversity in each population. **Table S11.** The statistical results of *F*_ST_ in population. **Table S12.** The statistical results of unique SNPs in Agrali and Tibetan sheep, separately (partial results). **Table S13.** The annotation results of unique SNPs in Argali (partial results). **Table S14.** The annotation results of unique SNPs in Tibetan sheep (partial results). **Table S15.** The KEGG enrichment results of unique SNPs in Argali (*P* < 0.05). **Table S16.** The GO enrichment results of unique SNPs in Argali (*P* < 0.05). **Table S17.** The KEGG enrichment results of unique SNPs in Tibetan sheep (*P* < 0.05). **Table S18.** The GO enrichment results of unique SNPs in Tibetan sheep (*P* < 0.05). **Table S19.** The gene annotation of the top 1% in XP-CLR analysis of Tibetan sheep and Argali (partial results). **Table S20.** The key genes associated with evolution in selected regions. **Table S21.** The KEGG enrichment analysis of genes in selective sweep regions of Tibetan sheep and Argali (*P* < 0.05). **Table S22.** The GO enrichment analysis of genes in selective sweep regions of Tibetan sheep and Argali (*P* < 0.05). **Table S23.** The gene annotation of the top 1% in XP-CLR analysis of Zhashijia sheep and Valley Tibetan sheep (partial results). **Table 24.** The key genes associated with altitude adaptation in selected regions. **Table S25.** The KEGG enrichment analysis of genes in selective sweep regions of Zhashijia sheep and Valley Tibetan sheep (*P* < 0.05). **Table S26.** The GO enrichment analysis of genes in selective sweep regions of Zhashijia sheep and Valley Tibetan sheep (*P* < 0.05). **Table S27.** The common genes in unique SNPs and the selective sweep region of Tibetan sheep relative to Argali. **Table S28.** The KEGG enrichment pathways shared by unique SNPs and the selective sweep region of Tibetan sheep relative to Argali (*P* < 0.05). **Table S29.** The GO pathways shared by unique SNPs and the selective sweep region of Tibetan sheep relative to Argali (*P* < 0.05). **Table S30.** The results of transcriptome in heart and lung tissues for Zhashijia sheep and Valley Tibetan sheep. **Table S31.** The KEGG enrichment of differentially expressed genes in heart transcriptome for Zhashijia sheep and Valley Tibetan sheep (*P* < 0.05). **Table S32.** The GO enrichment of differentially expressed genes in heart transcriptome for Zhashijia sheep and Valley Tibetan sheep (*P* < 0.05). **Table S33.** The genes were significantly differentially expressed in the heart transcriptome for Zhashijia sheep and Valley Tibetan sheep (|log_2_ (HJ/HS)| ≥ 2). **Table S34.** The KEGG enrichment of differentially expressed genes in lung transcriptome for Zhashijia sheep and Valley Tibetan sheep (*P* < 0.05). **Table S35.** The GO enrichment of of differentially expressed genes in lung transcriptome for Zhashijia sheep and Valley Tibetan sheep (*P* < 0.05). **Table S36.** The genes were significantly differentially expressed in the lung transcriptome for Zhashijia sheep and Valley Tibetan sheep (|log_2_ (LJ/LS)| ≥ 2). **Table S37.** The common differentially expressed genes in selective sweep of plateau adaptation, heart transcriptome and lung transcriptome. **Table S38.** The KEGG enrichment of common differentially expressed genes in selective sweep, heart transcriptome and lung transcriptome (*P* < 0.05). **Table S39.** The GO enrichment of common differentially expressed genes in selective sweep, heart transcriptome and lung transcriptome (*P* < 0.05).

## Data Availability

All the data supporting the findings of this study are available within this article and its supplementary files. All the sequencing data reported in this study are available upon request for research purposes.

## Abbreviations

EGLN1: Egl-9 family hypoxia inducible factor 1
EL: Euler Tibetan sheep
EPAS1: Endothelial PAS domain protein 1
*F*_IS_: Inbreeding coefficients
*F*_ST_: Population differentiation
GB: Guinan black fur sheep
GO: Gene Ontology
He: Expected heterozygosity
Ho: Observed heterozygosity
HQ reads: High-quality reads
IBS: Identical by state
InDel: Insertions/deletions
KEGG: Kyoto Encyclopedia of Genes and Genomes
LD: Linkage disequilibrium
PBS: Phosphate-buffered saline
PCA: Principal component analysis
Pi: Nucleotide diversity
PPI: Protein‒protein interaction
PT: Plateau Tibetan sheep
RAAS: Renin-angiotensin-aldosterone system
ROS: Reactive oxygen species
SNP: Single-nucleotide polymorphism
SNV: Synonymous single-nucleotide variants
TS: Tibetan sheep
Ts: Transitions
Tv: Transversions
VEGF: Vascular endothelial growth factor
VT: Valley Tibetan sheep
XR-CLR: Cross-population composite likelihood ratio test
ZK: Zeku sheep
ZS: Zhashijia sheep
