# Supplementary material for: Whole-genome resequencing to investigate the genetic diversity and mechanisms of plateau adaptation in Tibetan sheep
Source: J Anim Sci Biotechnol. 2024 Dec 6;15:164. doi: 10.1186/s40104-024-01125-1 (PMC11622566; doi:10.1186/s40104-024-01125-1)
Supplement: Supplementary file 1 — Additional file 1. Whole genome and transcriptome sequencing and analysis results. Table S1. The information of sequencing sample and download data. Table S2. The statistical results of of sequencing data results. Table S3. The statistical results of high-quality sequencing data. Table S4. The results of the read mapping. Table S5. The statistical results of population SNPs in each sample. Table S6. The results of SNPs annotation. Table S7. The statistical results of population InDels in each sample. Table S8. The results of InDels annotation. Table S9. The results of IBS similarity value. Table S10. The results of genetic diversity in each population. Table S11. The statistical results of FST in population. Table S12. The statistical results of unique SNPs in Agrali and Tibetan sheep, separately (partial results). Table S13. The annotation results of unique SNPs in Argali (partial results). Table S14. The annotation results of unique SNPs in Tibetan sheep (partial results). Table S15. The KEGG enrichment results of unique SNPs in Argali (P < 0.05). Table S16. The GO enrichment results of unique SNPs in Argali (P < 0.05). Table S17. The KEGG enrichment results of unique SNPs in Tibetan sheep (P < 0.05). Table S18. The GO enrichment results of unique SNPs in Tibetan sheep (P < 0.05). Table S19. The gene annotation of the top 1% in XP-CLR analysis of Tibetan sheep and Argali (partial results). Table S20. The key genes associated with evolution in selected regions. Table S21. The KEGG enrichment analysis of genes in selective sweep regions of Tibetan sheep and Argali (P < 0.05). Table S22. The GO enrichment analysis of genes in selective sweep regions of Tibetan sheep and Argali (P < 0.05). Table S23. The gene annotation of the top 1% in XP-CLR analysis of Zhashijia sheep and Valley Tibetan sheep (partial results). Table 24. The key genes associated with altitude adaptation in selected regions. Table S25. The KEGG enrichment analysis of genes in selective sweep regions [file 40104_2024_1125_MOESM1_ESM.doc]

**The supplementary file includes:** Tables S1 to S39

SI References

**Table S1** The information of sequencing sample and download data

| **Sample** | **Population** | **Longitude (°)** | **Latitude (°)** | **Altitude (m)** |
| --- | --- | --- | --- | --- |
| PA01-10 | PA | 75.99 | 39.47 | 2,000 |
| GB01-20 | GB | 100.75 | 35.59 | 3,100 |
| PT01-13 | PT | 99.02 | 37.3 | 3,300 |
| ZS01-19 | ZS | 95.8 | 34.13 | 4,300 |
| ZK01-20 | ZK | 101.47 | 35.03 | 3,600 |
| EL01-20 | EL | 101.61 | 34.74 | 3,500 |
| VT01-20 | VT | 101.96 | 36.84 | 1,800 |

Sample: Sample name. PA: Argali. GB: Guide black fur sheep. PT: Plateau Tibetan sheep. ZS: Zhashijia sheep. ZK: Zeku sheep. EL: Euler tibetan Sheep. VT: Valley Tibetan sheep

**Table S2** The statistical results of sequencing data results

| **Sample** | **Reads Num** | **Total bases (bp)** | **N(%)** | **GC(%)** | **Q20(%)** | **Q30(%)** | **Sequencing depth** |
| --- | --- | --- | --- | --- | --- | --- | --- |
| PA01 | 200000000 | 30000000000 | 0.02 | 44.62 | 93.23 | 85.88 | 11.15 |
| PA02 | 200000000 | 30000000000 | 0.02 | 44.90 | 95.08 | 88.75 | 10.32 |
| PA03 | 200000000 | 30000000000 | 0.01 | 44.04 | 94.29 | 87.64 | 11.07 |
| PA04 | 200000000 | 30000000000 | 0.01 | 45.52 | 95.10 | 88.89 | 10.78 |
| PA05 | 200000000 | 30000000000 | 0.01 | 43.79 | 95.52 | 89.82 | 10.79 |
| PA06 | 200000000 | 30000000000 | 0.01 | 45.23 | 97.68 | 94.31 | 11.37 |
| PA07 | 200000000 | 30000000000 | 0.01 | 45.29 | 93.62 | 85.84 | 9.39 |
| PA08 | 200000000 | 30000000000 | 0.01 | 44.02 | 96.69 | 92.62 | 11.60 |
| PA09 | 200000000 | 30000000000 | 0.01 | 41.85 | 97.27 | 93.66 | 10.06 |
| PA10 | 200000000 | 30000000000 | 0.02 | 43.09 | 95.88 | 90.92 | 11.37 |
| GB01 | 182651750 | 27580414250 | 0.00 | 45.48 | 97.51 | 93.55 | 10.86 |
| GB02 | 205488816 | 31028811216 | 0.00 | 44.94 | 97.28 | 93.17 | 9.35 |
| GB03 | 244074022 | 36855177322 | 0.00 | 45.80 | 96.94 | 92.82 | 9.62 |
| GB04 | 249175144 | 37625446744 | 0.00 | 45.15 | 97.16 | 92.94 | 10.70 |
| GB05 | 155041278 | 23411232978 | 0.00 | 45.94 | 96.92 | 92.15 | 9.76 |
| GB06 | 188492708 | 28462398908 | 0.00 | 45.25 | 97.12 | 92.78 | 10.83 |
| GB07 | 197261426 | 29786475326 | 0.00 | 45.34 | 96.63 | 91.60 | 10.84 |
| GB08 | 194621644 | 29387868244 | 0.00 | 45.06 | 97.13 | 92.66 | 9.69 |
| GB09 | 160097312 | 24174694112 | 0.00 | 45.31 | 97.15 | 92.62 | 11.42 |
| GB10 | 194069498 | 29304494198 | 0.00 | 44.82 | 97.05 | 92.43 | 11.42 |
| GB11 | 189966206 | 28684897106 | 0.00 | 45.88 | 96.77 | 92.13 | 11.42 |
| GB12 | 177475236 | 26798760636 | 0.00 | 45.55 | 97.01 | 92.40 | 11.42 |
| GB13 | 172604138 | 26063224838 | 0.00 | 45.16 | 97.13 | 92.52 | 11.42 |
| GB14 | 188365022 | 28443118322 | 0.00 | 45.16 | 97.24 | 92.69 | 11.42 |
| GB15 | 195636030 | 29541040530 | 0.00 | 45.48 | 97.15 | 92.45 | 11.42 |
| GB16 | 173975894 | 26270359994 | 0.00 | 45.59 | 96.98 | 92.41 | 11.42 |
| GB17 | 190042516 | 28696419916 | 0.00 | 46.00 | 96.56 | 91.70 | 11.42 |
| GB18 | 173942434 | 26265307534 | 0.00 | 45.23 | 96.79 | 91.85 | 11.42 |
| GB19 | 165686306 | 25018632206 | 0.00 | 45.56 | 97.06 | 92.48 | 11.50 |
| GB20 | 202708168 | 30608933368 | 0.00 | 45.14 | 97.02 | 92.36 | 10.72 |
| PT01 | 201466634 | 30219995100 | 0.00 | 44.44 | 97.54 | 93.36 | 10.90 |
| PT02 | 187750038 | 28162505700 | 0.00 | 44.81 | 97.77 | 94.09 | 11.13 |
| PT03 | 224226482 | 33633972300 | 0.00 | 45.28 | 97.52 | 93.56 | 12.59 |
| PT04 | 194963632 | 29244544800 | 0.00 | 44.61 | 97.43 | 93.19 | 9.74 |
| PT05 | 195191168 | 29278675200 | 0.00 | 45.42 | 97.55 | 93.6 | 10.17 |
| PT06 | 198954750 | 29843212500 | 0.00 | 44.89 | 97.16 | 92.54 | 9.26 |
| PT07 | 195819412 | 29568731212 | 0.00 | 45.2 | 97.73 | 93.71 | 9.44 |
| PT08 | 183795352 | 27569302800 | 0.00 | 44.72 | 97.44 | 93.29 | 9.22 |
| PT09 | 183054986 | 27458247900 | 0.00 | 44.71 | 97.73 | 93.89 | 9.20 |
| PT10 | 223920344 | 33588051600 | 0.00 | 45.04 | 97.43 | 93.27 | 11.78 |
| PT11 | 188057082 | 28208562300 | 0.00 | 44.69 | 97.81 | 94.03 | 11.41 |
| PT12 | 172320726 | 26020429626 | 0.00 | 45.7 | 96.85 | 91.99 | 11.48 |
| PT13 | 214753014 | 32212952100 | 0.00 | 44.33 | 97.65 | 93.67 | 12.38 |
| PT14 | 177242838 | 26763668538 | 0.00 | 44.66 | 97.03 | 92.32 | 9.58 |
| PT15 | 189049366 | 28546454266 | 0.00 | 45.3 | 96.97 | 92.33 | 12.56 |
| PT16 | 186656896 | 28185191296 | 0.00 | 44.59 | 96.4 | 90.93 | 11.31 |
| PT17 | 180922132 | 27319241932 | 0.00 | 44.83 | 96.84 | 91.89 | 13.85 |
| PT18 | 202213550 | 30534246050 | 0.00 | 44.76 | 96.91 | 92.02 | 13.03 |
| PT19 | 168948950 | 25511291450 | 0.00 | 45.31 | 96.8 | 91.88 | 13.19 |
| PT20 | 191375862 | 28706379300 | 0.00 | 44.62 | 97.58 | 93.54 | 11.33 |
| ZS01 | 192068650 | 29002366150 | 0.00 | 44.29 | 96.84 | 91.58 | 12.40 |
| ZS02 | 193646698 | 29240651398 | 0.00 | 44.14 | 97.18 | 92.38 | 11.12 |
| ZS03 | 195269858 | 29290478700 | 0.00 | 45.17 | 97.16 | 92.53 | 11.11 |
| ZS04 | 180827908 | 27124186200 | 0.00 | 44.4 | 97.55 | 93.45 | 10.73 |
| ZS05 | 192598570 | 29082384070 | 0.00 | 44.25 | 96.93 | 91.73 | 11.81 |
| ZS06 | 187642840 | 28334068840 | 0.00 | 44.61 | 96.81 | 91.62 | 11.20 |
| ZS07 | 188968228 | 28345234200 | 0.00 | 44.67 | 97.6 | 93.59 | 12.32 |
| ZS08 | 197902748 | 29883314948 | 0.00 | 45.1 | 97.21 | 92.5 | 11.76 |
| ZS09 | 163470000 | 24683970000 | 0.00 | 44.92 | 97.32 | 92.81 | 11.36 |
| ZS10 | 201973824 | 30498047424 | 0.00 | 44.43 | 96.96 | 91.86 | 10.74 |
| ZS11 | 175146680 | 26447148680 | 0.00 | 45.37 | 97.13 | 92.42 | 10.38 |
| ZS12 | 197864686 | 29877567586 | 0.00 | 44.63 | 97.02 | 92.05 | 12.45 |
| ZS13 | 189637188 | 28635215388 | 0.00 | 45.13 | 96.79 | 91.58 | 11.68 |
| ZS14 | 193777688 | 29260430888 | 0.00 | 44.94 | 97.09 | 92.24 | 11.90 |
| ZS15 | 219142042 | 33090448342 | 0.00 | 44.97 | 96.84 | 91.68 | 11.70 |
| ZS16 | 170704118 | 25605617700 | 0.00 | 44.62 | 97.3 | 92.84 | 11.13 |
| ZS17 | 178100246 | 26715036900 | 0.00 | 44.83 | 97.7 | 93.91 | 11.74 |
| ZS18 | 162313356 | 24347003400 | 0.00 | 44.62 | 97.62 | 93.6 | 12.28 |
| ZS19 | 165477264 | 24821589600 | 0.00 | 45.88 | 97.43 | 93.33 | 11.82 |
| ZS20 | 161587254 | 24238088100 | 0.00 | 44.88 | 97.55 | 93.53 | 11.18 |
| ZK01 | 186632706 | 28181538606 | 0.00 | 45.50 | 97.02 | 92.52 | 12.80 |
| ZK02 | 189821184 | 28662998784 | 0.00 | 46.10 | 96.55 | 92.00 | 11.13 |
| ZK03 | 166795392 | 25186104192 | 0.00 | 45.06 | 96.99 | 92.35 | 11.14 |
| ZK04 | 194327180 | 29343404180 | 0.01 | 45.71 | 97.13 | 92.99 | 11.36 |
| ZK05 | 180665972 | 27280561772 | 0.01 | 45.45 | 97.52 | 93.67 | 11.25 |
| ZK06 | 213886584 | 32296874184 | 0.01 | 46.28 | 96.93 | 92.96 | 10.49 |
| ZK07 | 211815694 | 31984169794 | 0.01 | 45.37 | 97.29 | 93.21 | 10.45 |
| ZK08 | 164528882 | 24843861182 | 0.01 | 45.91 | 97.33 | 93.53 | 12.78 |
| ZK09 | 180071956 | 27190865356 | 0.01 | 46.18 | 97.19 | 93.08 | 10.73 |
| ZK10 | 172946170 | 26114871670 | 0.01 | 46.42 | 96.89 | 92.91 | 9.90 |
| ZK11 | 228453640 | 34496499640 | 0.00 | 46.40 | 96.72 | 92.43 | 12.26 |
| ZK12 | 221474636 | 33442670036 | 0.00 | 45.30 | 97.39 | 93.48 | 10.18 |
| ZK13 | 188964178 | 28533590878 | 0.00 | 45.29 | 97.51 | 93.56 | 10.86 |
| ZK14 | 162681388 | 24564889588 | 0.01 | 45.83 | 97.16 | 92.95 | 10.72 |
| ZK15 | 167516106 | 25294932006 | 0.01 | 46.02 | 96.97 | 92.67 | 10.40 |
| ZK16 | 186208326 | 28117457226 | 0.00 | 46.75 | 96.63 | 92.12 | 11.62 |
| ZK17 | 169837002 | 25645387302 | 0.01 | 45.69 | 97.27 | 93.27 | 9.71 |
| ZK18 | 188474076 | 28459585476 | 0.01 | 45.70 | 97.27 | 93.18 | 10.92 |
| ZK19 | 188724356 | 28497377756 | 0.01 | 45.47 | 97.22 | 93.01 | 11.04 |
| ZK20 | 168615918 | 25461003618 | 0.01 | 45.67 | 97.41 | 93.59 | 11.13 |
| EL01 | 161269390 | 24190408500 | 0.00 | 44.57 | 97.77 | 93.92 | 12.14 |
| EL02 | 204996996 | 30954546396 | 0.00 | 45.07 | 97.48 | 93.11 | 11.76 |
| EL03 | 199977282 | 29996592300 | 0.00 | 45.11 | 97.73 | 93.98 | 11.04 |
| EL04 | 199735136 | 30160005536 | 0.00 | 45.58 | 97.14 | 92.38 | 10.49 |
| EL05 | 215492014 | 32539294114 | 0.00 | 45.15 | 97.47 | 93.03 | 10.98 |
| EL06 | 167920444 | 25188066600 | 0.00 | 44.9 | 97.68 | 93.73 | 12.08 |
| EL07 | 218577092 | 33005140892 | 0.00 | 44.93 | 97.15 | 92.33 | 11.51 |
| EL08 | 198226442 | 29733966300 | 0.00 | 45.17 | 97.54 | 93.5 | 11.51 |
| EL09 | 241037868 | 36396718068 | 0.00 | 45.02 | 97.23 | 92.48 | 10.86 |
| EL10 | 226772978 | 34242719678 | 0.00 | 44.88 | 97.14 | 92.24 | 9.35 |
| EL11 | 229615304 | 34671910904 | 0.00 | 44.66 | 97 | 92.03 | 9.62 |
| EL12 | 197120862 | 29765250162 | 0.00 | 48.13 | 97.11 | 92.29 | 10.70 |
| EL13 | 215811182 | 32587488482 | 0.00 | 44.76 | 96.81 | 91.65 | 9.76 |
| EL14 | 193478082 | 29215190382 | 0.00 | 44.97 | 96.57 | 91.14 | 10.83 |
| EL15 | 194719312 | 29207896800 | 0.00 | 44.85 | 97.63 | 93.7 | 10.84 |
| EL16 | 186826630 | 28210821130 | 0.00 | 44.55 | 96.96 | 91.84 | 9.69 |
| EL17 | 205536802 | 31036057102 | 0.00 | 44.89 | 96.73 | 91.46 | 11.42 |
| EL18 | 194929870 | 29434410370 | 0.00 | 43.53 | 96.7 | 91.32 | 11.42 |
| EL19 | 214419194 | 32377298294 | 0.00 | 44.93 | 97.04 | 92.16 | 11.42 |
| EL20 | 204608896 | 30895943296 | 0.00 | 44.54 | 96.37 | 90.64 | 11.42 |
| VT01 | 199095272 | 29864290800 | 0.00 | 44.65 | 97.56 | 93.48 | 11.42 |
| VT02 | 188209136 | 28231370400 | 0.00 | 44.8 | 97.63 | 93.69 | 11.42 |
| VT03 | 181876974 | 27281546100 | 0.00 | 44.56 | 97.66 | 93.75 | 11.42 |
| VT04 | 218061252 | 32709187800 | 0.00 | 44.9 | 97.32 | 92.99 | 11.42 |
| VT05 | 204601996 | 30690299400 | 0.00 | 45.2 | 97.63 | 93.78 | 11.42 |
| VT06 | 208435538 | 31265330700 | 0.00 | 45.18 | 97.41 | 93.26 | 11.42 |
| VT07 | 204931864 | 30739779600 | 0.00 | 44.58 | 97.5 | 93.39 | 11.50 |
| VT08 | 194948988 | 29242348200 | 0.00 | 44.89 | 97.39 | 93.14 | 10.72 |
| VT09 | 205739588 | 30860938200 | 0.00 | 44.4 | 97.69 | 93.84 | 12.80 |
| VT10 | 213804074 | 32284415174 | 0.00 | 44.46 | 97.67 | 93.47 | 11.13 |
| VT11 | 207170458 | 31075568700 | 0.00 | 45.04 | 97.66 | 93.84 | 11.14 |
| VT12 | 195862878 | 29379431700 | 0.00 | 45.32 | 97.76 | 94.09 | 11.36 |
| VT13 | 212653012 | 31897951800 | 0.00 | 44.52 | 97.59 | 93.57 | 11.25 |
| VT14 | 206058638 | 30908795700 | 0.00 | 44.96 | 97.65 | 93.78 | 10.49 |
| VT15 | 193347570 | 29002135500 | 0.00 | 44.6 | 97.3 | 92.93 | 10.45 |
| VT16 | 183794840 | 27569226000 | 0.00 | 45.2 | 97.62 | 93.67 | 12.78 |
| VT17 | 192381856 | 28857278400 | 0.00 | 44.66 | 97.66 | 93.73 | 10.73 |
| VT18 | 211698306 | 31754745900 | 0.00 | 44.75 | 97.53 | 93.43 | 9.90 |
| VT19 | 201712624 | 30256893600 | 0.00 | 45.06 | 97.42 | 93.16 | 12.26 |
| VT20 | 201695722 | 30254358300 | 0.00 | 44.82 | 97.41 | 93.1 | 10.18 |
| Average | 193331436 | 29122003940 | 0.00 | 45.12 | 97.23 | 92.82 | 11.08 |

Sample: Sample name. Reads Num: Total number of reads. Total bases(bp): Total number of bases. N(%): Percentage of fuzzy bases. GC(%): GC content. Q20(%): Percentage of bases with base recognition accuracy above 99. Q30 (%): Percentage of bases with base recognition accuracy above 99.9

**Table S3 The statistical results of high-quality sequencing data**

| **Sample** | **HQ_Reads** | **HQ_Reads (%)** | **HQ_Data (bp)** | **HQ_Data (**%**)** |
| --- | --- | --- | --- | --- |
| PA01 | 164819042 | 82.41 | 22991293002 | 76.64 |
| PA02 | 175276112 | 87.64 | 24790635059 | 82.64 |
| PA03 | 177127494 | 88.56 | 24855439899 | 82.85 |
| PA04 | 181891572 | 90.95 | 25645083785 | 85.48 |
| PA05 | 174898964 | 87.45 | 25354379295 | 84.51 |
| PA06 | 184086650 | 92.04 | 27057197312 | 90.19 |
| PA07 | 172907816 | 86.45 | 24315737012 | 81.05 |
| PA08 | 183918320 | 91.96 | 26536721812 | 88.46 |
| PA09 | 176257400 | 88.13 | 25850927709 | 86.17 |
| PA10 | 173197114 | 86.60 | 24964196498 | 83.21 |
| GB01 | 174299538 | 95.43 | 25996319143 | 94.26 |
| GB02 | 194402430 | 94.60 | 28986829613 | 93.42 |
| GB03 | 228352808 | 93.56 | 34029953474 | 92.33 |
| GB04 | 235819094 | 94.64 | 35126838580 | 93.36 |
| GB05 | 147097660 | 94.88 | 21874982109 | 93.44 |
| GB06 | 178317134 | 94.60 | 26567746400 | 93.34 |
| GB07 | 185684422 | 94.13 | 27593979916 | 92.64 |
| GB08 | 184524856 | 94.81 | 27485285096 | 93.53 |
| GB09 | 152434160 | 95.21 | 22689309396 | 93.86 |
| GB10 | 183827222 | 94.72 | 27370664987 | 93.40 |
| GB11 | 178241868 | 93.83 | 26517208329 | 92.44 |
| GB12 | 168252676 | 94.80 | 25042073307 | 93.44 |
| GB13 | 164447538 | 95.27 | 24482333192 | 93.93 |
| GB14 | 179862134 | 95.49 | 26793425472 | 94.20 |
| GB15 | 186720048 | 95.44 | 27791897514 | 94.08 |
| GB16 | 164130934 | 94.34 | 24440315587 | 93.03 |
| GB17 | 176997544 | 93.14 | 26321897087 | 91.73 |
| GB18 | 164271760 | 94.44 | 24431949996 | 93.02 |
| GB19 | 157311126 | 94.95 | 23401944658 | 93.54 |
| GB20 | 192319366 | 94.87 | 28626195976 | 93.52 |
| PT01 | 194060136 | 96.32 | 28730307128 | 95.07 |
| PT02 | 181665066 | 96.76 | 26905834839 | 95.54 |
| PT03 | 215483522 | 96.10 | 31874605747 | 94.77 |
| PT04 | 187318822 | 96.08 | 27712308528 | 94.76 |
| PT05 | 187677230 | 96.15 | 27767211515 | 94.84 |
| PT06 | 190332358 | 95.67 | 28123647822 | 94.24 |
| PT07 | 190298644 | 97.18 | 28383670741 | 95.99 |
| PT08 | 176548912 | 96.06 | 26107704370 | 94.70 |
| PT09 | 177012066 | 96.70 | 26228307475 | 95.52 |
| PT10 | 214928620 | 95.98 | 31782567457 | 94.62 |
| PT11 | 182283260 | 96.93 | 27021857533 | 95.79 |
| PT12 | 164468874 | 95.44 | 24383476618 | 93.71 |
| PT13 | 207463798 | 96.61 | 30736829771 | 95.42 |
| PT14 | 169908938 | 95.86 | 25206115467 | 94.18 |
| PT15 | 180720660 | 95.59 | 26804602981 | 93.90 |
| PT16 | 176673834 | 94.65 | 26142501677 | 92.75 |
| PT17 | 172768558 | 95.49 | 25618760459 | 93.78 |
| PT18 | 193519646 | 95.70 | 28692089468 | 93.97 |
| PT19 | 161143272 | 95.38 | 23882880160 | 93.62 |
| PT20 | 184694198 | 96.51 | 27356912853 | 95.30 |
| ZS01 | 183666378 | 95.63 | 27260483701 | 93.99 |
| ZS02 | 186126238 | 96.12 | 27672050537 | 94.64 |
| ZS03 | 187424398 | 95.98 | 27664557974 | 94.45 |
| ZS04 | 174629206 | 96.57 | 25830934295 | 95.23 |
| ZS05 | 184508790 | 95.80 | 27403948107 | 94.23 |
| ZS06 | 179015352 | 95.40 | 26563822344 | 93.75 |
| ZS07 | 182388510 | 96.52 | 26980617622 | 95.19 |
| ZS08 | 190213490 | 96.11 | 28282417054 | 94.64 |
| ZS09 | 157473292 | 96.33 | 23415079329 | 94.86 |
| ZS10 | 193401330 | 95.76 | 28734766475 | 94.22 |
| ZS11 | 167684230 | 95.74 | 24916717071 | 94.21 |
| ZS12 | 189582204 | 95.81 | 28166591453 | 94.27 |
| ZS13 | 180915544 | 95.40 | 26842861068 | 93.74 |
| ZS14 | 185865346 | 95.92 | 27609961650 | 94.36 |
| ZS15 | 209307828 | 95.51 | 31063255502 | 93.87 |
| ZS16 | 163946870 | 96.04 | 24227305549 | 94.62 |
| ZS17 | 172042608 | 96.60 | 25457055488 | 95.29 |
| ZS18 | 156750016 | 96.57 | 23192840126 | 95.26 |
| ZS19 | 159064120 | 96.12 | 23510777410 | 94.72 |
| ZS20 | 155855448 | 96.45 | 23045632244 | 95.08 |
| ZK01 | 176126806 | 94.37 | 26227048712 | 93.06 |
| ZK02 | 175611208 | 92.51 | 26136552393 | 91.19 |
| ZK03 | 158042612 | 94.75 | 23522516020 | 93.39 |
| ZK04 | 182890116 | 94.11 | 27228450884 | 92.79 |
| ZK05 | 171612248 | 94.99 | 25574531226 | 93.75 |
| ZK06 | 199328904 | 93.19 | 29666507473 | 91.86 |
| ZK07 | 200484210 | 94.65 | 29843843906 | 93.31 |
| ZK08 | 155175118 | 94.31 | 23118398090 | 93.05 |
| ZK09 | 169637744 | 94.21 | 25255960463 | 92.88 |
| ZK10 | 161039912 | 93.12 | 23960641368 | 91.75 |
| ZK11 | 212685440 | 93.10 | 31652701846 | 91.76 |
| ZK12 | 210164770 | 94.89 | 31335250982 | 93.70 |
| ZK13 | 180329626 | 95.43 | 26892928591 | 94.25 |
| ZK14 | 153309440 | 94.24 | 22821481655 | 92.90 |
| ZK15 | 157192160 | 93.84 | 23374125815 | 92.41 |
| ZK16 | 173430598 | 93.14 | 25775143071 | 91.67 |
| ZK17 | 160361276 | 94.42 | 23875484513 | 93.10 |
| ZK18 | 178248260 | 94.57 | 26525732842 | 93.20 |
| ZK19 | 178412148 | 94.54 | 26553818428 | 93.18 |
| ZK20 | 159605370 | 94.66 | 23766040174 | 93.34 |
| EL01 | 156261590 | 96.89 | 23147827701 | 95.69 |
| EL02 | 198807662 | 96.98 | 29591025979 | 95.60 |
| EL03 | 193640950 | 96.83 | 28659448630 | 95.54 |
| EL04 | 192925056 | 96.59 | 28665557816 | 95.04 |
| EL05 | 209023260 | 97.00 | 31111674635 | 95.61 |
| EL06 | 162533312 | 96.79 | 24060279141 | 95.52 |
| EL07 | 211058266 | 96.56 | 31368999908 | 95.04 |
| EL08 | 191404030 | 96.56 | 28312204369 | 95.22 |
| EL09 | 233249194 | 96.77 | 34677058653 | 95.28 |
| EL10 | 219255468 | 96.69 | 32597277366 | 95.19 |
| EL11 | 219836174 | 95.74 | 32657158009 | 94.19 |
| EL12 | 188965404 | 95.86 | 28095649397 | 94.39 |
| EL13 | 205956042 | 95.43 | 30569041524 | 93.81 |
| EL14 | 183806220 | 95.00 | 27246246854 | 93.26 |
| EL15 | 188304136 | 96.71 | 27863124965 | 95.40 |
| EL16 | 178987696 | 95.80 | 26591998248 | 94.26 |
| EL17 | 195803984 | 95.26 | 29049875920 | 93.60 |
| EL18 | 185666496 | 95.25 | 27522879538 | 93.51 |
| EL19 | 205383274 | 95.79 | 30517109732 | 94.25 |
| EL20 | 193629908 | 94.63 | 28675650815 | 92.81 |
| VT01 | 192081744 | 96.48 | 28434593609 | 95.21 |
| VT02 | 181987932 | 96.69 | 26932556089 | 95.40 |
| VT03 | 175783098 | 96.65 | 26022019967 | 95.38 |
| VT04 | 209847506 | 96.23 | 31016339592 | 94.82 |
| VT05 | 197591466 | 96.57 | 29227575007 | 95.23 |
| VT06 | 200579426 | 96.23 | 29661270569 | 94.87 |
| VT07 | 197657958 | 96.45 | 29239148545 | 95.12 |
| VT08 | 187725922 | 96.29 | 27761924656 | 94.94 |
| VT09 | 198956022 | 96.70 | 29457644884 | 95.45 |
| VT10 | 207871796 | 97.23 | 31005184682 | 96.04 |
| VT11 | 200141186 | 96.61 | 29617757057 | 95.31 |
| VT12 | 189361386 | 96.68 | 28036582213 | 95.43 |
| VT13 | 205497310 | 96.64 | 30411412209 | 95.34 |
| VT14 | 199170460 | 96.66 | 29477167689 | 95.37 |
| VT15 | 185941490 | 96.17 | 27487619580 | 94.78 |
| VT16 | 177707666 | 96.69 | 26299162169 | 95.39 |
| VT17 | 186152684 | 96.76 | 27553869046 | 95.48 |
| VT18 | 204533548 | 96.62 | 30257492738 | 95.28 |
| VT19 | 194287782 | 96.32 | 28739897700 | 94.99 |
| VT20 | 194389302 | 96.38 | 28760594649 | 95.06 |
| Average | 184846648 | 95.60 | 27436350648 | 94.20 |

Sample: Sample name. HQ reads: Number of high-quality reads. HQ reads (%): Percentage of high-quality reads to original reads. HQ Bases (bp): Number of bases for high-quality reads. HQ Bases(%): Percentage of base number of high-quality reads to total number of original bases

**Table S4** The results of the read mapping

| **Sample** | **Total_reads** | **Mapped_reads** | **Mapping_rate (%)** |
| --- | --- | --- | --- |
| PA01 | 167875988 | 166874536 | 99.40 |
| PA02 | 178462525 | 177682285 | 99.56 |
| PA03 | 180087000 | 179428881 | 99.63 |
| PA04 | 184612539 | 183754315 | 99.54 |
| PA05 | 200346583 | 199440929 | 99.55 |
| PA06 | 220620578 | 220039142 | 99.74 |
| PA07 | 195643774 | 194862273 | 99.60 |
| PA08 | 225400739 | 224790669 | 99.73 |
| PA09 | 212850265 | 212216618 | 99.70 |
| PA10 | 210975230 | 210262684 | 99.66 |
| GB01 | 174663211 | 174481306 | 99.90 |
| GB02 | 194814833 | 194581901 | 99.88 |
| GB03 | 228824434 | 228335718 | 99.79 |
| GB04 | 236328353 | 235961904 | 99.84 |
| GB05 | 147405947 | 147255986 | 99.90 |
| GB06 | 178682096 | 178515013 | 99.91 |
| GB07 | 186086614 | 185855888 | 99.88 |
| GB08 | 184918866 | 184668510 | 99.86 |
| GB09 | 152769241 | 152602039 | 99.89 |
| GB10 | 184215133 | 183938990 | 99.85 |
| GB11 | 178652402 | 178153892 | 99.72 |
| GB12 | 168641781 | 168327227 | 99.81 |
| GB13 | 164839363 | 164504140 | 99.80 |
| GB14 | 180293395 | 179958682 | 99.81 |
| GB15 | 187159914 | 186649625 | 99.73 |
| GB16 | 164514976 | 164214036 | 99.82 |
| GB17 | 177402599 | 176906259 | 99.72 |
| GB18 | 164652753 | 164350095 | 99.82 |
| GB19 | 157694151 | 157185831 | 99.68 |
| GB20 | 192774973 | 192371616 | 99.79 |
| PT01 | 194510453 | 194192678 | 99.84 |
| PT02 | 182103796 | 181707672 | 99.78 |
| PT03 | 215993790 | 215495314 | 99.77 |
| PT04 | 187754437 | 187492438 | 99.86 |
| PT05 | 188124783 | 187625595 | 99.73 |
| PT06 | 190761346 | 190431856 | 99.83 |
| PT07 | 190717222 | 190250430 | 99.76 |
| PT08 | 176979491 | 176349501 | 99.64 |
| PT09 | 177431537 | 177134636 | 99.83 |
| PT10 | 215446802 | 214790243 | 99.70 |
| PT11 | 182665587 | 182419378 | 99.87 |
| PT12 | 164801665 | 164475155 | 99.80 |
| PT13 | 207901991 | 207654321 | 99.88 |
| PT14 | 170260125 | 170144855 | 99.93 |
| PT15 | 181081567 | 180899113 | 99.90 |
| PT16 | 177046550 | 176945509 | 99.94 |
| PT17 | 173119549 | 172886966 | 99.87 |
| PT18 | 193910674 | 193453761 | 99.76 |
| PT19 | 161477071 | 161288783 | 99.88 |
| PT20 | 185079939 | 184900479 | 99.90 |
| ZS01 | 184029201 | 183819325 | 99.89 |
| ZS02 | 186508876 | 186201926 | 99.84 |
| ZS03 | 187844015 | 187533041 | 99.83 |
| ZS04 | 175029218 | 174656911 | 99.79 |
| ZS05 | 184925922 | 184550026 | 99.80 |
| ZS06 | 179374119 | 178851014 | 99.71 |
| ZS07 | 182810174 | 182543769 | 99.85 |
| ZS08 | 190626674 | 190165856 | 99.76 |
| ZS09 | 157866384 | 157567424 | 99.81 |
| ZS10 | 193845108 | 193437917 | 99.79 |
| ZS11 | 168065833 | 167488663 | 99.66 |
| ZS12 | 189961351 | 189630403 | 99.83 |
| ZS13 | 181341357 | 180852178 | 99.73 |
| ZS14 | 186302660 | 185038306 | 99.32 |
| ZS15 | 209787759 | 209308178 | 99.77 |
| ZS16 | 164335614 | 163937212 | 99.76 |
| ZS17 | 172438170 | 172118393 | 99.81 |
| ZS18 | 157114594 | 156866979 | 99.84 |
| ZS19 | 159427999 | 159082982 | 99.78 |
| ZS20 | 156210622 | 155951413 | 99.83 |
| ZK01 | 176531971 | 176209850 | 99.82 |
| ZK02 | 176004264 | 175440008 | 99.68 |
| ZK03 | 158412736 | 158133518 | 99.82 |
| ZK04 | 183304942 | 182962455 | 99.81 |
| ZK05 | 172008880 | 171485498 | 99.70 |
| ZK06 | 199795774 | 199455981 | 99.83 |
| ZK07 | 200938395 | 200461292 | 99.76 |
| ZK08 | 155537846 | 155295738 | 99.84 |
| ZK09 | 170032667 | 169730076 | 99.82 |
| ZK10 | 161397502 | 160892072 | 99.69 |
| ZK11 | 213137826 | 212418735 | 99.66 |
| ZK12 | 210616209 | 210358404 | 99.88 |
| ZK13 | 180707552 | 180532967 | 99.90 |
| ZK14 | 153633512 | 153351720 | 99.82 |
| ZK15 | 157524693 | 157332086 | 99.88 |
| ZK16 | 173798271 | 173387427 | 99.76 |
| ZK17 | 160696470 | 160380815 | 99.80 |
| ZK18 | 178618536 | 178421002 | 99.89 |
| ZK19 | 178787419 | 178142516 | 99.64 |
| ZK20 | 159939585 | 159727141 | 99.87 |
| EL01 | 156626461 | 156317243 | 99.80 |
| EL02 | 199270324 | 198725431 | 99.73 |
| EL03 | 194108876 | 192644392 | 99.25 |
| EL04 | 193375084 | 192827851 | 99.72 |
| EL05 | 209505978 | 209047371 | 99.78 |
| EL06 | 162915572 | 162506072 | 99.75 |
| EL07 | 211536971 | 210845404 | 99.67 |
| EL08 | 191860859 | 191522738 | 99.82 |
| EL09 | 233723555 | 233185077 | 99.77 |
| EL10 | 219699120 | 219438558 | 99.88 |
| EL11 | 220281854 | 219738341 | 99.75 |
| EL12 | 189263891 | 188224606 | 99.45 |
| EL13 | 206379467 | 205805748 | 99.72 |
| EL14 | 184173993 | 183674256 | 99.73 |
| EL15 | 188730252 | 188051071 | 99.64 |
| EL16 | 179342179 | 178757835 | 99.67 |
| EL17 | 196191346 | 195676731 | 99.74 |
| EL18 | 186381635 | 161752439 | 86.79 |
| EL19 | 205797920 | 204934597 | 99.58 |
| EL20 | 194017225 | 193720621 | 99.85 |
| VT01 | 192527540 | 191768588 | 99.61 |
| VT02 | 182360581 | 181760983 | 99.67 |
| VT03 | 176194414 | 175894600 | 99.83 |
| VT04 | 210285918 | 210071223 | 99.90 |
| VT05 | 198003120 | 197741515 | 99.87 |
| VT06 | 201042556 | 200680356 | 99.82 |
| VT07 | 198117488 | 197720840 | 99.80 |
| VT08 | 188113772 | 187599357 | 99.73 |
| VT09 | 199361426 | 199202212 | 99.92 |
| VT10 | 208301188 | 208082121 | 99.89 |
| VT11 | 200605735 | 200280115 | 99.84 |
| VT12 | 189814933 | 189368409 | 99.76 |
| VT13 | 205932633 | 205664603 | 99.87 |
| VT14 | 199647590 | 199167594 | 99.76 |
| VT15 | 186329696 | 186136488 | 99.90 |
| VT16 | 178123930 | 177846282 | 99.84 |
| VT17 | 186586877 | 186251079 | 99.82 |
| VT18 | 204958314 | 204769701 | 99.91 |
| VT19 | 194749140 | 194369588 | 99.81 |
| VT20 | 194755799 | 194441582 | 99.84 |
| Average | 185256411 | 184660768 | 99.68 |

Sample: Sample name. Total reads: Total reads quantity. Mapped reads: Reads number aligned to the reference genome (including single-sided and double-ended alignments). Mapping rate: Alignment rate, which is the percentage of reads on the reference genome that are aligned to the total number of reads

**Table S5** The statistical results of population SNPs in each sample

| **Sample** | **Total sites** | **HET** | **UNKNOWN** | **HOM** | **Ts** | **Tv** | **Ts/Tv** |
| --- | --- | --- | --- | --- | --- | --- | --- |
| PA01 | 27945703 | 4547347 | 1845911 | 1442373 | 13394484 | 5576596 | 2.4 |
| PA02 | 28141989 | 4871742 | 1259410 | 14489553 | 13654964 | 5706331 | 2.39 |
| PA03 | 28327407 | 5221138 | 692023 | 14522126 | 13900422 | 5842842 | 2.38 |
| PA04 | 28238872 | 5091503 | 948897 | 14483422 | 13796237 | 5778688 | 2.39 |
| PA05 | 13352443 | 428703 | 28181247 | 6800301 | 5145543 | 2083461 | 2.47 |
| PA06 | 25160143 | 2509284 | 8710251 | 12383016 | 10582507 | 4309793 | 2.46 |
| PA07 | 19551644 | 1044412 | 18119700 | 10046938 | 7892805 | 3198545 | 2.47 |
| PA08 | 27589806 | 3744130 | 3447596 | 13981162 | 12545081 | 5180211 | 2.42 |
| PA09 | 27038010 | 3211252 | 4773381 | 13740051 | 11977777 | 4973526 | 2.41 |
| PA10 | 27657310 | 4416999 | 2882134 | 13806251 | 12890562 | 5332688 | 2.42 |
| GB01 | 34266516 | 8307860 | 466961 | 5721357 | 9880631 | 4148586 | 2.38 |
| GB02 | 34137453 | 8678687 | 321985 | 5624569 | 10069934 | 4233322 | 2.38 |
| GB03 | 34081309 | 8892800 | 264204 | 5524381 | 10143486 | 4273695 | 2.37 |
| GB04 | 34070005 | 8932130 | 216855 | 5543704 | 10184002 | 4291832 | 2.37 |
| GB05 | 34356338 | 7831367 | 683761 | 5891228 | 9670401 | 4052194 | 2.39 |
| GB06 | 34197362 | 8567601 | 367249 | 5630482 | 9991627 | 4206456 | 2.38 |
| GB07 | 34143731 | 8702499 | 331480 | 5584984 | 10055767 | 4231716 | 2.38 |
| GB08 | 34166492 | 8707339 | 306859 | 5582004 | 10057186 | 4232157 | 2.38 |
| GB09 | 34327269 | 8003037 | 614477 | 5817911 | 9736319 | 4084629 | 2.38 |
| GB10 | 34195842 | 8607283 | 317387 | 5642182 | 10028627 | 4220838 | 2.38 |
| GB11 | 34195607 | 8215201 | 536265 | 5815621 | 9879739 | 4151083 | 2.38 |
| GB12 | 34245439 | 8232357 | 467459 | 5817439 | 9886907 | 4162889 | 2.38 |
| GB13 | 34158644 | 8348374 | 470712 | 5784964 | 9945318 | 4188020 | 2.37 |
| GB14 | 34150400 | 8464521 | 414463 | 5733310 | 9987492 | 4210339 | 2.37 |
| GB15 | 34104525 | 8564780 | 379575 | 5713814 | 10044977 | 4233617 | 2.37 |
| GB16 | 34448650 | 7683558 | 575279 | 6055207 | 9673742 | 4065023 | 2.38 |
| GB17 | 34306181 | 7947063 | 550133 | 5959317 | 9788819 | 4117561 | 2.38 |
| GB18 | 34230069 | 8218264 | 552444 | 5761917 | 9844470 | 4135711 | 2.38 |
| GB19 | 34182035 | 8091284 | 605644 | 5883731 | 9838246 | 4136769 | 2.38 |
| GB20 | 34160809 | 8577751 | 347587 | 5676547 | 10025334 | 4228964 | 2.37 |
| PT01 | 34146254 | 8508815 | 374808 | 5732817 | 10016620 | 4225012 | 2.37 |
| PT02 | 34218518 | 8067432 | 572449 | 5904295 | 9830066 | 4141661 | 2.37 |
| PT03 | 33948667 | 8860547 | 355034 | 5598446 | 10171099 | 4287894 | 2.37 |
| PT06 | 34217353 | 8099787 | 601011 | 5844543 | 9821110 | 4123220 | 2.38 |
| PT07 | 34054487 | 8307789 | 530686 | 5869732 | 9977607 | 4199914 | 2.38 |
| PT08 | 34198840 | 8020181 | 628024 | 5915649 | 9808696 | 4127134 | 2.38 |
| PT09 | 33953531 | 8713831 | 373316 | 5722016 | 10152909 | 4282938 | 2.37 |
| PT10 | 34183832 | 8524384 | 350113 | 5704365 | 10011459 | 4217290 | 2.37 |
| PT11 | 34200228 | 8469086 | 375444 | 5717936 | 9985843 | 4201179 | 2.38 |
| PT12 | 34208792 | 8516533 | 368034 | 5669335 | 9987166 | 4198702 | 2.38 |
| PT04 | 34180857 | 8255748 | 462257 | 5863832 | 9933241 | 4186339 | 2.37 |
| PT13 | 34278654 | 8427616 | 377436 | 5678988 | 9931169 | 4175435 | 2.38 |
| PT14 | 34351595 | 8094924 | 448710 | 5867465 | 9826747 | 4135642 | 2.38 |
| ZS01 | 34388034 | 8319744 | 277412 | 5777504 | 9919964 | 4177284 | 2.37 |
| ZS02 | 34352560 | 8278568 | 281647 | 5849919 | 9939062 | 4189425 | 2.37 |
| ZS03 | 34215714 | 8263050 | 454433 | 5829497 | 9915277 | 4177270 | 2.37 |
| ZS04 | 34355416 | 7903147 | 519385 | 5984746 | 9772325 | 4115568 | 2.37 |
| ZS05 | 34126625 | 8551151 | 315591 | 5769327 | 10070440 | 4250038 | 2.37 |
| ZS06 | 34230249 | 8453346 | 319438 | 5759661 | 10000618 | 4212389 | 2.37 |
| ZS07 | 34427887 | 7838360 | 464771 | 6031676 | 9758171 | 4111865 | 2.37 |
| ZS08 | 34123868 | 8478241 | 362464 | 5798121 | 10040845 | 4235517 | 2.37 |
| ZS09 | 34336555 | 7750999 | 584837 | 6090303 | 9743168 | 4098134 | 2.38 |
| ZS10 | 34091199 | 8624312 | 312274 | 5734909 | 10098745 | 4260476 | 2.37 |
| ZS11 | 34227017 | 8112470 | 500656 | 5922551 | 9878125 | 4156896 | 2.38 |
| ZS12 | 34191851 | 8571648 | 281256 | 5717939 | 10051612 | 4237975 | 2.37 |
| ZS13 | 34404545 | 7975052 | 382584 | 6000513 | 9830988 | 4144577 | 2.37 |
| ZS14 | 34236005 | 8369691 | 344625 | 5812373 | 9975041 | 4207023 | 2.37 |
| ZS15 | 34254709 | 8293885 | 321149 | 5892951 | 9972423 | 4214413 | 2.37 |
| ZS16 | 34259443 | 7946184 | 594384 | 5962683 | 9788680 | 4120187 | 2.38 |
| ZS17 | 34266802 | 7810404 | 623767 | 6061721 | 9764317 | 4107808 | 2.38 |
| ZS18 | 34243272 | 7560245 | 837385 | 6121792 | 9635261 | 4046776 | 2.38 |
| ZS19 | 34327481 | 7697570 | 704613 | 6033030 | 9665672 | 4064928 | 2.38 |
| ZK01 | 34226328 | 8183362 | 489359 | 5863645 | 9890804 | 4156203 | 2.38 |
| ZK02 | 34231698 | 8033141 | 587097 | 5910758 | 9817629 | 4126270 | 2.38 |
| ZK03 | 34320086 | 7912209 | 579747 | 5950652 | 9760267 | 4102594 | 2.38 |
| ZK04 | 34255801 | 8244326 | 428612 | 5833955 | 9907460 | 4170821 | 2.38 |
| ZK05 | 34263804 | 8181356 | 440719 | 5876815 | 9895009 | 4163162 | 2.38 |
| ZK06 | 34156958 | 8358801 | 407352 | 5839583 | 9994264 | 4204120 | 2.38 |
| ZK07 | 34168623 | 8557244 | 298678 | 5738149 | 10054724 | 4240669 | 2.37 |
| ZK08 | 34270347 | 7769711 | 695039 | 6027597 | 9716556 | 4080752 | 2.38 |
| ZK09 | 34149553 | 8181056 | 562538 | 5869547 | 9895158 | 4155445 | 2.38 |
| ZK10 | 34168284 | 7811239 | 787523 | 5995648 | 9731730 | 4075157 | 2.39 |
| ZK11 | 34153701 | 8521829 | 364475 | 5722689 | 10027709 | 4216809 | 2.38 |
| ZK12 | 34221657 | 8569857 | 283841 | 5687339 | 10035051 | 4222145 | 2.38 |
| ZK13 | 34335637 | 8235018 | 404235 | 5787804 | 9874000 | 4148822 | 2.38 |
| ZK14 | 34347928 | 7827671 | 635359 | 5951736 | 9711105 | 4068302 | 2.39 |
| ZK15 | 34329547 | 7925629 | 568914 | 5938604 | 9768990 | 4095243 | 2.39 |
| ZK16 | 34209177 | 8145239 | 545526 | 5862752 | 9867270 | 4140721 | 2.38 |
| ZK17 | 34354980 | 8008418 | 501158 | 5898138 | 9798464 | 4108092 | 2.39 |
| ZK18 | 34232375 | 8398072 | 383461 | 5748786 | 9958691 | 4188167 | 2.38 |
| ZK19 | 34257471 | 8374812 | 352781 | 5777630 | 9962378 | 4190064 | 2.38 |
| ZK20 | 34354989 | 7959502 | 555933 | 5892270 | 9759579 | 4092193 | 2.38 |
| EL01 | 34352338 | 7694186 | 633712 | 6082458 | 9696436 | 4080208 | 2.38 |
| EL02 | 34238444 | 8276038 | 384221 | 5863991 | 9950845 | 4189184 | 2.38 |
| EL03 | 34172340 | 8245026 | 456901 | 5888427 | 9941170 | 4192283 | 2.37 |
| EL04 | 34195136 | 8262630 | 461562 | 5843366 | 9927247 | 4178749 | 2.38 |
| EL05 | 34286920 | 8252468 | 325598 | 5897708 | 9954074 | 4196102 | 2.37 |
| EL06 | 34202703 | 7946945 | 618755 | 5994291 | 9810416 | 4130820 | 2.37 |
| EL07 | 34123942 | 8516689 | 354114 | 5767949 | 10047717 | 4236921 | 2.37 |
| EL08 | 34224192 | 8104746 | 512667 | 5921089 | 9868706 | 4157129 | 2.37 |
| EL09 | 34069049 | 8853201 | 258517 | 5581927 | 10155267 | 4279861 | 2.37 |
| EL10 | 34098745 | 8763095 | 260527 | 5640327 | 10132679 | 4270743 | 2.37 |
| EL11 | 34067628 | 8908993 | 213682 | 5572391 | 10184319 | 4297065 | 2.37 |
| EL12 | 33031723 | 7657080 | 2340666 | 5733225 | 9489802 | 3900503 | 2.43 |
| EL13 | 34072543 | 8848868 | 238550 | 5602733 | 10162684 | 4288917 | 2.37 |
| EL14 | 34127481 | 8654094 | 310212 | 5670907 | 10078618 | 4246383 | 2.37 |
| EL15 | 34176135 | 8221335 | 463210 | 5902014 | 9933716 | 4189633 | 2.37 |
| EL16 | 34147418 | 8601757 | 325720 | 5687799 | 10053965 | 4235591 | 2.37 |
| EL17 | 34095809 | 8662957 | 311692 | 5692236 | 10101612 | 4253581 | 2.37 |
| EL18 | 33359209 | 9225797 | 425266 | 5752422 | 10523982 | 4454237 | 2.36 |
| EL19 | 33973400 | 8977556 | 248688 | 5563050 | 10229061 | 4311545 | 2.37 |
| EL20 | 34181896 | 8645854 | 261601 | 5673343 | 10073722 | 4245475 | 2.37 |
| VT01 | 34041163 | 8592412 | 419577 | 5709542 | 10058142 | 4243812 | 2.37 |
| VT02 | 34153048 | 8591437 | 437710 | 5580499 | 9974773 | 4197163 | 2.38 |
| VT03 | 34163602 | 8324526 | 517266 | 5757300 | 9909849 | 4171977 | 2.38 |
| VT04 | 34163230 | 8639141 | 345416 | 5614907 | 10028576 | 4225472 | 2.37 |
| VT05 | 34312167 | 8387285 | 431888 | 5631354 | 9870205 | 4148434 | 2.38 |
| VT06 | 34390445 | 8078176 | 432921 | 5861152 | 9803539 | 4135789 | 2.37 |
| VT07 | 33914993 | 8947982 | 416797 | 5482922 | 10153923 | 4276981 | 2.37 |
| VT08 | 34170315 | 8517953 | 387974 | 5686452 | 9993630 | 4210775 | 2.37 |
| VT09 | 34173419 | 8807290 | 325328 | 5456657 | 10035570 | 4228377 | 2.37 |
| VT10 | 34234576 | 8507531 | 375114 | 5645473 | 9963109 | 4189895 | 2.38 |
| VT11 | 34068475 | 8665129 | 392820 | 5636270 | 10058621 | 4242778 | 2.37 |
| VT12 | 34107176 | 8502347 | 466099 | 5687072 | 9984303 | 4205116 | 2.37 |
| VT13 | 34167391 | 8716499 | 316982 | 5561822 | 10043607 | 4234714 | 2.37 |
| VT14 | 34098387 | 8507094 | 393950 | 5763263 | 10037972 | 4232385 | 2.37 |
| VT15 | 34248874 | 8686087 | 386604 | 5441129 | 9940978 | 4186238 | 2.37 |
| VT16 | 34157285 | 8234674 | 523287 | 5847448 | 9906787 | 4175335 | 2.37 |
| VT17 | 34771513 | 7304568 | 473273 | 6213340 | 9505422 | 4012486 | 2.37 |
| VT18 | 34176529 | 8902897 | 300915 | 5382353 | 10050939 | 4234311 | 2.37 |
| VT19 | 34152883 | 8374345 | 444207 | 5791259 | 9965906 | 4199698 | 2.37 |
| VT20 | 34118274 | 8772796 | 458113 | 5413511 | 9989676 | 4196631 | 2.38 |
| Average | 33461628 | 7941828 | 995703 | 6363536 | 10072183 | 4233181 | 2.38 |

Sample: Sample name. Total sites: Total number of SNPs. Heter: Number of heterozygous SNPs. Homo: Number of homozygous SNPs. Ts: Number of transition. Tv: Number of transversion. Ts/Tv: Ratio of transitions to transversions

**Table S6** The results of SNPs annotation

| **Type** | **Number** | **Percentage (%)** |
| --- | --- | --- |
| exonic total | 646994 | 1.33 |
| synonymous SNV | 417551 | 0.86 |
| nonsynonymous SNV | 209531 | 0.43 |
| stopgain | 2672 | 0.01 |
| stoploss | 312 | 0.0 |
| unknown | 16928 | 0.03 |
| splicing | 1520 | 0.0 |
| ncRNA total | 8758 | 0.02 |
| ncRNA_exonic | 4341 | 0.01 |
| ncRNA_splicing | 6 | 0.0 |
| ncRNA_exonic;splicing | 0 | 0.0 |
| ncRNA_intronic | 4411 | 0.01 |
| intronic | 17507454 | 35.9 |
| intergenic | 29425564 | 60.34 |
| UTR5 | 166518 | 0.34 |
| UTR3 | 378989 | 0.78 |
| UTR5;UTR3 | 663 | 0.0 |
| upstream | 303614 | 0.62 |
| downstream | 312350 | 0.64 |
| upstream;downstream | 10270 | 0.02 |
| Total | 48762694 | 100.0 |

Type: The type of SNPs. Number: The number of SNPs of this type. Percentage (%): The proportion of this type SNPs of to the total number of SNPs

**Table S7** The statistical results of population InDels in each sample

| **Sample** | **Total sites** | **HET** | **UNKNOWN** | **HOM** | **Insertion** | **Deletion** |
| --- | --- | --- | --- | --- | --- | --- |
| PA01 | 2494569 | 498404 | 422525 | 1720588 | 1031877 | 1187115 |
| PA02 | 2531396 | 542562 | 312029 | 1750099 | 1063313 | 1229348 |
| PA03 | 2572127 | 603286 | 189103 | 1771570 | 1099834 | 1275022 |
| PA04 | 2554577 | 572035 | 254777 | 1754697 | 1079235 | 1247497 |
| PA05 | 1185583 | 52010 | 3151206 | 747287 | 376066 | 423231 |
| PA06 | 2240025 | 255445 | 1240004 | 1400612 | 777483 | 878574 |
| PA07 | 1719974 | 120456 | 2189982 | 1105674 | 578972 | 647158 |
| PA08 | 2479301 | 397214 | 621770 | 1637801 | 950553 | 1084462 |
| PA09 | 2459776 | 357681 | 691132 | 1627497 | 923642 | 1061536 |
| PA10 | 2514192 | 467215 | 510121 | 1644558 | 986551 | 1125222 |
| GB01 | 3181013 | 961943 | 119615 | 873515 | 833379 | 1002079 |
| GB02 | 3166343 | 1015342 | 92284 | 862117 | 851106 | 1026353 |
| GB03 | 3156955 | 1045110 | 80961 | 853060 | 860544 | 1037626 |
| GB04 | 3150603 | 1061510 | 71263 | 852710 | 867041 | 1047179 |
| GB05 | 3197455 | 900152 | 151117 | 887362 | 813147 | 974367 |
| GB06 | 3175146 | 997366 | 100503 | 863071 | 844403 | 1016034 |
| GB07 | 3170138 | 1014892 | 94548 | 856508 | 849302 | 1022098 |
| GB08 | 3172510 | 1019751 | 88606 | 855219 | 850014 | 1024956 |
| GB09 | 3194829 | 920924 | 140477 | 879856 | 819530 | 981250 |
| GB10 | 3173964 | 1009571 | 90862 | 861689 | 848458 | 1022802 |
| GB11 | 3177425 | 948889 | 126786 | 882986 | 830631 | 1001244 |
| GB12 | 3183347 | 955915 | 116612 | 880212 | 833444 | 1002683 |
| GB13 | 3173580 | 970515 | 114882 | 877109 | 837612 | 1010012 |
| GB14 | 3173870 | 983649 | 106974 | 871593 | 841375 | 1013867 |
| GB15 | 3166127 | 998462 | 100735 | 870762 | 847300 | 1021924 |
| GB16 | 3208852 | 886223 | 134108 | 906903 | 815693 | 977433 |
| GB17 | 3189305 | 917572 | 132425 | 896784 | 824330 | 990026 |
| GB18 | 3181145 | 949013 | 131508 | 874420 | 827073 | 996360 |
| GB19 | 3175941 | 929284 | 140201 | 890660 | 826330 | 993614 |
| GB20 | 3169049 | 1004379 | 94057 | 868601 | 849237 | 1023743 |
| PT01 | 3175249 | 987359 | 102446 | 871032 | 843183 | 1015208 |
| PT02 | 3183372 | 925694 | 136359 | 890661 | 825343 | 991012 |
| PT03 | 3156849 | 1020535 | 100104 | 858598 | 851871 | 1027262 |
| PT06 | 3185270 | 921719 | 145514 | 883583 | 820649 | 984653 |
| PT07 | 3177821 | 938991 | 133160 | 886114 | 829489 | 995616 |
| PT08 | 3183802 | 917674 | 143774 | 890836 | 821896 | 986614 |
| PT09 | 3166804 | 995270 | 103448 | 870564 | 845904 | 1019930 |
| PT10 | 3174764 | 994190 | 98599 | 868533 | 845960 | 1016763 |
| PT11 | 3176039 | 985444 | 105172 | 869431 | 843026 | 1011849 |
| PT12 | 3177992 | 988288 | 105167 | 864639 | 842324 | 1010603 |
| PT04 | 3181151 | 949858 | 119328 | 885749 | 833347 | 1002260 |
| PT13 | 3184636 | 980821 | 105596 | 865033 | 839376 | 1006478 |
| PT14 | 3194018 | 939387 | 116984 | 885697 | 829334 | 995750 |
| ZS01 | 3185461 | 982702 | 93327 | 874596 | 844075 | 1013223 |
| ZS02 | 3181584 | 977844 | 91868 | 884790 | 846452 | 1016182 |
| ZS03 | 3175937 | 954120 | 123647 | 882382 | 835353 | 1001149 |
| ZS04 | 3192762 | 912730 | 132230 | 898364 | 823167 | 987927 |
| ZS05 | 3159472 | 1003527 | 96112 | 876975 | 852849 | 1027653 |
| ZS06 | 3172251 | 991458 | 98902 | 873475 | 847380 | 1017553 |
| ZS07 | 3201683 | 908440 | 121560 | 904403 | 823963 | 988880 |
| ZS08 | 3159421 | 987064 | 108439 | 881162 | 847279 | 1020947 |
| ZS09 | 3190064 | 892678 | 141617 | 911727 | 820113 | 984292 |
| ZS10 | 3156738 | 1016374 | 93100 | 869874 | 854751 | 1031497 |
| ZS11 | 3179110 | 934369 | 127546 | 895061 | 830847 | 998583 |
| ZS12 | 3167811 | 1007556 | 89613 | 871106 | 851938 | 1026724 |
| ZS13 | 3194052 | 932914 | 108007 | 901113 | 832918 | 1001109 |
| ZS14 | 3174424 | 982193 | 98261 | 881208 | 844212 | 1019189 |
| ZS15 | 3169509 | 982503 | 93458 | 890616 | 848796 | 1024323 |
| ZS16 | 3187616 | 910362 | 144906 | 893202 | 821165 | 982399 |
| ZS17 | 3184642 | 897143 | 147514 | 906787 | 820536 | 983394 |
| ZS18 | 3186558 | 855559 | 181004 | 912965 | 805391 | 963133 |
| ZS19 | 3194086 | 877915 | 161244 | 902841 | 810360 | 970396 |
| ZK01 | 3176802 | 947348 | 123120 | 888816 | 832756 | 1003408 |
| ZK02 | 3180398 | 927747 | 135744 | 892197 | 825580 | 994364 |
| ZK03 | 3192102 | 914049 | 134958 | 894977 | 821777 | 987249 |
| ZK04 | 3178974 | 957046 | 113975 | 886091 | 835873 | 1007264 |
| ZK05 | 3183024 | 949478 | 113995 | 889589 | 835279 | 1003788 |
| ZK06 | 3169026 | 975045 | 105344 | 886671 | 843714 | 1018002 |
| ZK07 | 3169149 | 1005318 | 87709 | 873910 | 851437 | 1027791 |
| ZK08 | 3185003 | 890849 | 155508 | 904726 | 815536 | 980039 |
| ZK09 | 3170989 | 938902 | 137150 | 889045 | 829440 | 998507 |
| ZK10 | 3175221 | 886268 | 172382 | 902215 | 812943 | 975540 |
| ZK11 | 3168743 | 993086 | 98937 | 875320 | 847643 | 1020763 |
| ZK12 | 3173505 | 1005913 | 86442 | 870226 | 849826 | 1026313 |
| ZK13 | 3189074 | 960391 | 107208 | 879413 | 835589 | 1004215 |
| ZK14 | 3195185 | 893827 | 150768 | 896306 | 814332 | 975801 |
| ZK15 | 3190622 | 908902 | 138343 | 898219 | 822333 | 984788 |
| ZK16 | 3177724 | 933733 | 135222 | 889407 | 829014 | 994126 |
| ZK17 | 3192228 | 921212 | 128324 | 894322 | 825375 | 990159 |
| ZK18 | 3177901 | 978917 | 103869 | 875399 | 841669 | 1012647 |
| ZK19 | 3180150 | 976509 | 100305 | 879122 | 842116 | 1013515 |
| ZK20 | 3192278 | 919639 | 134730 | 889439 | 822503 | 986575 |
| EL01 | 3196025 | 883527 | 148302 | 908232 | 814105 | 977654 |
| EL02 | 3178565 | 966524 | 104788 | 886209 | 839836 | 1012897 |
| EL03 | 3176180 | 957522 | 113089 | 889295 | 838593 | 1008224 |
| EL04 | 3170407 | 959051 | 123244 | 883384 | 837568 | 1004867 |
| EL05 | 3180383 | 971500 | 92733 | 891470 | 845033 | 1017937 |
| EL06 | 3181269 | 907219 | 146970 | 900628 | 821980 | 985867 |
| EL07 | 3164666 | 999436 | 96881 | 875103 | 849719 | 1024820 |
| EL08 | 3178894 | 942036 | 123433 | 891723 | 833096 | 1000663 |
| EL09 | 3153445 | 1047046 | 81260 | 854335 | 861523 | 1039858 |
| EL10 | 3156340 | 1035214 | 84066 | 860466 | 859151 | 1036529 |
| EL11 | 3150019 | 1057729 | 74077 | 854261 | 866383 | 1045607 |
| EL12 | 2938313 | 772760 | 597579 | 827434 | 737381 | 862813 |
| EL13 | 3149815 | 1051007 | 79395 | 855869 | 864675 | 1042201 |
| EL14 | 3161847 | 1013678 | 95724 | 864837 | 852524 | 1025991 |
| EL15 | 3166193 | 951681 | 126255 | 891957 | 837832 | 1005806 |
| EL16 | 3162369 | 1005442 | 101696 | 866579 | 849396 | 1022625 |
| EL17 | 3156465 | 1018938 | 93045 | 867638 | 856030 | 1030546 |
| EL18 | 3171864 | 981353 | 116831 | 866038 | 839823 | 1007568 |
| EL19 | 3145369 | 1055690 | 81578 | 853449 | 865777 | 1043362 |
| EL20 | 3163392 | 1023212 | 87426 | 862056 | 856435 | 1028833 |
| VT01 | 3162720 | 996307 | 108543 | 868516 | 846691 | 1018132 |
| VT02 | 3175598 | 993135 | 112864 | 854489 | 839404 | 1008220 |
| VT03 | 3174440 | 958671 | 128006 | 874969 | 831996 | 1001644 |
| VT04 | 3170334 | 1012512 | 97173 | 856067 | 848804 | 1019775 |
| VT05 | 3189392 | 976391 | 111739 | 858564 | 833404 | 1001551 |
| VT06 | 3196887 | 944564 | 109636 | 884999 | 830925 | 998638 |
| VT07 | 3149594 | 1033213 | 110029 | 843250 | 850548 | 1025915 |
| VT08 | 3174840 | 987739 | 106086 | 867421 | 843096 | 1012064 |
| VT09 | 3175320 | 1026622 | 94703 | 839441 | 847344 | 1018719 |
| VT10 | 3178742 | 985474 | 110748 | 861122 | 838311 | 1008285 |
| VT11 | 3163020 | 1003004 | 106827 | 863235 | 846007 | 1020232 |
| VT12 | 3169811 | 980612 | 118445 | 867218 | 839072 | 1008758 |
| VT13 | 3170950 | 1022384 | 91420 | 851332 | 850117 | 1023599 |
| VT14 | 3163271 | 993202 | 104058 | 875555 | 846936 | 1021821 |
| VT15 | 3188025 | 1006938 | 106330 | 834793 | 836789 | 1004942 |
| VT16 | 3172889 | 949078 | 128978 | 885141 | 833653 | 1000566 |
| VT17 | 3238175 | 859462 | 116147 | 922302 | 810328 | 971436 |
| VT18 | 3174895 | 1040280 | 89013 | 831898 | 850001 | 1022177 |
| VT19 | 3170070 | 974949 | 114288 | 876779 | 840436 | 1011292 |
| VT20 | 3155244 | 1001084 | 150170 | 829588 | 834708 | 995964 |
| Average | 3100444 | 919179 | 187105 | 929358 | 841237 | 1007300 |

Sample: Sample name. Total sites: Total number of InDel. Heter: Number of heterozygous InDels. Hom: Number of Homozygous InDels. Insertion: The type in the InDel is the number of insertions. Deletion: The number of deletions in the InDel

**Table S8** The results of InDels annotation

| **Type** | **Number** | **Percentage (%)** |
| --- | --- | --- |
| exonic total | 8417 | 0.16 |
| frameshift deletion | 2497 | 0.05 |
| frameshift insertion | 1712 | 0.03 |
| nonframeshift deletion | 2144 | 0.04 |
| nonframeshift insertion | 1200 | 0.02 |
| nonframeshift substitution | 0 | 0.00 |
| stopgain | 99 | 0.00 |
| stoploss | 13 | 0.00 |
| unknown | 752 | 0.01 |
| splicing | 412 | 0.01 |
| ncRNA total | 791 | 0.02 |
| ncRNA_exonic | 287 | 0.01 |
| ncRNA_exonic;splicing | 0 | 0.00 |
| ncRNA_splicing | 1 | 0.00 |
| ncRNA_intronic | 503 | 0.01 |
| ncRNA_UTR5 | 0 | 0.00 |
| intronic | 1917016 | 37.32 |
| intergenic | 3067062 | 59.72 |
| UTR5 | 17582 | 0.34 |
| UTR3 | 47165 | 0.92 |
| UTR5;UTR3 | 76 | 0.00 |
| upstream | 37468 | 0.73 |
| downstream | 38594 | 0.75 |
| upstream;downstream | 1503 | 0.03 |
| Total | 5136086 | 100.00 |

Type: The type of InDels. Number: The number of InDels of this type. Percentage (%): The proportion of this type of InDels to the total number

**Table S9** The results of IBS similarity value

| **Pop ID** | **PA** | **GB** | **PT** | **ZS** | **ZK** | **EL** | **VT** |
| --- | --- | --- | --- | --- | --- | --- | --- |
| VT | 0.351 | 0.166 | 0.166 | 0.164 | 0.165 | 0.165 | 0.146 |
| EL | 0.352 | 0.164 | 0.164 | 0.162 | 0.161 | 0.149 |  |
| ZK | 0.351 | 0.164 | 0.163 | 0.162 | 0.162 |  |  |
| ZS | 0.351 | 0.164 | 0.163 | 0.149 |  |  |  |
| PT | 0.351 | 0.165 | 0.151 |  |  |  |  |
| GB | 0.351 | 0.153 |  |  |  |  |  |
| PA | 0.042 |  |  |  |  |  |  |

Pop ID: The name of population. PA: Argali. GB: Guide black fur sheep. PT: Plateau Tibetan sheep. ZS: Zhashijia sheep. ZK: Zeku sheep. EL: Euler Tibetan sheep. VT: Valley Tibetan sheep. Pop ID: The name of the population

**Table S10** The results of genetic diversity in each population

| **Pop ID** | **Obs_Het** | **Exp_Het** | **Pi** | ***F*_IS_** |
| --- | --- | --- | --- | --- |
| PA | 0.054 | 0.044 | 0.046 | -0.007 |
| GB | 0.241 | 0.259 | 0.265 | 0.069 |
| PT | 0.241 | 0.257 | 0.267 | 0.071 |
| ZS | 0.236 | 0.255 | 0.262 | 0.074 |
| ZK | 0.237 | 0.256 | 0.263 | 0.075 |
| EL | 0.245 | 0.255 | 0.262 | 0.074 |
| VT | 0.246 | 0.253 | 0.259 | 0.042 |

Pop ID: The name of the population. PA: Argali. GB: Guide black fur sheep. PT: Plateau Tibetan sheep. ZS: Zhashijia sheep. ZK: Zeku sheep. EL: Euler Tibetan sheep. VT: Valley Tibetan sheep. Obs_Het: Average observed heterozygosity of all loci in the population. Exp_Het: The average expected heterozygosity of in loci of the population was calculated using the Hardy-Weinberg equilibrium. Pi: average nucleotide diversity at all sites of the population (π values). *F*_IS_: The average coefficient of inbreeding in individuals within a population

**Table S11** The statistical results of *F*_ST_ in population

| **Pop ID** | **PA** | **GB** | **PT** | **ZS** | **ZK** | **EL** | **VT** |
| --- | --- | --- | --- | --- | --- | --- | --- |
| VT | 0.340 | 0.031 | 0.031 | 0.031 | 0.031 | 0.032 | 0 |
| EL | 0.336 | 0.024 | 0.023 | 0.024 | 0.022 | 0 |  |
| ZK | 0.334 | 0.022 | 0.022 | 0.022 | 0 |  |  |
| ZS | 0.339 | 0.023 | 0.022 | 0 |  |  |  |
| PT | 0.361 | 0.022 | 0 |  |  |  |  |
| GB | 0.331 | 0 |  |  |  |  |  |
| PA | 0 |  |  |  |  |  |  |

Pop ID: The name of the population. PA: Argali. GB: Guide black fur sheep. PT: Plateau Tibetan sheep. ZS: Zhashijia sheep. ZK: Zeku sheep. EL: Euler Tibetan sheep. VT: Valley Tibetan sheep. *F*_ST_ = 0 ~ 0.05: the differentiation is very small and can be ignored. *F*_ST_ = 0.05 ~ 0.15: moderate genetic differentiation between populations. *F*_ST_ = 0.15 ~ 0.25: genetic differentiation between populations was large. *F*_ST_ > 0.25: there is a great deal of genetic differentiation between populations

**Table S12** The statistical results of unique SNPs in Agrali and Tibetan sheep, separately (partial results)

| **Agrali** | | | | **Tibetan sheep** | | | |
| --- | --- | --- | --- | --- | --- | --- | --- |
| **Chr** | **Chr_posi** | **Ref** | **Alt** | **Chr** | **Chr_posi** | **Ref** | **Alt** |
| NC_056054.1 | 79188 | A | G | NC_056054.1 | 34742 | G | A |
| NC_056054.1 | 79380 | G | A | NC_056054.1 | 61132 | A | G |
| NC_056054.1 | 83466 | C | T | NC_056054.1 | 61692 | C | G |
| NC_056054.1 | 108971 | C | T | NC_056054.1 | 61791 | G | A |
| NC_056054.1 | 108985 | C | T | NC_056054.1 | 63579 | A | G |
| NC_056054.1 | 109313 | G | A | NC_056054.1 | 63625 | T | C |
| NC_056054.1 | 109405 | C | T | NC_056054.1 | 63657 | A | G |
| NC_056054.1 | 121303 | G | A | NC_056054.1 | 63658 | T | C |
| NC_056054.1 | 125644 | C | G | NC_056054.1 | 63668 | T | C |
| NC_056054.1 | 127686 | C | G | NC_056054.1 | 63689 | T | C |
| NC_056054.1 | 130211 | C | T | NC_056054.1 | 63723 | T | C |
| NC_056054.1 | 132386 | G | T | NC_056054.1 | 63935 | T | A |
| NC_056054.1 | 134869 | A | T | NC_056054.1 | 65206 | A | C |
| NC_056054.1 | 134871 | G | A | NC_056054.1 | 65208 | A | G |
| NC_056054.1 | 140013 | A | G | NC_056054.1 | 65214 | A | G |
| NC_056054.1 | 140039 | T | G | NC_056054.1 | 65233 | T | G |
| NC_056054.1 | 140060 | T | C | NC_056054.1 | 65256 | C | G |
| NC_056054.1 | 151644 | A | G | NC_056054.1 | 65265 | A | C |
| NC_056054.1 | 153122 | A | G | NC_056054.1 | 65700 | A | G |
| NC_056054.1 | 153766 | A | G | NC_056054.1 | 66788 | C | T |
| NC_056054.1 | 154136 | G | C | NC_056054.1 | 79380 | G | A |
| NC_056054.1 | 154382 | T | C | NC_056054.1 | 79617 | T | C |
| NC_056054.1 | 162131 | A | G | NC_056054.1 | 80273 | C | T |
| NC_056054.1 | 172138 | T | C | NC_056054.1 | 80909 | C | T |
| NC_056054.1 | 173338 | A | G | NC_056054.1 | 82672 | T | C |
| NC_056054.1 | 173459 | A | C | NC_056054.1 | 94255 | G | A |
| NC_056054.1 | 173479 | A | G | NC_056054.1 | 95467 | T | C |
| NC_056054.1 | 181229 | C | T | NC_056054.1 | 96109 | G | A |
| NC_056054.1 | 187784 | G | A | NC_056054.1 | 96427 | A | G |
| NC_056054.1 | 192379 | A | C | NC_056054.1 | 96973 | A | G |
| NC_056054.1 | 195687 | C | A | NC_056054.1 | 97144 | T | C |
| NC_056054.1 | 196445 | C | G | NC_056054.1 | 97993 | G | T |
| NC_056054.1 | 196465 | T | C | NC_056054.1 | 98303 | G | A |
| NC_056054.1 | 196467 | A | C | NC_056054.1 | 100609 | C | T |
| NC_056054.1 | 196473 | T | C | NC_056054.1 | 100616 | C | T |
| NC_056054.1 | 196480 | A | G | NC_056054.1 | 100618 | G | A |
| NC_056054.1 | 196503 | T | C | NC_056054.1 | 105017 | G | A |
| NC_056054.1 | 196516 | A | G | NC_056054.1 | 108985 | C | T |
| NC_056054.1 | 196520 | A | G | NC_056054.1 | 109061 | G | A |
| NC_056054.1 | 197217 | G | A | NC_056054.1 | 110124 | G | A |
| NC_056054.1 | 201638 | A | G | NC_056054.1 | 110512 | G | A |
| NC_056054.1 | 201798 | C | T | NC_056054.1 | 110920 | C | T |
| NC_056054.1 | 204328 | G | A | NC_056054.1 | 111839 | G | A |
| NC_056054.1 | 204361 | A | G | NC_056054.1 | 112190 | G | A |
| NC_056054.1 | 204638 | T | C | NC_056054.1 | 112503 | T | C |
| NC_056054.1 | 205627 | G | A | NC_056054.1 | 119950 | T | C |
| NC_056054.1 | 205637 | T | C | NC_056054.1 | 119988 | A | G |
| NC_056054.1 | 206674 | G | A | NC_056054.1 | 121303 | G | A |
| NC_056054.1 | 209056 | C | T | NC_056054.1 | 121983 | G | T |

Chr: Chromosome. Chr_posi: The location of the SNP on this chromosome. Ref: Reference allele. Alt: Alternative allele

**Table S13** The annotation results of unique SNPs in Agrali (partial results)

| **Type** | **Gene** | **Chrom** | **Chr_posi** | **REF** | **ALT** |
| --- | --- | --- | --- | --- | --- |
| intergenic | gene-LOC114110836(dist=26318);gene-FAM240C(dist=38729) | NC_056054.1 | 79188 | A | G |
| intergenic | gene-LOC114110836(dist=26510);gene-FAM240C(dist=38537) | NC_056054.1 | 79380 | G | A |
| intergenic | gene-LOC114110836(dist=30596);gene-FAM240C(dist=34451) | NC_056054.1 | 83466 | C | T |
| intergenic | gene-LOC114110836(dist=56101);gene-FAM240C(dist=8946) | NC_056054.1 | 108971 | C | T |
| intergenic | gene-LOC114110836(dist=56115);gene-FAM240C(dist=8932) | NC_056054.1 | 108985 | C | T |
| intergenic | gene-LOC114110836(dist=56443);gene-FAM240C(dist=8604) | NC_056054.1 | 109313 | G | A |
| intergenic | gene-LOC114110836(dist=56535);gene-FAM240C(dist=8512) | NC_056054.1 | 109405 | C | T |
| intronic | gene-FAM240C | NC_056054.1 | 121303 | G | A |
| intronic | gene-FAM240C | NC_056054.1 | 125644 | C | G |
| intronic | gene-FAM240C | NC_056054.1 | 127686 | C | G |
| downstream | gene-FAM240C(dist=739) | NC_056054.1 | 130211 | C | T |
| intergenic | gene-FAM240C(dist=2914);gene-PDCD1(dist=13627) | NC_056054.1 | 132386 | G | T |
| intergenic | gene-FAM240C(dist=5397);gene-PDCD1(dist=11144) | NC_056054.1 | 134869 | A | T |
| intergenic | gene-FAM240C(dist=5399);gene-PDCD1(dist=11142) | NC_056054.1 | 134871 | G | A |
| intergenic | gene-FAM240C(dist=10541);gene-PDCD1(dist=6000) | NC_056054.1 | 140013 | A | G |
| intergenic | gene-FAM240C(dist=10567);gene-PDCD1(dist=5974) | NC_056054.1 | 140039 | T | G |
| intergenic | gene-FAM240C(dist=10588);gene-PDCD1(dist=5953) | NC_056054.1 | 140060 | T | C |
| UTR5 | gene-PDCD1(rna-XM_027964169.2#NC_056054.1#146012:c.-6577A>G;rna-XM_042239178.1#NC_056054.1#146012:c.-9491A>G;rna-XM_042239174.1#NC_056054.1#146012:c.-9491A>G) | NC_056054.1 | 151644 | A | G |
| UTR5 | gene-PDCD1(rna-XM_027964169.2#NC_056054.1#146012:c.-5099A>G;rna-XM_042239178.1#NC_056054.1#146012:c.-8013A>G;rna-XM_042239174.1#NC_056054.1#146012:c.-8013A>G) | NC_056054.1 | 153122 | A | G |
| UTR5 | gene-PDCD1(rna-XM_027964169.2#NC_056054.1#146012:c.-4455A>G;rna-XM_042239178.1#NC_056054.1#146012:c.-7369A>G;rna-XM_042239174.1#NC_056054.1#146012:c.-7369A>G) | NC_056054.1 | 153766 | A | G |
| UTR5 | gene-PDCD1(rna-XM_027964169.2#NC_056054.1#146012:c.-4085G>C;rna-XM_042239178.1#NC_056054.1#146012:c.-6999G>C;rna-XM_042239174.1#NC_056054.1#146012:c.-6999G>C) | NC_056054.1 | 154136 | G | C |
| UTR5 | gene-PDCD1(rna-XM_027964169.2#NC_056054.1#146012:c.-3839T>C;rna-XM_042239178.1#NC_056054.1#146012:c.-6753T>C;rna-XM_042239174.1#NC_056054.1#146012:c.-6753T>C) | NC_056054.1 | 154382 | T | C |
| intronic | gene-PDCD1 | NC_056054.1 | 162131 | A | G |
| intergenic | gene-PDCD1(dist=4600);gene-NEU4(dist=3937) | NC_056054.1 | 172138 | T | C |
| intergenic | gene-PDCD1(dist=5800);gene-NEU4(dist=2737) | NC_056054.1 | 173338 | A | G |
| intergenic | gene-PDCD1(dist=5921);gene-NEU4(dist=2616) | NC_056054.1 | 173459 | A | C |
| intergenic | gene-PDCD1(dist=5941);gene-NEU4(dist=2596) | NC_056054.1 | 173479 | A | G |
| upstream;downstream | gene-NEU4(dist=831);gene-GAL3ST2(dist=146) | NC_056054.1 | 181229 | C | T |
| intronic | gene-GAL3ST2 | NC_056054.1 | 187784 | G | A |
| intronic | gene-GAL3ST2 | NC_056054.1 | 192379 | A | C |
| intronic | gene-GAL3ST2 | NC_056054.1 | 195687 | C | A |
| intronic | gene-GAL3ST2 | NC_056054.1 | 196445 | C | G |
| intronic | gene-GAL3ST2 | NC_056054.1 | 196465 | T | C |
| intronic | gene-GAL3ST2 | NC_056054.1 | 196467 | A | C |
| intronic | gene-GAL3ST2 | NC_056054.1 | 196473 | T | C |
| intronic | gene-GAL3ST2 | NC_056054.1 | 196480 | A | G |
| intronic | gene-GAL3ST2 | NC_056054.1 | 196503 | T | C |
| intronic | gene-GAL3ST2 | NC_056054.1 | 196516 | A | G |
| intronic | gene-GAL3ST2 | NC_056054.1 | 196520 | A | G |
| intronic | gene-GAL3ST2 | NC_056054.1 | 197217 | G | A |
| intronic | gene-GAL3ST2 | NC_056054.1 | 201638 | A | G |
| intronic | gene-GAL3ST2 | NC_056054.1 | 201798 | C | T |
| intergenic | gene-GAL3ST2(dist=1275);gene-D2HGDH(dist=5509) | NC_056054.1 | 204328 | G | A |
| intergenic | gene-GAL3ST2(dist=1308);gene-D2HGDH(dist=5476) | NC_056054.1 | 204361 | A | G |
| intergenic | gene-GAL3ST2(dist=1585);gene-D2HGDH(dist=5199) | NC_056054.1 | 204638 | T | C |
| intergenic | gene-GAL3ST2(dist=2574);gene-D2HGDH(dist=4210) | NC_056054.1 | 205627 | G | A |
| intergenic | gene-GAL3ST2(dist=2584);gene-D2HGDH(dist=4200) | NC_056054.1 | 205637 | T | C |
| intergenic | gene-GAL3ST2(dist=3621);gene-D2HGDH(dist=3163) | NC_056054.1 | 206674 | G | A |
| downstream | gene-D2HGDH(dist=781) | NC_056054.1 | 209056 | C | T |
| intronic | gene-D2HGDH | NC_056054.1 | 216199 | C | T |
| intronic | gene-D2HGDH | NC_056054.1 | 219673 | C | T |
| intronic | gene-D2HGDH | NC_056054.1 | 225466 | G | A |
| intronic | gene-D2HGDH | NC_056054.1 | 229575 | C | T |
| intronic | gene-D2HGDH | NC_056054.1 | 233276 | C | A |
| intronic | gene-D2HGDH | NC_056054.1 | 236350 | T | C |
| upstream;downstream | gene-D2HGDH(dist=486);gene-ING5(dist=516) | NC_056054.1 | 237146 | G | A |
| UTR3 | gene-ING5(rna-XM_027966767.2#NC_056054.1#237661:c.*964C>T;rna-XM_027966759.2#NC_056054.1#237661:c.*964C>T) | NC_056054.1 | 239731 | G | A |
| intergenic | gene-ING5(dist=1326);gene-DTYMK(dist=5773) | NC_056054.1 | 256361 | G | A |
| intergenic | gene-ING5(dist=3260);gene-DTYMK(dist=3839) | NC_056054.1 | 258295 | C | T |
| intergenic | gene-ING5(dist=3281);gene-DTYMK(dist=3818) | NC_056054.1 | 258316 | A | G |
| intergenic | gene-ING5(dist=4285);gene-DTYMK(dist=2814) | NC_056054.1 | 259320 | G | C |
| intergenic | gene-ING5(dist=4320);gene-DTYMK(dist=2779) | NC_056054.1 | 259355 | C | T |
| intergenic | gene-ING5(dist=4340);gene-DTYMK(dist=2759) | NC_056054.1 | 259375 | A | G |
| intergenic | gene-ING5(dist=4760);gene-DTYMK(dist=2339) | NC_056054.1 | 259795 | G | A |
| intergenic | gene-ING5(dist=5681);gene-DTYMK(dist=1418) | NC_056054.1 | 260716 | G | C |
| intronic | gene-DTYMK | NC_056054.1 | 263508 | T | G |
| intronic | gene-DTYMK | NC_056054.1 | 263902 | C | T |
| intronic | gene-DTYMK | NC_056054.1 | 265887 | C | T |
| intronic | gene-DTYMK | NC_056054.1 | 265932 | G | A |
| intronic | gene-DTYMK | NC_056054.1 | 266304 | G | A |
| intronic | gene-DTYMK | NC_056054.1 | 266408 | C | T |
| intronic | gene-DTYMK | NC_056054.1 | 267295 | G | T |
| UTR3 | gene-DTYMK(rna-XM_027966775.2#NC_056054.1#262133:c.*25A>G;rna-XM_004003428.5#NC_056054.1#262133:c.*25A>G) | NC_056054.1 | 269249 | A | G |
| downstream | gene-ATG4B(dist=651) | NC_056054.1 | 270820 | T | C |
| intronic | gene-ATG4B | NC_056054.1 | 274385 | C | T |
| intronic | gene-ATG4B | NC_056054.1 | 274399 | C | T |
| intronic | gene-ATG4B | NC_056054.1 | 275523 | C | T |
| intronic | gene-ATG4B | NC_056054.1 | 275960 | T | C |
| intronic | gene-ATG4B | NC_056054.1 | 276704 | C | A |
| intronic | gene-ATG4B | NC_056054.1 | 277424 | C | G |
| intronic | gene-ATG4B | NC_056054.1 | 283877 | G | A |
| intronic | gene-ATG4B | NC_056054.1 | 284903 | G | C |
| intronic | gene-ATG4B | NC_056054.1 | 286080 | G | A |
| UTR5 | gene-THAP4(rna-XM_027966795.2#NC_056054.1#288972:c.-779A>C) | NC_056054.1 | 289464 | A | C |
| UTR5 | gene-THAP4(rna-XM_027966795.2#NC_056054.1#288972:c.-302C>G) | NC_056054.1 | 289941 | C | G |
| intronic | gene-THAP4 | NC_056054.1 | 291434 | T | C |
| intronic | gene-THAP4 | NC_056054.1 | 291901 | C | G |
| intronic | gene-THAP4 | NC_056054.1 | 294530 | C | T |
| intronic | gene-THAP4 | NC_056054.1 | 297169 | A | C |
| intronic | gene-THAP4 | NC_056054.1 | 298710 | G | A |
| intronic | gene-THAP4 | NC_056054.1 | 299226 | C | T |
| intronic | gene-THAP4 | NC_056054.1 | 301353 | G | A |
| intronic | gene-THAP4 | NC_056054.1 | 304596 | A | G |
| intronic | gene-THAP4 | NC_056054.1 | 305265 | G | A |
| intronic | gene-THAP4 | NC_056054.1 | 305702 | C | T |
| intronic | gene-THAP4 | NC_056054.1 | 306461 | G | A |
| intronic | gene-THAP4 | NC_056054.1 | 308109 | A | G |
| intronic | gene-THAP4 | NC_056054.1 | 308119 | A | G |
| intronic | gene-THAP4 | NC_056054.1 | 308136 | G | A |

Type: The type of SNPs. Gene: The gene to which the SNP is annotated. Chr: Chromosome. Chr_posi: The location of the SNP on this chromosome. Ref: Reference allele. Alt: Alternative allele

**Table S14** The annotation results of unique SNPs in Tibetan sheep (partial results)

| **Type** | **Gene** | **Chrom** | **Chr_posi** | **REF** | **ALT** |
| --- | --- | --- | --- | --- | --- |
| intergenic | NONE(dist=NONE),gene-LOC114112203(dist=12013) | NC_056054.1 | 34742 | G | A |
| intergenic | gene-LOC114110836(dist=8262),gene-FAM240C(dist=56785) | NC_056054.1 | 61132 | A | G |
| intergenic | gene-LOC114110836(dist=8822),gene-FAM240C(dist=56225) | NC_056054.1 | 61692 | C | G |
| intergenic | gene-LOC114110836(dist=8921),gene-FAM240C(dist=56126) | NC_056054.1 | 61791 | G | A |
| intergenic | gene-LOC114110836(dist=10709),gene-FAM240C(dist=54338) | NC_056054.1 | 63579 | A | G |
| intergenic | gene-LOC114110836(dist=10755),gene-FAM240C(dist=54292) | NC_056054.1 | 63625 | T | C |
| intergenic | gene-LOC114110836(dist=10787),gene-FAM240C(dist=54260) | NC_056054.1 | 63657 | A | G |
| intergenic | gene-LOC114110836(dist=10788),gene-FAM240C(dist=54259) | NC_056054.1 | 63658 | T | C |
| intergenic | gene-LOC114110836(dist=10798),gene-FAM240C(dist=54249) | NC_056054.1 | 63668 | T | C |
| intergenic | gene-LOC114110836(dist=10819),gene-FAM240C(dist=54228) | NC_056054.1 | 63689 | T | C |
| intergenic | gene-LOC114110836(dist=10853),gene-FAM240C(dist=54194) | NC_056054.1 | 63723 | T | C |
| intergenic | gene-LOC114110836(dist=11065),gene-FAM240C(dist=53982) | NC_056054.1 | 63935 | T | A |
| intergenic | gene-LOC114110836(dist=12336),gene-FAM240C(dist=52711) | NC_056054.1 | 65206 | A | C |
| intergenic | gene-LOC114110836(dist=12338),gene-FAM240C(dist=52709) | NC_056054.1 | 65208 | A | G |
| intergenic | gene-LOC114110836(dist=12344),gene-FAM240C(dist=52703) | NC_056054.1 | 65214 | A | G |
| intergenic | gene-LOC114110836(dist=12363),gene-FAM240C(dist=52684) | NC_056054.1 | 65233 | T | G |
| intergenic | gene-LOC114110836(dist=12386),gene-FAM240C(dist=52661) | NC_056054.1 | 65256 | C | G |
| intergenic | gene-LOC114110836(dist=12395),gene-FAM240C(dist=52652) | NC_056054.1 | 65265 | A | C |
| intergenic | gene-LOC114110836(dist=12830),gene-FAM240C(dist=52217) | NC_056054.1 | 65700 | A | G |
| intergenic | gene-LOC114110836(dist=13918),gene-FAM240C(dist=51129) | NC_056054.1 | 66788 | C | T |
| intergenic | gene-LOC114110836(dist=26510),gene-FAM240C(dist=38537) | NC_056054.1 | 79380 | G | A |
| intergenic | gene-LOC114110836(dist=26747),gene-FAM240C(dist=38300) | NC_056054.1 | 79617 | T | C |
| intergenic | gene-LOC114110836(dist=27403),gene-FAM240C(dist=37644) | NC_056054.1 | 80273 | C | T |
| intergenic | gene-LOC114110836(dist=28039),gene-FAM240C(dist=37008) | NC_056054.1 | 80909 | C | T |
| intergenic | gene-LOC114110836(dist=29802),gene-FAM240C(dist=35245) | NC_056054.1 | 82672 | T | C |
| intergenic | gene-LOC114110836(dist=41385),gene-FAM240C(dist=23662) | NC_056054.1 | 94255 | G | A |
| intergenic | gene-LOC114110836(dist=42597),gene-FAM240C(dist=22450) | NC_056054.1 | 95467 | T | C |
| intergenic | gene-LOC114110836(dist=43239),gene-FAM240C(dist=21808) | NC_056054.1 | 96109 | G | A |
| intergenic | gene-LOC114110836(dist=43557),gene-FAM240C(dist=21490) | NC_056054.1 | 96427 | A | G |
| intergenic | gene-LOC114110836(dist=44103),gene-FAM240C(dist=20944) | NC_056054.1 | 96973 | A | G |
| intergenic | gene-LOC114110836(dist=44274),gene-FAM240C(dist=20773) | NC_056054.1 | 97144 | T | C |
| intergenic | gene-LOC114110836(dist=45123),gene-FAM240C(dist=19924) | NC_056054.1 | 97993 | G | T |
| intergenic | gene-LOC114110836(dist=45433),gene-FAM240C(dist=19614) | NC_056054.1 | 98303 | G | A |
| intergenic | gene-LOC114110836(dist=47739),gene-FAM240C(dist=17308) | NC_056054.1 | 100609 | C | T |
| intergenic | gene-LOC114110836(dist=47746),gene-FAM240C(dist=17301) | NC_056054.1 | 100616 | C | T |
| intergenic | gene-LOC114110836(dist=47748),gene-FAM240C(dist=17299) | NC_056054.1 | 100618 | G | A |
| intergenic | gene-LOC114110836(dist=52147),gene-FAM240C(dist=12900) | NC_056054.1 | 105017 | G | A |
| intergenic | gene-LOC114110836(dist=56115),gene-FAM240C(dist=8932) | NC_056054.1 | 108985 | C | T |
| intergenic | gene-LOC114110836(dist=56191),gene-FAM240C(dist=8856) | NC_056054.1 | 109061 | G | A |
| intergenic | gene-LOC114110836(dist=57254),gene-FAM240C(dist=7793) | NC_056054.1 | 110124 | G | A |
| intergenic | gene-LOC114110836(dist=57642),gene-FAM240C(dist=7405) | NC_056054.1 | 110512 | G | A |
| intergenic | gene-LOC114110836(dist=58050),gene-FAM240C(dist=6997) | NC_056054.1 | 110920 | C | T |
| intergenic | gene-LOC114110836(dist=58969),gene-FAM240C(dist=6078) | NC_056054.1 | 111839 | G | A |
| intergenic | gene-LOC114110836(dist=59320),gene-FAM240C(dist=5727) | NC_056054.1 | 112190 | G | A |
| intergenic | gene-LOC114110836(dist=59633),gene-FAM240C(dist=5414) | NC_056054.1 | 112503 | T | C |
| intronic | gene-FAM240C | NC_056054.1 | 119950 | T | C |
| intronic | gene-FAM240C | NC_056054.1 | 119988 | A | G |
| intronic | gene-FAM240C | NC_056054.1 | 121303 | G | A |
| intronic | gene-FAM240C | NC_056054.1 | 121983 | G | T |
| intronic | gene-FAM240C | NC_056054.1 | 122558 | C | T |
| intergenic | NONE(dist=NONE),gene-LOC114112203(dist=12013) | NC_056054.1 | 34742 | G | A |
| intergenic | gene-LOC114110836(dist=8262),gene-FAM240C(dist=56785) | NC_056054.1 | 61132 | A | G |
| intergenic | gene-LOC114110836(dist=8822),gene-FAM240C(dist=56225) | NC_056054.1 | 61692 | C | G |
| intergenic | gene-LOC114110836(dist=8921),gene-FAM240C(dist=56126) | NC_056054.1 | 61791 | G | A |
| intergenic | gene-LOC114110836(dist=10709),gene-FAM240C(dist=54338) | NC_056054.1 | 63579 | A | G |
| intergenic | gene-LOC114110836(dist=10755),gene-FAM240C(dist=54292) | NC_056054.1 | 63625 | T | C |
| intergenic | gene-LOC114110836(dist=10787),gene-FAM240C(dist=54260) | NC_056054.1 | 63657 | A | G |
| intergenic | gene-LOC114110836(dist=10788),gene-FAM240C(dist=54259) | NC_056054.1 | 63658 | T | C |
| intergenic | gene-LOC114110836(dist=10798),gene-FAM240C(dist=54249) | NC_056054.1 | 63668 | T | C |
| intergenic | gene-LOC114110836(dist=10819),gene-FAM240C(dist=54228) | NC_056054.1 | 63689 | T | C |
| intergenic | gene-LOC114110836(dist=10853),gene-FAM240C(dist=54194) | NC_056054.1 | 63723 | T | C |
| intergenic | gene-LOC114110836(dist=11065),gene-FAM240C(dist=53982) | NC_056054.1 | 63935 | T | A |
| intergenic | gene-LOC114110836(dist=12336),gene-FAM240C(dist=52711) | NC_056054.1 | 65206 | A | C |
| intergenic | gene-LOC114110836(dist=12338),gene-FAM240C(dist=52709) | NC_056054.1 | 65208 | A | G |
| intergenic | gene-LOC114110836(dist=12344),gene-FAM240C(dist=52703) | NC_056054.1 | 65214 | A | G |
| intergenic | gene-LOC114110836(dist=12363),gene-FAM240C(dist=52684) | NC_056054.1 | 65233 | T | G |
| intergenic | gene-LOC114110836(dist=12386),gene-FAM240C(dist=52661) | NC_056054.1 | 65256 | C | G |
| intergenic | gene-LOC114110836(dist=12395),gene-FAM240C(dist=52652) | NC_056054.1 | 65265 | A | C |
| intergenic | gene-LOC114110836(dist=12830),gene-FAM240C(dist=52217) | NC_056054.1 | 65700 | A | G |
| intergenic | gene-LOC114110836(dist=13918),gene-FAM240C(dist=51129) | NC_056054.1 | 66788 | C | T |
| intergenic | gene-LOC114110836(dist=26510),gene-FAM240C(dist=38537) | NC_056054.1 | 79380 | G | A |
| intergenic | gene-LOC114110836(dist=26747),gene-FAM240C(dist=38300) | NC_056054.1 | 79617 | T | C |
| intergenic | gene-LOC114110836(dist=27403),gene-FAM240C(dist=37644) | NC_056054.1 | 80273 | C | T |
| intergenic | gene-LOC114110836(dist=28039),gene-FAM240C(dist=37008) | NC_056054.1 | 80909 | C | T |
| intergenic | gene-LOC114110836(dist=29802),gene-FAM240C(dist=35245) | NC_056054.1 | 82672 | T | C |
| intergenic | gene-LOC114110836(dist=41385),gene-FAM240C(dist=23662) | NC_056054.1 | 94255 | G | A |
| intergenic | gene-LOC114110836(dist=42597),gene-FAM240C(dist=22450) | NC_056054.1 | 95467 | T | C |
| intergenic | gene-LOC114110836(dist=43239),gene-FAM240C(dist=21808) | NC_056054.1 | 96109 | G | A |
| intergenic | gene-LOC114110836(dist=43557),gene-FAM240C(dist=21490) | NC_056054.1 | 96427 | A | G |
| intergenic | gene-LOC114110836(dist=44103),gene-FAM240C(dist=20944) | NC_056054.1 | 96973 | A | G |
| intergenic | gene-LOC114110836(dist=44274),gene-FAM240C(dist=20773) | NC_056054.1 | 97144 | T | C |
| intergenic | gene-LOC114110836(dist=45123),gene-FAM240C(dist=19924) | NC_056054.1 | 97993 | G | T |
| intergenic | gene-LOC114110836(dist=45433),gene-FAM240C(dist=19614) | NC_056054.1 | 98303 | G | A |
| intergenic | gene-LOC114110836(dist=47739),gene-FAM240C(dist=17308) | NC_056054.1 | 100609 | C | T |
| intergenic | gene-LOC114110836(dist=47746),gene-FAM240C(dist=17301) | NC_056054.1 | 100616 | C | T |
| intergenic | gene-LOC114110836(dist=47748),gene-FAM240C(dist=17299) | NC_056054.1 | 100618 | G | A |
| intergenic | gene-LOC114110836(dist=52147),gene-FAM240C(dist=12900) | NC_056054.1 | 105017 | G | A |
| intergenic | gene-LOC114110836(dist=56115),gene-FAM240C(dist=8932) | NC_056054.1 | 108985 | C | T |
| intergenic | gene-LOC114110836(dist=56191),gene-FAM240C(dist=8856) | NC_056054.1 | 109061 | G | A |
| intergenic | gene-LOC114110836(dist=57254),gene-FAM240C(dist=7793) | NC_056054.1 | 110124 | G | A |
| intergenic | gene-LOC114110836(dist=57642),gene-FAM240C(dist=7405) | NC_056054.1 | 110512 | G | A |
| intergenic | gene-LOC114110836(dist=58050),gene-FAM240C(dist=6997) | NC_056054.1 | 110920 | C | T |
| intergenic | gene-LOC114110836(dist=58969),gene-FAM240C(dist=6078) | NC_056054.1 | 111839 | G | A |
| intergenic | gene-LOC114110836(dist=59320),gene-FAM240C(dist=5727) | NC_056054.1 | 112190 | G | A |
| intergenic | gene-LOC114110836(dist=59633),gene-FAM240C(dist=5414) | NC_056054.1 | 112503 | T | C |
| intronic | gene-FAM240C | NC_056054.1 | 119950 | T | C |
| intronic | gene-FAM240C | NC_056054.1 | 119988 | A | G |
| intronic | gene-FAM240C | NC_056054.1 | 121303 | G | A |
| intronic | gene-FAM240C | NC_056054.1 | 121983 | G | T |

Type: The type of SNPs. Gene: The gene to which the SNP is annotated. Chr: Chromosome. Chr_posi: The location of the SNP on this chromosome. Ref: Reference allele. Alt: Alternative allele

**Table S15** The KEGG enrichment results of unique SNPs in Argali (*P* < 0.05)

| **PathwayID** | **Pathway** | **List_number** | **Total_number** | ***P_*value** | **FDR** |
| --- | --- | --- | --- | --- | --- |
| ko04512 | ECM-receptor interaction | 39 | 90 | 3.74E-07 | 0.000129 |
| ko02010 | ABC transporters | 25 | 56 | 0.000024 | 0.004198 |
| ko04930 | Type II diabetes mellitus | 20 | 47 | 0.000342 | 0.031336 |
| ko04070 | Phosphatidylinositol signaling system | 34 | 97 | 0.000363 | 0.031336 |
| ko00500 | Starch and sucrose metabolism | 15 | 32 | 0.000542 | 0.037399 |
| ko00562 | Inositol phosphate metabolism | 26 | 74 | 0.001640 | 0.091895 |
| ko00051 | Fructose and mannose metabolism | 14 | 32 | 0.001896 | 0.091895 |
| ko04750 | Inflammatory mediator regulation of TRP channels | 34 | 106 | 0.002131 | 0.091895 |
| ko04611 | Platelet activation | 38 | 123 | 0.002576 | 0.098731 |
| ko04713 | Circadian entrainment | 30 | 94 | 0.004090 | 0.140963 |
| ko04510 | Focal adhesion | 56 | 203 | 0.005242 | 0.140963 |
| ko04270 | Vascular smooth muscle contraction | 40 | 136 | 0.005292 | 0.140963 |
| ko04020 | Calcium signaling pathway | 64 | 238 | 0.005643 | 0.140963 |
| ko04912 | GnRH signaling pathway | 29 | 92 | 0.005720 | 0.140963 |
| ko04742 | Taste transduction | 26 | 81 | 0.006623 | 0.143705 |
| ko00524 | Neomycin, kanamycin and gentamicin biosynthesis | 4 | 5 | 0.006665 | 0.143705 |
| ko04928 | Parathyroid hormone synthesis, secretion and action | 32 | 106 | 0.007812 | 0.158530 |
| ko04927 | Cortisol synthesis and secretion | 22 | 67 | 0.008941 | 0.161348 |
| ko00052 | Galactose metabolism | 12 | 30 | 0.009291 | 0.161348 |
| ko04658 | Th1 and Th2 cell differentiation | 28 | 91 | 0.009354 | 0.161348 |
| ko04392 | Hippo signaling pathway - multiple species | 11 | 28 | 0.014586 | 0.217988 |
| ko04915 | Estrogen signaling pathway | 38 | 136 | 0.015393 | 0.217988 |
| ko03460 | Fanconi anemia pathway | 17 | 51 | 0.017253 | 0.217988 |
| ko04360 | Axon guidance | 48 | 180 | 0.017379 | 0.217988 |
| ko04114 | Oocyte meiosis | 33 | 116 | 0.017550 | 0.217988 |
| ko04911 | Insulin secretion | 26 | 87 | 0.017586 | 0.217988 |
| ko04934 | Cushing syndrome | 43 | 159 | 0.018504 | 0.217988 |
| ko04935 | Growth hormone synthesis, secretion and action | 34 | 121 | 0.019359 | 0.217988 |
| ko04925 | Aldosterone synthesis and secretion | 28 | 96 | 0.019622 | 0.217988 |
| ko04914 | Progesterone-mediated oocyte maturation | 26 | 88 | 0.020345 | 0.217988 |
| ko04725 | Cholinergic synapse | 31 | 109 | 0.020934 | 0.217988 |
| ko04918 | Thyroid hormone synthesis | 23 | 76 | 0.021184 | 0.217988 |
| ko04971 | Gastric acid secretion | 23 | 76 | 0.021184 | 0.217988 |
| ko04391 | Hippo signaling pathway - fly | 21 | 68 | 0.021483 | 0.217988 |
| ko04726 | Serotonergic synapse | 32 | 114 | 0.023090 | 0.227386 |
| ko04931 | Insulin resistance | 31 | 110 | 0.023727 | 0.227386 |
| ko04022 | cGMP-PKG signaling pathway | 43 | 165 | 0.033584 | 0.309302 |
| ko04720 | Long-term potentiation | 20 | 67 | 0.034769 | 0.309302 |
| ko04724 | Glutamatergic synapse | 30 | 109 | 0.034965 | 0.309302 |
| ko01522 | Endocrine resistance | 27 | 97 | 0.038356 | 0.330823 |
| ko04921 | Oxytocin signaling pathway | 39 | 150 | 0.042729 | 0.359546 |
| ko04071 | Sphingolipid signaling pathway | 32 | 120 | 0.045375 | 0.369125 |
| ko04211 | Longevity regulating pathway | 25 | 90 | 0.046007 | 0.369125 |
| ko04923 | Regulation of lipolysis in adipocytes | 17 | 57 | 0.049399 | 0.387336 |

**Table S16** The GO enrichment results of unique SNPs in Argali (*P* < 0.05)

| **Category** | **GO.ID** | **Term** | **List** | **Total** | ***P_*value** | **FDR** |
| --- | --- | --- | --- | --- | --- | --- |
| MF | GO:0032559 | adenyl ribonucleotide binding | 349 | 1231 | 4.62E-12 | 4.14E-08 |
| MF | GO:0030554 | adenyl nucleotide binding | 349 | 1235 | 7.32E-12 | 4.14E-08 |
| MF | GO:0005524 | ATP binding | 340 | 1200 | 1.01E-11 | 4.14E-08 |
| MF | GO:0008144 | drug binding | 362 | 1331 | 5.69E-10 | 0.000002 |
| MF | GO:0043168 | anion binding | 524 | 2063 | 3.84E-09 | 0.000009 |
| MF | GO:0097367 | carbohydrate derivative binding | 434 | 1680 | 1.40E-08 | 0.000029 |
| MF | GO:0043167 | ion binding | 952 | 4071 | 4.24E-08 | 0.000075 |
| CC | GO:0005604 | basement membrane | 30 | 59 | 1.99E-07 | 0.000306 |
| CC | GO:0031012 | extracellular matrix | 72 | 201 | 2.68E-07 | 0.000343 |
| CC | GO:0062023 | collagen-containing extracellular matrix | 51 | 127 | 2.79E-07 | 0.000343 |
| CC | GO:0005856 | cytoskeleton | 351 | 1366 | 4.16E-07 | 0.000430 |
| MF | GO:0042623 | ATPase activity, coupled | 80 | 231 | 4.18E-07 | 0.000430 |
| MF | GO:0003774 | motor activity | 39 | 89 | 0.000001 | 0.000580 |
| MF | GO:0008569 | ATP-dependent microtubule motor activity, minus-end-directed | 13 | 17 | 0.000001 | 0.001070 |
| MF | GO:0032553 | ribonucleotide binding | 385 | 1524 | 0.000001 | 0.001199 |
| CC | GO:0044430 | cytoskeletal part | 268 | 1019 | 0.000002 | 0.001254 |
| MF | GO:0036094 | small molecule binding | 479 | 1952 | 0.000002 | 0.001617 |
| MF | GO:0032555 | purine ribonucleotide binding | 381 | 1513 | 0.000002 | 0.001617 |
| MF | GO:0005509 | calcium ion binding | 144 | 494 | 0.000003 | 0.001738 |
| MF | GO:0035639 | purine ribonucleoside triphosphate binding | 371 | 1477 | 0.000004 | 0.002635 |
| MF | GO:0017076 | purine nucleotide binding | 381 | 1523 | 0.000005 | 0.002767 |
| MF | GO:0016787 | hydrolase activity | 481 | 1981 | 0.000008 | 0.004251 |
| MF | GO:0000166 | nucleotide binding | 424 | 1725 | 0.000009 | 0.004385 |
| MF | GO:1901265 | nucleoside phosphate binding | 424 | 1725 | 0.000009 | 0.004385 |
| MF | GO:0005085 | guanyl-nucleotide exchange factor activity | 60 | 173 | 0.000011 | 0.005307 |
| BP | GO:0007155 | cell adhesion | 202 | 740 | 0.000030 | 0.014317 |
| BP | GO:0022610 | biological adhesion | 203 | 746 | 0.000035 | 0.016202 |
| BP | GO:0051128 | regulation of cellular component organization | 368 | 1455 | 0.000039 | 0.017273 |
| MF | GO:0016887 | ATPase activity | 95 | 319 | 0.000055 | 0.023407 |
| MF | GO:0099604 | ligand-gated calcium channel activity | 10 | 14 | 0.000060 | 0.024700 |
| MF | GO:0016301 | kinase activity | 158 | 584 | 0.000079 | 0.031479 |
| CC | GO:0098862 | cluster of actin-based cell projections | 33 | 86 | 0.000099 | 0.036924 |
| CC | GO:0015630 | microtubule cytoskeleton | 204 | 790 | 0.000099 | 0.036924 |
| CC | GO:0120025 | plasma membrane bounded cell projection | 259 | 1034 | 0.000108 | 0.039002 |
| CC | GO:0005829 | cytosol | 506 | 2160 | 0.000116 | 0.040734 |
| MF | GO:0022804 | active transmembrane transporter activity | 73 | 238 | 0.000143 | 0.049107 |
| MF | GO:0016773 | phosphotransferase activity, alcohol group as acceptor | 144 | 533 | 0.000175 | 0.058241 |
| CC | GO:0005581 | collagen trimer | 18 | 38 | 0.000180 | 0.058263 |
| CC | GO:0044420 | extracellular matrix component | 16 | 32 | 0.000186 | 0.058674 |
| CC | GO:0042995 | cell projection | 266 | 1074 | 0.000192 | 0.059198 |
| CC | GO:0005903 | brush border | 22 | 51 | 0.000199 | 0.059842 |
| BP | GO:0033043 | regulation of organelle organization | 201 | 759 | 0.000211 | 0.060434 |
| MF | GO:0042800 | histone methyltransferase activity (H3-K4 specific) | 9 | 13 | 0.000211 | 0.060434 |
| BP | GO:0008589 | regulation of smoothened signaling pathway | 24 | 56 | 0.000217 | 0.060652 |
| MF | GO:0051020 | GTPase binding | 98 | 343 | 0.000234 | 0.064002 |
| MF | GO:0042626 | ATPase activity, coupled to transmembrane movement of substances | 33 | 89 | 0.000246 | 0.065998 |
| CC | GO:0044463 | cell projection part | 176 | 680 | 0.000262 | 0.067184 |
| CC | GO:0120038 | plasma membrane bounded cell projection part | 176 | 680 | 0.000262 | 0.067184 |
| CC | GO:0005858 | axonemal dynein complex | 8 | 11 | 0.000276 | 0.069337 |
| MF | GO:0046872 | metal ion binding | 560 | 2416 | 0.000300 | 0.073879 |
| MF | GO:0015399 | primary active transmembrane transporter activity | 33 | 90 | 0.000313 | 0.074191 |
| MF | GO:0015405 | P-P-bond-hydrolysis-driven transmembrane transporter activity | 33 | 90 | 0.000313 | 0.074191 |
| MF | GO:0019899 | enzyme binding | 321 | 1322 | 0.000332 | 0.077241 |
| CC | GO:0000421 | autophagosome membrane | 11 | 19 | 0.000377 | 0.085496 |
| MF | GO:0015278 | calcium-release channel activity | 7 | 9 | 0.000381 | 0.085496 |
| MF | GO:0043492 | ATPase activity, coupled to movement of substances | 33 | 91 | 0.000395 | 0.087000 |
| BP | GO:0044648 | histone H3-K4 dimethylation | 5 | 5 | 0.000432 | 0.093396 |
| BP | GO:1901620 | regulation of smoothened signaling pathway involved in dorsal/ventral neural tube patterning | 7 | 9 | 0.000464 | 0.098701 |
| CC | GO:0071944 | cell periphery | 675 | 2984 | 0.000482 | 0.100687 |
| MF | GO:0043169 | cation binding | 566 | 2457 | 0.000526 | 0.105968 |
| CC | GO:0044450 | microtubule organizing center part | 56 | 181 | 0.000527 | 0.105968 |
| BP | GO:0021904 | dorsal/ventral neural tube patterning | 11 | 19 | 0.000533 | 0.105968 |
| BP | GO:0030030 | cell projection organization | 218 | 845 | 0.000562 | 0.109104 |
| BP | GO:0120036 | plasma membrane bounded cell projection organization | 214 | 828 | 0.000569 | 0.109104 |
| CC | GO:0016459 | myosin complex | 18 | 41 | 0.000575 | 0.109104 |
| CC | GO:0031253 | cell projection membrane | 45 | 139 | 0.000621 | 0.115916 |
| MF | GO:0005539 | glycosaminoglycan binding | 37 | 108 | 0.000650 | 0.119625 |
| BP | GO:0021532 | neural tube patterning | 15 | 31 | 0.000709 | 0.126862 |
| BP | GO:0060249 | anatomical structure homeostasis | 72 | 239 | 0.000710 | 0.126862 |
| MF | GO:0035091 | phosphatidylinositol binding | 49 | 155 | 0.000803 | 0.141388 |
| BP | GO:0060831 | smoothened signaling pathway involved in dorsal/ventral neural tube patterning | 8 | 12 | 0.000888 | 0.152946 |
| MF | GO:0140097 | catalytic activity, acting on DNA | 46 | 144 | 0.000893 | 0.152946 |
| MF | GO:0016772 | transferase activity, transferring phosphorus-containing groups | 175 | 688 | 0.000961 | 0.162328 |
| MF | GO:0004672 | protein kinase activity | 117 | 437 | 0.000989 | 0.164791 |
| BP | GO:0046605 | regulation of centrosome cycle | 16 | 35 | 0.001036 | 0.168743 |
| CC | GO:0005886 | plasma membrane | 655 | 2910 | 0.001040 | 0.168743 |
| CC | GO:0099568 | cytoplasmic region | 68 | 235 | 0.001126 | 0.179674 |
| MF | GO:0005201 | extracellular matrix structural constituent | 15 | 33 | 0.001151 | 0.179674 |
| CC | GO:0044447 | axoneme part | 11 | 21 | 0.001170 | 0.179674 |
| CC | GO:0005930 | axoneme | 26 | 71 | 0.001182 | 0.179674 |
| CC | GO:0097014 | ciliary plasm | 26 | 71 | 0.001182 | 0.179674 |
| BP | GO:0001894 | tissue homeostasis | 45 | 138 | 0.001195 | 0.179674 |
| MF | GO:0005200 | structural constituent of cytoskeleton | 14 | 30 | 0.001219 | 0.181069 |
| BP | GO:0018027 | peptidyl-lysine dimethylation | 9 | 15 | 0.001238 | 0.181723 |
| BP | GO:0016043 | cellular component organization | 811 | 3530 | 0.001303 | 0.188945 |
| MF | GO:0003777 | microtubule motor activity | 22 | 57 | 0.001391 | 0.197089 |
| MF | GO:1990939 | ATP-dependent microtubule motor activity | 22 | 57 | 0.001391 | 0.197089 |
| MF | GO:0003810 | protein-glutamine gamma-glutamyltransferase activity | 6 | 8 | 0.001455 | 0.200305 |
| MF | GO:0043138 | 3'-5' DNA helicase activity | 6 | 8 | 0.001455 | 0.200305 |
| BP | GO:0090066 | regulation of anatomical structure size | 80 | 277 | 0.001472 | 0.200305 |
| BP | GO:0051493 | regulation of cytoskeleton organization | 94 | 334 | 0.001479 | 0.200305 |
| BP | GO:0007010 | cytoskeleton organization | 209 | 821 | 0.001505 | 0.201620 |
| BP | GO:0044087 | regulation of cellular component biogenesis | 156 | 594 | 0.001559 | 0.206624 |
| BP | GO:1905508 | protein localization to microtubule organizing center | 12 | 24 | 0.001692 | 0.220751 |
| BP | GO:0032634 | interleukin-5 production | 6 | 8 | 0.001719 | 0.220751 |
| BP | GO:0032674 | regulation of interleukin-5 production | 6 | 8 | 0.001719 | 0.220751 |
| CC | GO:0017146 | NMDA selective glutamate receptor complex | 4 | 4 | 0.001748 | 0.222091 |
| CC | GO:0008091 | spectrin | 5 | 6 | 0.001778 | 0.223608 |
| CC | GO:0044441 | ciliary part | 84 | 306 | 0.001825 | 0.227193 |
| CC | GO:0005815 | microtubule organizing center | 128 | 496 | 0.001950 | 0.240324 |
| MF | GO:0005540 | hyaluronic acid binding | 10 | 19 | 0.002000 | 0.244062 |
| MF | GO:0004713 | protein tyrosine kinase activity | 33 | 99 | 0.002084 | 0.251855 |
| BP | GO:0097692 | histone H3-K4 monomethylation | 5 | 6 | 0.002133 | 0.255287 |
| CC | GO:0015629 | actin cytoskeleton | 81 | 295 | 0.002162 | 0.256188 |
| BP | GO:0007098 | centrosome cycle | 30 | 86 | 0.002387 | 0.280231 |
| MF | GO:0004714 | transmembrane receptor protein tyrosine kinase activity | 29 | 85 | 0.002556 | 0.297186 |
| CC | GO:0044304 | main axon | 13 | 29 | 0.002632 | 0.300772 |
| BP | GO:0045124 | regulation of bone resorption | 11 | 22 | 0.002635 | 0.300772 |
| MF | GO:1901981 | phosphatidylinositol phosphate binding | 30 | 89 | 0.002683 | 0.300816 |
| CC | GO:0005776 | autophagosome | 22 | 60 | 0.002688 | 0.300816 |
| CC | GO:0030286 | dynein complex | 16 | 39 | 0.002709 | 0.300816 |
| MF | GO:0042169 | SH2 domain binding | 12 | 26 | 0.003040 | 0.332451 |
| CC | GO:0030864 | cortical actin cytoskeleton | 15 | 36 | 0.003048 | 0.332451 |
| MF | GO:0042910 | xenobiotic transmembrane transporter activity | 9 | 17 | 0.003200 | 0.345999 |
| CC | GO:0016020 | membrane | 1331 | 6202 | 0.003236 | 0.346893 |
| MF | GO:0016849 | phosphorus-oxygen lyase activity | 10 | 20 | 0.003267 | 0.347094 |
| MF | GO:0004715 | non-membrane spanning protein tyrosine kinase activity | 15 | 36 | 0.003305 | 0.348134 |
| MF | GO:0005488 | binding | 1802 | 8485 | 0.003345 | 0.349397 |
| MF | GO:0140096 | catalytic activity, acting on a protein | 372 | 1602 | 0.003377 | 0.349839 |
| BP | GO:0007224 | smoothened signaling pathway | 33 | 99 | 0.003448 | 0.354143 |
| BP | GO:0035556 | intracellular signal transduction | 364 | 1521 | 0.003522 | 0.357194 |
| MF | GO:0016462 | pyrophosphatase activity | 171 | 690 | 0.003593 | 0.357194 |
| MF | GO:0016817 | hydrolase activity, acting on acid anhydrides | 171 | 690 | 0.003593 | 0.357194 |
| MF | GO:0016818 | hydrolase activity, acting on acid anhydrides, in phosphorus-containing anhydrides | 171 | 690 | 0.003593 | 0.357194 |
| MF | GO:0005543 | phospholipid binding | 71 | 256 | 0.003645 | 0.359457 |
| BP | GO:0045005 | DNA-dependent DNA replication maintenance of fidelity | 13 | 29 | 0.003744 | 0.363021 |
| BP | GO:0031023 | microtubule organizing center organization | 31 | 92 | 0.003746 | 0.363021 |
| BP | GO:0006928 | movement of cell or subcellular component | 293 | 1206 | 0.003776 | 0.363021 |
| BP | GO:0065007 | biological regulation | 1501 | 6803 | 0.003799 | 0.363021 |
| MF | GO:0004386 | helicase activity | 39 | 126 | 0.003937 | 0.371866 |
| BP | GO:0010824 | regulation of centrosome duplication | 12 | 26 | 0.003952 | 0.371866 |
| BP | GO:0043087 | regulation of GTPase activity | 74 | 262 | 0.004049 | 0.375622 |
| BP | GO:0007154 | cell communication | 740 | 3236 | 0.004053 | 0.375622 |
| BP | GO:0071539 | protein localization to centrosome | 11 | 23 | 0.004091 | 0.376334 |
| BP | GO:0071840 | cellular component organization or biogenesis | 824 | 3627 | 0.004458 | 0.399012 |
| MF | GO:0017111 | nucleoside-triphosphatase activity | 161 | 649 | 0.004462 | 0.399012 |
| BP | GO:0042310 | vasoconstriction | 15 | 36 | 0.004467 | 0.399012 |
| BP | GO:0097756 | negative regulation of blood vessel diameter | 15 | 36 | 0.004467 | 0.399012 |
| CC | GO:0005654 | nucleoplasm | 558 | 2494 | 0.004622 | 0.407349 |
| MF | GO:0003779 | actin binding | 74 | 271 | 0.004647 | 0.407349 |
| MF | GO:0016500 | protein-hormone receptor activity | 7 | 12 | 0.004660 | 0.407349 |
| CC | GO:0031526 | brush border membrane | 10 | 21 | 0.004797 | 0.416418 |
| BP | GO:0030155 | regulation of cell adhesion | 111 | 418 | 0.004863 | 0.419208 |
| BP | GO:0031589 | cell-substrate adhesion | 60 | 207 | 0.004945 | 0.423309 |
| BP | GO:0031333 | negative regulation of protein complex assembly | 30 | 90 | 0.005141 | 0.436430 |
| CC | GO:0043194 | axon initial segment | 5 | 7 | 0.005169 | 0.436430 |
| BP | GO:0046777 | protein autophosphorylation | 43 | 140 | 0.005304 | 0.444771 |
| MF | GO:0031994 | insulin-like growth factor I binding | 5 | 7 | 0.005364 | 0.446699 |
| BP | GO:0043547 | positive regulation of GTPase activity | 63 | 220 | 0.005454 | 0.450264 |
| BP | GO:0009173 | pyrimidine ribonucleoside monophosphate metabolic process | 7 | 12 | 0.005563 | 0.450264 |
| BP | GO:0046049 | UMP metabolic process | 7 | 12 | 0.005563 | 0.450264 |
| BP | GO:0051130 | positive regulation of cellular component organization | 179 | 712 | 0.005594 | 0.450264 |
| BP | GO:0048870 | cell motility | 234 | 953 | 0.005626 | 0.450264 |
| BP | GO:0051674 | localization of cell | 234 | 953 | 0.005626 | 0.450264 |
| CC | GO:0005929 | cilium | 104 | 405 | 0.005741 | 0.454868 |
| BP | GO:0046850 | regulation of bone remodeling | 12 | 27 | 0.005757 | 0.454868 |
| MF | GO:0019199 | transmembrane receptor protein kinase activity | 32 | 101 | 0.005798 | 0.455225 |
| BP | GO:0032501 | multicellular organismal process | 915 | 4059 | 0.005978 | 0.465397 |
| MF | GO:0016788 | hydrolase activity, acting on ester bonds | 136 | 543 | 0.006074 | 0.465397 |
| BP | GO:0051716 | cellular response to stimulus | 863 | 3818 | 0.006088 | 0.465397 |
| BP | GO:0032536 | regulation of cell projection size | 5 | 7 | 0.006154 | 0.465397 |
| BP | GO:0033080 | immature T cell proliferation in thymus | 5 | 7 | 0.006154 | 0.465397 |
| BP | GO:1900247 | regulation of cytoplasmic translational elongation | 5 | 7 | 0.006154 | 0.465397 |
| MF | GO:0005262 | calcium channel activity | 23 | 67 | 0.006235 | 0.466154 |
| BP | GO:0009129 | pyrimidine nucleoside monophosphate metabolic process | 8 | 15 | 0.006240 | 0.466154 |
| MF | GO:1902936 | phosphatidylinositol bisphosphate binding | 20 | 56 | 0.006351 | 0.471548 |
| BP | GO:0023052 | signaling | 729 | 3200 | 0.006474 | 0.477866 |
| CC | GO:0031981 | nuclear lumen | 617 | 2785 | 0.006675 | 0.489720 |
| BP | GO:0120035 | regulation of plasma membrane bounded cell projection organization | 92 | 342 | 0.006737 | 0.491395 |
| MF | GO:0003824 | catalytic activity | 964 | 4419 | 0.006830 | 0.492755 |
| BP | GO:0019318 | hexose metabolic process | 41 | 134 | 0.006850 | 0.492755 |
| BP | GO:0033044 | regulation of chromosome organization | 61 | 214 | 0.006876 | 0.492755 |
| CC | GO:0005614 | interstitial matrix | 6 | 10 | 0.007135 | 0.508357 |
| BP | GO:0110053 | regulation of actin filament organization | 50 | 170 | 0.007181 | 0.508683 |
| MF | GO:0070679 | inositol 1,4,5 trisphosphate binding | 6 | 10 | 0.007437 | 0.513298 |
| MF | GO:0004340 | glucokinase activity | 4 | 5 | 0.007537 | 0.513298 |
| MF | GO:0004396 | hexokinase activity | 4 | 5 | 0.007537 | 0.513298 |
| MF | GO:0004668 | protein-arginine deiminase activity | 4 | 5 | 0.007537 | 0.513298 |
| MF | GO:0008865 | fructokinase activity | 4 | 5 | 0.007537 | 0.513298 |
| MF | GO:0015431 | glutathione S-conjugate-exporting ATPase activity | 4 | 5 | 0.007537 | 0.513298 |
| MF | GO:0071997 | glutathione S-conjugate-transporting ATPase activity | 4 | 5 | 0.007537 | 0.513298 |
| BP | GO:0051276 | chromosome organization | 155 | 613 | 0.007688 | 0.520687 |
| BP | GO:0031344 | regulation of cell projection organization | 92 | 344 | 0.007969 | 0.531293 |
| MF | GO:0043178 | alcohol binding | 15 | 39 | 0.008004 | 0.531293 |
| BP | GO:0007193 | adenylate cyclase-inhibiting G protein-coupled receptor signaling pathway | 15 | 38 | 0.008082 | 0.531293 |
| MF | GO:0009975 | cyclase activity | 9 | 19 | 0.008155 | 0.531293 |
| BP | GO:0015914 | phospholipid transport | 12 | 28 | 0.008157 | 0.531293 |
| BP | GO:0021955 | central nervous system neuron axonogenesis | 12 | 28 | 0.008157 | 0.531293 |
| MF | GO:0004435 | phosphatidylinositol phospholipase C activity | 8 | 16 | 0.008446 | 0.531293 |
| MF | GO:0004629 | phospholipase C activity | 8 | 16 | 0.008446 | 0.531293 |
| BP | GO:0001820 | serotonin secretion | 4 | 5 | 0.008448 | 0.531293 |
| BP | GO:0007195 | adenylate cyclase-inhibiting dopamine receptor signaling pathway | 4 | 5 | 0.008448 | 0.531293 |
| BP | GO:0018101 | protein citrullination | 4 | 5 | 0.008448 | 0.531293 |
| BP | GO:0019372 | lipoxygenase pathway | 4 | 5 | 0.008448 | 0.531293 |
| BP | GO:0021631 | optic nerve morphogenesis | 4 | 5 | 0.008448 | 0.531293 |
| BP | GO:0071716 | leukotriene transport | 4 | 5 | 0.008448 | 0.531293 |
| BP | GO:0009130 | pyrimidine nucleoside monophosphate biosynthetic process | 6 | 10 | 0.008678 | 0.531955 |
| BP | GO:0045780 | positive regulation of bone resorption | 6 | 10 | 0.008678 | 0.531955 |
| BP | GO:0046852 | positive regulation of bone remodeling | 6 | 10 | 0.008678 | 0.531955 |
| MF | GO:0015085 | calcium ion transmembrane transporter activity | 27 | 84 | 0.008701 | 0.531955 |
| MF | GO:0005220 | inositol 1,4,5-trisphosphate-sensitive calcium-release channel activity | 3 | 3 | 0.008761 | 0.531955 |
| MF | GO:0008525 | phosphatidylcholine transporter activity | 3 | 3 | 0.008761 | 0.531955 |
| MF | GO:0019531 | oxalate transmembrane transporter activity | 3 | 3 | 0.008761 | 0.531955 |
| BP | GO:0120031 | plasma membrane bounded cell projection assembly | 86 | 320 | 0.008815 | 0.532601 |
| BP | GO:0034968 | histone lysine methylation | 20 | 56 | 0.008940 | 0.537145 |
| BP | GO:0044089 | positive regulation of cellular component biogenesis | 89 | 333 | 0.009113 | 0.537145 |
| MF | GO:0018024 | histone-lysine N-methyltransferase activity | 14 | 36 | 0.009186 | 0.537145 |
| BP | GO:0048588 | developmental cell growth | 33 | 105 | 0.009341 | 0.537145 |
| BP | GO:0051336 | regulation of hydrolase activity | 142 | 560 | 0.009453 | 0.537145 |
| BP | GO:0009187 | cyclic nucleotide metabolic process | 10 | 22 | 0.009502 | 0.537145 |
| BP | GO:0060271 | cilium assembly | 60 | 213 | 0.009536 | 0.537145 |
| MF | GO:0008201 | heparin binding | 24 | 73 | 0.009583 | 0.537145 |
| BP | GO:0003335 | corneocyte development | 3 | 3 | 0.009587 | 0.537145 |
| BP | GO:0006702 | androgen biosynthetic process | 3 | 3 | 0.009587 | 0.537145 |
| BP | GO:0032466 | negative regulation of cytokinesis | 3 | 3 | 0.009587 | 0.537145 |
| BP | GO:0060763 | mammary duct terminal end bud growth | 3 | 3 | 0.009587 | 0.537145 |
| BP | GO:0061073 | ciliary body morphogenesis | 3 | 3 | 0.009587 | 0.537145 |
| BP | GO:1903971 | positive regulation of response to macrophage colony-stimulating factor | 3 | 3 | 0.009587 | 0.537145 |
| BP | GO:1903974 | positive regulation of cellular response to macrophage colony-stimulating factor stimulus | 3 | 3 | 0.009587 | 0.537145 |
| BP | GO:0007156 | homophilic cell adhesion via plasma membrane adhesion molecules | 24 | 71 | 0.009587 | 0.537145 |
| BP | GO:0048731 | system development | 624 | 2730 | 0.009798 | 0.540150 |
| BP | GO:0006259 | DNA metabolic process | 155 | 617 | 0.009816 | 0.540150 |
| BP | GO:0009190 | cyclic nucleotide biosynthetic process | 7 | 13 | 0.009860 | 0.540150 |
| BP | GO:0060074 | synapse maturation | 7 | 13 | 0.009860 | 0.540150 |
| BP | GO:0080182 | histone H3-K4 trimethylation | 7 | 13 | 0.009860 | 0.540150 |
| BP | GO:0000902 | cell morphogenesis | 145 | 574 | 0.010069 | 0.548153 |
| BP | GO:0015844 | monoamine transport | 13 | 32 | 0.010131 | 0.548153 |
| BP | GO:0045061 | thymic T cell selection | 8 | 16 | 0.010181 | 0.548153 |
| BP | GO:0033077 | T cell differentiation in thymus | 19 | 53 | 0.010216 | 0.548153 |
| MF | GO:0022857 | transmembrane transporter activity | 200 | 839 | 0.010287 | 0.548153 |
| BP | GO:0035296 | regulation of tube diameter | 22 | 64 | 0.010362 | 0.548153 |
| BP | GO:0050880 | regulation of blood vessel size | 22 | 64 | 0.010362 | 0.548153 |
| BP | GO:0097746 | regulation of blood vessel diameter | 22 | 64 | 0.010362 | 0.548153 |
| BP | GO:0051298 | centrosome duplication | 17 | 46 | 0.010567 | 0.556631 |
| BP | GO:0032535 | regulation of cellular component size | 59 | 210 | 0.010682 | 0.560279 |
| CC | GO:0034451 | centriolar satellite | 26 | 82 | 0.010810 | 0.561853 |
| BP | GO:0006796 | phosphate-containing compound metabolic process | 373 | 1589 | 0.010915 | 0.561853 |
| BP | GO:0050770 | regulation of axonogenesis | 27 | 83 | 0.010935 | 0.561853 |
| BP | GO:0007266 | Rho protein signal transduction | 23 | 68 | 0.010985 | 0.561853 |
| BP | GO:0016571 | histone methylation | 23 | 68 | 0.010985 | 0.561853 |
| CC | GO:0042383 | sarcolemma | 20 | 59 | 0.011020 | 0.561853 |
| CC | GO:0098590 | plasma membrane region | 143 | 588 | 0.011054 | 0.561853 |
| BP | GO:0050794 | regulation of cellular process | 1285 | 5816 | 0.011077 | 0.561853 |
| BP | GO:0030031 | cell projection assembly | 86 | 323 | 0.011318 | 0.571723 |
| CC | GO:0033270 | paranode region of axon | 5 | 8 | 0.011467 | 0.571939 |
| BP | GO:0007015 | actin filament organization | 69 | 252 | 0.011476 | 0.571939 |
| MF | GO:0002039 | p53 binding | 16 | 44 | 0.011506 | 0.571939 |
| CC | GO:0030315 | T-tubule | 9 | 20 | 0.011530 | 0.571939 |
| BP | GO:0060284 | regulation of cell development | 129 | 507 | 0.011592 | 0.571939 |
| BP | GO:0051495 | positive regulation of cytoskeleton organization | 41 | 138 | 0.011686 | 0.571939 |
| BP | GO:0032956 | regulation of actin cytoskeleton organization | 60 | 215 | 0.011702 | 0.571939 |
| BP | GO:0005996 | monosaccharide metabolic process | 43 | 146 | 0.011719 | 0.571939 |
| MF | GO:0008092 | cytoskeletal protein binding | 148 | 607 | 0.011739 | 0.571939 |
| MF | GO:0008559 | xenobiotic transmembrane transporting ATPase activity | 5 | 8 | 0.011878 | 0.576425 |
| BP | GO:0006793 | phosphorus metabolic process | 376 | 1605 | 0.011968 | 0.578506 |
| BP | GO:0003018 | vascular process in circulatory system | 25 | 76 | 0.012062 | 0.580748 |
| BP | GO:0010564 | regulation of cell cycle process | 91 | 345 | 0.012134 | 0.581972 |
| CC | GO:0044437 | vacuolar part | 44 | 156 | 0.012409 | 0.590403 |
| BP | GO:0044782 | cilium organization | 63 | 228 | 0.012453 | 0.590403 |
| BP | GO:0016310 | phosphorylation | 256 | 1067 | 0.012454 | 0.590403 |
| BP | GO:0035150 | regulation of tube size | 22 | 65 | 0.012593 | 0.594718 |
| BP | GO:0006974 | cellular response to DNA damage stimulus | 131 | 517 | 0.012642 | 0.594770 |
| CC | GO:0016324 | apical plasma membrane | 47 | 169 | 0.013071 | 0.600620 |
| BP | GO:0031668 | cellular response to extracellular stimulus | 38 | 127 | 0.013157 | 0.600620 |
| BP | GO:0010975 | regulation of neuron projection development | 64 | 233 | 0.013519 | 0.600620 |
| MF | GO:0004993 | G protein-coupled serotonin receptor activity | 6 | 11 | 0.013528 | 0.600620 |
| MF | GO:0030169 | low-density lipoprotein particle binding | 6 | 11 | 0.013528 | 0.600620 |
| MF | GO:0099589 | serotonin receptor activity | 6 | 11 | 0.013528 | 0.600620 |
| BP | GO:0042130 | negative regulation of T cell proliferation | 13 | 33 | 0.013529 | 0.600620 |
| BP | GO:0002182 | cytoplasmic translational elongation | 5 | 8 | 0.013546 | 0.600620 |
| BP | GO:0006007 | glucose catabolic process | 5 | 8 | 0.013546 | 0.600620 |
| BP | GO:0006222 | UMP biosynthetic process | 5 | 8 | 0.013546 | 0.600620 |
| BP | GO:0009174 | pyrimidine ribonucleoside monophosphate biosynthetic process | 5 | 8 | 0.013546 | 0.600620 |
| BP | GO:0033079 | immature T cell proliferation | 5 | 8 | 0.013546 | 0.600620 |
| BP | GO:0034204 | lipid translocation | 5 | 8 | 0.013546 | 0.600620 |
| BP | GO:0043383 | negative T cell selection | 5 | 8 | 0.013546 | 0.600620 |
| BP | GO:0045332 | phospholipid translocation | 5 | 8 | 0.013546 | 0.600620 |
| BP | GO:0046512 | sphingosine biosynthetic process | 5 | 8 | 0.013546 | 0.600620 |
| BP | GO:0031297 | replication fork processing | 10 | 23 | 0.013665 | 0.602980 |
| BP | GO:0006801 | superoxide metabolic process | 15 | 40 | 0.013705 | 0.602980 |
| BP | GO:0031175 | neuron projection development | 130 | 514 | 0.013746 | 0.602980 |
| BP | GO:0050896 | response to stimulus | 1049 | 4715 | 0.013853 | 0.605509 |
| BP | GO:0009653 | anatomical structure morphogenesis | 379 | 1623 | 0.014090 | 0.613700 |
| CC | GO:0005875 | microtubule associated complex | 22 | 68 | 0.014215 | 0.616969 |
| BP | GO:0006325 | chromatin organization | 94 | 360 | 0.014419 | 0.622593 |
| BP | GO:0018022 | peptidyl-lysine methylation | 21 | 62 | 0.014446 | 0.622593 |
| CC | GO:0032838 | plasma membrane bounded cell projection cytoplasm | 32 | 108 | 0.014758 | 0.628812 |
| BP | GO:2000279 | negative regulation of DNA biosynthetic process | 9 | 20 | 0.014791 | 0.628812 |
| BP | GO:0006479 | protein methylation | 26 | 81 | 0.014804 | 0.628812 |
| BP | GO:0008213 | protein alkylation | 26 | 81 | 0.014804 | 0.628812 |
| BP | GO:0032970 | regulation of actin filament-based process | 64 | 234 | 0.014845 | 0.628812 |
| BP | GO:0033554 | cellular response to stress | 262 | 1098 | 0.014978 | 0.632240 |
| BP | GO:0018023 | peptidyl-lysine trimethylation | 12 | 30 | 0.015227 | 0.637941 |
| BP | GO:0030042 | actin filament depolymerization | 16 | 44 | 0.015268 | 0.637941 |
| BP | GO:0072527 | pyrimidine-containing compound metabolic process | 16 | 44 | 0.015268 | 0.637941 |
| MF | GO:0005546 | phosphatidylinositol-4,5-bisphosphate binding | 14 | 38 | 0.015513 | 0.640848 |
| MF | GO:0004252 | serine-type endopeptidase activity | 38 | 132 | 0.015541 | 0.640848 |
| BP | GO:0045995 | regulation of embryonic development | 28 | 89 | 0.015562 | 0.640848 |
| BP | GO:0006837 | serotonin transport | 6 | 11 | 0.015688 | 0.640848 |
| BP | GO:0019320 | hexose catabolic process | 6 | 11 | 0.015688 | 0.640848 |
| BP | GO:2001135 | regulation of endocytic recycling | 6 | 11 | 0.015688 | 0.640848 |
| BP | GO:0046337 | phosphatidylethanolamine metabolic process | 8 | 17 | 0.015701 | 0.640848 |
| MF | GO:0070615 | nucleosome-dependent ATPase activity | 12 | 31 | 0.016016 | 0.644118 |
| CC | GO:0005813 | centrosome | 101 | 406 | 0.016056 | 0.644118 |
| BP | GO:0006670 | sphingosine metabolic process | 7 | 14 | 0.016147 | 0.644118 |
| BP | GO:0007413 | axonal fasciculation | 7 | 14 | 0.016147 | 0.644118 |
| BP | GO:0034312 | diol biosynthetic process | 7 | 14 | 0.016147 | 0.644118 |
| BP | GO:0090330 | regulation of platelet aggregation | 7 | 14 | 0.016147 | 0.644118 |
| BP | GO:0106030 | neuron projection fasciculation | 7 | 14 | 0.016147 | 0.644118 |
| BP | GO:1902903 | regulation of supramolecular fiber organization | 64 | 235 | 0.016278 | 0.647222 |
| BP | GO:0030336 | negative regulation of cell migration | 45 | 157 | 0.016595 | 0.657699 |
| BP | GO:0048468 | cell development | 298 | 1262 | 0.016679 | 0.658913 |
| BP | GO:0007275 | multicellular organism development | 674 | 2979 | 0.017035 | 0.670343 |
| BP | GO:0098751 | bone cell development | 11 | 27 | 0.017077 | 0.670343 |
| BP | GO:0010638 | positive regulation of organelle organization | 96 | 371 | 0.017260 | 0.675404 |
| BP | GO:0015696 | ammonium transport | 15 | 41 | 0.017465 | 0.681237 |
| BP | GO:0006996 | organelle organization | 501 | 2185 | 0.017610 | 0.684295 |
| BP | GO:0001775 | cell activation | 141 | 566 | 0.017660 | 0.684295 |
| CC | GO:0030863 | cortical cytoskeleton | 17 | 50 | 0.017710 | 0.684295 |
| BP | GO:0007017 | microtubule-based process | 129 | 514 | 0.017949 | 0.691378 |
| BP | GO:0043409 | negative regulation of MAPK cascade | 28 | 90 | 0.018130 | 0.696155 |
| BP | GO:0050789 | regulation of biological process | 1395 | 6356 | 0.018266 | 0.699210 |
| CC | GO:0014731 | spectrin-associated cytoskeleton | 4 | 6 | 0.018376 | 0.701236 |
| BP | GO:0007162 | negative regulation of cell adhesion | 43 | 150 | 0.018817 | 0.715845 |
| BP | GO:0045879 | negative regulation of smoothened signaling pathway | 10 | 24 | 0.019053 | 0.718184 |
| BP | GO:0060603 | mammary gland duct morphogenesis | 10 | 24 | 0.019053 | 0.718184 |
| BP | GO:0070849 | response to epidermal growth factor | 10 | 24 | 0.019053 | 0.718184 |
| BP | GO:0060560 | developmental growth involved in morphogenesis | 38 | 130 | 0.019224 | 0.719027 |
| BP | GO:0051056 | regulation of small GTPase mediated signal transduction | 37 | 126 | 0.019250 | 0.719027 |
| BP | GO:1902905 | positive regulation of supramolecular fiber organization | 37 | 126 | 0.019250 | 0.719027 |
| BP | GO:0048856 | anatomical structure development | 744 | 3308 | 0.019683 | 0.724392 |
| BP | GO:0051962 | positive regulation of nervous system development | 68 | 254 | 0.019869 | 0.724392 |
| CC | GO:0030054 | cell junction | 113 | 463 | 0.019926 | 0.724392 |
| BP | GO:0010811 | positive regulation of cell-substrate adhesion | 25 | 79 | 0.019934 | 0.724392 |
| BP | GO:0007584 | response to nutrient | 14 | 38 | 0.019981 | 0.724392 |
| BP | GO:0045453 | bone resorption | 14 | 38 | 0.019981 | 0.724392 |
| MF | GO:0005215 | transporter activity | 207 | 885 | 0.020051 | 0.724392 |
| BP | GO:0007165 | signal transduction | 659 | 2916 | 0.020252 | 0.724392 |
| CC | GO:0005938 | cell cortex | 36 | 127 | 0.020485 | 0.724392 |
| BP | GO:0006468 | protein phosphorylation | 216 | 900 | 0.020564 | 0.724392 |
| BP | GO:1901293 | nucleoside phosphate biosynthetic process | 54 | 196 | 0.020651 | 0.724392 |
| BP | GO:0046649 | lymphocyte activation | 107 | 421 | 0.020770 | 0.724392 |
| MF | GO:0005272 | sodium channel activity | 12 | 32 | 0.020928 | 0.724392 |
| MF | GO:0030246 | carbohydrate binding | 41 | 147 | 0.020975 | 0.724392 |
| BP | GO:0043254 | regulation of protein complex assembly | 74 | 280 | 0.021046 | 0.724392 |
| BP | GO:0002604 | regulation of dendritic cell antigen processing and presentation | 4 | 6 | 0.021078 | 0.724392 |
| BP | GO:0017182 | peptidyl-diphthamide metabolic process | 4 | 6 | 0.021078 | 0.724392 |
| BP | GO:0017183 | peptidyl-diphthamide biosynthetic process from peptidyl-histidine | 4 | 6 | 0.021078 | 0.724392 |
| BP | GO:0021910 | smoothened signaling pathway involved in ventral spinal cord patterning | 4 | 6 | 0.021078 | 0.724392 |
| BP | GO:0030388 | fructose 1,6-bisphosphate metabolic process | 4 | 6 | 0.021078 | 0.724392 |
| BP | GO:0030948 | negative regulation of vascular endothelial growth factor receptor signaling pathway | 4 | 6 | 0.021078 | 0.724392 |
| BP | GO:0033084 | regulation of immature T cell proliferation in thymus | 4 | 6 | 0.021078 | 0.724392 |
| BP | GO:0033085 | negative regulation of T cell differentiation in thymus | 4 | 6 | 0.021078 | 0.724392 |
| BP | GO:0040016 | embryonic cleavage | 4 | 6 | 0.021078 | 0.724392 |
| BP | GO:0045945 | positive regulation of transcription by RNA polymerase III | 4 | 6 | 0.021078 | 0.724392 |
| BP | GO:0051782 | negative regulation of cell division | 4 | 6 | 0.021078 | 0.724392 |
| BP | GO:0031670 | cellular response to nutrient | 9 | 21 | 0.021098 | 0.724392 |
| BP | GO:0034311 | diol metabolic process | 9 | 21 | 0.021098 | 0.724392 |
| BP | GO:0046834 | lipid phosphorylation | 9 | 21 | 0.021098 | 0.724392 |
| BP | GO:0071897 | DNA biosynthetic process | 29 | 95 | 0.021266 | 0.726631 |
| BP | GO:0040011 | locomotion | 257 | 1084 | 0.021281 | 0.726631 |
| BP | GO:0046578 | regulation of Ras protein signal transduction | 31 | 103 | 0.021610 | 0.735812 |
| BP | GO:0042490 | mechanoreceptor differentiation | 18 | 53 | 0.021904 | 0.739481 |
| BP | GO:0048660 | regulation of smooth muscle cell proliferation | 18 | 53 | 0.021904 | 0.739481 |
| CC | GO:0045177 | apical part of cell | 57 | 217 | 0.022197 | 0.739481 |
| MF | GO:0004016 | adenylate cyclase activity | 5 | 9 | 0.022225 | 0.739481 |
| MF | GO:0005337 | nucleoside transmembrane transporter activity | 5 | 9 | 0.022225 | 0.739481 |
| MF | GO:0030228 | lipoprotein particle receptor activity | 5 | 9 | 0.022225 | 0.739481 |
| CC | GO:0044459 | plasma membrane part | 287 | 1266 | 0.022500 | 0.739481 |
| BP | GO:0007528 | neuromuscular junction development | 11 | 28 | 0.022848 | 0.739481 |
| BP | GO:0010676 | positive regulation of cellular carbohydrate metabolic process | 11 | 28 | 0.022848 | 0.739481 |
| BP | GO:0060122 | inner ear receptor cell stereocilium organization | 11 | 28 | 0.022848 | 0.739481 |
| BP | GO:0032233 | positive regulation of actin filament bundle assembly | 13 | 35 | 0.022863 | 0.739481 |
| BP | GO:0009165 | nucleotide biosynthetic process | 53 | 193 | 0.023054 | 0.739481 |
| BP | GO:0045616 | regulation of keratinocyte differentiation | 8 | 18 | 0.023096 | 0.739481 |
| CC | GO:0005774 | vacuolar membrane | 40 | 145 | 0.023625 | 0.739481 |
| BP | GO:0090305 | nucleic acid phosphodiester bond hydrolysis | 16 | 46 | 0.023700 | 0.739481 |
| BP | GO:0048589 | developmental growth | 97 | 380 | 0.023906 | 0.739481 |
| BP | GO:0007041 | lysosomal transport | 20 | 61 | 0.024015 | 0.739481 |
| BP | GO:0042866 | pyruvate biosynthetic process | 20 | 61 | 0.024015 | 0.739481 |
| BP | GO:0007265 | Ras protein signal transduction | 50 | 181 | 0.024024 | 0.739481 |
| MF | GO:0003678 | DNA helicase activity | 18 | 55 | 0.024055 | 0.739481 |
| BP | GO:0051052 | regulation of DNA metabolic process | 71 | 269 | 0.024102 | 0.739481 |
| BP | GO:0048015 | phosphatidylinositol-mediated signaling | 29 | 96 | 0.024457 | 0.739481 |
| BP | GO:0008154 | actin polymerization or depolymerization | 37 | 128 | 0.024511 | 0.739481 |
| BP | GO:0034105 | positive regulation of tissue remodeling | 7 | 15 | 0.024816 | 0.739481 |
| BP | GO:0048240 | sperm capacitation | 7 | 15 | 0.024816 | 0.739481 |
| BP | GO:0071526 | semaphorin-plexin signaling pathway | 7 | 15 | 0.024816 | 0.739481 |
| BP | GO:1902992 | negative regulation of amyloid precursor protein catabolic process | 7 | 15 | 0.024816 | 0.739481 |
| BP | GO:0061564 | axon development | 69 | 261 | 0.025016 | 0.739481 |
| BP | GO:0006171 | cAMP biosynthetic process | 5 | 9 | 0.025193 | 0.739481 |
| BP | GO:0006734 | NADH metabolic process | 5 | 9 | 0.025193 | 0.739481 |
| BP | GO:0036005 | response to macrophage colony-stimulating factor | 5 | 9 | 0.025193 | 0.739481 |
| BP | GO:0036006 | cellular response to macrophage colony-stimulating factor stimulus | 5 | 9 | 0.025193 | 0.739481 |
| BP | GO:0046520 | sphingoid biosynthetic process | 5 | 9 | 0.025193 | 0.739481 |
| BP | GO:0061430 | bone trabecula morphogenesis | 5 | 9 | 0.025193 | 0.739481 |
| BP | GO:2000345 | regulation of hepatocyte proliferation | 5 | 9 | 0.025193 | 0.739481 |
| BP | GO:0060113 | inner ear receptor cell differentiation | 17 | 50 | 0.025209 | 0.739481 |
| BP | GO:0016570 | histone modification | 59 | 219 | 0.025237 | 0.739481 |
| MF | GO:0019838 | growth factor binding | 27 | 91 | 0.025617 | 0.739481 |
| BP | GO:0006448 | regulation of translational elongation | 6 | 12 | 0.025814 | 0.739481 |
| BP | GO:0006590 | thyroid hormone generation | 6 | 12 | 0.025814 | 0.739481 |
| BP | GO:0032196 | transposition | 6 | 12 | 0.025814 | 0.739481 |
| BP | GO:0090407 | organophosphate biosynthetic process | 83 | 321 | 0.025830 | 0.739481 |
| BP | GO:0045880 | positive regulation of smoothened signaling pathway | 10 | 25 | 0.025846 | 0.739481 |
| BP | GO:0051568 | histone H3-K4 methylation | 10 | 25 | 0.025846 | 0.739481 |
| BP | GO:0051345 | positive regulation of hydrolase activity | 96 | 377 | 0.026087 | 0.739481 |
| BP | GO:0016477 | cell migration | 204 | 852 | 0.026222 | 0.739481 |
| BP | GO:0032502 | developmental process | 783 | 3501 | 0.026854 | 0.739481 |
| BP | GO:0019319 | hexose biosynthetic process | 15 | 43 | 0.027282 | 0.739481 |
| BP | GO:0032945 | negative regulation of mononuclear cell proliferation | 15 | 43 | 0.027282 | 0.739481 |
| BP | GO:2000146 | negative regulation of cell motility | 46 | 166 | 0.027960 | 0.739481 |
| BP | GO:0032989 | cellular component morphogenesis | 155 | 636 | 0.027981 | 0.739481 |
| BP | GO:0031669 | cellular response to nutrient levels | 32 | 109 | 0.028103 | 0.739481 |
| MF | GO:0097110 | scaffold protein binding | 10 | 26 | 0.028214 | 0.739481 |
| BP | GO:0050790 | regulation of catalytic activity | 232 | 979 | 0.028388 | 0.739481 |
| BP | GO:0051960 | regulation of nervous system development | 123 | 496 | 0.028922 | 0.739481 |
| CC | GO:0043256 | laminin complex | 3 | 4 | 0.028964 | 0.739481 |
| CC | GO:0044214 | spanning component of plasma membrane | 3 | 4 | 0.028964 | 0.739481 |
| CC | GO:0089717 | spanning component of membrane | 3 | 4 | 0.028964 | 0.739481 |
| CC | GO:0098966 | perisynaptic extracellular matrix | 3 | 4 | 0.028964 | 0.739481 |
| CC | GO:0099535 | synapse-associated extracellular matrix | 3 | 4 | 0.028964 | 0.739481 |
| BP | GO:0006213 | pyrimidine nucleoside metabolic process | 9 | 22 | 0.029126 | 0.739481 |
| BP | GO:0009218 | pyrimidine ribonucleotide metabolic process | 9 | 22 | 0.029126 | 0.739481 |
| BP | GO:0021952 | central nervous system projection neuron axonogenesis | 9 | 22 | 0.029126 | 0.739481 |
| BP | GO:0036297 | interstrand cross-link repair | 9 | 22 | 0.029126 | 0.739481 |
| BP | GO:0046131 | pyrimidine ribonucleoside metabolic process | 9 | 22 | 0.029126 | 0.739481 |
| BP | GO:0097035 | regulation of membrane lipid distribution | 9 | 22 | 0.029126 | 0.739481 |
| CC | GO:0043232 | intracellular non-membrane-bounded organelle | 609 | 2800 | 0.029427 | 0.739481 |
| MF | GO:0001727 | lipid kinase activity | 3 | 4 | 0.029629 | 0.739481 |
| MF | GO:0004052 | arachidonate 12-lipoxygenase activity | 3 | 4 | 0.029629 | 0.739481 |
| MF | GO:0042608 | T cell receptor binding | 3 | 4 | 0.029629 | 0.739481 |
| CC | GO:0043235 | receptor complex | 53 | 203 | 0.029681 | 0.739481 |
| BP | GO:0050865 | regulation of cell activation | 83 | 323 | 0.029771 | 0.739481 |
| BP | GO:0000184 | nuclear-transcribed mRNA catabolic process, nonsense-mediated decay | 11 | 29 | 0.029923 | 0.739481 |
| MF | GO:0005216 | ion channel activity | 79 | 315 | 0.029992 | 0.739481 |
| CC | GO:0043228 | non-membrane-bounded organelle | 609 | 2801 | 0.030170 | 0.739481 |
| BP | GO:0006338 | chromatin remodeling | 23 | 74 | 0.030573 | 0.739481 |
| BP | GO:0045321 | leukocyte activation | 125 | 506 | 0.031013 | 0.739481 |
| MF | GO:0010857 | calcium-dependent protein kinase activity | 7 | 16 | 0.031093 | 0.739481 |
| CC | GO:0016235 | aggresome | 9 | 23 | 0.031189 | 0.739481 |
| BP | GO:1990138 | neuron projection extension | 25 | 82 | 0.031348 | 0.739481 |
| MF | GO:0001784 | phosphotyrosine residue binding | 11 | 30 | 0.031415 | 0.739481 |
| BP | GO:0030837 | negative regulation of actin filament polymerization | 14 | 40 | 0.031439 | 0.739481 |
| BP | GO:0034103 | regulation of tissue remodeling | 14 | 40 | 0.031439 | 0.739481 |
| BP | GO:0042110 | T cell activation | 73 | 281 | 0.031560 | 0.739481 |
| BP | GO:0008064 | regulation of actin polymerization or depolymerization | 33 | 114 | 0.031666 | 0.739481 |
| BP | GO:0030832 | regulation of actin filament length | 33 | 114 | 0.031666 | 0.739481 |
| BP | GO:0071496 | cellular response to external stimulus | 44 | 159 | 0.031725 | 0.739481 |
| BP | GO:0048659 | smooth muscle cell proliferation | 18 | 55 | 0.031808 | 0.739481 |
| BP | GO:0048017 | inositol lipid-mediated signaling | 29 | 98 | 0.031944 | 0.739481 |
| BP | GO:0043405 | regulation of MAP kinase activity | 43 | 155 | 0.032144 | 0.739481 |
| BP | GO:0001973 | adenosine receptor signaling pathway | 3 | 4 | 0.032242 | 0.739481 |
| BP | GO:0002606 | positive regulation of dendritic cell antigen processing and presentation | 3 | 4 | 0.032242 | 0.739481 |
| BP | GO:0006556 | S-adenosylmethionine biosynthetic process | 3 | 4 | 0.032242 | 0.739481 |
| BP | GO:0019249 | lactate biosynthetic process | 3 | 4 | 0.032242 | 0.739481 |
| BP | GO:0021540 | corpus callosum morphogenesis | 3 | 4 | 0.032242 | 0.739481 |
| BP | GO:0030321 | transepithelial chloride transport | 3 | 4 | 0.032242 | 0.739481 |
| BP | GO:0032241 | positive regulation of nucleobase-containing compound transport | 3 | 4 | 0.032242 | 0.739481 |
| BP | GO:0032532 | regulation of microvillus length | 3 | 4 | 0.032242 | 0.739481 |
| BP | GO:0032754 | positive regulation of interleukin-5 production | 3 | 4 | 0.032242 | 0.739481 |
| BP | GO:0032792 | negative regulation of CREB transcription factor activity | 3 | 4 | 0.032242 | 0.739481 |
| BP | GO:0033087 | negative regulation of immature T cell proliferation | 3 | 4 | 0.032242 | 0.739481 |
| BP | GO:0033088 | negative regulation of immature T cell proliferation in thymus | 3 | 4 | 0.032242 | 0.739481 |
| BP | GO:0035583 | sequestering of TGFbeta in extracellular matrix | 3 | 4 | 0.032242 | 0.739481 |
| BP | GO:0035588 | G protein-coupled purinergic receptor signaling pathway | 3 | 4 | 0.032242 | 0.739481 |
| BP | GO:0044806 | G-quadruplex DNA unwinding | 3 | 4 | 0.032242 | 0.739481 |
| BP | GO:0046487 | glyoxylate metabolic process | 3 | 4 | 0.032242 | 0.739481 |
| BP | GO:0046833 | positive regulation of RNA export from nucleus | 3 | 4 | 0.032242 | 0.739481 |
| BP | GO:0061050 | regulation of cell growth involved in cardiac muscle cell development | 3 | 4 | 0.032242 | 0.739481 |
| BP | GO:0061517 | macrophage proliferation | 3 | 4 | 0.032242 | 0.739481 |
| BP | GO:0070428 | regulation of nucleotide-binding oligomerization domain containing 1 signaling pathway | 3 | 4 | 0.032242 | 0.739481 |
| BP | GO:0071205 | protein localization to juxtaparanode region of axon | 3 | 4 | 0.032242 | 0.739481 |
| BP | GO:0072103 | glomerulus vasculature morphogenesis | 3 | 4 | 0.032242 | 0.739481 |
| BP | GO:0072104 | glomerular capillary formation | 3 | 4 | 0.032242 | 0.739481 |
| BP | GO:0072429 | response to intra-S DNA damage checkpoint signaling | 3 | 4 | 0.032242 | 0.739481 |
| BP | GO:0090215 | regulation of 1-phosphatidylinositol-4-phosphate 5-kinase activity | 3 | 4 | 0.032242 | 0.739481 |
| BP | GO:1901030 | positive regulation of mitochondrial outer membrane permeabilization involved in apoptotic signaling pathway | 3 | 4 | 0.032242 | 0.739481 |
| BP | GO:1904781 | positive regulation of protein localization to centrosome | 3 | 4 | 0.032242 | 0.739481 |
| BP | GO:1904970 | brush border assembly | 3 | 4 | 0.032242 | 0.739481 |
| BP | GO:2001205 | negative regulation of osteoclast development | 3 | 4 | 0.032242 | 0.739481 |
| BP | GO:0022604 | regulation of cell morphogenesis | 64 | 243 | 0.032263 | 0.739481 |
| BP | GO:0090322 | regulation of superoxide metabolic process | 8 | 19 | 0.032628 | 0.739481 |
| BP | GO:0009116 | nucleoside metabolic process | 19 | 59 | 0.032870 | 0.739481 |
| MF | GO:0042578 | phosphoric ester hydrolase activity | 68 | 268 | 0.033091 | 0.739481 |
| CC | GO:0032589 | neuron projection membrane | 6 | 13 | 0.033229 | 0.739481 |
| BP | GO:0048661 | positive regulation of smooth muscle cell proliferation | 12 | 33 | 0.033375 | 0.739481 |
| BP | GO:0072698 | protein localization to microtubule cytoskeleton | 12 | 33 | 0.033375 | 0.739481 |
| MF | GO:0042803 | protein homodimerization activity | 102 | 419 | 0.033496 | 0.739481 |
| BP | GO:0070664 | negative regulation of leukocyte proliferation | 15 | 44 | 0.033496 | 0.739481 |
| BP | GO:0030182 | neuron differentiation | 174 | 724 | 0.033631 | 0.739481 |
| MF | GO:0008013 | beta-catenin binding | 18 | 57 | 0.034237 | 0.739481 |
| BP | GO:0120039 | plasma membrane bounded cell projection morphogenesis | 86 | 338 | 0.034336 | 0.739481 |
| BP | GO:0050767 | regulation of neurogenesis | 106 | 425 | 0.034859 | 0.739481 |
| BP | GO:0045664 | regulation of neuron differentiation | 82 | 321 | 0.034896 | 0.739481 |
| BP | GO:0001578 | microtubule bundle formation | 22 | 71 | 0.035048 | 0.739481 |
| BP | GO:0007272 | ensheathment of neurons | 22 | 71 | 0.035048 | 0.739481 |
| BP | GO:0008366 | axon ensheathment | 22 | 71 | 0.035048 | 0.739481 |
| CC | GO:0043005 | neuron projection | 117 | 491 | 0.035124 | 0.739481 |
| BP | GO:0048666 | neuron development | 144 | 592 | 0.035197 | 0.739481 |
| CC | GO:0044428 | nuclear part | 659 | 3047 | 0.035279 | 0.739481 |
| CC | GO:0005814 | centriole | 26 | 90 | 0.035314 | 0.739481 |
| BP | GO:0007507 | heart development | 81 | 317 | 0.035631 | 0.739481 |
| BP | GO:2000026 | regulation of multicellular organismal development | 278 | 1192 | 0.035667 | 0.739481 |
| CC | GO:0005790 | smooth endoplasmic reticulum | 5 | 10 | 0.035841 | 0.739481 |
| CC | GO:0034992 | microtubule organizing center attachment site | 4 | 7 | 0.035984 | 0.739481 |
| CC | GO:0034993 | meiotic nuclear membrane microtubule tethering complex | 4 | 7 | 0.035984 | 0.739481 |
| CC | GO:0106083 | nuclear membrane protein complex | 4 | 7 | 0.035984 | 0.739481 |
| CC | GO:0106094 | nuclear membrane microtubule tethering complex | 4 | 7 | 0.035984 | 0.739481 |
| BP | GO:0007257 | activation of JUN kinase activity | 7 | 16 | 0.036199 | 0.739481 |
| BP | GO:0030224 | monocyte differentiation | 7 | 16 | 0.036199 | 0.739481 |
| BP | GO:0033198 | response to ATP | 7 | 16 | 0.036199 | 0.739481 |
| BP | GO:0046058 | cAMP metabolic process | 7 | 16 | 0.036199 | 0.739481 |
| BP | GO:1903010 | regulation of bone development | 7 | 16 | 0.036199 | 0.739481 |
| BP | GO:1903131 | mononuclear cell differentiation | 7 | 16 | 0.036199 | 0.739481 |
| BP | GO:0007088 | regulation of mitotic nuclear division | 26 | 87 | 0.036241 | 0.739481 |
| BP | GO:0030010 | establishment of cell polarity | 27 | 91 | 0.036322 | 0.739481 |
| BP | GO:0042733 | embryonic digit morphogenesis | 17 | 52 | 0.036664 | 0.739481 |
| CC | GO:0016234 | inclusion body | 16 | 50 | 0.036930 | 0.739481 |
| MF | GO:0015926 | glucosidase activity | 4 | 7 | 0.036989 | 0.739481 |
| MF | GO:0047555 | 3',5'-cyclic-GMP phosphodiesterase activity | 4 | 7 | 0.036989 | 0.739481 |
| MF | GO:0008271 | secondary active sulfate transmembrane transporter activity | 5 | 10 | 0.037013 | 0.739481 |
| MF | GO:0015116 | sulfate transmembrane transporter activity | 5 | 10 | 0.037013 | 0.739481 |
| MF | GO:0015291 | secondary active transmembrane transporter activity | 39 | 144 | 0.037096 | 0.739481 |
| BP | GO:0051239 | regulation of multicellular organismal process | 426 | 1867 | 0.037583 | 0.739481 |
| MF | GO:0030215 | semaphorin receptor binding | 8 | 20 | 0.037872 | 0.739481 |
| BP | GO:0051240 | positive regulation of multicellular organismal process | 245 | 1045 | 0.038228 | 0.739481 |
| BP | GO:0032715 | negative regulation of interleukin-6 production | 11 | 30 | 0.038431 | 0.739481 |
| MF | GO:0016278 | lysine N-methyltransferase activity | 15 | 46 | 0.038629 | 0.739481 |
| MF | GO:0016279 | protein-lysine N-methyltransferase activity | 15 | 46 | 0.038629 | 0.739481 |
| MF | GO:0042054 | histone methyltransferase activity | 15 | 46 | 0.038629 | 0.739481 |
| MF | GO:0048029 | monosaccharide binding | 15 | 46 | 0.038629 | 0.739481 |
| CC | GO:0098858 | actin-based cell projection | 33 | 120 | 0.038676 | 0.739481 |
| BP | GO:0006094 | gluconeogenesis | 14 | 41 | 0.038685 | 0.739481 |
| BP | GO:0030834 | regulation of actin filament depolymerization | 14 | 41 | 0.038685 | 0.739481 |
| BP | GO:0045740 | positive regulation of DNA replication | 9 | 23 | 0.039066 | 0.739481 |
| BP | GO:0048858 | cell projection morphogenesis | 86 | 340 | 0.039156 | 0.739481 |
| BP | GO:0046339 | diacylglycerol metabolic process | 6 | 13 | 0.039489 | 0.739481 |
| BP | GO:0046365 | monosaccharide catabolic process | 6 | 13 | 0.039489 | 0.739481 |
| BP | GO:0050858 | negative regulation of antigen receptor-mediated signaling pathway | 6 | 13 | 0.039489 | 0.739481 |
| BP | GO:0072574 | hepatocyte proliferation | 6 | 13 | 0.039489 | 0.739481 |
| BP | GO:0072575 | epithelial cell proliferation involved in liver morphogenesis | 6 | 13 | 0.039489 | 0.739481 |
| BP | GO:1902430 | negative regulation of amyloid-beta formation | 6 | 13 | 0.039489 | 0.739481 |
| BP | GO:0006281 | DNA repair | 82 | 323 | 0.039915 | 0.739481 |
| CC | GO:0005905 | clathrin-coated pit | 12 | 35 | 0.039998 | 0.739481 |
| BP | GO:0097435 | supramolecular fiber organization | 105 | 423 | 0.040025 | 0.739481 |
| MF | GO:1901363 | heterocyclic compound binding | 813 | 3767 | 0.040325 | 0.739481 |
| MF | GO:0000287 | magnesium ion binding | 43 | 162 | 0.040630 | 0.739481 |
| BP | GO:0010595 | positive regulation of endothelial cell migration | 22 | 72 | 0.040662 | 0.739481 |
| BP | GO:0050769 | positive regulation of neurogenesis | 56 | 212 | 0.040739 | 0.739481 |
| BP | GO:0060348 | bone development | 40 | 145 | 0.040923 | 0.739481 |
| BP | GO:0001865 | NK T cell differentiation | 4 | 7 | 0.040977 | 0.739481 |
| BP | GO:0002468 | dendritic cell antigen processing and presentation | 4 | 7 | 0.040977 | 0.739481 |
| BP | GO:0030643 | cellular phosphate ion homeostasis | 4 | 7 | 0.040977 | 0.739481 |
| BP | GO:0031643 | positive regulation of myelination | 4 | 7 | 0.040977 | 0.739481 |
| BP | GO:0033083 | regulation of immature T cell proliferation | 4 | 7 | 0.040977 | 0.739481 |
| BP | GO:0033182 | regulation of histone ubiquitination | 4 | 7 | 0.040977 | 0.739481 |
| BP | GO:0033683 | nucleotide-excision repair, DNA incision | 4 | 7 | 0.040977 | 0.739481 |
| BP | GO:0033700 | phospholipid efflux | 4 | 7 | 0.040977 | 0.739481 |
| BP | GO:0035507 | regulation of myosin-light-chain-phosphatase activity | 4 | 7 | 0.040977 | 0.739481 |
| BP | GO:0043129 | surfactant homeostasis | 4 | 7 | 0.040977 | 0.739481 |
| BP | GO:0043248 | proteasome assembly | 4 | 7 | 0.040977 | 0.739481 |
| BP | GO:0045060 | negative thymic T cell selection | 4 | 7 | 0.040977 | 0.739481 |
| BP | GO:0045618 | positive regulation of keratinocyte differentiation | 4 | 7 | 0.040977 | 0.739481 |
| BP | GO:0072396 | response to cell cycle checkpoint signaling | 4 | 7 | 0.040977 | 0.739481 |
| BP | GO:0072402 | response to DNA integrity checkpoint signaling | 4 | 7 | 0.040977 | 0.739481 |
| BP | GO:0072423 | response to DNA damage checkpoint signaling | 4 | 7 | 0.040977 | 0.739481 |
| BP | GO:0072501 | cellular divalent inorganic anion homeostasis | 4 | 7 | 0.040977 | 0.739481 |
| BP | GO:0072502 | cellular trivalent inorganic anion homeostasis | 4 | 7 | 0.040977 | 0.739481 |
| BP | GO:0090673 | endothelial cell-matrix adhesion | 4 | 7 | 0.040977 | 0.739481 |
| BP | GO:0099612 | protein localization to axon | 4 | 7 | 0.040977 | 0.739481 |
| BP | GO:1904779 | regulation of protein localization to centrosome | 4 | 7 | 0.040977 | 0.739481 |
| BP | GO:0010769 | regulation of cell morphogenesis involved in differentiation | 39 | 141 | 0.041496 | 0.739481 |
| BP | GO:0071900 | regulation of protein serine/threonine kinase activity | 55 | 208 | 0.041522 | 0.739481 |
| BP | GO:0010528 | regulation of transposition | 5 | 10 | 0.041708 | 0.739481 |
| BP | GO:0010529 | negative regulation of transposition | 5 | 10 | 0.041708 | 0.739481 |
| BP | GO:0018195 | peptidyl-arginine modification | 5 | 10 | 0.041708 | 0.739481 |
| BP | GO:0021554 | optic nerve development | 5 | 10 | 0.041708 | 0.739481 |
| BP | GO:0033599 | regulation of mammary gland epithelial cell proliferation | 5 | 10 | 0.041708 | 0.739481 |
| BP | GO:0045736 | negative regulation of cyclin-dependent protein serine/threonine kinase activity | 5 | 10 | 0.041708 | 0.739481 |
| BP | GO:1904030 | negative regulation of cyclin-dependent protein kinase activity | 5 | 10 | 0.041708 | 0.739481 |
| BP | GO:0048812 | neuron projection morphogenesis | 83 | 328 | 0.041730 | 0.739481 |
| BP | GO:0034655 | nucleobase-containing compound catabolic process | 70 | 272 | 0.041763 | 0.739481 |
| BP | GO:0043086 | negative regulation of catalytic activity | 70 | 272 | 0.041763 | 0.739481 |
| CC | GO:0005826 | actomyosin contractile ring | 2 | 2 | 0.041832 | 0.739481 |
| CC | GO:0033093 | Weibel-Palade body | 2 | 2 | 0.041832 | 0.739481 |
| CC | GO:0042827 | platelet dense granule | 2 | 2 | 0.041832 | 0.739481 |
| CC | GO:0043159 | acrosomal matrix | 2 | 2 | 0.041832 | 0.739481 |
| CC | GO:0043259 | laminin-10 complex | 2 | 2 | 0.041832 | 0.739481 |
| CC | GO:0060171 | stereocilium membrane | 2 | 2 | 0.041832 | 0.739481 |
| CC | GO:0097059 | CNTFR-CLCF1 complex | 2 | 2 | 0.041832 | 0.739481 |
| CC | GO:0097165 | nuclear stress granule | 2 | 2 | 0.041832 | 0.739481 |
| CC | GO:0097208 | alveolar lamellar body | 2 | 2 | 0.041832 | 0.739481 |
| CC | GO:0097232 | lamellar body membrane | 2 | 2 | 0.041832 | 0.739481 |
| CC | GO:0097635 | extrinsic component of autophagosome membrane | 2 | 2 | 0.041832 | 0.739481 |
| BP | GO:0006220 | pyrimidine nucleotide metabolic process | 12 | 34 | 0.041905 | 0.739481 |
| BP | GO:0045058 | T cell selection | 12 | 34 | 0.041905 | 0.739481 |
| BP | GO:0060443 | mammary gland morphogenesis | 12 | 34 | 0.041905 | 0.739481 |
| MF | GO:0004518 | nuclease activity | 38 | 141 | 0.042003 | 0.739481 |
| BP | GO:0032271 | regulation of protein polymerization | 38 | 137 | 0.042059 | 0.739481 |
| MF | GO:0043177 | organic acid binding | 28 | 99 | 0.042267 | 0.739481 |
| BP | GO:0030193 | regulation of blood coagulation | 16 | 49 | 0.042311 | 0.739481 |
| MF | GO:0000827 | inositol-1,3,4,5,6-pentakisphosphate kinase activity | 2 | 2 | 0.042512 | 0.739481 |
| MF | GO:0000829 | inositol heptakisphosphate kinase activity | 2 | 2 | 0.042512 | 0.739481 |
| MF | GO:0001010 | RNA polymerase II sequence-specific DNA-binding transcription factor recruiting activity | 2 | 2 | 0.042512 | 0.739481 |
| MF | GO:0001147 | transcription termination site sequence-specific DNA binding | 2 | 2 | 0.042512 | 0.739481 |
| MF | GO:0001160 | transcription termination site DNA binding | 2 | 2 | 0.042512 | 0.739481 |
| MF | GO:0001641 | group II metabotropic glutamate receptor activity | 2 | 2 | 0.042512 | 0.739481 |
| MF | GO:0003880 | protein C-terminal carboxyl O-methyltransferase activity | 2 | 2 | 0.042512 | 0.739481 |
| MF | GO:0004450 | isocitrate dehydrogenase (NADP+) activity | 2 | 2 | 0.042512 | 0.739481 |
| MF | GO:0004534 | 5'-3' exoribonuclease activity | 2 | 2 | 0.042512 | 0.739481 |
| MF | GO:0004558 | alpha-1,4-glucosidase activity | 2 | 2 | 0.042512 | 0.739481 |
| MF | GO:0004817 | cysteine-tRNA ligase activity | 2 | 2 | 0.042512 | 0.739481 |
| MF | GO:0004818 | glutamate-tRNA ligase activity | 2 | 2 | 0.042512 | 0.739481 |
| MF | GO:0004972 | NMDA glutamate receptor activity | 2 | 2 | 0.042512 | 0.739481 |
| MF | GO:0005001 | transmembrane receptor protein tyrosine phosphatase activity | 2 | 2 | 0.042512 | 0.739481 |
| MF | GO:0005005 | transmembrane-ephrin receptor activity | 2 | 2 | 0.042512 | 0.739481 |
| MF | GO:0008126 | acetylesterase activity | 2 | 2 | 0.042512 | 0.739481 |
| MF | GO:0009378 | four-way junction helicase activity | 2 | 2 | 0.042512 | 0.739481 |
| MF | GO:0010484 | H3 histone acetyltransferase activity | 2 | 2 | 0.042512 | 0.739481 |
| MF | GO:0016316 | phosphatidylinositol-3,4-bisphosphate 4-phosphatase activity | 2 | 2 | 0.042512 | 0.739481 |
| MF | GO:0019198 | transmembrane receptor protein phosphatase activity | 2 | 2 | 0.042512 | 0.739481 |
| MF | GO:0022849 | glutamate-gated calcium ion channel activity | 2 | 2 | 0.042512 | 0.739481 |
| MF | GO:0032574 | 5'-3' RNA helicase activity | 2 | 2 | 0.042512 | 0.739481 |
| MF | GO:0033857 | diphosphoinositol-pentakisphosphate kinase activity | 2 | 2 | 0.042512 | 0.739481 |
| MF | GO:0034338 | short-chain carboxylesterase activity | 2 | 2 | 0.042512 | 0.739481 |
| MF | GO:0034416 | bisphosphoglycerate phosphatase activity | 2 | 2 | 0.042512 | 0.739481 |
| MF | GO:0036384 | cytidine diphosphatase activity | 2 | 2 | 0.042512 | 0.739481 |
| MF | GO:0043140 | ATP-dependent 3'-5' DNA helicase activity | 2 | 2 | 0.042512 | 0.739481 |
| MF | GO:0050656 | 3'-phosphoadenosine 5'-phosphosulfate binding | 2 | 2 | 0.042512 | 0.739481 |
| MF | GO:0050659 | N-acetylgalactosamine 4-sulfate 6-O-sulfotransferase activity | 2 | 2 | 0.042512 | 0.739481 |
| MF | GO:0052723 | inositol hexakisphosphate 1-kinase activity | 2 | 2 | 0.042512 | 0.739481 |
| MF | GO:0052724 | inositol hexakisphosphate 3-kinase activity | 2 | 2 | 0.042512 | 0.739481 |
| MF | GO:0061749 | forked DNA-dependent helicase activity | 2 | 2 | 0.042512 | 0.739481 |
| MF | GO:0070336 | flap-structured DNA binding | 2 | 2 | 0.042512 | 0.739481 |
| MF | GO:0070853 | myosin VI binding | 2 | 2 | 0.042512 | 0.739481 |
| MF | GO:0072345 | NAADP-sensitive calcium-release channel activity | 2 | 2 | 0.042512 | 0.739481 |
| MF | GO:0102092 | 5-diphosphoinositol pentakisphosphate 3-kinase activity | 2 | 2 | 0.042512 | 0.739481 |
| MF | GO:0120014 | intermembrane phospholipid transfer activity | 2 | 2 | 0.042512 | 0.739481 |
| MF | GO:0120019 | intermembrane phosphotidylcholine transfer activity | 2 | 2 | 0.042512 | 0.739481 |
| MF | GO:1990518 | single-stranded DNA-dependent ATP-dependent 3'-5' DNA helicase activity | 2 | 2 | 0.042512 | 0.739481 |
| MF | GO:0005548 | phospholipid transporter activity | 9 | 24 | 0.043041 | 0.739481 |
| MF | GO:0016896 | exoribonuclease activity, producing 5'-phosphomonoesters | 9 | 24 | 0.043041 | 0.739481 |
| CC | GO:0099080 | supramolecular complex | 110 | 463 | 0.043076 | 0.739481 |
| CC | GO:0099081 | supramolecular polymer | 110 | 463 | 0.043076 | 0.739481 |
| BP | GO:0006260 | DNA replication | 43 | 158 | 0.043098 | 0.739481 |
| BP | GO:0007160 | cell-matrix adhesion | 36 | 129 | 0.043152 | 0.739481 |
| BP | GO:0010810 | regulation of cell-substrate adhesion | 36 | 129 | 0.043152 | 0.739481 |
| CC | GO:0031256 | leading edge membrane | 21 | 71 | 0.043235 | 0.739481 |
| BP | GO:0032637 | interleukin-8 production | 17 | 53 | 0.043634 | 0.739481 |
| BP | GO:0032677 | regulation of interleukin-8 production | 17 | 53 | 0.043634 | 0.739481 |
| BP | GO:0018205 | peptidyl-lysine modification | 52 | 196 | 0.043943 | 0.739481 |
| MF | GO:0005261 | cation channel activity | 58 | 228 | 0.044057 | 0.739481 |
| CC | GO:0016605 | PML body | 17 | 55 | 0.044123 | 0.739481 |
| BP | GO:0007163 | establishment or maintenance of cell polarity | 34 | 121 | 0.044181 | 0.739481 |
| MF | GO:0005515 | protein binding | 938 | 4372 | 0.044473 | 0.739481 |
| BP | GO:0000731 | DNA synthesis involved in DNA repair | 8 | 20 | 0.044520 | 0.739481 |
| BP | GO:0019229 | regulation of vasoconstriction | 8 | 20 | 0.044520 | 0.739481 |
| BP | GO:0021602 | cranial nerve morphogenesis | 8 | 20 | 0.044520 | 0.739481 |
| BP | GO:0045471 | response to ethanol | 8 | 20 | 0.044520 | 0.739481 |
| MF | GO:0008289 | lipid binding | 100 | 415 | 0.044574 | 0.739481 |
| BP | GO:0045913 | positive regulation of carbohydrate metabolic process | 13 | 38 | 0.044744 | 0.739481 |
| BP | GO:0060119 | inner ear receptor cell development | 13 | 38 | 0.044744 | 0.739481 |
| BP | GO:0097306 | cellular response to alcohol | 13 | 38 | 0.044744 | 0.739481 |
| MF | GO:0004175 | endopeptidase activity | 81 | 330 | 0.044953 | 0.739481 |
| BP | GO:0000393 | spliceosomal conformational changes to generate catalytic conformation | 2 | 2 | 0.045144 | 0.739481 |
| BP | GO:0002249 | lymphocyte anergy | 2 | 2 | 0.045144 | 0.739481 |
| BP | GO:0002458 | peripheral T cell tolerance induction | 2 | 2 | 0.045144 | 0.739481 |
| BP | GO:0002667 | regulation of T cell anergy | 2 | 2 | 0.045144 | 0.739481 |
| BP | GO:0002669 | positive regulation of T cell anergy | 2 | 2 | 0.045144 | 0.739481 |
| BP | GO:0002677 | negative regulation of chronic inflammatory response | 2 | 2 | 0.045144 | 0.739481 |
| BP | GO:0002870 | T cell anergy | 2 | 2 | 0.045144 | 0.739481 |
| BP | GO:0002911 | regulation of lymphocyte anergy | 2 | 2 | 0.045144 | 0.739481 |
| BP | GO:0002913 | positive regulation of lymphocyte anergy | 2 | 2 | 0.045144 | 0.739481 |
| BP | GO:0003010 | voluntary skeletal muscle contraction | 2 | 2 | 0.045144 | 0.739481 |
| BP | GO:0003290 | atrial septum secundum morphogenesis | 2 | 2 | 0.045144 | 0.739481 |
| BP | GO:0006097 | glyoxylate cycle | 2 | 2 | 0.045144 | 0.739481 |
| BP | GO:0006102 | isocitrate metabolic process | 2 | 2 | 0.045144 | 0.739481 |
| BP | GO:0006423 | cysteinyl-tRNA aminoacylation | 2 | 2 | 0.045144 | 0.739481 |
| BP | GO:0006424 | glutamyl-tRNA aminoacylation | 2 | 2 | 0.045144 | 0.739481 |
| BP | GO:0006649 | phospholipid transfer to membrane | 2 | 2 | 0.045144 | 0.739481 |
| BP | GO:0006876 | cellular cadmium ion homeostasis | 2 | 2 | 0.045144 | 0.739481 |
| BP | GO:0007198 | adenylate cyclase-inhibiting serotonin receptor signaling pathway | 2 | 2 | 0.045144 | 0.739481 |
| BP | GO:0007227 | signal transduction downstream of smoothened | 2 | 2 | 0.045144 | 0.739481 |
| BP | GO:0007418 | ventral midline development | 2 | 2 | 0.045144 | 0.739481 |
| BP | GO:0010265 | SCF complex assembly | 2 | 2 | 0.045144 | 0.739481 |
| BP | GO:0014062 | regulation of serotonin secretion | 2 | 2 | 0.045144 | 0.739481 |
| BP | GO:0014721 | twitch skeletal muscle contraction | 2 | 2 | 0.045144 | 0.739481 |
| BP | GO:0014724 | regulation of twitch skeletal muscle contraction | 2 | 2 | 0.045144 | 0.739481 |
| BP | GO:0014916 | regulation of lung blood pressure | 2 | 2 | 0.045144 | 0.739481 |
| BP | GO:0019483 | beta-alanine biosynthetic process | 2 | 2 | 0.045144 | 0.739481 |
| BP | GO:0021564 | vagus nerve development | 2 | 2 | 0.045144 | 0.739481 |
| BP | GO:0021812 | neuronal-glial interaction involved in cerebral cortex radial glia guided migration | 2 | 2 | 0.045144 | 0.739481 |
| BP | GO:0021847 | ventricular zone neuroblast division | 2 | 2 | 0.045144 | 0.739481 |
| BP | GO:0031443 | fast-twitch skeletal muscle fiber contraction | 2 | 2 | 0.045144 | 0.739481 |
| BP | GO:0031446 | regulation of fast-twitch skeletal muscle fiber contraction | 2 | 2 | 0.045144 | 0.739481 |
| BP | GO:0031448 | positive regulation of fast-twitch skeletal muscle fiber contraction | 2 | 2 | 0.045144 | 0.739481 |
| BP | GO:0032877 | positive regulation of DNA endoreduplication | 2 | 2 | 0.045144 | 0.739481 |
| BP | GO:0033058 | directional locomotion | 2 | 2 | 0.045144 | 0.739481 |
| BP | GO:0035544 | negative regulation of SNARE complex assembly | 2 | 2 | 0.045144 | 0.739481 |
| BP | GO:0035722 | interleukin-12-mediated signaling pathway | 2 | 2 | 0.045144 | 0.739481 |
| BP | GO:0035898 | parathyroid hormone secretion | 2 | 2 | 0.045144 | 0.739481 |
| BP | GO:0035989 | tendon development | 2 | 2 | 0.045144 | 0.739481 |
| BP | GO:0036482 | neuron intrinsic apoptotic signaling pathway in response to hydrogen peroxide | 2 | 2 | 0.045144 | 0.739481 |
| BP | GO:0036506 | maintenance of unfolded protein | 2 | 2 | 0.045144 | 0.739481 |
| BP | GO:0042262 | DNA protection | 2 | 2 | 0.045144 | 0.739481 |
| BP | GO:0042351 | 'de novo' GDP-L-fucose biosynthetic process | 2 | 2 | 0.045144 | 0.739481 |
| BP | GO:0042496 | detection of diacyl bacterial lipopeptide | 2 | 2 | 0.045144 | 0.739481 |
| BP | GO:0043578 | nuclear matrix organization | 2 | 2 | 0.045144 | 0.739481 |
| BP | GO:0044211 | CTP salvage | 2 | 2 | 0.045144 | 0.739481 |
| BP | GO:0046710 | GDP metabolic process | 2 | 2 | 0.045144 | 0.739481 |
| BP | GO:0046712 | GDP catabolic process | 2 | 2 | 0.045144 | 0.739481 |
| BP | GO:0050915 | sensory perception of sour taste | 2 | 2 | 0.045144 | 0.739481 |
| BP | GO:0051389 | inactivation of MAPKK activity | 2 | 2 | 0.045144 | 0.739481 |
| BP | GO:0051673 | membrane disruption in other organism | 2 | 2 | 0.045144 | 0.739481 |
| BP | GO:0060032 | notochord regression | 2 | 2 | 0.045144 | 0.739481 |
| BP | GO:0060168 | positive regulation of adenosine receptor signaling pathway | 2 | 2 | 0.045144 | 0.739481 |
| BP | GO:0061034 | olfactory bulb mitral cell layer development | 2 | 2 | 0.045144 | 0.739481 |
| BP | GO:0061052 | negative regulation of cell growth involved in cardiac muscle cell development | 2 | 2 | 0.045144 | 0.739481 |
| BP | GO:0070358 | actin polymerization-dependent cell motility | 2 | 2 | 0.045144 | 0.739481 |
| BP | GO:0070994 | detection of oxidative stress | 2 | 2 | 0.045144 | 0.739481 |
| BP | GO:0071879 | positive regulation of adenylate cyclase-activating adrenergic receptor signaling pathway | 2 | 2 | 0.045144 | 0.739481 |
| BP | GO:0086036 | regulation of cardiac muscle cell membrane potential | 2 | 2 | 0.045144 | 0.739481 |
| BP | GO:0090292 | nuclear matrix anchoring at nuclear membrane | 2 | 2 | 0.045144 | 0.739481 |
| BP | GO:0090675 | intermicrovillar adhesion | 2 | 2 | 0.045144 | 0.739481 |
| BP | GO:0097050 | type B pancreatic cell apoptotic process | 2 | 2 | 0.045144 | 0.739481 |
| BP | GO:0098928 | presynaptic signal transduction | 2 | 2 | 0.045144 | 0.739481 |
| BP | GO:0099526 | presynapse to nucleus signaling pathway | 2 | 2 | 0.045144 | 0.739481 |
| BP | GO:0110112 | regulation of lipid transporter activity | 2 | 2 | 0.045144 | 0.739481 |
| BP | GO:0110113 | positive regulation of lipid transporter activity | 2 | 2 | 0.045144 | 0.739481 |
| BP | GO:1902954 | regulation of early endosome to recycling endosome transport | 2 | 2 | 0.045144 | 0.739481 |
| BP | GO:1903383 | regulation of hydrogen peroxide-induced neuron intrinsic apoptotic signaling pathway | 2 | 2 | 0.045144 | 0.739481 |
| BP | GO:1903384 | negative regulation of hydrogen peroxide-induced neuron intrinsic apoptotic signaling pathway | 2 | 2 | 0.045144 | 0.739481 |
| BP | GO:1903750 | regulation of intrinsic apoptotic signaling pathway in response to hydrogen peroxide | 2 | 2 | 0.045144 | 0.739481 |
| BP | GO:1903751 | negative regulation of intrinsic apoptotic signaling pathway in response to hydrogen peroxide | 2 | 2 | 0.045144 | 0.739481 |
| BP | GO:1904170 | regulation of bleb assembly | 2 | 2 | 0.045144 | 0.739481 |
| BP | GO:1904172 | positive regulation of bleb assembly | 2 | 2 | 0.045144 | 0.739481 |
| BP | GO:1904378 | maintenance of unfolded protein involved in ERAD pathway | 2 | 2 | 0.045144 | 0.739481 |
| BP | GO:1904464 | regulation of matrix metallopeptidase secretion | 2 | 2 | 0.045144 | 0.739481 |
| BP | GO:1904466 | positive regulation of matrix metallopeptidase secretion | 2 | 2 | 0.045144 | 0.739481 |
| BP | GO:1904627 | response to phorbol 13-acetate 12-myristate | 2 | 2 | 0.045144 | 0.739481 |
| BP | GO:1904628 | cellular response to phorbol 13-acetate 12-myristate | 2 | 2 | 0.045144 | 0.739481 |
| BP | GO:1904743 | negative regulation of telomeric DNA binding | 2 | 2 | 0.045144 | 0.739481 |
| BP | GO:1905832 | positive regulation of spindle assembly | 2 | 2 | 0.045144 | 0.739481 |
| BP | GO:1990705 | cholangiocyte proliferation | 2 | 2 | 0.045144 | 0.739481 |
| BP | GO:1990773 | matrix metallopeptidase secretion | 2 | 2 | 0.045144 | 0.739481 |
| BP | GO:2000405 | negative regulation of T cell migration | 2 | 2 | 0.045144 | 0.739481 |
| BP | GO:2000640 | positive regulation of SREBP signaling pathway | 2 | 2 | 0.045144 | 0.739481 |
| BP | GO:2001027 | negative regulation of endothelial cell chemotaxis | 2 | 2 | 0.045144 | 0.739481 |
| BP | GO:2001226 | negative regulation of chloride transport | 2 | 2 | 0.045144 | 0.739481 |
| MF | GO:0051015 | actin filament binding | 29 | 104 | 0.046577 | 0.739481 |
| BP | GO:0009166 | nucleotide catabolic process | 28 | 97 | 0.046588 | 0.739481 |
| BP | GO:0042552 | myelination | 21 | 69 | 0.046600 | 0.739481 |
| MF | GO:0099094 | ligand-gated cation channel activity | 20 | 67 | 0.046980 | 0.739481 |
| BP | GO:0050672 | negative regulation of lymphocyte proliferation | 14 | 42 | 0.047046 | 0.739481 |
| BP | GO:0000723 | telomere maintenance | 25 | 85 | 0.047130 | 0.739481 |
| BP | GO:0032200 | telomere organization | 25 | 85 | 0.047130 | 0.739481 |
| BP | GO:0051053 | negative regulation of DNA metabolic process | 25 | 85 | 0.047130 | 0.739481 |
| BP | GO:0032273 | positive regulation of protein polymerization | 24 | 81 | 0.047158 | 0.739481 |
| BP | GO:0022008 | neurogenesis | 212 | 902 | 0.047169 | 0.739481 |
| BP | GO:0010639 | negative regulation of organelle organization | 64 | 248 | 0.047295 | 0.739481 |
| MF | GO:0022838 | substrate-specific channel activity | 79 | 322 | 0.047619 | 0.739481 |
| BP | GO:0016569 | covalent chromatin modification | 60 | 231 | 0.047670 | 0.739481 |
| BP | GO:0000226 | microtubule cytoskeleton organization | 94 | 378 | 0.047789 | 0.739481 |
| MF | GO:0097159 | organic cyclic compound binding | 818 | 3799 | 0.047921 | 0.739481 |
| BP | GO:0051129 | negative regulation of cellular component organization | 107 | 435 | 0.048032 | 0.739481 |
| CC | GO:0097730 | non-motile cilium | 23 | 80 | 0.048095 | 0.739481 |
| BP | GO:0030029 | actin filament-based process | 112 | 457 | 0.048112 | 0.739481 |
| CC | GO:1990752 | microtubule end | 8 | 21 | 0.048359 | 0.739481 |
| BP | GO:0048513 | animal organ development | 450 | 1986 | 0.048417 | 0.739481 |
| BP | GO:0051496 | positive regulation of stress fiber assembly | 11 | 31 | 0.048486 | 0.739481 |
| BP | GO:0033047 | regulation of mitotic sister chromatid segregation | 15 | 46 | 0.048894 | 0.739481 |
| BP | GO:1903312 | negative regulation of mRNA metabolic process | 15 | 46 | 0.048894 | 0.739481 |
| BP | GO:0031346 | positive regulation of cell projection organization | 46 | 172 | 0.049106 | 0.739481 |

**Table S17** The KEGG enrichment results of unique SNPs in Tibetan sheep (*P* < 0.05)

| **Pathway ID** | **Pathway** | **List_number** | **Total_number** | ***P_*value** | **FDR** |
| --- | --- | --- | --- | --- | --- |
| ko04740 | Olfactory transduction | 719 | 930 | 0.000001 | 0.000417 |
| ko04974 | Protein digestion and absorption | 113 | 129 | 0.000004 | 0.000645 |
| ko04512 | ECM-receptor interaction | 81 | 90 | 0.000008 | 0.000966 |
| ko02010 | ABC transporters | 51 | 56 | 0.000201 | 0.017825 |
| ko00562 | Inositol phosphate metabolism | 65 | 74 | 0.000375 | 0.026530 |
| ko04610 | Complement and coagulation cascades | 74 | 86 | 0.000621 | 0.036647 |
| ko04724 | Glutamatergic synapse | 91 | 109 | 0.001345 | 0.068018 |
| ko04976 | Bile secretion | 82 | 98 | 0.002013 | 0.089078 |
| ko04973 | Carbohydrate digestion and absorption | 40 | 45 | 0.003228 | 0.126955 |
| ko04725 | Cholinergic synapse | 89 | 109 | 0.005666 | 0.200592 |
| ko00500 | Starch and sucrose metabolism | 29 | 32 | 0.006555 | 0.210954 |
| ko04072 | Phospholipase D signaling pathway | 119 | 150 | 0.009711 | 0.277640 |
| ko00561 | Glycerolipid metabolism | 56 | 67 | 0.010658 | 0.277640 |
| ko00052 | Galactose metabolism | 27 | 30 | 0.010980 | 0.277640 |
| ko00970 | Aminoacyl-tRNA biosynthesis | 38 | 44 | 0.012168 | 0.287173 |
| ko04611 | Platelet activation | 98 | 123 | 0.014543 | 0.321766 |
| ko04070 | Phosphatidylinositol signaling system | 78 | 97 | 0.018779 | 0.391044 |
| ko00051 | Fructose and mannose metabolism | 28 | 32 | 0.022079 | 0.415500 |
| ko05230 | Central carbon metabolism in cancer | 58 | 71 | 0.023195 | 0.415500 |
| ko00100 | Steroid biosynthesis | 20 | 22 | 0.023475 | 0.415500 |
| ko00410 | beta-Alanine metabolism | 31 | 36 | 0.025391 | 0.428021 |
| ko04915 | Estrogen signaling pathway | 106 | 136 | 0.033398 | 0.537407 |
| ko05412 | Arrhythmogenic right ventricular cardiomyopathy | 61 | 76 | 0.037966 | 0.559991 |
| ko04971 | Gastric acid secretion | 61 | 76 | 0.037966 | 0.559991 |
| ko03460 | Fanconi anemia pathway | 42 | 51 | 0.040365 | 0.571573 |
| ko04911 | Insulin secretion | 69 | 87 | 0.043571 | 0.593234 |
| ko00640 | Propanoate metabolism | 28 | 33 | 0.047691 | 0.625276 |

**Table S18** The GO enrichment results of unique SNPs in Tibetan sheep (*P* < 0.05)

| **Category** | **GO.ID** | **Term** | **list** | **Total** | ***P_*value** | **FDR** |
| --- | --- | --- | --- | --- | --- | --- |
| MF | GO:0032559 | adenyl ribonucleotide binding | 1009 | 1231 | 2.24E-18 | 2.33E-14 |
| MF | GO:0005524 | ATP binding | 985 | 1200 | 2.63E-18 | 2.33E-14 |
| MF | GO:0030554 | adenyl nucleotide binding | 1010 | 1235 | 8.31E-18 | 4.90E-14 |
| MF | GO:0008144 | drug binding | 1077 | 1331 | 3.41E-16 | 1.51E-12 |
| MF | GO:0043167 | ion binding | 3088 | 4071 | 4.98E-13 | 1.76E-09 |
| MF | GO:0003774 | motor activity | 87 | 89 | 9.23E-11 | 2.72E-07 |
| MF | GO:0043168 | anion binding | 1587 | 2063 | 4.62E-09 | 0.000010 |
| MF | GO:0046872 | metal ion binding | 1847 | 2416 | 5.04E-09 | 0.000010 |
| MF | GO:0097367 | carbohydrate derivative binding | 1303 | 1680 | 5.09E-09 | 0.000010 |
| CC | GO:0005856 | cytoskeleton | 1057 | 1366 | 7.70E-09 | 0.000014 |
| MF | GO:0038023 | signaling receptor activity | 840 | 1062 | 8.65E-09 | 0.000014 |
| MF | GO:0003824 | catalytic activity | 3304 | 4419 | 1.54E-08 | 0.000023 |
| MF | GO:0000166 | nucleotide binding | 1332 | 1725 | 2.36E-08 | 0.000030 |
| MF | GO:1901265 | nucleoside phosphate binding | 1332 | 1725 | 2.36E-08 | 0.000030 |
| MF | GO:0043169 | cation binding | 1871 | 2457 | 3.16E-08 | 0.000037 |
| MF | GO:0060089 | molecular transducer activity | 851 | 1081 | 3.51E-08 | 0.000039 |
| CC | GO:0071944 | cell periphery | 2232 | 2984 | 3.88E-08 | 0.000040 |
| MF | GO:0032555 | purine ribonucleotide binding | 1173 | 1513 | 4.20E-08 | 0.000041 |
| MF | GO:0035639 | purine ribonucleoside triphosphate binding | 1146 | 1477 | 4.57E-08 | 0.000041 |
| MF | GO:0004888 | transmembrane signaling receptor activity | 773 | 978 | 4.60E-08 | 0.000041 |
| MF | GO:0042623 | ATPase activity, coupled | 200 | 231 | 5.53E-08 | 0.000047 |
| CC | GO:0005886 | plasma membrane | 2176 | 2910 | 7.41E-08 | 0.000060 |
| MF | GO:0032553 | ribonucleotide binding | 1179 | 1524 | 8.94E-08 | 0.000069 |
| MF | GO:0016887 | ATPase activity | 268 | 319 | 1.68E-07 | 0.000124 |
| MF | GO:0017076 | purine nucleotide binding | 1176 | 1523 | 1.92E-07 | 0.000136 |
| CC | GO:0044430 | cytoskeletal part | 790 | 1019 | 4.35E-07 | 0.000296 |
| MF | GO:0016787 | hydrolase activity | 1511 | 1981 | 0.000001 | 0.000361 |
| MF | GO:0005085 | guanyl-nucleotide exchange factor activity | 151 | 173 | 0.000001 | 0.000503 |
| MF | GO:0004714 | transmembrane receptor protein tyrosine kinase activity | 79 | 85 | 0.000001 | 0.000676 |
| MF | GO:0003777 | microtubule motor activity | 55 | 57 | 0.000002 | 0.000947 |
| MF | GO:1990939 | ATP-dependent microtubule motor activity | 55 | 57 | 0.000002 | 0.000947 |
| MF | GO:0051020 | GTPase binding | 282 | 343 | 0.000004 | 0.002221 |
| MF | GO:0036094 | small molecule binding | 1482 | 1952 | 0.000005 | 0.002423 |
| CC | GO:0005903 | brush border | 49 | 51 | 0.000006 | 0.002891 |
| CC | GO:0120025 | plasma membrane bounded cell projection | 794 | 1034 | 0.000006 | 0.002891 |
| MF | GO:0022804 | active transmembrane transporter activity | 200 | 238 | 0.000006 | 0.002961 |
| MF | GO:0019199 | transmembrane receptor protein kinase activity | 91 | 101 | 0.000006 | 0.003064 |
| CC | GO:0042995 | cell projection | 822 | 1074 | 0.000009 | 0.004305 |
| CC | GO:0044450 | microtubule organizing center part | 153 | 181 | 0.000013 | 0.005866 |
| MF | GO:0140097 | catalytic activity, acting on DNA | 125 | 144 | 0.000013 | 0.005892 |
| CC | GO:0044463 | cell projection part | 528 | 680 | 0.000029 | 0.012082 |
| CC | GO:0120038 | plasma membrane bounded cell projection part | 528 | 680 | 0.000029 | 0.012082 |
| MF | GO:0015081 | sodium ion transmembrane transporter activity | 80 | 89 | 0.000029 | 0.012082 |
| MF | GO:0004713 | protein tyrosine kinase activity | 88 | 99 | 0.000034 | 0.013622 |
| CC | GO:0005581 | collagen trimer | 37 | 38 | 0.000035 | 0.013622 |
| MF | GO:0008092 | cytoskeletal protein binding | 477 | 607 | 0.000060 | 0.022956 |
| MF | GO:0015291 | secondary active transmembrane transporter activity | 123 | 144 | 0.000083 | 0.031175 |
| MF | GO:0016788 | hydrolase activity, acting on ester bonds | 428 | 543 | 0.000086 | 0.031779 |
| CC | GO:0016020 | membrane | 4494 | 6202 | 0.000094 | 0.033735 |
| CC | GO:0098862 | cluster of actin-based cell projections | 76 | 86 | 0.000095 | 0.033735 |
| MF | GO:0016301 | kinase activity | 458 | 584 | 0.000119 | 0.040591 |
| CC | GO:0099513 | polymeric cytoskeletal fiber | 268 | 336 | 0.000120 | 0.040591 |
| MF | GO:0016874 | ligase activity | 102 | 118 | 0.000121 | 0.040591 |
| BP | GO:0007156 | homophilic cell adhesion via plasma membrane adhesion molecules | 64 | 71 | 0.000129 | 0.041871 |
| BP | GO:0046777 | protein autophosphorylation | 119 | 140 | 0.000130 | 0.041871 |
| CC | GO:0031224 | intrinsic component of membrane | 3156 | 4326 | 0.000140 | 0.044379 |
| MF | GO:0016773 | phosphotransferase activity, alcohol group as acceptor | 419 | 533 | 0.000158 | 0.048961 |
| CC | GO:0031012 | extracellular matrix | 165 | 201 | 0.000168 | 0.051341 |
| CC | GO:0016021 | integral component of membrane | 3116 | 4272 | 0.000177 | 0.052986 |
| MF | GO:0022857 | transmembrane transporter activity | 647 | 839 | 0.000186 | 0.054797 |
| CC | GO:0015630 | microtubule cytoskeleton | 603 | 790 | 0.000251 | 0.072754 |
| BP | GO:0007010 | cytoskeleton organization | 630 | 821 | 0.000291 | 0.083232 |
| CC | GO:0044441 | ciliary part | 243 | 306 | 0.000408 | 0.113406 |
| BP | GO:0046503 | glycerolipid catabolic process | 30 | 31 | 0.000410 | 0.113406 |
| CC | GO:0005604 | basement membrane | 53 | 59 | 0.000439 | 0.119436 |
| CC | GO:0044425 | membrane part | 3602 | 4962 | 0.000445 | 0.119436 |
| CC | GO:0044304 | main axon | 28 | 29 | 0.000599 | 0.158105 |
| MF | GO:0016772 | transferase activity, transferring phosphorus-containing groups | 531 | 688 | 0.000612 | 0.158105 |
| MF | GO:0005215 | transporter activity | 677 | 885 | 0.000631 | 0.158105 |
| MF | GO:0003779 | actin binding | 218 | 271 | 0.000634 | 0.158105 |
| BP | GO:0022610 | biological adhesion | 572 | 746 | 0.000634 | 0.158105 |
| CC | GO:0099080 | supramolecular complex | 359 | 463 | 0.000653 | 0.158508 |
| CC | GO:0099081 | supramolecular polymer | 359 | 463 | 0.000653 | 0.158508 |
| MF | GO:0005509 | calcium ion binding | 386 | 494 | 0.000674 | 0.161367 |
| MF | GO:0051015 | actin filament binding | 89 | 104 | 0.000693 | 0.163676 |
| MF | GO:0004715 | non-membrane spanning protein tyrosine kinase activity | 34 | 36 | 0.000740 | 0.170769 |
| CC | GO:0098590 | plasma membrane region | 451 | 588 | 0.000747 | 0.170769 |
| BP | GO:0007155 | cell adhesion | 567 | 740 | 0.000752 | 0.170769 |
| CC | GO:0044459 | plasma membrane part | 946 | 1266 | 0.000822 | 0.182089 |
| CC | GO:0070161 | anchoring junction | 148 | 182 | 0.000827 | 0.182089 |
| MF | GO:0038024 | cargo receptor activity | 48 | 53 | 0.000833 | 0.182089 |
| CC | GO:0030055 | cell-substrate junction | 98 | 117 | 0.000908 | 0.196184 |
| MF | GO:0046873 | metal ion transmembrane transporter activity | 230 | 288 | 0.000996 | 0.212477 |
| CC | GO:0099512 | supramolecular fiber | 354 | 458 | 0.001063 | 0.224077 |
| MF | GO:0004672 | protein kinase activity | 342 | 437 | 0.001102 | 0.229526 |
| MF | GO:0071949 | FAD binding | 27 | 28 | 0.001122 | 0.231126 |
| CC | GO:0005912 | adherens junction | 139 | 171 | 0.001216 | 0.247564 |
| BP | GO:0007018 | microtubule-based movement | 146 | 179 | 0.001272 | 0.255984 |
| BP | GO:0007041 | lysosomal transport | 54 | 61 | 0.001297 | 0.257996 |
| MF | GO:0008066 | glutamate receptor activity | 20 | 20 | 0.001327 | 0.261079 |
| MF | GO:0008509 | anion transmembrane transporter activity | 176 | 218 | 0.001488 | 0.289536 |
| BP | GO:0048066 | developmental pigmentation | 31 | 33 | 0.001539 | 0.293317 |
| CC | GO:0062023 | collagen-containing extracellular matrix | 105 | 127 | 0.001544 | 0.293317 |
| CC | GO:0005815 | microtubule organizing center | 381 | 496 | 0.001557 | 0.293317 |
| BP | GO:0044242 | cellular lipid catabolic process | 101 | 121 | 0.001587 | 0.295854 |
| CC | GO:0044420 | extracellular matrix component | 30 | 32 | 0.001615 | 0.298019 |
| CC | GO:0005874 | microtubule | 165 | 206 | 0.001642 | 0.299829 |
| MF | GO:0005201 | extracellular matrix structural constituent | 31 | 33 | 0.001702 | 0.307566 |
| BP | GO:0030318 | melanocyte differentiation | 19 | 19 | 0.001720 | 0.307627 |
| MF | GO:0004386 | helicase activity | 105 | 126 | 0.001793 | 0.315702 |
| CC | GO:0005924 | cell-substrate adherens junction | 94 | 113 | 0.001818 | 0.315702 |
| MF | GO:0015293 | symporter activity | 65 | 75 | 0.001818 | 0.315702 |
| BP | GO:0016042 | lipid catabolic process | 159 | 197 | 0.001944 | 0.334203 |
| CC | GO:0005814 | centriole | 76 | 90 | 0.002120 | 0.357692 |
| MF | GO:0004930 | G protein-coupled receptor activity | 518 | 676 | 0.002121 | 0.357692 |
| CC | GO:0005925 | focal adhesion | 93 | 112 | 0.002154 | 0.359913 |
| BP | GO:0043087 | regulation of GTPase activity | 208 | 262 | 0.002175 | 0.359915 |
| MF | GO:0005272 | sodium channel activity | 30 | 32 | 0.002239 | 0.367174 |
| CC | GO:0099568 | cytoplasmic region | 186 | 235 | 0.002485 | 0.401722 |
| BP | GO:0050931 | pigment cell differentiation | 24 | 25 | 0.002520 | 0.401722 |
| MF | GO:0022836 | gated channel activity | 200 | 251 | 0.002540 | 0.401722 |
| BP | GO:0044782 | cilium organization | 182 | 228 | 0.002541 | 0.401722 |
| MF | GO:0019200 | carbohydrate kinase activity | 18 | 18 | 0.002575 | 0.403573 |
| MF | GO:0015075 | ion transmembrane transporter activity | 478 | 623 | 0.002613 | 0.405981 |
| CC | GO:0045111 | intermediate filament cytoskeleton | 95 | 115 | 0.002687 | 0.413738 |
| MF | GO:0008017 | microtubule binding | 139 | 171 | 0.002735 | 0.417484 |
| CC | GO:0005887 | integral component of plasma membrane | 416 | 546 | 0.002789 | 0.420813 |
| BP | GO:0120031 | plasma membrane bounded cell projection assembly | 251 | 320 | 0.002804 | 0.420813 |
| MF | GO:0000287 | magnesium ion binding | 132 | 162 | 0.002911 | 0.433270 |
| MF | GO:0070615 | nucleosome-dependent ATPase activity | 29 | 31 | 0.002941 | 0.434008 |
| BP | GO:0030031 | cell projection assembly | 253 | 323 | 0.003098 | 0.451156 |
| BP | GO:0042116 | macrophage activation | 46 | 52 | 0.003108 | 0.451156 |
| BP | GO:0007017 | microtubule-based process | 395 | 514 | 0.003261 | 0.469537 |
| BP | GO:0000723 | telomere maintenance | 72 | 85 | 0.003387 | 0.479802 |
| BP | GO:0032200 | telomere organization | 72 | 85 | 0.003387 | 0.479802 |
| BP | GO:0060271 | cilium assembly | 170 | 213 | 0.003511 | 0.493544 |
| MF | GO:0008569 | ATP-dependent microtubule motor activity, minus-end-directed | 17 | 17 | 0.003587 | 0.496320 |
| MF | GO:0042910 | xenobiotic transmembrane transporter activity | 17 | 17 | 0.003587 | 0.496320 |
| MF | GO:0008237 | metallopeptidase activity | 133 | 164 | 0.003949 | 0.542103 |
| MF | GO:0022839 | ion gated channel activity | 196 | 247 | 0.003982 | 0.542485 |
| BP | GO:0043547 | positive regulation of GTPase activity | 175 | 220 | 0.004039 | 0.546071 |
| BP | GO:0046434 | organophosphate catabolic process | 118 | 145 | 0.004195 | 0.562881 |
| MF | GO:0005216 | ion channel activity | 247 | 315 | 0.004292 | 0.571471 |
| CC | GO:0031226 | intrinsic component of plasma membrane | 446 | 589 | 0.004374 | 0.578075 |
| BP | GO:0072523 | purine-containing compound catabolic process | 27 | 29 | 0.004629 | 0.607291 |
| BP | GO:0051056 | regulation of small GTPase mediated signal transduction | 103 | 126 | 0.005559 | 0.723862 |
| CC | GO:0005938 | cell cortex | 103 | 127 | 0.005760 | 0.727475 |
| MF | GO:0008081 | phosphoric diester hydrolase activity | 59 | 69 | 0.005770 | 0.727475 |
| CC | GO:0030425 | dendrite | 152 | 192 | 0.005823 | 0.727475 |
| CC | GO:0097447 | dendritic tree | 152 | 192 | 0.005823 | 0.727475 |
| CC | GO:0005882 | intermediate filament | 67 | 80 | 0.005830 | 0.727475 |
| CC | GO:0005929 | cilium | 310 | 405 | 0.005833 | 0.727475 |
| BP | GO:0098742 | cell-cell adhesion via plasma-membrane adhesion molecules | 96 | 117 | 0.005918 | 0.732529 |
| BP | GO:0006259 | DNA metabolic process | 469 | 617 | 0.005956 | 0.732529 |
| BP | GO:0015914 | phospholipid transport | 26 | 28 | 0.006067 | 0.738826 |
| MF | GO:0050660 | flavin adenine dinucleotide binding | 55 | 64 | 0.006091 | 0.738826 |
| CC | GO:0043005 | neuron projection | 373 | 491 | 0.006188 | 0.745543 |
| CC | GO:0014069 | postsynaptic density | 96 | 118 | 0.006352 | 0.754979 |
| CC | GO:0032279 | asymmetric synapse | 96 | 118 | 0.006352 | 0.754979 |
| BP | GO:0006909 | phagocytosis | 92 | 112 | 0.006567 | 0.762421 |
| BP | GO:0031639 | plasminogen activation | 15 | 15 | 0.006576 | 0.762421 |
| BP | GO:0046475 | glycerophospholipid catabolic process | 15 | 15 | 0.006576 | 0.762421 |
| MF | GO:0005245 | voltage-gated calcium channel activity | 26 | 28 | 0.006587 | 0.762421 |
| MF | GO:0008514 | organic anion transmembrane transporter activity | 103 | 126 | 0.006700 | 0.770486 |
| MF | GO:0003887 | DNA-directed DNA polymerase activity | 15 | 15 | 0.006961 | 0.786445 |
| MF | GO:0004970 | ionotropic glutamate receptor activity | 15 | 15 | 0.006961 | 0.786445 |
| CC | GO:0031526 | brush border membrane | 20 | 21 | 0.007016 | 0.786445 |
| CC | GO:0044447 | axoneme part | 20 | 21 | 0.007016 | 0.786445 |
| BP | GO:0032481 | positive regulation of type I interferon production | 34 | 38 | 0.007409 | 0.825252 |
| MF | GO:0008238 | exopeptidase activity | 68 | 81 | 0.007926 | 0.877334 |
| BP | GO:2000181 | negative regulation of blood vessel morphogenesis | 60 | 71 | 0.008140 | 0.885500 |
| MF | GO:0004812 | aminoacyl-tRNA ligase activity | 34 | 38 | 0.008150 | 0.885500 |
| MF | GO:0016875 | ligase activity, forming carbon-oxygen bonds | 34 | 38 | 0.008150 | 0.885500 |
| MF | GO:0005261 | cation channel activity | 180 | 228 | 0.008320 | 0.895255 |
| MF | GO:0015297 | antiporter activity | 38 | 43 | 0.008341 | 0.895255 |
| CC | GO:0034451 | centriolar satellite | 68 | 82 | 0.008805 | 0.939429 |
| BP | GO:0006623 | protein targeting to vacuole | 14 | 14 | 0.009194 | 0.969431 |
| MF | GO:0005342 | organic acid transmembrane transporter activity | 74 | 89 | 0.009247 | 0.969431 |
| BP | GO:0006418 | tRNA aminoacylation for protein translation | 33 | 37 | 0.009360 | 0.969431 |
| BP | GO:0051965 | positive regulation of synapse assembly | 33 | 37 | 0.009360 | 0.969431 |
| CC | GO:0016459 | myosin complex | 36 | 41 | 0.009360 | 0.969431 |
| MF | GO:0008233 | peptidase activity | 395 | 517 | 0.009416 | 0.969534 |
| MF | GO:0140096 | catalytic activity, acting on a protein | 1190 | 1602 | 0.009648 | 0.987622 |
| MF | GO:0016829 | lyase activity | 110 | 136 | 0.009772 | 0.994616 |
| MF | GO:0015318 | inorganic molecular entity transmembrane transporter activity | 445 | 585 | 0.010092 | 1.000000 |
| MF | GO:0016298 | lipase activity | 70 | 84 | 0.010123 | 1.000000 |
| MF | GO:0070011 | peptidase activity, acting on L-amino acid peptides | 388 | 508 | 0.010350 | 1.000000 |
| MF | GO:0005096 | GTPase activator activity | 106 | 131 | 0.010837 | 1.000000 |
| BP | GO:0071158 | positive regulation of cell cycle arrest | 19 | 20 | 0.011030 | 1.000000 |
| MF | GO:0019899 | enzyme binding | 985 | 1322 | 0.011076 | 1.000000 |
| MF | GO:0022832 | voltage-gated channel activity | 109 | 135 | 0.011138 | 1.000000 |
| MF | GO:0008376 | acetylgalactosaminyltransferase activity | 24 | 26 | 0.011155 | 1.000000 |
| MF | GO:0042169 | SH2 domain binding | 24 | 26 | 0.011155 | 1.000000 |
| BP | GO:0009395 | phospholipid catabolic process | 28 | 31 | 0.011423 | 1.000000 |
| MF | GO:0016740 | transferase activity | 1226 | 1653 | 0.011578 | 1.000000 |
| BP | GO:0016525 | negative regulation of angiogenesis | 58 | 69 | 0.011606 | 1.000000 |
| BP | GO:0015696 | ammonium transport | 36 | 41 | 0.011712 | 1.000000 |
| BP | GO:0043038 | amino acid activation | 36 | 41 | 0.011712 | 1.000000 |
| MF | GO:0030215 | semaphorin receptor binding | 19 | 20 | 0.011759 | 1.000000 |
| MF | GO:0005044 | scavenger receptor activity | 28 | 31 | 0.012390 | 1.000000 |
| MF | GO:0046943 | carboxylic acid transmembrane transporter activity | 72 | 87 | 0.012673 | 1.000000 |
| MF | GO:0005244 | voltage-gated ion channel activity | 108 | 134 | 0.012675 | 1.000000 |
| BP | GO:0043473 | pigmentation | 54 | 64 | 0.012677 | 1.000000 |
| CC | GO:0034706 | sodium channel complex | 18 | 19 | 0.012775 | 1.000000 |
| BP | GO:0043173 | nucleotide salvage | 13 | 13 | 0.012855 | 1.000000 |
| BP | GO:0072010 | glomerular epithelium development | 13 | 13 | 0.012855 | 1.000000 |
| CC | GO:0005930 | axoneme | 59 | 71 | 0.013181 | 1.000000 |
| CC | GO:0031256 | leading edge membrane | 59 | 71 | 0.013181 | 1.000000 |
| CC | GO:0097014 | ciliary plasm | 59 | 71 | 0.013181 | 1.000000 |
| BP | GO:0006195 | purine nucleotide catabolic process | 23 | 25 | 0.013474 | 1.000000 |
| BP | GO:0006281 | DNA repair | 249 | 323 | 0.013499 | 1.000000 |
| MF | GO:0004198 | calcium-dependent cysteine-type endopeptidase activity | 13 | 13 | 0.013505 | 1.000000 |
| MF | GO:0030506 | ankyrin binding | 13 | 13 | 0.013505 | 1.000000 |
| MF | GO:0042805 | actinin binding | 13 | 13 | 0.013505 | 1.000000 |
| MF | GO:0003678 | DNA helicase activity | 47 | 55 | 0.013781 | 1.000000 |
| MF | GO:0022838 | substrate-specific channel activity | 249 | 322 | 0.013915 | 1.000000 |
| BP | GO:0006820 | anion transport | 211 | 272 | 0.013934 | 1.000000 |
| MF | GO:0004520 | endodeoxyribonuclease activity | 23 | 25 | 0.014464 | 1.000000 |
| BP | GO:0043039 | tRNA aminoacylation | 35 | 40 | 0.014544 | 1.000000 |
| BP | GO:0043030 | regulation of macrophage activation | 27 | 30 | 0.014570 | 1.000000 |
| BP | GO:0070129 | regulation of mitochondrial translation | 18 | 19 | 0.014738 | 1.000000 |
| MF | GO:0031267 | small GTPase binding | 141 | 178 | 0.014880 | 1.000000 |
| MF | GO:0005248 | voltage-gated sodium channel activity | 18 | 19 | 0.015654 | 1.000000 |
| MF | GO:0016405 | CoA-ligase activity | 18 | 19 | 0.015654 | 1.000000 |
| CC | GO:0015629 | actin cytoskeleton | 226 | 295 | 0.015818 | 1.000000 |
| MF | GO:0004536 | deoxyribonuclease activity | 31 | 35 | 0.016120 | 1.000000 |
| BP | GO:0060249 | anatomical structure homeostasis | 186 | 239 | 0.016222 | 1.000000 |
| MF | GO:0016835 | carbon-oxygen lyase activity | 46 | 54 | 0.016610 | 1.000000 |
| MF | GO:0004497 | monooxygenase activity | 53 | 63 | 0.016989 | 1.000000 |
| BP | GO:0009166 | nucleotide catabolic process | 79 | 97 | 0.017017 | 1.000000 |
| BP | GO:0001578 | microtubule bundle formation | 59 | 71 | 0.017550 | 1.000000 |
| BP | GO:0120036 | plasma membrane bounded cell projection organization | 619 | 828 | 0.017714 | 1.000000 |
| CC | GO:0031253 | cell projection membrane | 110 | 139 | 0.017775 | 1.000000 |
| BP | GO:0002385 | mucosal immune response | 12 | 12 | 0.017973 | 1.000000 |
| BP | GO:0072413 | signal transduction involved in mitotic cell cycle checkpoint | 12 | 12 | 0.017973 | 1.000000 |
| BP | GO:0072673 | lamellipodium morphogenesis | 12 | 12 | 0.017973 | 1.000000 |
| BP | GO:1905515 | non-motile cilium assembly | 34 | 39 | 0.018009 | 1.000000 |
| BP | GO:0021675 | nerve development | 45 | 53 | 0.018023 | 1.000000 |
| MF | GO:0005543 | phospholipid binding | 199 | 256 | 0.018267 | 1.000000 |
| BP | GO:0050766 | positive regulation of phagocytosis | 26 | 29 | 0.018531 | 1.000000 |
| BP | GO:0021545 | cranial nerve development | 30 | 34 | 0.018564 | 1.000000 |
| MF | GO:0017154 | semaphorin receptor activity | 12 | 12 | 0.018810 | 1.000000 |
| MF | GO:0042974 | retinoic acid receptor binding | 12 | 12 | 0.018810 | 1.000000 |
| BP | GO:0050764 | regulation of phagocytosis | 41 | 48 | 0.019508 | 1.000000 |
| BP | GO:0007163 | establishment or maintenance of cell polarity | 97 | 121 | 0.019586 | 1.000000 |
| BP | GO:0045616 | regulation of keratinocyte differentiation | 17 | 18 | 0.019650 | 1.000000 |
| BP | GO:0046461 | neutral lipid catabolic process | 17 | 18 | 0.019650 | 1.000000 |
| BP | GO:0046464 | acylglycerol catabolic process | 17 | 18 | 0.019650 | 1.000000 |
| BP | GO:1903319 | positive regulation of protein maturation | 17 | 18 | 0.019650 | 1.000000 |
| MF | GO:0015085 | calcium ion transmembrane transporter activity | 69 | 84 | 0.020029 | 1.000000 |
| MF | GO:0030374 | nuclear receptor transcription coactivator activity | 30 | 34 | 0.020121 | 1.000000 |
| CC | GO:0098984 | neuron to neuron synapse | 106 | 134 | 0.020160 | 1.000000 |
| MF | GO:0005217 | intracellular ligand-gated ion channel activity | 17 | 18 | 0.020794 | 1.000000 |
| MF | GO:0015103 | inorganic anion transmembrane transporter activity | 85 | 105 | 0.020907 | 1.000000 |
| MF | GO:0017111 | nucleoside-triphosphatase activity | 489 | 649 | 0.021353 | 1.000000 |
| MF | GO:0015294 | solute:cation symporter activity | 41 | 48 | 0.021478 | 1.000000 |
| CC | GO:0030054 | cell junction | 348 | 463 | 0.021656 | 1.000000 |
| MF | GO:0016462 | pyrophosphatase activity | 519 | 690 | 0.021854 | 1.000000 |
| MF | GO:0016817 | hydrolase activity, acting on acid anhydrides | 519 | 690 | 0.021854 | 1.000000 |
| MF | GO:0016818 | hydrolase activity, acting on acid anhydrides, in phosphorus-containing anhydrides | 519 | 690 | 0.021854 | 1.000000 |
| CC | GO:0016605 | PML body | 46 | 55 | 0.022298 | 1.000000 |
| MF | GO:0052689 | carboxylic ester hydrolase activity | 78 | 96 | 0.022550 | 1.000000 |
| BP | GO:0009154 | purine ribonucleotide catabolic process | 21 | 23 | 0.022635 | 1.000000 |
| BP | GO:0046460 | neutral lipid biosynthetic process | 21 | 23 | 0.022635 | 1.000000 |
| BP | GO:0046463 | acylglycerol biosynthetic process | 21 | 23 | 0.022635 | 1.000000 |
| CC | GO:0005858 | axonemal dynein complex | 11 | 11 | 0.022764 | 1.000000 |
| BP | GO:0007626 | locomotory behavior | 108 | 136 | 0.023140 | 1.000000 |
| BP | GO:0072665 | protein localization to vacuole | 25 | 28 | 0.023497 | 1.000000 |
| CC | GO:0005813 | centrosome | 306 | 406 | 0.024056 | 1.000000 |
| MF | GO:0008324 | cation transmembrane transporter activity | 339 | 446 | 0.024338 | 1.000000 |
| MF | GO:0004518 | nuclease activity | 112 | 141 | 0.024467 | 1.000000 |
| MF | GO:0005539 | glycosaminoglycan binding | 87 | 108 | 0.024479 | 1.000000 |
| BP | GO:0035264 | multicellular organism growth | 89 | 111 | 0.024570 | 1.000000 |
| CC | GO:0044448 | cell cortex part | 68 | 84 | 0.024573 | 1.000000 |
| MF | GO:0015267 | channel activity | 265 | 346 | 0.024715 | 1.000000 |
| MF | GO:0022803 | passive transmembrane transporter activity | 265 | 346 | 0.024715 | 1.000000 |
| MF | GO:0019838 | growth factor binding | 74 | 91 | 0.025076 | 1.000000 |
| MF | GO:0043492 | ATPase activity, coupled to movement of substances | 74 | 91 | 0.025076 | 1.000000 |
| BP | GO:0006622 | protein targeting to lysosome | 11 | 11 | 0.025129 | 1.000000 |
| BP | GO:0006837 | serotonin transport | 11 | 11 | 0.025129 | 1.000000 |
| BP | GO:0031571 | mitotic G1 DNA damage checkpoint | 11 | 11 | 0.025129 | 1.000000 |
| BP | GO:0042730 | fibrinolysis | 11 | 11 | 0.025129 | 1.000000 |
| BP | GO:0044819 | mitotic G1/S transition checkpoint | 11 | 11 | 0.025129 | 1.000000 |
| BP | GO:0051386 | regulation of neurotrophin TRK receptor signaling pathway | 11 | 11 | 0.025129 | 1.000000 |
| BP | GO:0061318 | renal filtration cell differentiation | 11 | 11 | 0.025129 | 1.000000 |
| BP | GO:0072112 | glomerular visceral epithelial cell differentiation | 11 | 11 | 0.025129 | 1.000000 |
| BP | GO:0072311 | glomerular epithelial cell differentiation | 11 | 11 | 0.025129 | 1.000000 |
| BP | GO:1902884 | positive regulation of response to oxidative stress | 11 | 11 | 0.025129 | 1.000000 |
| MF | GO:0004175 | endopeptidase activity | 253 | 330 | 0.025773 | 1.000000 |
| BP | GO:0010954 | positive regulation of protein processing | 16 | 17 | 0.026136 | 1.000000 |
| MF | GO:0016712 | oxidoreductase activity, acting on paired donors, with incorporation or reduction of molecular oxygen, reduced flavin or flavoprotein as one donor, and incorporation of one atom of oxygen | 11 | 11 | 0.026198 | 1.000000 |
| MF | GO:0016894 | endonuclease activity, active with either ribo- or deoxyribonucleic acids and producing 3'-phosphomonoesters | 11 | 11 | 0.026198 | 1.000000 |
| MF | GO:0030169 | low-density lipoprotein particle binding | 11 | 11 | 0.026198 | 1.000000 |
| CC | GO:0045177 | apical part of cell | 167 | 217 | 0.026632 | 1.000000 |
| MF | GO:0070273 | phosphatidylinositol-4-phosphate binding | 16 | 17 | 0.027554 | 1.000000 |
| MF | GO:0004519 | endonuclease activity | 70 | 86 | 0.027899 | 1.000000 |
| CC | GO:0030864 | cortical actin cytoskeleton | 31 | 36 | 0.027994 | 1.000000 |
| BP | GO:0007264 | small GTPase mediated signal transduction | 206 | 268 | 0.028092 | 1.000000 |
| MF | GO:0030594 | neurotransmitter receptor activity | 60 | 73 | 0.028606 | 1.000000 |
| MF | GO:0015399 | primary active transmembrane transporter activity | 73 | 90 | 0.028811 | 1.000000 |
| MF | GO:0015405 | P-P-bond-hydrolysis-driven transmembrane transporter activity | 73 | 90 | 0.028811 | 1.000000 |
| BP | GO:0015844 | monoamine transport | 28 | 32 | 0.028866 | 1.000000 |
| BP | GO:0002269 | leukocyte activation involved in inflammatory response | 20 | 22 | 0.029204 | 1.000000 |
| BP | GO:0032814 | regulation of natural killer cell activation | 20 | 22 | 0.029204 | 1.000000 |
| BP | GO:0061462 | protein localization to lysosome | 20 | 22 | 0.029204 | 1.000000 |
| CC | GO:0016323 | basolateral plasma membrane | 97 | 123 | 0.029364 | 1.000000 |
| BP | GO:0016242 | negative regulation of macroautophagy | 24 | 27 | 0.029697 | 1.000000 |
| BP | GO:0000226 | microtubule cytoskeleton organization | 287 | 378 | 0.030031 | 1.000000 |
| CC | GO:0045095 | keratin filament | 27 | 31 | 0.030337 | 1.000000 |
| BP | GO:0051606 | detection of stimulus | 78 | 97 | 0.030393 | 1.000000 |
| BP | GO:0043062 | extracellular structure organization | 153 | 197 | 0.030823 | 1.000000 |
| BP | GO:1901292 | nucleoside phosphate catabolic process | 81 | 101 | 0.030897 | 1.000000 |
| MF | GO:0004181 | metallocarboxypeptidase activity | 20 | 22 | 0.031004 | 1.000000 |
| MF | GO:0016878 | acid-thiol ligase activity | 20 | 22 | 0.031004 | 1.000000 |
| MF | GO:0004180 | carboxypeptidase activity | 28 | 32 | 0.031060 | 1.000000 |
| MF | GO:0015370 | solute:sodium symporter activity | 28 | 32 | 0.031060 | 1.000000 |
| MF | GO:0015298 | solute:cation antiporter activity | 24 | 27 | 0.031742 | 1.000000 |
| CC | GO:0005614 | interstitial matrix | 10 | 10 | 0.032111 | 1.000000 |
| CC | GO:0042405 | nuclear inclusion body | 10 | 10 | 0.032111 | 1.000000 |
| CC | GO:0016324 | apical plasma membrane | 131 | 169 | 0.032307 | 1.000000 |
| BP | GO:0034404 | nucleobase-containing small molecule biosynthetic process | 96 | 121 | 0.032468 | 1.000000 |
| CC | GO:1990752 | microtubule end | 19 | 21 | 0.032855 | 1.000000 |
| MF | GO:0042626 | ATPase activity, coupled to transmembrane movement of substances | 72 | 89 | 0.033033 | 1.000000 |
| MF | GO:0005516 | calmodulin binding | 59 | 72 | 0.033243 | 1.000000 |
| BP | GO:0030030 | cell projection organization | 628 | 845 | 0.033847 | 1.000000 |
| MF | GO:0015171 | amino acid transmembrane transporter activity | 42 | 50 | 0.034122 | 1.000000 |
| BP | GO:0015748 | organophosphate ester transport | 38 | 45 | 0.034309 | 1.000000 |
| BP | GO:1903317 | regulation of protein maturation | 38 | 45 | 0.034309 | 1.000000 |
| MF | GO:0030695 | GTPase regulator activity | 115 | 146 | 0.034580 | 1.000000 |
| BP | GO:0006638 | neutral lipid metabolic process | 58 | 71 | 0.034587 | 1.000000 |
| MF | GO:0022843 | voltage-gated cation channel activity | 78 | 97 | 0.034611 | 1.000000 |
| BP | GO:0032727 | positive regulation of interferon-alpha production | 15 | 16 | 0.034671 | 1.000000 |
| BP | GO:0032878 | regulation of establishment or maintenance of cell polarity | 15 | 16 | 0.034671 | 1.000000 |
| BP | GO:0042044 | fluid transport | 15 | 16 | 0.034671 | 1.000000 |
| BP | GO:0046058 | cAMP metabolic process | 15 | 16 | 0.034671 | 1.000000 |
| BP | GO:0046473 | phosphatidic acid metabolic process | 15 | 16 | 0.034671 | 1.000000 |
| MF | GO:0042578 | phosphoric ester hydrolase activity | 206 | 268 | 0.035106 | 1.000000 |
| BP | GO:0002323 | natural killer cell activation involved in immune response | 10 | 10 | 0.035131 | 1.000000 |
| BP | GO:0006474 | N-terminal protein amino acid acetylation | 10 | 10 | 0.035131 | 1.000000 |
| BP | GO:0016045 | detection of bacterium | 10 | 10 | 0.035131 | 1.000000 |
| BP | GO:0019377 | glycolipid catabolic process | 10 | 10 | 0.035131 | 1.000000 |
| BP | GO:0019985 | translesion synthesis | 10 | 10 | 0.035131 | 1.000000 |
| BP | GO:0032823 | regulation of natural killer cell differentiation | 10 | 10 | 0.035131 | 1.000000 |
| BP | GO:0045176 | apical protein localization | 10 | 10 | 0.035131 | 1.000000 |
| BP | GO:0046485 | ether lipid metabolic process | 10 | 10 | 0.035131 | 1.000000 |
| BP | GO:0095500 | acetylcholine receptor signaling pathway | 10 | 10 | 0.035131 | 1.000000 |
| BP | GO:0098543 | detection of other organism | 10 | 10 | 0.035131 | 1.000000 |
| BP | GO:1900409 | positive regulation of cellular response to oxidative stress | 10 | 10 | 0.035131 | 1.000000 |
| BP | GO:1900424 | regulation of defense response to bacterium | 10 | 10 | 0.035131 | 1.000000 |
| BP | GO:1902402 | signal transduction involved in mitotic DNA damage checkpoint | 10 | 10 | 0.035131 | 1.000000 |
| BP | GO:1902403 | signal transduction involved in mitotic DNA integrity checkpoint | 10 | 10 | 0.035131 | 1.000000 |
| BP | GO:1903831 | signal transduction involved in cellular response to ammonium ion | 10 | 10 | 0.035131 | 1.000000 |
| BP | GO:1903978 | regulation of microglial cell activation | 10 | 10 | 0.035131 | 1.000000 |
| BP | GO:1905145 | cellular response to acetylcholine | 10 | 10 | 0.035131 | 1.000000 |
| BP | GO:2001224 | positive regulation of neuron migration | 10 | 10 | 0.035131 | 1.000000 |
| BP | GO:0061028 | establishment of endothelial barrier | 27 | 31 | 0.035816 | 1.000000 |
| MF | GO:0099516 | ion antiporter activity | 31 | 36 | 0.036226 | 1.000000 |
| CC | GO:0016604 | nuclear body | 404 | 543 | 0.036296 | 1.000000 |
| MF | GO:0016799 | hydrolase activity, hydrolyzing N-glycosyl compounds | 15 | 16 | 0.036416 | 1.000000 |
| MF | GO:0043531 | ADP binding | 15 | 16 | 0.036416 | 1.000000 |
| MF | GO:0005283 | amino acid:sodium symporter activity | 10 | 10 | 0.036487 | 1.000000 |
| MF | GO:0008271 | secondary active sulfate transmembrane transporter activity | 10 | 10 | 0.036487 | 1.000000 |
| MF | GO:0015116 | sulfate transmembrane transporter activity | 10 | 10 | 0.036487 | 1.000000 |
| MF | GO:0050308 | sugar-phosphatase activity | 10 | 10 | 0.036487 | 1.000000 |
| BP | GO:1901343 | negative regulation of vasculature development | 64 | 79 | 0.036734 | 1.000000 |
| MF | GO:0005262 | calcium channel activity | 55 | 67 | 0.036981 | 1.000000 |
| CC | GO:0044437 | vacuolar part | 121 | 156 | 0.037484 | 1.000000 |
| BP | GO:0045981 | positive regulation of nucleotide metabolic process | 19 | 21 | 0.037553 | 1.000000 |
| BP | GO:1900544 | positive regulation of purine nucleotide metabolic process | 19 | 21 | 0.037553 | 1.000000 |
| BP | GO:0030837 | negative regulation of actin filament polymerization | 34 | 40 | 0.037647 | 1.000000 |
| BP | GO:0032456 | endocytic recycling | 34 | 40 | 0.037647 | 1.000000 |
| CC | GO:0030119 | AP-type membrane coat adaptor complex | 26 | 30 | 0.037824 | 1.000000 |
| MF | GO:0016877 | ligase activity, forming carbon-sulfur bonds | 27 | 31 | 0.038398 | 1.000000 |
| MF | GO:0004222 | metalloendopeptidase activity | 74 | 92 | 0.038626 | 1.000000 |
| BP | GO:0030029 | actin filament-based process | 344 | 457 | 0.038956 | 1.000000 |
| BP | GO:0030010 | establishment of cell polarity | 73 | 91 | 0.038970 | 1.000000 |
| CC | GO:0031252 | cell leading edge | 174 | 228 | 0.039074 | 1.000000 |
| MF | GO:0016798 | hydrolase activity, acting on glycosyl bonds | 77 | 96 | 0.039359 | 1.000000 |
| BP | GO:0071897 | DNA biosynthetic process | 76 | 95 | 0.039501 | 1.000000 |
| BP | GO:0007596 | blood coagulation | 79 | 99 | 0.039942 | 1.000000 |
| BP | GO:0006639 | acylglycerol metabolic process | 57 | 70 | 0.040138 | 1.000000 |
| MF | GO:0008235 | metalloexopeptidase activity | 41 | 49 | 0.040558 | 1.000000 |
| CC | GO:0009986 | cell surface | 293 | 391 | 0.041069 | 1.000000 |
| BP | GO:0030042 | actin filament depolymerization | 37 | 44 | 0.041157 | 1.000000 |
| BP | GO:0070613 | regulation of protein processing | 37 | 44 | 0.041157 | 1.000000 |
| CC | GO:0098644 | complex of collagen trimers | 14 | 15 | 0.041163 | 1.000000 |
| BP | GO:0051345 | positive regulation of hydrolase activity | 285 | 377 | 0.041913 | 1.000000 |
| BP | GO:1901136 | carbohydrate derivative catabolic process | 63 | 78 | 0.042278 | 1.000000 |
| CC | GO:0044309 | neuron spine | 39 | 47 | 0.043122 | 1.000000 |
| BP | GO:0030198 | extracellular matrix organization | 129 | 166 | 0.043587 | 1.000000 |
| BP | GO:0030036 | actin cytoskeleton organization | 312 | 414 | 0.043599 | 1.000000 |
| BP | GO:0007098 | centrosome cycle | 69 | 86 | 0.043824 | 1.000000 |
| BP | GO:0098657 | import into cell | 287 | 380 | 0.044132 | 1.000000 |
| BP | GO:0002253 | activation of immune response | 189 | 247 | 0.044795 | 1.000000 |
| BP | GO:0007599 | hemostasis | 81 | 102 | 0.045572 | 1.000000 |
| BP | GO:0002755 | MyD88-dependent toll-like receptor signaling pathway | 14 | 15 | 0.045856 | 1.000000 |
| BP | GO:0030810 | positive regulation of nucleotide biosynthetic process | 14 | 15 | 0.045856 | 1.000000 |
| BP | GO:0048240 | sperm capacitation | 14 | 15 | 0.045856 | 1.000000 |
| BP | GO:0048384 | retinoic acid receptor signaling pathway | 14 | 15 | 0.045856 | 1.000000 |
| BP | GO:0048679 | regulation of axon regeneration | 14 | 15 | 0.045856 | 1.000000 |
| BP | GO:0051194 | positive regulation of cofactor metabolic process | 14 | 15 | 0.045856 | 1.000000 |
| BP | GO:0071526 | semaphorin-plexin signaling pathway | 14 | 15 | 0.045856 | 1.000000 |
| BP | GO:0150077 | regulation of neuroinflammatory response | 14 | 15 | 0.045856 | 1.000000 |
| BP | GO:1900373 | positive regulation of purine nucleotide biosynthetic process | 14 | 15 | 0.045856 | 1.000000 |
| BP | GO:2000765 | regulation of cytoplasmic translation | 14 | 15 | 0.045856 | 1.000000 |
| MF | GO:0004620 | phospholipase activity | 60 | 74 | 0.045959 | 1.000000 |
| BP | GO:0009135 | purine nucleoside diphosphate metabolic process | 56 | 69 | 0.046459 | 1.000000 |
| BP | GO:0009179 | purine ribonucleoside diphosphate metabolic process | 56 | 69 | 0.046459 | 1.000000 |
| BP | GO:0009185 | ribonucleoside diphosphate metabolic process | 56 | 69 | 0.046459 | 1.000000 |
| BP | GO:0032204 | regulation of telomere maintenance | 43 | 52 | 0.046736 | 1.000000 |
| BP | GO:0007099 | centriole replication | 22 | 25 | 0.046931 | 1.000000 |
| BP | GO:0016073 | snRNA metabolic process | 22 | 25 | 0.046931 | 1.000000 |
| BP | GO:0042119 | neutrophil activation | 22 | 25 | 0.046931 | 1.000000 |
| BP | GO:0071242 | cellular response to ammonium ion | 22 | 25 | 0.046931 | 1.000000 |
| MF | GO:0035091 | phosphatidylinositol binding | 121 | 155 | 0.047093 | 1.000000 |
| MF | GO:0008028 | monocarboxylic acid transmembrane transporter activity | 26 | 30 | 0.047301 | 1.000000 |
| MF | GO:0030246 | carbohydrate binding | 115 | 147 | 0.047344 | 1.000000 |
| BP | GO:0006928 | movement of cell or subcellular component | 888 | 1206 | 0.047575 | 1.000000 |
| BP | GO:0051188 | cofactor biosynthetic process | 131 | 169 | 0.047624 | 1.000000 |
| MF | GO:0004653 | polypeptide N-acetylgalactosaminyltransferase activity | 14 | 15 | 0.047986 | 1.000000 |
| BP | GO:0000731 | DNA synthesis involved in DNA repair | 18 | 20 | 0.048114 | 1.000000 |
| BP | GO:0021602 | cranial nerve morphogenesis | 18 | 20 | 0.048114 | 1.000000 |
| BP | GO:0038084 | vascular endothelial growth factor signaling pathway | 18 | 20 | 0.048114 | 1.000000 |
| BP | GO:0002566 | somatic diversification of immune receptors via somatic mutation | 9 | 9 | 0.049112 | 1.000000 |
| BP | GO:0006188 | IMP biosynthetic process | 9 | 9 | 0.049112 | 1.000000 |
| BP | GO:0006896 | Golgi to vacuole transport | 9 | 9 | 0.049112 | 1.000000 |
| BP | GO:0010457 | centriole-centriole cohesion | 9 | 9 | 0.049112 | 1.000000 |
| BP | GO:0010755 | regulation of plasminogen activation | 9 | 9 | 0.049112 | 1.000000 |
| BP | GO:0017000 | antibiotic biosynthetic process | 9 | 9 | 0.049112 | 1.000000 |
| BP | GO:0031573 | intra-S DNA damage checkpoint | 9 | 9 | 0.049112 | 1.000000 |
| BP | GO:0034134 | toll-like receptor 2 signaling pathway | 9 | 9 | 0.049112 | 1.000000 |
| BP | GO:0046479 | glycosphingolipid catabolic process | 9 | 9 | 0.049112 | 1.000000 |
| BP | GO:0048681 | negative regulation of axon regeneration | 9 | 9 | 0.049112 | 1.000000 |
| BP | GO:0060856 | establishment of blood-brain barrier | 9 | 9 | 0.049112 | 1.000000 |
| BP | GO:0090160 | Golgi to lysosome transport | 9 | 9 | 0.049112 | 1.000000 |
| BP | GO:2000310 | regulation of NMDA receptor activity | 9 | 9 | 0.049112 | 1.000000 |
| CC | GO:0030136 | clathrin-coated vesicle | 54 | 67 | 0.049230 | 1.000000 |
| CC | GO:0098794 | postsynapse | 215 | 285 | 0.049304 | 1.000000 |
| CC | GO:0097458 | neuron part | 550 | 747 | 0.049315 | 1.000000 |
| BP | GO:0098609 | cell-cell adhesion | 321 | 427 | 0.049420 | 1.000000 |
| CC | GO:0099572 | postsynaptic specialization | 104 | 134 | 0.049927 | 1.000000 |
| BP | GO:0016052 | carbohydrate catabolic process | 68 | 85 | 0.049979 | 1.000000 |

**Table S19 The gene annotation of the top 1% in XP-CLR analysis of Tibetan sheep and Argali (partial results)**

| **Chr** | **Bin_Start** | **Bin_End** | **xpclr_norm** | **Chr** | **Gene_Start** | **Gene_End** | **GeneID** |
| --- | --- | --- | --- | --- | --- | --- | --- |
| NC_056080.1 | 56180001 | 56185000 | 16.9511 | NC_056080.1 | 56178694 | 56182653 | gene-TIMM17B |
| NC_056078.1 | 25555001 | 25560000 | 15.7779 | NC_056078.1 | 25530068 | 25569595 | gene-AIFM2 |
| NC_056060.1 | 35275001 | 35280000 | 13.2968 | NC_056060.1 | 35232770 | 35383214 | gene-MGA |
| NC_056074.1 | 22430001 | 22435000 | 13.1118 | NC_056074.1 | 22345557 | 23145541 | gene-NAV2 |
| NC_056057.1 | 11650001 | 11655000 | 12.7414 | NC_056057.1 | 11558208 | 11671605 | gene-CALCR |
| NC_056060.1 | 35285001 | 35290000 | 12.7133 | NC_056060.1 | 35232770 | 35383214 | gene-MGA |
| NC_056057.1 | 11645001 | 11650000 | 11.9334 | NC_056057.1 | 11558208 | 11671605 | gene-CALCR |
| NC_056077.1 | 3585001 | 3590000 | 11.4440 | NC_056077.1 | 3563672 | 3613563 | gene-TRAP1 |
| NC_056059.1 | 91370001 | 91375000 | 11.2791 | NC_056059.1 | 91316458 | 91393341 | gene-USO1 |
| NC_056076.1 | 2555001 | 2560000 | 11.2003 | NC_056076.1 | 2529713 | 2598591 | gene-ZNF236 |
| NC_056076.1 | 14260001 | 14265000 | 11.1854 | NC_056076.1 | 14132832 | 14293753 | gene-PIK3C3 |
| NC_056073.1 | 50940001 | 50945000 | 11.0937 | NC_056073.1 | 50943220 | 50990769 | gene-DUSP22 |
| NC_056072.1 | 2755001 | 2760000 | 11.0896 | NC_056072.1 | 2688539 | 2768692 | gene-CMC1 |
| NC_056072.1 | 2755001 | 2760000 | 11.0896 | NC_056072.1 | 2688642 | 2768521 | gene-LOC114108644 |
| NC_056060.1 | 90305001 | 90310000 | 11.0480 | NC_056060.1 | 90309511 | 90309582 | gene-TRNAS-GGA-140 |
| NC_056060.1 | 90305001 | 90310000 | 11.0480 | NC_056060.1 | 89961361 | 90416423 | gene-CEP128 |
| NC_056062.1 | 20090001 | 20095000 | 10.9957 | NC_056062.1 | 20093826 | 20132511 | gene-LOC114116349 |
| NC_056065.1 | 75290001 | 75295000 | 10.9092 | NC_056065.1 | 75275636 | 75316341 | gene-LOC105611355 |
| NC_056074.1 | 22440001 | 22445000 | 10.6548 | NC_056074.1 | 22345557 | 23145541 | gene-NAV2 |
| NC_056060.1 | 35280001 | 35285000 | 10.6117 | NC_056060.1 | 35232770 | 35383214 | gene-MGA |
| NC_056058.1 | 79055001 | 79060000 | 10.5016 | NC_056058.1 | 78971121 | 79279487 | gene-SSBP2 |
| NC_056067.1 | 15175001 | 15180000 | 10.5015 | NC_056067.1 | 15159937 | 15193860 | gene-LOC105613342 |
| NC_056067.1 | 15175001 | 15180000 | 10.5015 | NC_056067.1 | 15123291 | 15419588 | gene-ITFG1 |
| NC_056068.1 | 82235001 | 82240000 | 10.4377 | NC_056068.1 | 82221201 | 82259659 | gene-GLB1L2 |
| NC_056072.1 | 50135001 | 50140000 | 10.3829 | NC_056072.1 | 50138182 | 50149232 | gene-SEMA3B |
| NC_056073.1 | 50635001 | 50640000 | 10.2902 | NC_056073.1 | 50261968 | 50681552 | gene-GMDS |
| NC_056057.1 | 116205001 | 116210000 | 10.2019 | NC_056057.1 | 116204693 | 116462140 | gene-KMT2C |
| NC_056059.1 | 91360001 | 91365000 | 10.1033 | NC_056059.1 | 91316458 | 91393341 | gene-USO1 |
| NC_056054.1 | 69625001 | 69630000 | 10.0712 | NC_056054.1 | 69551721 | 69666728 | gene-RPAP2 |
| NC_056054.1 | 69625001 | 69630000 | 10.0712 | NC_056054.1 | 69551721 | 69666728 | gene-RPAP2 |
| NC_056067.1 | 15185001 | 15190000 | 9.8912 | NC_056067.1 | 15159937 | 15193860 | gene-LOC105613342 |
| NC_056067.1 | 15185001 | 15190000 | 9.8912 | NC_056067.1 | 15123291 | 15419588 | gene-ITFG1 |
| NC_056059.1 | 85325001 | 85330000 | 9.8816 | NC_056059.1 | 85314490 | 85332612 | gene-LOC101117163 |
| NC_056064.1 | 25030001 | 25035000 | 9.7129 | NC_056064.1 | 25026948 | 25039127 | gene-XAF1 |
| NC_056056.1 | 680001 | 685000 | 9.6708 | NC_056056.1 | 681290 | 683586 | gene-TOR4A |
| NC_056061.1 | 29980001 | 29985000 | 9.6603 | NC_056061.1 | 29911774 | 30192303 | gene-PDSS2 |
| NC_056068.1 | 52955001 | 52960000 | 9.6481 | NC_056068.1 | 52943071 | 52969899 | gene-SPCS2 |
| NC_056080.1 | 115760001 | 115765000 | 9.5934 | NC_056080.1 | 115750955 | 115768108 | gene-LOC105605783 |
| NC_056055.1 | 138910001 | 138915000 | 9.5776 | NC_056055.1 | 138895758 | 138966304 | gene-SP5 |
| NC_056054.1 | 193895001 | 193900000 | 9.5695 | NC_056054.1 | 193873135 | 193953226 | gene-ATP13A3 |
| NC_056054.1 | 193895001 | 193900000 | 9.5695 | NC_056054.1 | 193873135 | 193953226 | gene-ATP13A3 |
| NC_056066.1 | 51160001 | 51165000 | 9.5611 | NC_056066.1 | 51152602 | 51165689 | gene-C13H20orf27 |
| NC_056074.1 | 22445001 | 22450000 | 9.5088 | NC_056074.1 | 22345557 | 23145541 | gene-NAV2 |
| NC_056071.1 | 43670001 | 43675000 | 9.4250 | NC_056071.1 | 43525705 | 43726747 | gene-RALGAPA1 |
| NC_056062.1 | 70795001 | 70800000 | 9.3915 | NC_056062.1 | 70785895 | 70797501 | gene-ABRA |
| NC_056062.1 | 70795001 | 70800000 | 9.3915 | NC_056062.1 | 70744084 | 70799286 | gene-LOC114116384 |
| NC_056068.1 | 48170001 | 48175000 | 9.2217 | NC_056068.1 | 48173080 | 48174030 | gene-OR52A1 |
| NC_056060.1 | 24090001 | 24095000 | 9.2088 | NC_056060.1 | 24057259 | 24113912 | gene-CHD8 |
| NC_056077.1 | 42245001 | 42250000 | 9.1602 | NC_056077.1 | 42231857 | 42246376 | gene-LOC121817829 |
| NC_056077.1 | 42245001 | 42250000 | 9.1602 | NC_056077.1 | 42245722 | 42270006 | gene-DNAAF5 |
| NC_056066.1 | 51165001 | 51170000 | 9.1392 | NC_056066.1 | 51152602 | 51165689 | gene-C13H20orf27 |
| NC_056066.1 | 51165001 | 51170000 | 9.1392 | NC_056066.1 | 51166059 | 51183195 | gene-HSPA12B |
| NC_056054.1 | 193900001 | 193905000 | 9.1210 | NC_056054.1 | 193873135 | 193953226 | gene-ATP13A3 |
| NC_056054.1 | 193900001 | 193905000 | 9.1210 | NC_056054.1 | 193873135 | 193953226 | gene-ATP13A3 |
| NC_056068.1 | 82240001 | 82245000 | 9.0445 | NC_056068.1 | 82221201 | 82259659 | gene-GLB1L2 |
| NC_056057.1 | 47395001 | 47400000 | 9.0103 | NC_056057.1 | 47018289 | 47620058 | gene-LHFPL3 |
| NC_056061.1 | 29960001 | 29965000 | 9.0099 | NC_056061.1 | 29963519 | 29963590 | gene-TRNAS-GGA-142 |
| NC_056061.1 | 29960001 | 29965000 | 9.0099 | NC_056061.1 | 29911774 | 30192303 | gene-PDSS2 |
| NC_056072.1 | 49650001 | 49655000 | 8.9980 | NC_056072.1 | 49445628 | 49738202 | gene-DOCK3 |
| NC_056079.1 | 28350001 | 28355000 | 8.9942 | NC_056079.1 | 28353627 | 28371632 | gene-RNF122 |
| NC_056078.1 | 25550001 | 25555000 | 8.9782 | NC_056078.1 | 25499131 | 25552309 | gene-LOC101108627 |
| NC_056078.1 | 25550001 | 25555000 | 8.9782 | NC_056078.1 | 25530068 | 25569595 | gene-AIFM2 |
| NC_056077.1 | 14045001 | 14050000 | 8.9731 | NC_056077.1 | 13861238 | 14060689 | gene-PARN |
| NC_056057.1 | 52970001 | 52975000 | 8.8440 | NC_056057.1 | 52966990 | 53024674 | gene-CAPZA2 |
| NC_056059.1 | 89750001 | 89755000 | 8.7905 | NC_056059.1 | 89743677 | 89814078 | gene-LOC105615523 |
| NC_056060.1 | 24085001 | 24090000 | 8.7511 | NC_056060.1 | 24057259 | 24113912 | gene-CHD8 |
| NC_056072.1 | 22255001 | 22260000 | 8.7463 | NC_056072.1 | 22020655 | 22276169 | gene-LOC105603432 |
| NC_056080.1 | 116030001 | 116035000 | 8.7410 | NC_056080.1 | 116018640 | 116066895 | gene-LOC114111361 |
| NC_056077.1 | 17625001 | 17630000 | 8.6660 | NC_056077.1 | 17603859 | 17626626 | gene-CCP110 |
| NC_056077.1 | 17625001 | 17630000 | 8.6660 | NC_056077.1 | 17629213 | 17770263 | gene-VPS35L |
| NC_056054.1 | 227765001 | 227770000 | 8.6613 | NC_056054.1 | 227756622 | 227887283 | gene-IFT80 |
| NC_056054.1 | 227765001 | 227770000 | 8.6613 | NC_056054.1 | 227756622 | 227887283 | gene-IFT80 |
| NC_056054.1 | 165885001 | 165890000 | 8.6561 | NC_056054.1 | 165828601 | 165938781 | gene-LOC101110467 |
| NC_056054.1 | 165885001 | 165890000 | 8.6561 | NC_056054.1 | 165828601 | 165938781 | gene-LOC101110467 |
| NC_056054.1 | 69630001 | 69635000 | 8.6183 | NC_056054.1 | 69551721 | 69666728 | gene-RPAP2 |
| NC_056054.1 | 69630001 | 69635000 | 8.6183 | NC_056054.1 | 69551721 | 69666728 | gene-RPAP2 |
| NC_056063.1 | 67385001 | 67390000 | 8.5370 | NC_056063.1 | 66350875 | 67935545 | gene-GPC5 |
| NC_056061.1 | 11370001 | 11375000 | 8.5347 | NC_056061.1 | 11351810 | 11446472 | gene-RSPO3 |
| NC_056069.1 | 67745001 | 67750000 | 8.5125 | NC_056069.1 | 67724526 | 67793793 | gene-ICE1 |
| NC_056073.1 | 31820001 | 31825000 | 8.4911 | NC_056073.1 | 31792601 | 32010334 | gene-RIPOR2 |
| NC_056059.1 | 118310001 | 118315000 | 8.4890 | NC_056059.1 | 118312566 | 118320643 | gene-SLC49A3 |
| NC_056060.1 | 36405001 | 36410000 | 8.4540 | NC_056060.1 | 36405048 | 36566939 | gene-UBR1 |
| NC_056074.1 | 34790001 | 34795000 | 8.4477 | NC_056074.1 | 34794656 | 34816787 | gene-CD5 |
| NC_056057.1 | 29760001 | 29765000 | 8.4444 | NC_056057.1 | 29762578 | 29848259 | gene-MACC1 |
| NC_056069.1 | 31610001 | 31615000 | 8.4179 | NC_056069.1 | 31575652 | 31651805 | gene-NIM1K |
| NC_056070.1 | 45290001 | 45295000 | 8.4096 | NC_056070.1 | 45290559 | 45313399 | gene-MMP17 |
| NC_056054.1 | 69615001 | 69620000 | 8.3985 | NC_056054.1 | 69551721 | 69666728 | gene-RPAP2 |
| NC_056054.1 | 69615001 | 69620000 | 8.3985 | NC_056054.1 | 69551721 | 69666728 | gene-RPAP2 |
| NC_056059.1 | 91365001 | 91370000 | 8.2996 | NC_056059.1 | 91316458 | 91393341 | gene-USO1 |
| NC_056080.1 | 116020001 | 116025000 | 8.2569 | NC_056080.1 | 116018640 | 116066895 | gene-LOC114111361 |
| NC_056055.1 | 138905001 | 138910000 | 8.1707 | NC_056055.1 | 138895758 | 138966304 | gene-SP5 |
| NC_056070.1 | 57560001 | 57565000 | 8.1674 | NC_056070.1 | 57244765 | 57707051 | gene-KSR2 |
| NC_056078.1 | 25735001 | 25740000 | 8.1657 | NC_056078.1 | 25708805 | 25785442 | gene-LRRC20 |
| NC_056055.1 | 74820001 | 74825000 | 8.1529 | NC_056055.1 | 74717223 | 75115708 | gene-KDM4C |
| NC_056060.1 | 6885001 | 6890000 | 8.1469 | NC_056060.1 | 6780637 | 6919394 | gene-CERT1 |
| NC_056078.1 | 25560001 | 25565000 | 8.1466 | NC_056078.1 | 25530068 | 25569595 | gene-AIFM2 |
| NC_056056.1 | 140525001 | 140530000 | 8.1244 | NC_056056.1 | 140490987 | 140582894 | gene-SCAF11 |
| NC_056066.1 | 63885001 | 63890000 | 8.1013 | NC_056066.1 | 63886117 | 63903729 | gene-DYNLRB1 |
| NC_056056.1 | 170500001 | 170505000 | 8.0919 | NC_056056.1 | 170214791 | 170585805 | gene-ANO4 |
| NC_056059.1 | 117250001 | 117255000 | 8.0586 | NC_056059.1 | 117112675 | 117273551 | gene-POLN |
| NC_056057.1 | 100375001 | 100380000 | 8.0290 | NC_056057.1 | 100343957 | 100443489 | gene-AGBL3 |
| NC_056071.1 | 43675001 | 43680000 | 8.0243 | NC_056071.1 | 43525705 | 43726747 | gene-RALGAPA1 |
| NC_056071.1 | 1480001 | 1485000 | 8.0194 | NC_056071.1 | 1482792 | 1482830 | gene-LOC114109145 |
| NC_056071.1 | 1480001 | 1485000 | 8.0194 | NC_056071.1 | 1484234 | 1526103 | gene-LOC105603013 |
| NC_056058.1 | 57745001 | 57750000 | 8.0116 | NC_056058.1 | 57636031 | 57859371 | gene-HTR4 |
| NC_056056.1 | 180715001 | 180720000 | 8.0013 | NC_056056.1 | 180617874 | 180736165 | gene-CACNG2 |

**Table S20** The key genes associated with evolution in selected regions

| **Gene ID** | **Gene name** | **Function** |
| --- | --- | --- |
| gene-RPAP2 | putative RNA polymerase II subunit B1 CTD phosphatase RPAP2 isoform X1 | Regulates RNA polymerase II-mediated gene transcription (Orphanides et al., 1996). |
| gene-ATP13A3 | polyamine-transporting ATPase 13A3 isoform X1 | Involved in cellular polyamine transport and affects cellular energy metabolism (Hamouda et al., 2021). |
| gene-SP5 | transcription factor Sp5 isoform X1 | Regulates the transcription of multiple genes to affect multiple developmental and physiological processes (Harrison et al., 2000). |
| gene-TOR4A | torsin-4A | Regulates energy and protein transport (Rampello et al., 2020) |
| gene-CALCR | calcitonin receptor | Regulates bone development and calcium absorption (Shyu et al., 2007). |
| gene-KMT2C | histone-lysine N-methyltransferase 2C isoform X1 | Involved in the regulation of gene expression (Wang et al., 2023). |
| gene-SSBP2 | single-stranded DNA-binding protein 2 isoform X1 | Associated with DNA replication and repair (Rex et al., 2014). |
| gene-USO1 | general vesicular transport factor p115 isoform X1 | Plays an important role in protein transport (Keogh et al., 2022). |
| gene-MGA | MAX gene-associated protein isoform X1 | Regulates cell proliferation, differentiation, and death (Duarte et al., 2021). |
| gene-PDSS2 | all trans-polyprenyl-diphosphate synthase PDSS2 isoform X1 | Regulates mitochondrial uncoupling proteins and apoptosis (Hu et al., 2019). |
| gene-GPC5 | glypican-5 | Regulates cell proliferation and differentiation (Rahbari et al., 2019). |
| gene-XAF1 | XIAP-associated factor 1 isoform X1 | Regulates cell proliferation and apoptosis (Zhang et al., 2019). |
| gene-ITFG1 | T-cell immunomodulatory protein | Regulates T cell function and immune response (Cui et al., 2019). |
| gene-OR52A1 | olfactory receptor 52A1-like | Olfactory-related receptors (Kostka and Bitzenhofer, 2022). |
| gene-GLBIL2 | beta-galactosidase-1-like protein 2 isoform X1 | Plays a role in sugar metabolism and digestion (Belarbi et al., 2020). |
| gene-CMC1 | COX assembly mitochondrial protein homolog isoform X1 | Involved in cellular energy metabolism (Kaiafas et al., 2020). |
| gene-DUSP22 | dual specificity protein phosphatase 22 isoform X1 | Regulates neuronal function and cell signaling (Zhang et al., 2022). |
| gene-GMDS | GDP-mannose 4,6 dehydratase isoform X1 | Participates in the synthesis of polysaccharides and glycoproteins and plays an important role in the glycosylation of amino acids (Mao et al., 2022). |
| gene-NAV2 | neuron navigator 2 isoform X1 | Regulates the migration of neurons and the development of dendrites (Aly et al., 2020). |
| gene-TRAP1 | heat shock protein 75 kDa, mitochondrial isoform X1 | It plays a role in cellular metabolism and stress response (Kengkoom and Ampawong, 2015). |
| gene-AIFM2 | ferroptosis suppressor protein 1 isoform X1 | Plays a role in apoptosis, mitochondrial function, and cell metabolism (Watanabe et al., 2023). |
| gene-TIMM17B | mitochondrial import inner membrane translocase subunit Tim17-B | Plays an important role in mitochondrial function and cellular metabolism (Wang et al., 2022). |

**Table S21** The KEGG enrichment analysis of genes in selective sweep regions of Tibetan sheep and Argali (*P* < 0.05)

| **PathwayID** | **Pathway** | **list_number** | **total_number** | ***P*_value** | **FDR** |
| --- | --- | --- | --- | --- | --- |
| ko04520 | Adherens junction | 19 | 73 | 1.17E-07 | 0.000020 |
| ko04935 | Growth hormone synthesis, secretion and action | 25 | 121 | 1.72E-07 | 0.000020 |
| ko04151 | PI3K-Akt signaling pathway | 50 | 373 | 0.000001 | 0.000043 |
| ko04919 | Thyroid hormone signaling pathway | 24 | 123 | 0.000001 | 0.000053 |
| ko04020 | Calcium signaling pathway | 35 | 238 | 0.000004 | 0.000175 |
| ko04725 | Cholinergic synapse | 21 | 109 | 0.000005 | 0.000206 |
| ko04510 | Focal adhesion | 31 | 203 | 0.000006 | 0.000206 |
| ko04929 | GnRH secretion | 15 | 64 | 0.000010 | 0.000297 |
| ko04211 | Longevity regulating pathway | 18 | 90 | 0.000015 | 0.000379 |
| ko04390 | Hippo signaling pathway | 25 | 156 | 0.000022 | 0.000420 |
| ko04012 | ErbB signaling pathway | 17 | 84 | 0.000022 | 0.000420 |
| ko04024 | cAMP signaling pathway | 32 | 226 | 0.000022 | 0.000420 |
| ko04014 | Ras signaling pathway | 32 | 232 | 0.000037 | 0.000658 |
| ko04015 | Rap1 signaling pathway | 30 | 212 | 0.000040 | 0.000658 |
| ko04915 | Estrogen signaling pathway | 22 | 136 | 0.000058 | 0.000885 |
| ko04152 | AMPK signaling pathway | 20 | 121 | 0.000092 | 0.001253 |
| ko04072 | Phospholipase D signaling pathway | 23 | 150 | 0.000093 | 0.001253 |
| ko04010 | MAPK signaling pathway | 36 | 292 | 0.000137 | 0.001738 |
| ko04310 | Wnt signaling pathway | 24 | 168 | 0.000203 | 0.002450 |
| ko04261 | Adrenergic signaling in cardiomyocytes | 22 | 149 | 0.000230 | 0.002636 |
| ko04722 | Neurotrophin signaling pathway | 19 | 120 | 0.000244 | 0.002666 |
| ko04140 | Autophagy - animal | 21 | 143 | 0.000343 | 0.003566 |
| ko04917 | Prolactin signaling pathway | 15 | 86 | 0.000376 | 0.003741 |
| ko04660 | T cell receptor signaling pathway | 17 | 105 | 0.000392 | 0.003741 |
| ko04911 | Insulin secretion | 15 | 87 | 0.000428 | 0.003918 |
| ko04973 | Carbohydrate digestion and absorption | 10 | 45 | 0.000496 | 0.004343 |
| ko04728 | Dopaminergic synapse | 19 | 127 | 0.000512 | 0.004343 |
| ko04213 | Longevity regulating pathway - multiple species | 12 | 62 | 0.000541 | 0.004424 |
| ko04360 | Axon guidance | 24 | 180 | 0.000576 | 0.004552 |
| ko04910 | Insulin signaling pathway | 20 | 139 | 0.000616 | 0.004706 |
| ko04361 | Axon regeneration | 15 | 91 | 0.000701 | 0.005176 |
| ko04136 | Autophagy - other | 8 | 32 | 0.000788 | 0.005641 |
| ko04810 | Regulation of actin cytoskeleton | 27 | 218 | 0.000863 | 0.005986 |
| ko04371 | Apelin signaling pathway | 19 | 133 | 0.000915 | 0.006087 |
| ko04611 | Platelet activation | 18 | 123 | 0.000930 | 0.006087 |
| ko04713 | Circadian entrainment | 15 | 94 | 0.000992 | 0.006309 |
| ko04370 | VEGF signaling pathway | 11 | 59 | 0.001265 | 0.007830 |
| ko04062 | Chemokine signaling pathway | 24 | 191 | 0.001346 | 0.008114 |
| ko04926 | Relaxin signaling pathway | 18 | 129 | 0.001626 | 0.009548 |
| ko04341 | Hedgehog signaling pathway - fly | 8 | 36 | 0.001802 | 0.010257 |
| ko04071 | Sphingolipid signaling pathway | 17 | 120 | 0.001836 | 0.010257 |
| ko04916 | Melanogenesis | 15 | 101 | 0.002087 | 0.011378 |
| ko04662 | B cell receptor signaling pathway | 12 | 73 | 0.002399 | 0.012778 |
| ko04960 | Aldosterone-regulated sodium reabsorption | 8 | 38 | 0.002597 | 0.013515 |
| ko04923 | Regulation of lipolysis in adipocytes | 10 | 57 | 0.003327 | 0.016930 |
| ko04144 | Endocytosis | 28 | 252 | 0.003582 | 0.017685 |
| ko04927 | Cortisol synthesis and secretion | 11 | 67 | 0.003630 | 0.017685 |
| ko04921 | Oxytocin signaling pathway | 19 | 150 | 0.003772 | 0.017995 |
| ko04730 | Long-term depression | 10 | 59 | 0.004308 | 0.020135 |
| ko04724 | Glutamatergic synapse | 15 | 109 | 0.004415 | 0.020219 |
| ko04540 | Gap junction | 13 | 89 | 0.004677 | 0.021002 |
| ko04022 | cGMP-PKG signaling pathway | 20 | 165 | 0.004979 | 0.021928 |
| ko04924 | Renin secretion | 11 | 71 | 0.005722 | 0.024723 |
| ko04320 | Dorso-ventral axis formation | 6 | 27 | 0.006699 | 0.028409 |
| ko04625 | C-type lectin receptor signaling pathway | 14 | 104 | 0.007116 | 0.029628 |
| ko04150 | mTOR signaling pathway | 19 | 160 | 0.007601 | 0.031084 |
| ko04925 | Aldosterone synthesis and secretion | 13 | 96 | 0.008879 | 0.035672 |
| ko04340 | Hedgehog signaling pathway | 9 | 57 | 0.010568 | 0.041502 |
| ko04720 | Long-term potentiation | 10 | 67 | 0.010693 | 0.041502 |
| ko04013 | MAPK signaling pathway - fly | 12 | 88 | 0.011087 | 0.042314 |
| ko04391 | Hippo signaling pathway - fly | 10 | 68 | 0.011832 | 0.044419 |
| ko04270 | Vascular smooth muscle contraction | 16 | 136 | 0.014835 | 0.054795 |
| ko04120 | Ubiquitin mediated proteolysis | 17 | 148 | 0.015301 | 0.055396 |
| ko04726 | Serotonergic synapse | 14 | 114 | 0.015482 | 0.055396 |
| ko04920 | Adipocytokine signaling pathway | 10 | 72 | 0.017325 | 0.061037 |
| ko04214 | Apoptosis - fly | 8 | 52 | 0.018028 | 0.062553 |
| ko04530 | Tight junction | 19 | 175 | 0.018708 | 0.063944 |
| ko04928 | Parathyroid hormone synthesis, secretion and action | 13 | 106 | 0.019441 | 0.065471 |
| ko00562 | Inositol phosphate metabolism | 10 | 74 | 0.020689 | 0.068662 |
| ko04070 | Phosphatidylinositol signaling system | 12 | 97 | 0.022748 | 0.074417 |
| ko04550 | Signaling pathways regulating pluripotency of stem cells | 16 | 144 | 0.024275 | 0.078296 |
| ko04914 | Progesterone-mediated oocyte maturation | 11 | 88 | 0.026618 | 0.084659 |
| ko04380 | Osteoclast differentiation | 14 | 123 | 0.028130 | 0.088245 |
| ko04723 | Retrograde endocannabinoid signaling | 16 | 147 | 0.028789 | 0.089090 |
| ko04664 | Fc epsilon RI signaling pathway | 9 | 68 | 0.030914 | 0.094390 |
| ko04670 | Leukocyte transendothelial migration | 13 | 115 | 0.035264 | 0.106257 |
| ko04750 | Inflammatory mediator regulation of TRP channels | 12 | 106 | 0.041649 | 0.123864 |
| ko04666 | Fc gamma R-mediated phagocytosis | 11 | 95 | 0.043386 | 0.127377 |
| ko03450 | Non-homologous end-joining | 3 | 13 | 0.047880 | 0.135785 |
| ko00430 | Taurine and hypotaurine metabolism | 3 | 13 | 0.047880 | 0.135785 |
| ko04727 | GABAergic synapse | 10 | 85 | 0.048029 | 0.135785 |

**Table S22** The GO enrichment analysis of genes in selective sweep regions of Tibetan sheep and Argali (*P* < 0.05)

| **Category** | **GO.ID** | **Term** | **List** | **Total** | ***P*_value** | **FDR** |
| --- | --- | --- | --- | --- | --- | --- |
| BP | GO:0120036 | plasma membrane bounded cell projection organization | 109 | 829 | 5.23E-09 | 0.000026 |
| MF | GO:0004714 | transmembrane receptor protein tyrosine kinase activity | 24 | 85 | 6.16E-09 | 0.000003 |
| MF | GO:0016773 | phosphotransferase activity, alcohol group as acceptor | 84 | 602 | 7.64E-09 | 0.000003 |
| BP | GO:0030030 | cell projection organization | 110 | 846 | 8.28E-09 | 0.000026 |
| MF | GO:0004672 | protein kinase activity | 74 | 506 | 8.54E-09 | 0.000003 |
| CC | GO:0042995 | cell projection | 126 | 1077 | 1.56E-08 | 0.000008 |
| MF | GO:0016301 | kinase activity | 88 | 652 | 1.65E-08 | 0.000005 |
| CC | GO:0120025 | plasma membrane bounded cell projection | 122 | 1037 | 1.98E-08 | 0.000008 |
| MF | GO:0019199 | transmembrane receptor protein kinase activity | 25 | 101 | 5.45E-08 | 0.000013 |
| BP | GO:0007399 | nervous system development | 143 | 1238 | 9.72E-08 | 0.000202 |
| MF | GO:0004713 | protein tyrosine kinase activity | 24 | 99 | 1.54E-07 | 0.000031 |
| MF | GO:0032559 | adenyl ribonucleotide binding | 138 | 1232 | 2.32E-07 | 0.000041 |
| MF | GO:0030554 | adenyl nucleotide binding | 138 | 1236 | 2.81E-07 | 0.000043 |
| MF | GO:0016772 | transferase activity, transferring phosphorus-containing groups | 94 | 759 | 3.38E-07 | 0.000046 |
| BP | GO:0006935 | chemotaxis | 50 | 319 | 0.000001 | 0.000984 |
| MF | GO:0005524 | ATP binding | 133 | 1200 | 0.000001 | 0.000089 |
| BP | GO:0042330 | taxis | 50 | 321 | 0.000001 | 0.000984 |
| BP | GO:0040011 | locomotion | 93 | 763 | 0.000003 | 0.002050 |
| BP | GO:0048731 | system development | 240 | 2438 | 0.000003 | 0.002050 |
| BP | GO:0022008 | neurogenesis | 106 | 902 | 0.000003 | 0.002050 |
| CC | GO:0071944 | cell periphery | 283 | 3162 | 0.000009 | 0.002194 |
| BP | GO:0048468 | cell development | 130 | 1191 | 0.000009 | 0.005534 |
| BP | GO:0007411 | axon guidance | 24 | 120 | 0.000010 | 0.005534 |
| BP | GO:0007275 | multicellular organism development | 264 | 2781 | 0.000012 | 0.005534 |
| BP | GO:0097485 | neuron projection guidance | 24 | 122 | 0.000013 | 0.005534 |
| BP | GO:0000902 | cell morphogenesis | 72 | 574 | 0.000014 | 0.005534 |
| BP | GO:0048699 | generation of neurons | 91 | 774 | 0.000014 | 0.005534 |
| BP | GO:0043201 | response to leucine | 5 | 6 | 0.000014 | 0.005534 |
| BP | GO:0071233 | cellular response to leucine | 5 | 6 | 0.000014 | 0.005534 |
| CC | GO:0005886 | plasma membrane | 263 | 2925 | 0.000015 | 0.002899 |
| BP | GO:0030182 | neuron differentiation | 86 | 724 | 0.000017 | 0.006184 |
| MF | GO:0017076 | purine nucleotide binding | 154 | 1524 | 0.000020 | 0.002082 |
| MF | GO:0140096 | catalytic activity, acting on a protein | 172 | 1739 | 0.000020 | 0.002082 |
| BP | GO:0050794 | regulation of cellular process | 502 | 5858 | 0.000023 | 0.007275 |
| BP | GO:0010562 | positive regulation of phosphorus metabolic process | 56 | 420 | 0.000023 | 0.007275 |
| BP | GO:0045937 | positive regulation of phosphate metabolic process | 56 | 420 | 0.000023 | 0.007275 |
| BP | GO:0023052 | signaling | 299 | 3239 | 0.000025 | 0.007378 |
| BP | GO:0030031 | cell projection assembly | 46 | 324 | 0.000026 | 0.007474 |
| BP | GO:0060271 | cilium assembly | 34 | 214 | 0.000030 | 0.008222 |
| MF | GO:0032553 | ribonucleotide binding | 153 | 1525 | 0.000031 | 0.002820 |
| CC | GO:0098590 | plasma membrane region | 71 | 610 | 0.000032 | 0.004333 |
| MF | GO:0032555 | purine ribonucleotide binding | 152 | 1514 | 0.000032 | 0.002820 |
| BP | GO:0007154 | cell communication | 301 | 3274 | 0.000032 | 0.008393 |
| CC | GO:0045211 | postsynaptic membrane | 22 | 120 | 0.000039 | 0.004333 |
| BP | GO:0018193 | peptidyl-amino acid modification | 72 | 592 | 0.000039 | 0.009844 |
| CC | GO:0099061 | integral component of postsynaptic density membrane | 8 | 20 | 0.000040 | 0.004333 |
| BP | GO:0120031 | plasma membrane bounded cell projection assembly | 45 | 321 | 0.000044 | 0.010509 |
| BP | GO:1902017 | regulation of cilium assembly | 12 | 42 | 0.000045 | 0.010509 |
| MF | GO:0097367 | carbohydrate derivative binding | 165 | 1681 | 0.000048 | 0.003775 |
| MF | GO:0045296 | cadherin binding | 8 | 20 | 0.000049 | 0.003775 |
| BP | GO:0031175 | neuron projection development | 64 | 514 | 0.000053 | 0.011470 |
| BP | GO:0044782 | cilium organization | 35 | 229 | 0.000053 | 0.011470 |
| MF | GO:0035639 | purine ribonucleoside triphosphate binding | 147 | 1477 | 0.000069 | 0.004986 |
| BP | GO:0048666 | neuron development | 71 | 592 | 0.000070 | 0.014577 |
| BP | GO:0048856 | anatomical structure development | 300 | 3300 | 0.000085 | 0.017121 |
| CC | GO:0099146 | intrinsic component of postsynaptic density membrane | 8 | 22 | 0.000089 | 0.007418 |
| BP | GO:0009653 | anatomical structure morphogenesis | 162 | 1624 | 0.000096 | 0.018758 |
| CC | GO:0097060 | synaptic membrane | 26 | 163 | 0.000097 | 0.007418 |
| CC | GO:0098839 | postsynaptic density membrane | 9 | 28 | 0.000098 | 0.007418 |
| BP | GO:0007165 | signal transduction | 270 | 2936 | 0.000109 | 0.020128 |
| BP | GO:0051716 | cellular response to stimulus | 339 | 3803 | 0.000110 | 0.020128 |
| BP | GO:0072359 | circulatory system development | 75 | 644 | 0.000114 | 0.020128 |
| BP | GO:0001934 | positive regulation of protein phosphorylation | 46 | 344 | 0.000116 | 0.020128 |
| CC | GO:0042641 | actomyosin | 10 | 35 | 0.000123 | 0.008077 |
| BP | GO:0034329 | cell junction assembly | 33 | 220 | 0.000125 | 0.021094 |
| MF | GO:0036094 | small molecule binding | 182 | 1919 | 0.000126 | 0.007941 |
| CC | GO:0031256 | leading edge membrane | 15 | 71 | 0.000128 | 0.008077 |
| MF | GO:0000166 | nucleotide binding | 166 | 1725 | 0.000130 | 0.007941 |
| MF | GO:1901265 | nucleoside phosphate binding | 166 | 1725 | 0.000130 | 0.007941 |
| BP | GO:0032502 | developmental process | 317 | 3533 | 0.000134 | 0.022057 |
| MF | GO:0106310 | protein serine kinase activity | 23 | 136 | 0.000151 | 0.008787 |
| CC | GO:0098794 | postsynapse | 38 | 285 | 0.000157 | 0.008898 |
| BP | GO:0032989 | cellular component morphogenesis | 52 | 409 | 0.000158 | 0.025297 |
| BP | GO:1902115 | regulation of organelle assembly | 21 | 117 | 0.000177 | 0.027672 |
| CC | GO:0001725 | stress fiber | 9 | 30 | 0.000177 | 0.008898 |
| CC | GO:0097517 | contractile actin filament bundle | 9 | 30 | 0.000177 | 0.008898 |
| CC | GO:0031226 | intrinsic component of plasma membrane | 66 | 589 | 0.000188 | 0.008898 |
| MF | GO:0050839 | cell adhesion molecule binding | 21 | 121 | 0.000199 | 0.011066 |
| CC | GO:0031252 | cell leading edge | 32 | 228 | 0.000199 | 0.008898 |
| BP | GO:0042327 | positive regulation of phosphorylation | 48 | 373 | 0.000211 | 0.031685 |
| BP | GO:0032990 | cell part morphogenesis | 46 | 353 | 0.000213 | 0.031685 |
| MF | GO:0043168 | anion binding | 171 | 1804 | 0.000220 | 0.011741 |
| MF | GO:0043167 | ion binding | 336 | 3897 | 0.000235 | 0.012015 |
| BP | GO:0018210 | peptidyl-threonine modification | 15 | 71 | 0.000237 | 0.033816 |
| BP | GO:1901701 | cellular response to oxygen-containing compound | 62 | 520 | 0.000238 | 0.033816 |
| BP | GO:0120035 | regulation of plasma membrane bounded cell projection organization | 42 | 316 | 0.000260 | 0.036037 |
| CC | GO:0014069 | postsynaptic density | 20 | 118 | 0.000264 | 0.010579 |
| CC | GO:0032279 | asymmetric synapse | 20 | 118 | 0.000264 | 0.010579 |
| BP | GO:0071495 | cellular response to endogenous stimulus | 76 | 673 | 0.000273 | 0.036115 |
| BP | GO:0000904 | cell morphogenesis involved in differentiation | 51 | 408 | 0.000277 | 0.036115 |
| BP | GO:0051881 | regulation of mitochondrial membrane potential | 11 | 43 | 0.000278 | 0.036115 |
| BP | GO:0030334 | regulation of cell migration | 62 | 524 | 0.000294 | 0.037445 |
| CC | GO:0032432 | actin filament bundle | 9 | 32 | 0.000305 | 0.011602 |
| BP | GO:0018107 | peptidyl-threonine phosphorylation | 14 | 65 | 0.000307 | 0.037890 |
| BP | GO:0030335 | positive regulation of cell migration | 40 | 299 | 0.000316 | 0.037890 |
| CC | GO:0030424 | axon | 32 | 234 | 0.000321 | 0.011605 |
| BP | GO:0016192 | vesicle-mediated transport | 89 | 820 | 0.000321 | 0.037890 |
| BP | GO:0031401 | positive regulation of protein modification process | 59 | 494 | 0.000322 | 0.037890 |
| BP | GO:0031344 | regulation of cell projection organization | 42 | 320 | 0.000340 | 0.039373 |
| CC | GO:0005856 | cytoskeleton | 130 | 1364 | 0.000377 | 0.013016 |
| MF | GO:0055100 | adiponectin binding | 3 | 3 | 0.000404 | 0.019834 |
| BP | GO:0006468 | protein phosphorylation | 77 | 693 | 0.000409 | 0.046490 |
| BP | GO:0071596 | ubiquitin-dependent protein catabolic process via the N-end rule pathway | 3 | 3 | 0.000439 | 0.048298 |
| BP | GO:0045945 | positive regulation of transcription by RNA polymerase III | 4 | 6 | 0.000441 | 0.048298 |
| BP | GO:0043491 | protein kinase B signaling | 19 | 108 | 0.000462 | 0.048348 |
| BP | GO:0045471 | response to ethanol | 7 | 20 | 0.000463 | 0.048348 |
| MF | GO:0016740 | transferase activity | 163 | 1733 | 0.000465 | 0.021948 |
| BP | GO:0051641 | cellular localization | 172 | 1796 | 0.000468 | 0.048348 |
| BP | GO:0016477 | cell migration | 91 | 852 | 0.000472 | 0.048348 |
| CC | GO:0043005 | neuron projection | 56 | 496 | 0.000474 | 0.015668 |
| BP | GO:0065007 | biological regulation | 555 | 6716 | 0.000524 | 0.052752 |
| BP | GO:0040012 | regulation of locomotion | 66 | 580 | 0.000561 | 0.053870 |
| CC | GO:0098984 | neuron to neuron synapse | 21 | 134 | 0.000562 | 0.017800 |
| BP | GO:0045859 | regulation of protein kinase activity | 35 | 258 | 0.000566 | 0.053870 |
| BP | GO:0120039 | plasma membrane bounded cell projection morphogenesis | 43 | 338 | 0.000567 | 0.053870 |
| BP | GO:0007626 | locomotory behavior | 22 | 136 | 0.000574 | 0.053870 |
| BP | GO:0034330 | cell junction organization | 46 | 369 | 0.000578 | 0.053870 |
| MF | GO:0005488 | binding | 659 | 8352 | 0.000591 | 0.026848 |
| CC | GO:0005887 | integral component of plasma membrane | 60 | 546 | 0.000606 | 0.018422 |
| BP | GO:0030900 | forebrain development | 32 | 230 | 0.000620 | 0.055961 |
| BP | GO:0051179 | localization | 255 | 2823 | 0.000622 | 0.055961 |
| BP | GO:0070925 | organelle assembly | 59 | 507 | 0.000627 | 0.055961 |
| BP | GO:0048858 | cell projection morphogenesis | 43 | 340 | 0.000642 | 0.056277 |
| MF | GO:0030695 | GTPase regulator activity | 40 | 318 | 0.000648 | 0.027407 |
| MF | GO:0060589 | nucleoside-triphosphatase regulator activity | 40 | 318 | 0.000648 | 0.027407 |
| BP | GO:0008038 | neuron recognition | 7 | 21 | 0.000649 | 0.056277 |
| BP | GO:0048870 | cell motility | 99 | 953 | 0.000698 | 0.059329 |
| BP | GO:2000147 | positive regulation of cell motility | 40 | 311 | 0.000703 | 0.059329 |
| CC | GO:0099568 | cytoplasmic region | 20 | 127 | 0.000707 | 0.020399 |
| CC | GO:0030054 | cell junction | 103 | 1056 | 0.000731 | 0.020399 |
| BP | GO:0045913 | positive regulation of carbohydrate metabolic process | 9 | 34 | 0.000756 | 0.062989 |
| BP | GO:0048041 | focal adhesion assembly | 11 | 48 | 0.000768 | 0.063128 |
| CC | GO:0030122 | AP-2 adaptor complex | 4 | 7 | 0.000778 | 0.020399 |
| CC | GO:1990907 | beta-catenin-TCF complex | 4 | 7 | 0.000778 | 0.020399 |
| CC | GO:0031982 | vesicle | 98 | 999 | 0.000816 | 0.020662 |
| BP | GO:0001932 | regulation of protein phosphorylation | 61 | 534 | 0.000819 | 0.066456 |
| BP | GO:0018105 | peptidyl-serine phosphorylation | 24 | 158 | 0.000839 | 0.067157 |
| CC | GO:0005768 | endosome | 52 | 463 | 0.000843 | 0.020662 |
| BP | GO:0050805 | negative regulation of synaptic transmission | 8 | 28 | 0.000855 | 0.067603 |
| MF | GO:0003779 | actin binding | 35 | 271 | 0.000861 | 0.035195 |
| BP | GO:0040017 | positive regulation of locomotion | 40 | 315 | 0.000904 | 0.070586 |
| BP | GO:0007409 | axonogenesis | 32 | 236 | 0.000968 | 0.073776 |
| BP | GO:0048638 | regulation of developmental growth | 25 | 169 | 0.000978 | 0.073776 |
| BP | GO:1901699 | cellular response to nitrogen compound | 38 | 296 | 0.000981 | 0.073776 |
| CC | GO:0038201 | TOR complex | 5 | 12 | 0.000981 | 0.023293 |
| BP | GO:0051896 | regulation of protein kinase B signaling | 16 | 89 | 0.000994 | 0.073864 |
| BP | GO:0051347 | positive regulation of transferase activity | 30 | 217 | 0.001005 | 0.073864 |
| BP | GO:0042221 | response to chemical | 182 | 1947 | 0.001037 | 0.075277 |
| BP | GO:0007224 | smoothened signaling pathway | 17 | 98 | 0.001063 | 0.076279 |
| CC | GO:0099055 | integral component of postsynaptic membrane | 10 | 45 | 0.001106 | 0.024386 |
| CC | GO:0031410 | cytoplasmic vesicle | 92 | 937 | 0.001148 | 0.024386 |
| CC | GO:0005901 | caveola | 11 | 53 | 0.001159 | 0.024386 |
| CC | GO:0098857 | membrane microdomain | 23 | 161 | 0.001177 | 0.024386 |
| BP | GO:1901700 | response to oxygen-containing compound | 74 | 685 | 0.001179 | 0.082077 |
| CC | GO:0097708 | intracellular vesicle | 92 | 938 | 0.001187 | 0.024386 |
| BP | GO:0009719 | response to endogenous stimulus | 78 | 730 | 0.001194 | 0.082077 |
| BP | GO:0032869 | cellular response to insulin stimulus | 17 | 99 | 0.001194 | 0.082077 |
| BP | GO:0001974 | blood vessel remodeling | 7 | 23 | 0.001196 | 0.082077 |
| BP | GO:0051900 | regulation of mitochondrial depolarization | 5 | 12 | 0.001270 | 0.086182 |
| BP | GO:0032870 | cellular response to hormone stimulus | 34 | 260 | 0.001293 | 0.086646 |
| CC | GO:0030425 | dendrite | 26 | 192 | 0.001301 | 0.025348 |
| CC | GO:0097447 | dendritic tree | 26 | 192 | 0.001301 | 0.025348 |
| BP | GO:2000145 | regulation of cell motility | 62 | 555 | 0.001304 | 0.086646 |
| MF | GO:0019787 | ubiquitin-like protein transferase activity | 32 | 247 | 0.001337 | 0.052894 |
| BP | GO:0060322 | head development | 47 | 395 | 0.001382 | 0.089662 |
| BP | GO:0001952 | regulation of cell-matrix adhesion | 14 | 75 | 0.001386 | 0.089662 |
| CC | GO:0099572 | postsynaptic specialization | 20 | 134 | 0.001401 | 0.025976 |
| BP | GO:0071375 | cellular response to peptide hormone stimulus | 19 | 118 | 0.001405 | 0.089662 |
| BP | GO:0016310 | phosphorylation | 84 | 802 | 0.001410 | 0.089662 |
| BP | GO:0048869 | cellular developmental process | 208 | 2278 | 0.001421 | 0.089662 |
| BP | GO:0051338 | regulation of transferase activity | 43 | 354 | 0.001455 | 0.090842 |
| CC | GO:0030128 | clathrin coat of endocytic vesicle | 4 | 8 | 0.001468 | 0.025976 |
| CC | GO:0005930 | axoneme | 13 | 71 | 0.001470 | 0.025976 |
| CC | GO:0097014 | ciliary plasm | 13 | 71 | 0.001470 | 0.025976 |
| MF | GO:0005007 | fibroblast growth factor receptor activity | 3 | 4 | 0.001528 | 0.055106 |
| MF | GO:0046624 | sphingolipid transporter activity | 3 | 4 | 0.001528 | 0.055106 |
| MF | GO:0070728 | leucine binding | 3 | 4 | 0.001528 | 0.055106 |
| CC | GO:0099699 | integral component of synaptic membrane | 12 | 63 | 0.001552 | 0.026603 |
| BP | GO:0032879 | regulation of localization | 110 | 1104 | 0.001561 | 0.096495 |
| CC | GO:0098936 | intrinsic component of postsynaptic membrane | 10 | 47 | 0.001575 | 0.026603 |
| BP | GO:0007420 | brain development | 44 | 366 | 0.001584 | 0.096789 |
| BP | GO:0071354 | cellular response to interleukin-6 | 6 | 18 | 0.001596 | 0.096789 |
| BP | GO:0045860 | positive regulation of protein kinase activity | 22 | 147 | 0.001651 | 0.098321 |
| BP | GO:0007158 | neuron cell-cell adhesion | 3 | 4 | 0.001656 | 0.098321 |
| BP | GO:0007417 | central nervous system development | 57 | 506 | 0.001669 | 0.098321 |
| BP | GO:0010810 | regulation of cell-substrate adhesion | 20 | 129 | 0.001712 | 0.099471 |
| BP | GO:0032868 | response to insulin | 19 | 120 | 0.001720 | 0.099471 |
| CC | GO:0032838 | plasma membrane bounded cell projection cytoplasm | 17 | 108 | 0.001735 | 0.028667 |
| BP | GO:0018209 | peptidyl-serine modification | 25 | 176 | 0.001754 | 0.100494 |
| BP | GO:0071417 | cellular response to organonitrogen compound | 34 | 265 | 0.001791 | 0.101073 |
| BP | GO:0035701 | hematopoietic stem cell migration | 4 | 8 | 0.001817 | 0.101073 |
| BP | GO:0043217 | myelin maintenance | 4 | 8 | 0.001817 | 0.101073 |
| BP | GO:0051247 | positive regulation of protein metabolic process | 77 | 730 | 0.001834 | 0.101073 |
| BP | GO:0044087 | regulation of cellular component biogenesis | 60 | 541 | 0.001845 | 0.101073 |
| CC | GO:0099060 | integral component of postsynaptic specialization membrane | 8 | 33 | 0.001904 | 0.030785 |
| BP | GO:0031399 | regulation of protein modification process | 82 | 788 | 0.001932 | 0.101535 |
| BP | GO:0022011 | myelination in peripheral nervous system | 5 | 13 | 0.001935 | 0.101535 |
| BP | GO:0032292 | peripheral nervous system axon ensheathment | 5 | 13 | 0.001935 | 0.101535 |
| BP | GO:0051882 | mitochondrial depolarization | 5 | 13 | 0.001935 | 0.101535 |
| BP | GO:0055003 | cardiac myofibril assembly | 5 | 13 | 0.001935 | 0.101535 |
| CC | GO:0031253 | cell projection membrane | 20 | 138 | 0.002011 | 0.031847 |
| BP | GO:0008104 | protein localization | 126 | 1301 | 0.002035 | 0.104388 |
| CC | GO:0015629 | actin cytoskeleton | 35 | 293 | 0.002059 | 0.031928 |
| BP | GO:0007610 | behavior | 44 | 371 | 0.002068 | 0.104388 |
| BP | GO:0043549 | regulation of kinase activity | 37 | 298 | 0.002072 | 0.104388 |
| BP | GO:0019220 | regulation of phosphate metabolic process | 76 | 722 | 0.002073 | 0.104388 |
| BP | GO:0051174 | regulation of phosphorus metabolic process | 76 | 722 | 0.002073 | 0.104388 |
| CC | GO:0045202 | synapse | 66 | 645 | 0.002121 | 0.032237 |
| BP | GO:1905515 | non-motile cilium assembly | 9 | 39 | 0.002168 | 0.108280 |
| BP | GO:0035640 | exploration behavior | 6 | 19 | 0.002185 | 0.108280 |
| MF | GO:0008092 | cytoskeletal protein binding | 64 | 605 | 0.002190 | 0.076729 |
| BP | GO:0043434 | response to peptide hormone | 21 | 141 | 0.002213 | 0.108812 |
| BP | GO:0070727 | cellular macromolecule localization | 126 | 1305 | 0.002275 | 0.110505 |
| BP | GO:0009725 | response to hormone | 38 | 310 | 0.002283 | 0.110505 |
| BP | GO:0050920 | regulation of chemotaxis | 18 | 114 | 0.002320 | 0.111467 |
| MF | GO:0042578 | phosphoric ester hydrolase activity | 33 | 266 | 0.002335 | 0.079521 |
| CC | GO:0044853 | plasma membrane raft | 12 | 66 | 0.002348 | 0.034449 |
| BP | GO:0001822 | kidney development | 25 | 180 | 0.002399 | 0.114283 |
| CC | GO:0030118 | clathrin coat | 7 | 27 | 0.002405 | 0.034449 |
| CC | GO:0045121 | membrane raft | 22 | 160 | 0.002441 | 0.034449 |
| CC | GO:0030669 | clathrin-coated endocytic vesicle membrane | 4 | 9 | 0.002493 | 0.034449 |
| CC | GO:0031258 | lamellipodium membrane | 4 | 9 | 0.002493 | 0.034449 |
| BP | GO:0007044 | cell-substrate junction assembly | 11 | 55 | 0.002497 | 0.114283 |
| MF | GO:0004842 | ubiquitin-protein transferase activity | 30 | 236 | 0.002498 | 0.082781 |
| BP | GO:0033674 | positive regulation of kinase activity | 24 | 171 | 0.002523 | 0.114283 |
| BP | GO:0072659 | protein localization to plasma membrane | 24 | 171 | 0.002523 | 0.114283 |
| BP | GO:0050790 | regulation of catalytic activity | 91 | 899 | 0.002537 | 0.114283 |
| BP | GO:0018108 | peptidyl-tyrosine phosphorylation | 22 | 152 | 0.002542 | 0.114283 |
| BP | GO:0065009 | regulation of molecular function | 127 | 1321 | 0.002548 | 0.114283 |
| BP | GO:0070887 | cellular response to chemical stimulus | 144 | 1525 | 0.002609 | 0.114283 |
| BP | GO:0051893 | regulation of focal adhesion assembly | 9 | 40 | 0.002612 | 0.114283 |
| BP | GO:0090109 | regulation of cell-substrate junction assembly | 9 | 40 | 0.002612 | 0.114283 |
| CC | GO:0005929 | cilium | 45 | 408 | 0.002617 | 0.035516 |
| BP | GO:0051972 | regulation of telomerase activity | 7 | 26 | 0.002625 | 0.114283 |
| BP | GO:1903533 | regulation of protein targeting | 7 | 26 | 0.002625 | 0.114283 |
| BP | GO:0061564 | axon development | 33 | 261 | 0.002635 | 0.114283 |
| BP | GO:1901653 | cellular response to peptide | 21 | 143 | 0.002635 | 0.114283 |
| CC | GO:0098978 | glutamatergic synapse | 25 | 192 | 0.002748 | 0.036637 |
| CC | GO:0098948 | intrinsic component of postsynaptic specialization membrane | 8 | 35 | 0.002840 | 0.037213 |
| BP | GO:0022607 | cellular component assembly | 145 | 1541 | 0.002885 | 0.122520 |
| BP | GO:0071310 | cellular response to organic substance | 117 | 1207 | 0.002885 | 0.122520 |
| BP | GO:0045724 | positive regulation of cilium assembly | 6 | 20 | 0.002923 | 0.122520 |
| BP | GO:0070741 | response to interleukin-6 | 6 | 20 | 0.002923 | 0.122520 |
| BP | GO:0048667 | cell morphogenesis involved in neuron differentiation | 37 | 304 | 0.002928 | 0.122520 |
| BP | GO:0042325 | regulation of phosphorylation | 65 | 608 | 0.002982 | 0.122520 |
| BP | GO:0018212 | peptidyl-tyrosine modification | 22 | 154 | 0.002998 | 0.122520 |
| BP | GO:0042391 | regulation of membrane potential | 25 | 183 | 0.003007 | 0.122520 |
| CC | GO:0099634 | postsynaptic specialization membrane | 9 | 43 | 0.003008 | 0.038747 |
| BP | GO:0043085 | positive regulation of catalytic activity | 63 | 586 | 0.003020 | 0.122520 |
| BP | GO:0007507 | heart development | 38 | 315 | 0.003021 | 0.122520 |
| BP | GO:0050789 | regulation of biological process | 517 | 6300 | 0.003044 | 0.122648 |
| BP | GO:0032570 | response to progesterone | 4 | 9 | 0.003074 | 0.123077 |
| CC | GO:0045334 | clathrin-coated endocytic vesicle | 5 | 15 | 0.003101 | 0.039282 |
| BP | GO:0031032 | actomyosin structure organization | 18 | 118 | 0.003411 | 0.135273 |
| BP | GO:0044093 | positive regulation of molecular function | 83 | 816 | 0.003422 | 0.135273 |
| CC | GO:0099240 | intrinsic component of synaptic membrane | 12 | 69 | 0.003450 | 0.042978 |
| BP | GO:0048812 | neuron projection morphogenesis | 39 | 328 | 0.003458 | 0.135822 |
| BP | GO:1990778 | protein localization to cell periphery | 26 | 195 | 0.003514 | 0.137151 |
| MF | GO:0098631 | cell adhesion mediator activity | 5 | 15 | 0.003526 | 0.110644 |
| MF | GO:0004726 | non-membrane spanning protein tyrosine phosphatase activity | 3 | 5 | 0.003610 | 0.110644 |
| MF | GO:0005021 | vascular endothelial growth factor receptor activity | 3 | 5 | 0.003610 | 0.110644 |
| BP | GO:0001944 | vasculature development | 50 | 447 | 0.003626 | 0.140538 |
| BP | GO:0099536 | synaptic signaling | 39 | 329 | 0.003646 | 0.140538 |
| BP | GO:0099537 | trans-synaptic signaling | 38 | 319 | 0.003752 | 0.143752 |
| BP | GO:0060491 | regulation of cell projection assembly | 17 | 110 | 0.003806 | 0.144941 |
| BP | GO:0010518 | positive regulation of phospholipase activity | 6 | 21 | 0.003832 | 0.145043 |
| BP | GO:0150115 | cell-substrate junction organization | 11 | 58 | 0.003857 | 0.145108 |
| BP | GO:0032287 | peripheral nervous system myelin maintenance | 3 | 5 | 0.003906 | 0.145821 |
| CC | GO:0030132 | clathrin coat of coated pit | 4 | 10 | 0.003920 | 0.047290 |
| CC | GO:0031932 | TORC2 complex | 4 | 10 | 0.003920 | 0.047290 |
| BP | GO:0007155 | cell adhesion | 76 | 740 | 0.003944 | 0.145821 |
| BP | GO:0014044 | Schwann cell development | 5 | 15 | 0.003972 | 0.145821 |
| BP | GO:0051668 | localization within membrane | 41 | 352 | 0.003996 | 0.145821 |
| BP | GO:0072001 | renal system development | 25 | 187 | 0.004015 | 0.145821 |
| BP | GO:0001954 | positive regulation of cell-matrix adhesion | 8 | 35 | 0.004028 | 0.145821 |
| BP | GO:0031589 | cell-substrate adhesion | 27 | 207 | 0.004040 | 0.145821 |
| BP | GO:0023051 | regulation of signaling | 171 | 1871 | 0.004063 | 0.145821 |
| BP | GO:0014706 | striated muscle tissue development | 18 | 120 | 0.004098 | 0.146054 |
| BP | GO:0032008 | positive regulation of TOR signaling | 7 | 28 | 0.004135 | 0.146054 |
| BP | GO:0032501 | multicellular organismal process | 338 | 3972 | 0.004173 | 0.146054 |
| BP | GO:0072006 | nephron development | 15 | 93 | 0.004228 | 0.146054 |
| BP | GO:0001657 | ureteric bud development | 12 | 67 | 0.004243 | 0.146054 |
| BP | GO:0072163 | mesonephric epithelium development | 12 | 67 | 0.004243 | 0.146054 |
| BP | GO:0072164 | mesonephric tubule development | 12 | 67 | 0.004243 | 0.146054 |
| CC | GO:0030904 | retromer complex | 5 | 16 | 0.004247 | 0.050434 |
| BP | GO:0051128 | regulation of cellular component organization | 119 | 1245 | 0.004256 | 0.146054 |
| MF | GO:0030552 | cAMP binding | 4 | 10 | 0.004363 | 0.119873 |
| MF | GO:0035004 | phosphatidylinositol 3-kinase activity | 4 | 10 | 0.004363 | 0.119873 |
| MF | GO:0098918 | structural constituent of synapse | 4 | 10 | 0.004363 | 0.119873 |
| MF | GO:0005085 | guanyl-nucleotide exchange factor activity | 23 | 173 | 0.004365 | 0.119873 |
| BP | GO:0150116 | regulation of cell-substrate junction organization | 9 | 43 | 0.004388 | 0.149759 |
| MF | GO:0004674 | protein serine/threonine kinase activity | 29 | 235 | 0.004495 | 0.119873 |
| BP | GO:0001655 | urogenital system development | 27 | 209 | 0.004611 | 0.156494 |
| BP | GO:0035556 | intracellular signal transduction | 137 | 1464 | 0.004647 | 0.156851 |
| BP | GO:0072073 | kidney epithelium development | 15 | 94 | 0.004687 | 0.157363 |
| BP | GO:0006206 | pyrimidine nucleobase metabolic process | 4 | 10 | 0.004818 | 0.159715 |
| BP | GO:0071625 | vocalization behavior | 4 | 10 | 0.004818 | 0.159715 |
| BP | GO:0030154 | cell differentiation | 202 | 2261 | 0.004834 | 0.159715 |
| BP | GO:0051973 | positive regulation of telomerase activity | 6 | 22 | 0.004936 | 0.161708 |
| BP | GO:0009987 | cellular process | 758 | 9677 | 0.004946 | 0.161708 |
| BP | GO:0032092 | positive regulation of protein binding | 11 | 60 | 0.005054 | 0.163814 |
| BP | GO:0021537 | telencephalon development | 21 | 151 | 0.005064 | 0.163814 |
| BP | GO:0048762 | mesenchymal cell differentiation | 19 | 132 | 0.005137 | 0.163814 |
| CC | GO:0060171 | stereocilium membrane | 2 | 2 | 0.005161 | 0.058171 |
| CC | GO:1902500 | vacuolar HOPS complex | 2 | 2 | 0.005161 | 0.058171 |
| CC | GO:1902501 | lysosomal HOPS complex | 2 | 2 | 0.005161 | 0.058171 |
| CC | GO:0036477 | somatodendritic compartment | 32 | 277 | 0.005205 | 0.058171 |
| MF | GO:0016791 | phosphatase activity | 25 | 196 | 0.005250 | 0.119873 |
| BP | GO:0001823 | mesonephros development | 12 | 69 | 0.005425 | 0.163814 |
| MF | GO:0001002 | RNA polymerase III type 1 promoter sequence-specific DNA binding | 2 | 2 | 0.005475 | 0.119873 |
| MF | GO:0001003 | RNA polymerase III type 2 promoter sequence-specific DNA binding | 2 | 2 | 0.005475 | 0.119873 |
| MF | GO:0001025 | RNA polymerase III general transcription initiation factor binding | 2 | 2 | 0.005475 | 0.119873 |
| MF | GO:0001156 | TFIIIC-class transcription factor complex binding | 2 | 2 | 0.005475 | 0.119873 |
| MF | GO:0046556 | alpha-L-arabinofuranosidase activity | 2 | 2 | 0.005475 | 0.119873 |
| MF | GO:0080084 | 5S rDNA binding | 2 | 2 | 0.005475 | 0.119873 |
| MF | GO:0097003 | adipokinetic hormone receptor activity | 2 | 2 | 0.005475 | 0.119873 |
| MF | GO:0099583 | neurotransmitter receptor activity involved in regulation of postsynaptic cytosolic calcium ion concentration | 2 | 2 | 0.005475 | 0.119873 |
| MF | GO:0140351 | glycosylceramide flippase activity | 2 | 2 | 0.005475 | 0.119873 |
| MF | GO:0140828 | metal cation:monoatomic cation antiporter activity | 6 | 23 | 0.005475 | 0.119873 |
| CC | GO:0043204 | perikaryon | 7 | 31 | 0.005538 | 0.061000 |
| BP | GO:0030029 | actin filament-based process | 50 | 457 | 0.005599 | 0.163814 |
| BP | GO:0048771 | tissue remodeling | 16 | 105 | 0.005648 | 0.163814 |
| BP | GO:0048589 | developmental growth | 43 | 381 | 0.005734 | 0.163814 |
| BP | GO:0060627 | regulation of vesicle-mediated transport | 32 | 264 | 0.005770 | 0.163814 |
| BP | GO:1900076 | regulation of cellular response to insulin stimulus | 8 | 37 | 0.005774 | 0.163814 |
| BP | GO:0010956 | negative regulation of calcidiol 1-monooxygenase activity | 2 | 2 | 0.005783 | 0.163814 |
| BP | GO:0019566 | arabinose metabolic process | 2 | 2 | 0.005783 | 0.163814 |
| BP | GO:0032286 | central nervous system myelin maintenance | 2 | 2 | 0.005783 | 0.163814 |
| BP | GO:0035552 | oxidative single-stranded DNA demethylation | 2 | 2 | 0.005783 | 0.163814 |
| BP | GO:0038110 | interleukin-2-mediated signaling pathway | 2 | 2 | 0.005783 | 0.163814 |
| BP | GO:0038116 | chemokine (C-C motif) ligand 21 signaling pathway | 2 | 2 | 0.005783 | 0.163814 |
| BP | GO:0038129 | ERBB3 signaling pathway | 2 | 2 | 0.005783 | 0.163814 |
| BP | GO:0046373 | L-arabinose metabolic process | 2 | 2 | 0.005783 | 0.163814 |
| BP | GO:0060916 | mesenchymal cell proliferation involved in lung development | 2 | 2 | 0.005783 | 0.163814 |
| BP | GO:0070103 | regulation of interleukin-6-mediated signaling pathway | 2 | 2 | 0.005783 | 0.163814 |
| BP | GO:0071352 | cellular response to interleukin-2 | 2 | 2 | 0.005783 | 0.163814 |
| BP | GO:0072658 | maintenance of protein location in membrane | 2 | 2 | 0.005783 | 0.163814 |
| BP | GO:0072660 | maintenance of protein location in plasma membrane | 2 | 2 | 0.005783 | 0.163814 |
| BP | GO:0097749 | membrane tubulation | 2 | 2 | 0.005783 | 0.163814 |
| BP | GO:0097750 | endosome membrane tubulation | 2 | 2 | 0.005783 | 0.163814 |
| BP | GO:0140285 | endosome fission | 2 | 2 | 0.005783 | 0.163814 |
| BP | GO:1902227 | negative regulation of macrophage colony-stimulating factor signaling pathway | 2 | 2 | 0.005783 | 0.163814 |
| BP | GO:1903760 | regulation of voltage-gated potassium channel activity involved in ventricular cardiac muscle cell action potential repolarization | 2 | 2 | 0.005783 | 0.163814 |
| BP | GO:1904016 | response to Thyroglobulin triiodothyronine | 2 | 2 | 0.005783 | 0.163814 |
| BP | GO:1904017 | cellular response to Thyroglobulin triiodothyronine | 2 | 2 | 0.005783 | 0.163814 |
| BP | GO:1901888 | regulation of cell junction assembly | 18 | 124 | 0.005816 | 0.163814 |
| BP | GO:0030036 | actin cytoskeleton organization | 46 | 414 | 0.005823 | 0.163814 |
| MF | GO:0019899 | enzyme binding | 110 | 1181 | 0.005851 | 0.124950 |
| MF | GO:0030234 | enzyme regulator activity | 69 | 690 | 0.005911 | 0.124950 |
| BP | GO:1905477 | positive regulation of protein localization to membrane | 10 | 53 | 0.005975 | 0.167010 |
| BP | GO:0007010 | cytoskeleton organization | 82 | 822 | 0.006013 | 0.167010 |
| BP | GO:0046620 | regulation of organ growth | 9 | 45 | 0.006017 | 0.167010 |
| BP | GO:0001568 | blood vessel development | 47 | 426 | 0.006096 | 0.168449 |
| BP | GO:0010646 | regulation of cell communication | 169 | 1866 | 0.006203 | 0.170479 |
| BP | GO:0035307 | positive regulation of protein dephosphorylation | 7 | 30 | 0.006224 | 0.170479 |
| BP | GO:1902930 | regulation of alcohol biosynthetic process | 6 | 23 | 0.006255 | 0.170592 |
| CC | GO:0031931 | TORC1 complex | 3 | 6 | 0.006278 | 0.068160 |
| BP | GO:0035239 | tube morphogenesis | 57 | 538 | 0.006347 | 0.172343 |
| MF | GO:0008569 | minus-end-directed microtubule motor activity | 5 | 17 | 0.006419 | 0.133381 |
| BP | GO:0050896 | response to stimulus | 388 | 4646 | 0.006513 | 0.175666 |
| BP | GO:0097305 | response to alcohol | 11 | 62 | 0.006526 | 0.175666 |
| BP | GO:0002573 | myeloid leukocyte differentiation | 19 | 135 | 0.006558 | 0.175782 |
| MF | GO:0099186 | structural constituent of postsynapse | 3 | 6 | 0.006823 | 0.139424 |
| BP | GO:0007156 | homophilic cell adhesion via plasma membrane adhesion molecules | 12 | 71 | 0.006855 | 0.182948 |
| BP | GO:0006359 | regulation of transcription by RNA polymerase III | 4 | 11 | 0.007119 | 0.185933 |
| BP | GO:0010715 | regulation of extracellular matrix disassembly | 4 | 11 | 0.007119 | 0.185933 |
| BP | GO:0035641 | locomotory exploration behavior | 4 | 11 | 0.007119 | 0.185933 |
| BP | GO:0050765 | negative regulation of phagocytosis | 4 | 11 | 0.007119 | 0.185933 |
| BP | GO:0070102 | interleukin-6-mediated signaling pathway | 4 | 11 | 0.007119 | 0.185933 |
| BP | GO:0007264 | small GTPase mediated signal transduction | 32 | 268 | 0.007191 | 0.185933 |
| BP | GO:0048799 | animal organ maturation | 5 | 17 | 0.007205 | 0.185933 |
| BP | GO:0090313 | regulation of protein targeting to membrane | 5 | 17 | 0.007205 | 0.185933 |
| BP | GO:0010975 | regulation of neuron projection development | 26 | 206 | 0.007270 | 0.186291 |
| BP | GO:0006207 | 'de novo' pyrimidine nucleobase biosynthetic process | 3 | 6 | 0.007372 | 0.186291 |
| BP | GO:0019856 | pyrimidine nucleobase biosynthetic process | 3 | 6 | 0.007372 | 0.186291 |
| BP | GO:0045162 | clustering of voltage-gated sodium channels | 3 | 6 | 0.007372 | 0.186291 |
| BP | GO:0006813 | potassium ion transport | 11 | 63 | 0.007378 | 0.186291 |
| BP | GO:0062197 | cellular response to chemical stress | 24 | 186 | 0.007404 | 0.186291 |
| BP | GO:1901652 | response to peptide | 23 | 176 | 0.007428 | 0.186291 |
| BP | GO:0006109 | regulation of carbohydrate metabolic process | 15 | 99 | 0.007628 | 0.190536 |
| BP | GO:0032880 | regulation of protein localization | 49 | 454 | 0.007811 | 0.192267 |
| BP | GO:0010676 | positive regulation of cellular carbohydrate metabolic process | 6 | 24 | 0.007814 | 0.192267 |
| BP | GO:0060043 | regulation of cardiac muscle cell proliferation | 6 | 24 | 0.007814 | 0.192267 |
| BP | GO:0001763 | morphogenesis of a branching structure | 20 | 147 | 0.007820 | 0.192267 |
| CC | GO:0030027 | lamellipodium | 16 | 115 | 0.008023 | 0.085876 |
| BP | GO:0120032 | regulation of plasma membrane bounded cell projection assembly | 16 | 109 | 0.008105 | 0.198504 |
| BP | GO:0051234 | establishment of localization | 215 | 2451 | 0.008301 | 0.202503 |
| MF | GO:0030551 | cyclic nucleotide binding | 5 | 18 | 0.008355 | 0.167571 |
| MF | GO:0004725 | protein tyrosine phosphatase activity | 13 | 84 | 0.008474 | 0.167571 |
| BP | GO:0007160 | cell-matrix adhesion | 18 | 129 | 0.008746 | 0.212535 |
| CC | GO:0042383 | sarcolemma | 10 | 59 | 0.008764 | 0.092511 |
| BP | GO:0033036 | macromolecule localization | 141 | 1540 | 0.008805 | 0.213129 |
| BP | GO:0035295 | tube development | 69 | 684 | 0.008863 | 0.213260 |
| CC | GO:0012506 | vesicle membrane | 39 | 367 | 0.008948 | 0.093158 |
| BP | GO:0010463 | mesenchymal cell proliferation | 7 | 32 | 0.009012 | 0.213260 |
| BP | GO:0035249 | synaptic transmission, glutamatergic | 7 | 32 | 0.009012 | 0.213260 |
| BP | GO:1902116 | negative regulation of organelle assembly | 7 | 32 | 0.009012 | 0.213260 |
| BP | GO:0007268 | chemical synaptic transmission | 36 | 315 | 0.009015 | 0.213260 |
| BP | GO:0098916 | anterograde trans-synaptic signaling | 36 | 315 | 0.009015 | 0.213260 |
| BP | GO:0002275 | myeloid cell activation involved in immune response | 9 | 48 | 0.009287 | 0.216841 |
| BP | GO:0032147 | activation of protein kinase activity | 9 | 48 | 0.009287 | 0.216841 |
| BP | GO:0010800 | positive regulation of peptidyl-threonine phosphorylation | 5 | 18 | 0.009362 | 0.216841 |
| BP | GO:0014037 | Schwann cell differentiation | 5 | 18 | 0.009362 | 0.216841 |
| BP | GO:0051647 | nucleus localization | 5 | 18 | 0.009362 | 0.216841 |
| BP | GO:0051649 | establishment of localization in cell | 101 | 1061 | 0.009375 | 0.216841 |
| BP | GO:0060560 | developmental growth involved in morphogenesis | 18 | 130 | 0.009455 | 0.217879 |
| BP | GO:0006470 | protein dephosphorylation | 13 | 83 | 0.009539 | 0.218787 |
| BP | GO:0048738 | cardiac muscle tissue development | 16 | 111 | 0.009621 | 0.218787 |
| BP | GO:0046777 | protein autophosphorylation | 19 | 140 | 0.009626 | 0.218787 |
| BP | GO:0010517 | regulation of phospholipase activity | 6 | 25 | 0.009634 | 0.218787 |
| BP | GO:0072657 | protein localization to membrane | 35 | 306 | 0.009813 | 0.222040 |
| BP | GO:0001667 | ameboidal-type cell migration | 30 | 253 | 0.009957 | 0.223811 |
| CC | GO:0030659 | cytoplasmic vesicle membrane | 38 | 358 | 0.009959 | 0.102278 |
| BP | GO:0032148 | activation of protein kinase B activity | 4 | 12 | 0.010044 | 0.223811 |
| BP | GO:0072673 | lamellipodium morphogenesis | 4 | 12 | 0.010044 | 0.223811 |
| BP | GO:0051897 | positive regulation of protein kinase B signaling | 10 | 57 | 0.010057 | 0.223811 |
| BP | GO:0001501 | skeletal system development | 38 | 339 | 0.010071 | 0.223811 |
| MF | GO:0015298 | solute:cation antiporter activity | 6 | 26 | 0.010327 | 0.200974 |
| BP | GO:0050730 | regulation of peptidyl-tyrosine phosphorylation | 19 | 141 | 0.010360 | 0.229417 |
| CC | GO:1990316 | Atg1/ULK1 kinase complex | 3 | 7 | 0.010403 | 0.105413 |
| BP | GO:0060348 | bone development | 20 | 151 | 0.010434 | 0.229660 |
| BP | GO:0060485 | mesenchyme development | 21 | 161 | 0.010444 | 0.229660 |
| MF | GO:0061659 | ubiquitin-like protein ligase activity | 19 | 145 | 0.010518 | 0.201094 |
| BP | GO:0051173 | positive regulation of nitrogen compound metabolic process | 131 | 1427 | 0.010641 | 0.233177 |
| MF | GO:0051219 | phosphoprotein binding | 10 | 59 | 0.010662 | 0.201094 |
| BP | GO:0008347 | glial cell migration | 7 | 33 | 0.010706 | 0.233771 |
| CC | GO:0010008 | endosome membrane | 23 | 192 | 0.010783 | 0.107718 |
| BP | GO:0022029 | telencephalon cell migration | 8 | 41 | 0.010921 | 0.235985 |
| BP | GO:0035306 | positive regulation of dephosphorylation | 8 | 41 | 0.010921 | 0.235985 |
| BP | GO:0046425 | regulation of receptor signaling pathway via JAK-STAT | 8 | 41 | 0.010921 | 0.235985 |
| BP | GO:0030155 | regulation of cell adhesion | 45 | 418 | 0.010982 | 0.236490 |
| CC | GO:0005905 | clathrin-coated pit | 7 | 35 | 0.011029 | 0.107718 |
| CC | GO:0031674 | I band | 10 | 61 | 0.011055 | 0.107718 |
| CC | GO:0032589 | neuron projection membrane | 4 | 13 | 0.011219 | 0.107934 |
| MF | GO:0043422 | protein kinase B binding | 3 | 7 | 0.011288 | 0.206549 |
| MF | GO:0070016 | armadillo repeat domain binding | 3 | 7 | 0.011288 | 0.206549 |
| BP | GO:0033138 | positive regulation of peptidyl-serine phosphorylation | 10 | 58 | 0.011349 | 0.243554 |
| BP | GO:0050806 | positive regulation of synaptic transmission | 11 | 67 | 0.011684 | 0.249313 |
| BP | GO:0016242 | negative regulation of macroautophagy | 6 | 26 | 0.011737 | 0.249313 |
| BP | GO:0060193 | positive regulation of lipase activity | 6 | 26 | 0.011737 | 0.249313 |
| BP | GO:0051966 | regulation of synaptic transmission, glutamatergic | 5 | 19 | 0.011926 | 0.250128 |
| BP | GO:0016043 | cellular component organization | 280 | 3294 | 0.012003 | 0.250128 |
| CC | GO:0030016 | myofibril | 15 | 110 | 0.012018 | 0.113191 |
| CC | GO:0098797 | plasma membrane protein complex | 35 | 328 | 0.012064 | 0.113191 |
| MF | GO:0031267 | small GTPase binding | 22 | 178 | 0.012089 | 0.217552 |
| BP | GO:0071805 | potassium ion transmembrane transport | 9 | 50 | 0.012114 | 0.250128 |
| BP | GO:0030953 | astral microtubule organization | 3 | 7 | 0.012176 | 0.250128 |
| BP | GO:0043619 | regulation of transcription from RNA polymerase II promoter in response to oxidative stress | 3 | 7 | 0.012176 | 0.250128 |
| BP | GO:0048149 | behavioral response to ethanol | 3 | 7 | 0.012176 | 0.250128 |
| BP | GO:0051968 | positive regulation of synaptic transmission, glutamatergic | 3 | 7 | 0.012176 | 0.250128 |
| BP | GO:0090091 | positive regulation of extracellular matrix disassembly | 3 | 7 | 0.012176 | 0.250128 |
| BP | GO:2000270 | negative regulation of fibroblast apoptotic process | 3 | 7 | 0.012176 | 0.250128 |
| BP | GO:2000392 | regulation of lamellipodium morphogenesis | 3 | 7 | 0.012176 | 0.250128 |
| BP | GO:0007416 | synapse assembly | 14 | 95 | 0.012354 | 0.252962 |
| MF | GO:0005158 | insulin receptor binding | 4 | 13 | 0.012421 | 0.217552 |
| MF | GO:0051139 | metal cation:proton antiporter activity | 4 | 13 | 0.012421 | 0.217552 |
| BP | GO:0010557 | positive regulation of macromolecule biosynthetic process | 78 | 800 | 0.012532 | 0.254154 |
| BP | GO:0051130 | positive regulation of cellular component organization | 57 | 557 | 0.012610 | 0.254154 |
| BP | GO:0000271 | polysaccharide biosynthetic process | 8 | 42 | 0.012616 | 0.254154 |
| BP | GO:0043266 | regulation of potassium ion transport | 8 | 42 | 0.012616 | 0.254154 |
| BP | GO:0050848 | regulation of calcium-mediated signaling | 8 | 42 | 0.012616 | 0.254154 |
| CC | GO:0005622 | intracellular anatomical structure | 671 | 8919 | 0.012767 | 0.118327 |
| BP | GO:0044085 | cellular component biogenesis | 150 | 1668 | 0.012872 | 0.258467 |
| MF | GO:0015079 | potassium ion transmembrane transporter activity | 15 | 108 | 0.013118 | 0.226524 |
| MF | GO:0044325 | transmembrane transporter binding | 10 | 61 | 0.013398 | 0.227915 |
| BP | GO:0034762 | regulation of transmembrane transport | 33 | 291 | 0.013432 | 0.268854 |
| BP | GO:0019722 | calcium-mediated signaling | 14 | 96 | 0.013486 | 0.269073 |
| BP | GO:0042063 | gliogenesis | 21 | 165 | 0.013595 | 0.269129 |
| BP | GO:0090314 | positive regulation of protein targeting to membrane | 4 | 13 | 0.013649 | 0.269129 |
| BP | GO:1902902 | negative regulation of autophagosome assembly | 4 | 13 | 0.013649 | 0.269129 |
| BP | GO:0031325 | positive regulation of cellular metabolic process | 128 | 1402 | 0.013687 | 0.269129 |
| BP | GO:0014033 | neural crest cell differentiation | 9 | 51 | 0.013747 | 0.269129 |
| BP | GO:0035304 | regulation of protein dephosphorylation | 9 | 51 | 0.013747 | 0.269129 |
| CC | GO:0030018 | Z disc | 9 | 54 | 0.014005 | 0.127138 |
| BP | GO:0045664 | regulation of neuron differentiation | 15 | 106 | 0.014031 | 0.273818 |
| CC | GO:0005925 | focal adhesion | 15 | 112 | 0.014052 | 0.127138 |
| BP | GO:0010799 | regulation of peptidyl-threonine phosphorylation | 6 | 27 | 0.014143 | 0.274792 |
| BP | GO:0050877 | nervous system process | 56 | 549 | 0.014169 | 0.274792 |
| BP | GO:0006810 | transport | 204 | 2344 | 0.014351 | 0.277469 |
| BP | GO:0021885 | forebrain cell migration | 8 | 43 | 0.014497 | 0.278294 |
| BP | GO:1904892 | regulation of receptor signaling pathway via STAT | 8 | 43 | 0.014497 | 0.278294 |
| BP | GO:0006357 | regulation of transcription by RNA polymerase II | 79 | 817 | 0.014527 | 0.278294 |
| BP | GO:0051056 | regulation of small GTPase mediated signal transduction | 17 | 126 | 0.014622 | 0.279256 |
| BP | GO:0007611 | learning or memory | 19 | 146 | 0.014723 | 0.280312 |
| CC | GO:0005958 | DNA-dependent protein kinase-DNA ligase 4 complex | 2 | 3 | 0.014744 | 0.131649 |
| BP | GO:0060191 | regulation of lipase activity | 7 | 35 | 0.014767 | 0.280312 |
| MF | GO:0003774 | cytoskeletal motor activity | 9 | 53 | 0.014860 | 0.227915 |
| CC | GO:0015630 | microtubule cytoskeleton | 73 | 790 | 0.014897 | 0.131649 |
| BP | GO:0038084 | vascular endothelial growth factor signaling pathway | 5 | 20 | 0.014927 | 0.281624 |
| BP | GO:0051497 | negative regulation of stress fiber assembly | 5 | 20 | 0.014927 | 0.281624 |
| BP | GO:0007267 | cell-cell signaling | 76 | 783 | 0.015055 | 0.283186 |
| MF | GO:0061630 | ubiquitin protein ligase activity | 18 | 140 | 0.015070 | 0.227915 |
| CC | GO:0005829 | cytosol | 178 | 2137 | 0.015233 | 0.133066 |
| BP | GO:0035265 | organ growth | 13 | 88 | 0.015282 | 0.286251 |
| BP | GO:0033043 | regulation of organelle organization | 63 | 632 | 0.015342 | 0.286251 |
| BP | GO:0048513 | animal organ development | 175 | 1986 | 0.015355 | 0.286251 |
| CC | GO:0044304 | main axon | 6 | 29 | 0.015440 | 0.133341 |
| BP | GO:0060341 | regulation of cellular localization | 52 | 506 | 0.015514 | 0.288342 |
| MF | GO:0000992 | RNA polymerase III cis-regulatory region sequence-specific DNA binding | 2 | 3 | 0.015616 | 0.227915 |
| MF | GO:0001006 | RNA polymerase III type 3 promoter sequence-specific DNA binding | 2 | 3 | 0.015616 | 0.227915 |
| MF | GO:0004065 | arylsulfatase activity | 2 | 3 | 0.015616 | 0.227915 |
| MF | GO:0038085 | vascular endothelial growth factor binding | 2 | 3 | 0.015616 | 0.227915 |
| MF | GO:0043734 | DNA-N1-methyladenine dioxygenase activity | 2 | 3 | 0.015616 | 0.227915 |
| MF | GO:0047696 | beta-adrenergic receptor kinase activity | 2 | 3 | 0.015616 | 0.227915 |
| MF | GO:0052658 | inositol-1,4,5-trisphosphate 5-phosphatase activity | 2 | 3 | 0.015616 | 0.227915 |
| MF | GO:0052659 | inositol-1,3,4,5-tetrakisphosphate 5-phosphatase activity | 2 | 3 | 0.015616 | 0.227915 |
| MF | GO:0098879 | structural constituent of postsynaptic specialization | 2 | 3 | 0.015616 | 0.227915 |
| MF | GO:0098919 | structural constituent of postsynaptic density | 2 | 3 | 0.015616 | 0.227915 |
| CC | GO:0005902 | microvillus | 8 | 46 | 0.015670 | 0.133811 |
| BP | GO:0010811 | positive regulation of cell-substrate adhesion | 12 | 79 | 0.015741 | 0.289343 |
| MF | GO:1901363 | heterocyclic compound binding | 309 | 3782 | 0.015919 | 0.229605 |
| BP | GO:0040008 | regulation of growth | 35 | 317 | 0.016297 | 0.289343 |
| BP | GO:0016199 | axon midline choice point recognition | 2 | 3 | 0.016472 | 0.289343 |
| BP | GO:0032962 | positive regulation of inositol trisphosphate biosynthetic process | 2 | 3 | 0.016472 | 0.289343 |
| BP | GO:0035511 | oxidative DNA demethylation | 2 | 3 | 0.016472 | 0.289343 |
| BP | GO:0035553 | oxidative single-stranded RNA demethylation | 2 | 3 | 0.016472 | 0.289343 |
| BP | GO:0036022 | limb joint morphogenesis | 2 | 3 | 0.016472 | 0.289343 |
| BP | GO:0036091 | positive regulation of transcription from RNA polymerase II promoter in response to oxidative stress | 2 | 3 | 0.016472 | 0.289343 |
| BP | GO:0038089 | positive regulation of cell migration by vascular endothelial growth factor signaling pathway | 2 | 3 | 0.016472 | 0.289343 |
| BP | GO:0044205 | 'de novo' UMP biosynthetic process | 2 | 3 | 0.016472 | 0.289343 |
| BP | GO:0071503 | response to heparin | 2 | 3 | 0.016472 | 0.289343 |
| BP | GO:0090038 | negative regulation of protein kinase C signaling | 2 | 3 | 0.016472 | 0.289343 |
| BP | GO:0097021 | lymphocyte migration into lymphoid organs | 2 | 3 | 0.016472 | 0.289343 |
| BP | GO:1901509 | regulation of endothelial tube morphogenesis | 2 | 3 | 0.016472 | 0.289343 |
| BP | GO:1901727 | positive regulation of histone deacetylase activity | 2 | 3 | 0.016472 | 0.289343 |
| BP | GO:1902226 | regulation of macrophage colony-stimulating factor signaling pathway | 2 | 3 | 0.016472 | 0.289343 |
| BP | GO:1903995 | regulation of non-membrane spanning protein tyrosine kinase activity | 2 | 3 | 0.016472 | 0.289343 |
| BP | GO:1990791 | dorsal root ganglion development | 2 | 3 | 0.016472 | 0.289343 |
| BP | GO:2000619 | negative regulation of histone H4-K16 acetylation | 2 | 3 | 0.016472 | 0.289343 |
| MF | GO:0003777 | microtubule motor activity | 5 | 21 | 0.016498 | 0.235199 |
| BP | GO:0007405 | neuroblast proliferation | 8 | 44 | 0.016573 | 0.289343 |
| BP | GO:0010631 | epithelial cell migration | 23 | 189 | 0.016740 | 0.289343 |
| BP | GO:0021955 | central nervous system neuron axonogenesis | 6 | 28 | 0.016873 | 0.289343 |
| BP | GO:0038202 | TORC1 signaling | 6 | 28 | 0.016873 | 0.289343 |
| BP | GO:0055021 | regulation of cardiac muscle tissue growth | 6 | 28 | 0.016873 | 0.289343 |
| BP | GO:0097120 | receptor localization to synapse | 6 | 28 | 0.016873 | 0.289343 |
| BP | GO:2001222 | regulation of neuron migration | 6 | 28 | 0.016873 | 0.289343 |
| BP | GO:0048514 | blood vessel morphogenesis | 39 | 362 | 0.016910 | 0.289343 |
| CC | GO:0030117 | membrane coat | 10 | 65 | 0.016963 | 0.141671 |
| CC | GO:0048475 | coated membrane | 10 | 65 | 0.016963 | 0.141671 |
| MF | GO:0008157 | protein phosphatase 1 binding | 3 | 8 | 0.017076 | 0.240641 |
| BP | GO:0007422 | peripheral nervous system development | 7 | 36 | 0.017163 | 0.289343 |
| BP | GO:0033692 | cellular polysaccharide biosynthetic process | 7 | 36 | 0.017163 | 0.289343 |
| BP | GO:1904356 | regulation of telomere maintenance via telomere lengthening | 7 | 36 | 0.017163 | 0.289343 |
| BP | GO:0051049 | regulation of transport | 86 | 907 | 0.017575 | 0.289343 |
| BP | GO:0090132 | epithelium migration | 23 | 190 | 0.017728 | 0.289343 |
| BP | GO:0044344 | cellular response to fibroblast growth factor stimulus | 10 | 62 | 0.017799 | 0.289343 |
| BP | GO:0055007 | cardiac muscle cell differentiation | 10 | 62 | 0.017799 | 0.289343 |
| BP | GO:0062013 | positive regulation of small molecule metabolic process | 10 | 62 | 0.017799 | 0.289343 |
| BP | GO:0071774 | response to fibroblast growth factor | 10 | 62 | 0.017799 | 0.289343 |
| BP | GO:0001764 | neuron migration | 15 | 109 | 0.017802 | 0.289343 |
| MF | GO:0008201 | heparin binding | 11 | 73 | 0.017859 | 0.248810 |
| BP | GO:0009967 | positive regulation of signal transduction | 78 | 813 | 0.017969 | 0.289343 |
| BP | GO:0003323 | type B pancreatic cell development | 4 | 14 | 0.017979 | 0.289343 |
| BP | GO:0007097 | nuclear migration | 4 | 14 | 0.017979 | 0.289343 |
| BP | GO:0036003 | positive regulation of transcription from RNA polymerase II promoter in response to stress | 4 | 14 | 0.017979 | 0.289343 |
| BP | GO:0046621 | negative regulation of organ growth | 4 | 14 | 0.017979 | 0.289343 |
| BP | GO:0060292 | long-term synaptic depression | 4 | 14 | 0.017979 | 0.289343 |
| BP | GO:0070977 | bone maturation | 4 | 14 | 0.017979 | 0.289343 |
| BP | GO:0099560 | synaptic membrane adhesion | 4 | 14 | 0.017979 | 0.289343 |
| BP | GO:0009966 | regulation of signal transduction | 147 | 1648 | 0.017990 | 0.289343 |
| BP | GO:0031668 | cellular response to extracellular stimulus | 16 | 119 | 0.018049 | 0.289343 |
| CC | GO:0030017 | sarcomere | 13 | 95 | 0.018088 | 0.146810 |
| BP | GO:0070848 | response to growth factor | 41 | 386 | 0.018106 | 0.289343 |
| BP | GO:0048736 | appendage development | 17 | 129 | 0.018139 | 0.289343 |
| BP | GO:0060173 | limb development | 17 | 129 | 0.018139 | 0.289343 |
| CC | GO:0005891 | voltage-gated calcium channel complex | 6 | 30 | 0.018158 | 0.146810 |
| CC | GO:0030119 | AP-type membrane coat adaptor complex | 6 | 30 | 0.018158 | 0.146810 |
| BP | GO:0043087 | regulation of GTPase activity | 30 | 265 | 0.018221 | 0.289343 |
| BP | GO:1902533 | positive regulation of intracellular signal transduction | 50 | 488 | 0.018316 | 0.289343 |
| BP | GO:0010819 | regulation of T cell chemotaxis | 3 | 8 | 0.018391 | 0.289343 |
| BP | GO:0032836 | glomerular basement membrane development | 3 | 8 | 0.018391 | 0.289343 |
| BP | GO:0042359 | vitamin D metabolic process | 3 | 8 | 0.018391 | 0.289343 |
| BP | GO:0045161 | neuronal ion channel clustering | 3 | 8 | 0.018391 | 0.289343 |
| BP | GO:0097091 | synaptic vesicle clustering | 3 | 8 | 0.018391 | 0.289343 |
| BP | GO:0010464 | regulation of mesenchymal cell proliferation | 5 | 21 | 0.018394 | 0.289343 |
| BP | GO:0032232 | negative regulation of actin filament bundle assembly | 5 | 21 | 0.018394 | 0.289343 |
| BP | GO:0042311 | vasodilation | 5 | 21 | 0.018394 | 0.289343 |
| BP | GO:2000403 | positive regulation of lymphocyte migration | 5 | 21 | 0.018394 | 0.289343 |
| CC | GO:0043292 | contractile fiber | 15 | 116 | 0.018911 | 0.149597 |
| CC | GO:0034703 | cation channel complex | 17 | 137 | 0.018981 | 0.149597 |
| CC | GO:0033017 | sarcoplasmic reticulum membrane | 4 | 15 | 0.019093 | 0.149597 |
| BP | GO:0010638 | positive regulation of organelle organization | 31 | 277 | 0.019229 | 0.300848 |
| BP | GO:0032956 | regulation of actin cytoskeleton organization | 24 | 202 | 0.019280 | 0.300848 |
| BP | GO:0050678 | regulation of epithelial cell proliferation | 24 | 202 | 0.019280 | 0.300848 |
| MF | GO:0019902 | phosphatase binding | 14 | 103 | 0.019297 | 0.265815 |
| BP | GO:0007169 | transmembrane receptor protein tyrosine kinase signaling pathway | 36 | 332 | 0.019336 | 0.300848 |
| BP | GO:0010647 | positive regulation of cell communication | 85 | 899 | 0.019409 | 0.300848 |
| BP | GO:0048522 | positive regulation of cellular process | 246 | 2893 | 0.019617 | 0.300848 |
| BP | GO:0034219 | carbohydrate transmembrane transport | 9 | 54 | 0.019624 | 0.300848 |
| MF | GO:0019903 | protein phosphatase binding | 11 | 74 | 0.019624 | 0.267324 |
| CC | GO:0030286 | dynein complex | 7 | 39 | 0.019673 | 0.151066 |
| CC | GO:0005938 | cell cortex | 16 | 127 | 0.019678 | 0.151066 |
| BP | GO:0016311 | dephosphorylation | 21 | 171 | 0.019685 | 0.300848 |
| BP | GO:2000278 | regulation of DNA biosynthetic process | 10 | 63 | 0.019765 | 0.300848 |
| BP | GO:0032835 | glomerulus development | 7 | 37 | 0.019821 | 0.300848 |
| BP | GO:0071229 | cellular response to acid chemical | 7 | 37 | 0.019821 | 0.300848 |
| BP | GO:0008333 | endosome to lysosome transport | 6 | 29 | 0.019944 | 0.300848 |
| BP | GO:0033173 | calcineurin-NFAT signaling cascade | 6 | 29 | 0.019944 | 0.300848 |
| BP | GO:0042551 | neuron maturation | 6 | 29 | 0.019944 | 0.300848 |
| BP | GO:0060038 | cardiac muscle cell proliferation | 6 | 29 | 0.019944 | 0.300848 |
| BP | GO:0060420 | regulation of heart growth | 6 | 29 | 0.019944 | 0.300848 |
| BP | GO:1901016 | regulation of potassium ion transmembrane transporter activity | 6 | 29 | 0.019944 | 0.300848 |
| CC | GO:0005884 | actin filament | 8 | 48 | 0.019981 | 0.151859 |
| MF | GO:0004112 | cyclic-nucleotide phosphodiesterase activity | 5 | 22 | 0.020083 | 0.267511 |
| MF | GO:0004114 | 3',5'-cyclic-nucleotide phosphodiesterase activity | 5 | 22 | 0.020083 | 0.267511 |
| BP | GO:0001525 | angiogenesis | 33 | 300 | 0.020236 | 0.304520 |
| CC | GO:0030055 | cell-substrate junction | 15 | 117 | 0.020305 | 0.152787 |
| BP | GO:0031669 | cellular response to nutrient levels | 14 | 101 | 0.020388 | 0.305729 |
| MF | GO:0097159 | organic cyclic compound binding | 310 | 3813 | 0.020391 | 0.267511 |
| BP | GO:0071496 | cellular response to external stimulus | 19 | 151 | 0.020415 | 0.305729 |
| BP | GO:0031929 | TOR signaling | 12 | 82 | 0.020700 | 0.306802 |
| BP | GO:1990138 | neuron projection extension | 12 | 82 | 0.020700 | 0.306802 |
| MF | GO:0001784 | phosphotyrosine residue binding | 6 | 30 | 0.020709 | 0.267511 |
| BP | GO:0035107 | appendage morphogenesis | 15 | 111 | 0.020717 | 0.306802 |
| BP | GO:0035108 | limb morphogenesis | 15 | 111 | 0.020717 | 0.306802 |
| MF | GO:0005267 | potassium channel activity | 13 | 94 | 0.020729 | 0.267511 |
| BP | GO:0050804 | modulation of chemical synaptic transmission | 25 | 214 | 0.020781 | 0.306802 |
| BP | GO:0099177 | regulation of trans-synaptic signaling | 25 | 214 | 0.020781 | 0.306802 |
| BP | GO:0002274 | myeloid leukocyte activation | 16 | 121 | 0.020849 | 0.307086 |
| BP | GO:0023056 | positive regulation of signaling | 85 | 902 | 0.020928 | 0.307523 |
| MF | GO:0003707 | nuclear steroid receptor activity | 4 | 15 | 0.021067 | 0.269038 |
| BP | GO:0009887 | animal organ morphogenesis | 62 | 631 | 0.021189 | 0.310627 |
| BP | GO:0045666 | positive regulation of neuron differentiation | 8 | 46 | 0.021354 | 0.311783 |
| BP | GO:0034614 | cellular response to reactive oxygen species | 11 | 73 | 0.021425 | 0.311783 |
| BP | GO:0055067 | monovalent inorganic cation homeostasis | 11 | 73 | 0.021425 | 0.311783 |
| BP | GO:0042692 | muscle cell differentiation | 24 | 204 | 0.021468 | 0.311783 |
| BP | GO:0022604 | regulation of cell morphogenesis | 19 | 152 | 0.021733 | 0.314899 |
| BP | GO:0030316 | osteoclast differentiation | 10 | 64 | 0.021886 | 0.315650 |
| BP | GO:0072080 | nephron tubule development | 10 | 64 | 0.021886 | 0.315650 |
| BP | GO:0051246 | regulation of protein metabolic process | 114 | 1254 | 0.022091 | 0.317876 |
| BP | GO:0003254 | regulation of membrane depolarization | 5 | 22 | 0.022352 | 0.317970 |
| BP | GO:0009112 | nucleobase metabolic process | 5 | 22 | 0.022352 | 0.317970 |
| BP | GO:0032743 | positive regulation of interleukin-2 production | 5 | 22 | 0.022352 | 0.317970 |
| BP | GO:0070884 | regulation of calcineurin-NFAT signaling cascade | 5 | 22 | 0.022352 | 0.317970 |
| BP | GO:1904358 | positive regulation of telomere maintenance via telomere lengthening | 5 | 22 | 0.022352 | 0.317970 |
| BP | GO:0006793 | phosphorus metabolic process | 124 | 1377 | 0.022539 | 0.319848 |
| BP | GO:0033135 | regulation of peptidyl-serine phosphorylation | 12 | 83 | 0.022587 | 0.319848 |
| BP | GO:1901379 | regulation of potassium ion transmembrane transport | 7 | 38 | 0.022753 | 0.321257 |
| BP | GO:0043269 | regulation of ion transport | 36 | 336 | 0.022789 | 0.321257 |
| BP | GO:1902531 | regulation of intracellular signal transduction | 83 | 882 | 0.023039 | 0.322342 |
| BP | GO:0006775 | fat-soluble vitamin metabolic process | 4 | 15 | 0.023072 | 0.322342 |
| BP | GO:0043586 | tongue development | 4 | 15 | 0.023072 | 0.322342 |
| BP | GO:0048384 | retinoic acid receptor signaling pathway | 4 | 15 | 0.023072 | 0.322342 |
| BP | GO:0000302 | response to reactive oxygen species | 13 | 93 | 0.023348 | 0.322946 |
| BP | GO:0048729 | tissue morphogenesis | 42 | 404 | 0.023368 | 0.322946 |
| BP | GO:0090130 | tissue migration | 23 | 195 | 0.023372 | 0.322946 |
| BP | GO:0060976 | coronary vasculature development | 6 | 30 | 0.023374 | 0.322946 |
| BP | GO:0097720 | calcineurin-mediated signaling | 6 | 30 | 0.023374 | 0.322946 |
| MF | GO:0015297 | antiporter activity | 8 | 48 | 0.023394 | 0.295681 |
| BP | GO:0050770 | regulation of axonogenesis | 11 | 74 | 0.023500 | 0.323261 |
| BP | GO:0072009 | nephron epithelium development | 11 | 74 | 0.023500 | 0.323261 |
| BP | GO:0010033 | response to organic substance | 132 | 1478 | 0.023869 | 0.326668 |
| BP | GO:0050679 | positive regulation of epithelial cell proliferation | 15 | 113 | 0.023980 | 0.326668 |
| CC | GO:0030125 | clathrin vesicle coat | 4 | 16 | 0.024042 | 0.179137 |
| BP | GO:0010507 | negative regulation of autophagy | 8 | 47 | 0.024077 | 0.326668 |
| BP | GO:0050764 | regulation of phagocytosis | 8 | 47 | 0.024077 | 0.326668 |
| BP | GO:0060675 | ureteric bud morphogenesis | 8 | 47 | 0.024077 | 0.326668 |
| BP | GO:1902117 | positive regulation of organelle assembly | 8 | 47 | 0.024077 | 0.326668 |
| BP | GO:0002761 | regulation of myeloid leukocyte differentiation | 10 | 65 | 0.024167 | 0.326668 |
| BP | GO:0060993 | kidney morphogenesis | 10 | 65 | 0.024167 | 0.326668 |
| MF | GO:0098632 | cell-cell adhesion mediator activity | 3 | 9 | 0.024225 | 0.299999 |
| MF | GO:0106019 | phosphatidylinositol-4,5-bisphosphate phosphatase activity | 3 | 9 | 0.024225 | 0.299999 |
| BP | GO:0050793 | regulation of developmental process | 125 | 1393 | 0.024303 | 0.327800 |
| BP | GO:0055085 | transmembrane transport | 51 | 508 | 0.024363 | 0.327899 |
| BP | GO:0048675 | axon extension | 9 | 56 | 0.024434 | 0.328156 |
| BP | GO:0007167 | enzyme-linked receptor protein signaling pathway | 54 | 543 | 0.024634 | 0.330123 |
| CC | GO:0098858 | actin-based cell projection | 15 | 120 | 0.024954 | 0.183244 |
| CC | GO:0008076 | voltage-gated potassium channel complex | 8 | 50 | 0.025075 | 0.183244 |
| MF | GO:0003713 | transcription coactivator activity | 18 | 148 | 0.025196 | 0.308907 |
| BP | GO:0042594 | response to starvation | 13 | 94 | 0.025282 | 0.334735 |
| BP | GO:0044262 | cellular carbohydrate metabolic process | 20 | 165 | 0.025465 | 0.334735 |
| BP | GO:0050890 | cognition | 20 | 165 | 0.025465 | 0.334735 |
| BP | GO:0002062 | chondrocyte differentiation | 11 | 75 | 0.025719 | 0.334735 |
| BP | GO:0007259 | receptor signaling pathway via JAK-STAT | 11 | 75 | 0.025719 | 0.334735 |
| BP | GO:0007166 | cell surface receptor signaling pathway | 128 | 1433 | 0.025875 | 0.334735 |
| BP | GO:0006278 | RNA-templated DNA biosynthetic process | 7 | 39 | 0.025971 | 0.334735 |
| BP | GO:0007004 | telomere maintenance via telomerase | 7 | 39 | 0.025971 | 0.334735 |
| BP | GO:0055013 | cardiac muscle cell development | 7 | 39 | 0.025971 | 0.334735 |
| BP | GO:0000460 | maturation of 5.8S rRNA | 3 | 9 | 0.026050 | 0.334735 |
| BP | GO:0003159 | morphogenesis of an endothelium | 3 | 9 | 0.026050 | 0.334735 |
| BP | GO:0019430 | removal of superoxide radicals | 3 | 9 | 0.026050 | 0.334735 |
| BP | GO:0036005 | response to macrophage colony-stimulating factor | 3 | 9 | 0.026050 | 0.334735 |
| BP | GO:0036006 | cellular response to macrophage colony-stimulating factor stimulus | 3 | 9 | 0.026050 | 0.334735 |
| BP | GO:0061154 | endothelial tube morphogenesis | 3 | 9 | 0.026050 | 0.334735 |
| BP | GO:0070886 | positive regulation of calcineurin-NFAT signaling cascade | 3 | 9 | 0.026050 | 0.334735 |
| BP | GO:0071379 | cellular response to prostaglandin stimulus | 3 | 9 | 0.026050 | 0.334735 |
| BP | GO:0072182 | regulation of nephron tubule epithelial cell differentiation | 3 | 9 | 0.026050 | 0.334735 |
| BP | GO:0106058 | positive regulation of calcineurin-mediated signaling | 3 | 9 | 0.026050 | 0.334735 |
| BP | GO:1902931 | negative regulation of alcohol biosynthetic process | 3 | 9 | 0.026050 | 0.334735 |
| MF | GO:0022843 | voltage-gated cation channel activity | 13 | 97 | 0.026206 | 0.311249 |
| MF | GO:0005451 | monovalent cation:proton antiporter activity | 4 | 16 | 0.026482 | 0.311249 |
| MF | GO:0010857 | calcium-dependent protein kinase activity | 4 | 16 | 0.026482 | 0.311249 |
| MF | GO:0015299 | solute:proton antiporter activity | 4 | 16 | 0.026482 | 0.311249 |
| BP | GO:0005976 | polysaccharide metabolic process | 10 | 66 | 0.026614 | 0.340138 |
| BP | GO:0006796 | phosphate-containing compound metabolic process | 122 | 1361 | 0.026716 | 0.340138 |
| BP | GO:0032970 | regulation of actin filament-based process | 25 | 219 | 0.026764 | 0.340138 |
| BP | GO:0006383 | transcription by RNA polymerase III | 5 | 23 | 0.026823 | 0.340138 |
| BP | GO:0106056 | regulation of calcineurin-mediated signaling | 5 | 23 | 0.026823 | 0.340138 |
| CC | GO:1990351 | transporter complex | 26 | 243 | 0.026875 | 0.191376 |
| MF | GO:0019900 | kinase binding | 42 | 419 | 0.026901 | 0.311249 |
| BP | GO:0071363 | cellular response to growth factor stimulus | 39 | 374 | 0.026978 | 0.340138 |
| BP | GO:0010833 | telomere maintenance via telomere lengthening | 8 | 48 | 0.027036 | 0.340138 |
| BP | GO:0072171 | mesonephric tubule morphogenesis | 8 | 48 | 0.027036 | 0.340138 |
| BP | GO:0097581 | lamellipodium organization | 9 | 57 | 0.027130 | 0.340138 |
| BP | GO:0002067 | glandular epithelial cell differentiation | 6 | 31 | 0.027178 | 0.340138 |
| BP | GO:0032210 | regulation of telomere maintenance via telomerase | 6 | 31 | 0.027178 | 0.340138 |
| BP | GO:0051055 | negative regulation of lipid biosynthetic process | 6 | 31 | 0.027178 | 0.340138 |
| BP | GO:0051057 | positive regulation of small GTPase mediated signal transduction | 6 | 31 | 0.027178 | 0.340138 |
| BP | GO:0071897 | DNA biosynthetic process | 13 | 95 | 0.027332 | 0.340691 |
| BP | GO:1905475 | regulation of protein localization to membrane | 13 | 95 | 0.027332 | 0.340691 |
| BP | GO:0006996 | organelle organization | 166 | 1908 | 0.028003 | 0.346130 |
| BP | GO:0035051 | cardiocyte differentiation | 11 | 76 | 0.028085 | 0.346130 |
| BP | GO:0051147 | regulation of muscle cell differentiation | 11 | 76 | 0.028085 | 0.346130 |
| CC | GO:0031588 | nucleotide-activated protein kinase complex | 2 | 4 | 0.028086 | 0.191376 |
| CC | GO:0033186 | CAF-1 complex | 2 | 4 | 0.028086 | 0.191376 |
| CC | GO:0071439 | clathrin complex | 2 | 4 | 0.028086 | 0.191376 |
| CC | GO:0097255 | R2TP complex | 2 | 4 | 0.028086 | 0.191376 |
| CC | GO:1990531 | phospholipid-translocating ATPase complex | 2 | 4 | 0.028086 | 0.191376 |
| CC | GO:0032991 | protein-containing complex | 247 | 3095 | 0.028130 | 0.191376 |
| BP | GO:0007017 | microtubule-based process | 55 | 559 | 0.028173 | 0.346130 |
| CC | GO:0099080 | supramolecular complex | 58 | 628 | 0.028203 | 0.191376 |
| BP | GO:0006897 | endocytosis | 27 | 242 | 0.028512 | 0.346130 |
| BP | GO:0009891 | positive regulation of biosynthetic process | 81 | 867 | 0.028650 | 0.346130 |
| CC | GO:0016328 | lateral plasma membrane | 7 | 42 | 0.028665 | 0.191843 |
| BP | GO:0003309 | type B pancreatic cell differentiation | 4 | 16 | 0.028956 | 0.346130 |
| BP | GO:0007608 | sensory perception of smell | 4 | 16 | 0.028956 | 0.346130 |
| BP | GO:0035767 | endothelial cell chemotaxis | 4 | 16 | 0.028956 | 0.346130 |
| BP | GO:0046427 | positive regulation of receptor signaling pathway via JAK-STAT | 4 | 16 | 0.028956 | 0.346130 |
| BP | GO:0050850 | positive regulation of calcium-mediated signaling | 4 | 16 | 0.028956 | 0.346130 |
| BP | GO:0051894 | positive regulation of focal adhesion assembly | 4 | 16 | 0.028956 | 0.346130 |
| BP | GO:0032006 | regulation of TOR signaling | 10 | 67 | 0.029233 | 0.346130 |
| BP | GO:0061326 | renal tubule development | 10 | 67 | 0.029233 | 0.346130 |
| MF | GO:0000976 | transcription cis-regulatory region binding | 51 | 528 | 0.029471 | 0.311249 |
| BP | GO:0001658 | branching involved in ureteric bud morphogenesis | 7 | 40 | 0.029486 | 0.346130 |
| BP | GO:0055006 | cardiac cell development | 7 | 40 | 0.029486 | 0.346130 |
| CC | GO:0043226 | organelle | 606 | 8058 | 0.029594 | 0.191843 |
| BP | GO:1901698 | response to nitrogen compound | 45 | 445 | 0.029651 | 0.346130 |
| BP | GO:0035303 | regulation of dephosphorylation | 14 | 106 | 0.029665 | 0.346130 |
| MF | GO:0001016 | RNA polymerase III transcription regulatory region sequence-specific DNA binding | 2 | 4 | 0.029703 | 0.311249 |
| MF | GO:0004445 | inositol-polyphosphate 5-phosphatase activity | 2 | 4 | 0.029703 | 0.311249 |
| MF | GO:0004784 | superoxide dismutase activity | 2 | 4 | 0.029703 | 0.311249 |
| MF | GO:0016721 | oxidoreductase activity, acting on superoxide radicals as acceptor | 2 | 4 | 0.029703 | 0.311249 |
| MF | GO:0032050 | clathrin heavy chain binding | 2 | 4 | 0.029703 | 0.311249 |
| MF | GO:0038064 | collagen receptor activity | 2 | 4 | 0.029703 | 0.311249 |
| MF | GO:0046030 | inositol trisphosphate phosphatase activity | 2 | 4 | 0.029703 | 0.311249 |
| MF | GO:0052743 | inositol tetrakisphosphate phosphatase activity | 2 | 4 | 0.029703 | 0.311249 |
| MF | GO:0052812 | phosphatidylinositol-3,4-bisphosphate 5-kinase activity | 2 | 4 | 0.029703 | 0.311249 |
| MF | GO:0097677 | STAT family protein binding | 2 | 4 | 0.029703 | 0.311249 |
| MF | GO:0140444 | cytoskeleton-nuclear membrane anchor activity | 2 | 4 | 0.029703 | 0.311249 |
| BP | GO:0051129 | negative regulation of cellular component organization | 40 | 388 | 0.029740 | 0.346130 |
| CC | GO:0070382 | exocytic vesicle | 10 | 71 | 0.029791 | 0.191843 |
| BP | GO:0046849 | bone remodeling | 9 | 58 | 0.030029 | 0.346130 |
| BP | GO:0071840 | cellular component organization or biogenesis | 283 | 3395 | 0.030125 | 0.346130 |
| BP | GO:0006885 | regulation of pH | 8 | 49 | 0.030237 | 0.346130 |
| BP | GO:0033555 | multicellular organismal response to stress | 8 | 49 | 0.030237 | 0.346130 |
| CC | GO:0005766 | primary lysosome | 3 | 10 | 0.030318 | 0.191843 |
| CC | GO:0030897 | HOPS complex | 3 | 10 | 0.030318 | 0.191843 |
| CC | GO:0031527 | filopodium membrane | 3 | 10 | 0.030318 | 0.191843 |
| CC | GO:0031941 | filamentous actin | 3 | 10 | 0.030318 | 0.191843 |
| CC | GO:0042582 | azurophil granule | 3 | 10 | 0.030318 | 0.191843 |
| MF | GO:0001067 | transcription regulatory region nucleic acid binding | 51 | 529 | 0.030371 | 0.315548 |
| CC | GO:0070161 | anchoring junction | 38 | 387 | 0.030543 | 0.191843 |
| BP | GO:0035270 | endocrine system development | 11 | 77 | 0.030604 | 0.346130 |
| BP | GO:0050921 | positive regulation of chemotaxis | 11 | 77 | 0.030604 | 0.346130 |
| BP | GO:0097696 | receptor signaling pathway via STAT | 11 | 77 | 0.030604 | 0.346130 |
| BP | GO:0010639 | negative regulation of organelle organization | 24 | 211 | 0.030674 | 0.346130 |
| BP | GO:0061138 | morphogenesis of a branching epithelium | 17 | 137 | 0.030705 | 0.346130 |
| BP | GO:0062012 | regulation of small molecule metabolic process | 21 | 179 | 0.030872 | 0.346130 |
| BP | GO:0022603 | regulation of anatomical structure morphogenesis | 50 | 504 | 0.030953 | 0.346130 |
| CC | GO:0005654 | nucleoplasm | 198 | 2447 | 0.031108 | 0.193791 |
| BP | GO:0010957 | negative regulation of vitamin D biosynthetic process | 2 | 4 | 0.031287 | 0.346130 |
| BP | GO:0021873 | forebrain neuroblast division | 2 | 4 | 0.031287 | 0.346130 |
| BP | GO:0032769 | negative regulation of monooxygenase activity | 2 | 4 | 0.031287 | 0.346130 |
| BP | GO:0032926 | negative regulation of activin receptor signaling pathway | 2 | 4 | 0.031287 | 0.346130 |
| BP | GO:0032960 | regulation of inositol trisphosphate biosynthetic process | 2 | 4 | 0.031287 | 0.346130 |
| BP | GO:0035513 | oxidative RNA demethylation | 2 | 4 | 0.031287 | 0.346130 |
| BP | GO:0035740 | CD8-positive, alpha-beta T cell proliferation | 2 | 4 | 0.031287 | 0.346130 |
| BP | GO:0035766 | cell chemotaxis to fibroblast growth factor | 2 | 4 | 0.031287 | 0.346130 |
| BP | GO:0035768 | endothelial cell chemotaxis to fibroblast growth factor | 2 | 4 | 0.031287 | 0.346130 |
| BP | GO:0038145 | macrophage colony-stimulating factor signaling pathway | 2 | 4 | 0.031287 | 0.346130 |
| BP | GO:0042473 | outer ear morphogenesis | 2 | 4 | 0.031287 | 0.346130 |
| BP | GO:0045054 | constitutive secretory pathway | 2 | 4 | 0.031287 | 0.346130 |
| BP | GO:0046137 | negative regulation of vitamin metabolic process | 2 | 4 | 0.031287 | 0.346130 |
| BP | GO:0048387 | negative regulation of retinoic acid receptor signaling pathway | 2 | 4 | 0.031287 | 0.346130 |
| BP | GO:0048625 | myoblast fate commitment | 2 | 4 | 0.031287 | 0.346130 |
| BP | GO:0060287 | epithelial cilium movement involved in determination of left/right asymmetry | 2 | 4 | 0.031287 | 0.346130 |
| BP | GO:0060484 | lung-associated mesenchyme development | 2 | 4 | 0.031287 | 0.346130 |
| BP | GO:0070341 | fat cell proliferation | 2 | 4 | 0.031287 | 0.346130 |
| BP | GO:0070344 | regulation of fat cell proliferation | 2 | 4 | 0.031287 | 0.346130 |
| BP | GO:0070669 | response to interleukin-2 | 2 | 4 | 0.031287 | 0.346130 |
| BP | GO:0072103 | glomerulus vasculature morphogenesis | 2 | 4 | 0.031287 | 0.346130 |
| BP | GO:0072104 | glomerular capillary formation | 2 | 4 | 0.031287 | 0.346130 |
| BP | GO:0075044 | positive regulation by symbiont of host autophagy | 2 | 4 | 0.031287 | 0.346130 |
| BP | GO:0075071 | modulation by symbiont of host autophagy | 2 | 4 | 0.031287 | 0.346130 |
| BP | GO:0097241 | hematopoietic stem cell migration to bone marrow | 2 | 4 | 0.031287 | 0.346130 |
| BP | GO:0150093 | amyloid-beta clearance by transcytosis | 2 | 4 | 0.031287 | 0.346130 |
| BP | GO:1901725 | regulation of histone deacetylase activity | 2 | 4 | 0.031287 | 0.346130 |
| BP | GO:1902857 | positive regulation of non-motile cilium assembly | 2 | 4 | 0.031287 | 0.346130 |
| BP | GO:1904847 | regulation of cell chemotaxis to fibroblast growth factor | 2 | 4 | 0.031287 | 0.346130 |
| BP | GO:2000051 | negative regulation of non-canonical Wnt signaling pathway | 2 | 4 | 0.031287 | 0.346130 |
| BP | GO:2000172 | regulation of branching morphogenesis of a nerve | 2 | 4 | 0.031287 | 0.346130 |
| BP | GO:2000544 | regulation of endothelial cell chemotaxis to fibroblast growth factor | 2 | 4 | 0.031287 | 0.346130 |
| BP | GO:2000564 | regulation of CD8-positive, alpha-beta T cell proliferation | 2 | 4 | 0.031287 | 0.346130 |
| BP | GO:0002886 | regulation of myeloid leukocyte mediated immunity | 6 | 32 | 0.031371 | 0.346130 |
| BP | GO:0035924 | cellular response to vascular endothelial growth factor stimulus | 6 | 32 | 0.031371 | 0.346130 |
| BP | GO:0098742 | cell-cell adhesion via plasma-membrane adhesion molecules | 15 | 117 | 0.031639 | 0.347028 |
| BP | GO:0048583 | regulation of response to stimulus | 184 | 2141 | 0.031749 | 0.347028 |
| BP | GO:0002762 | negative regulation of myeloid leukocyte differentiation | 5 | 24 | 0.031826 | 0.347028 |
| BP | GO:0035176 | social behavior | 5 | 24 | 0.031826 | 0.347028 |
| BP | GO:0070849 | response to epidermal growth factor | 5 | 24 | 0.031826 | 0.347028 |
| BP | GO:0072583 | clathrin-dependent endocytosis | 5 | 24 | 0.031826 | 0.347028 |
| BP | GO:0051099 | positive regulation of binding | 14 | 107 | 0.031841 | 0.347028 |
| BP | GO:0030038 | contractile actin filament bundle assembly | 10 | 68 | 0.032030 | 0.347875 |
| BP | GO:0043149 | stress fiber assembly | 10 | 68 | 0.032030 | 0.347875 |
| MF | GO:0005096 | GTPase activator activity | 16 | 131 | 0.032360 | 0.326763 |
| BP | GO:0007389 | pattern specification process | 30 | 278 | 0.032535 | 0.352740 |
| MF | GO:0004693 | cyclin-dependent protein serine/threonine kinase activity | 3 | 10 | 0.032737 | 0.326763 |
| MF | GO:0015368 | calcium:cation antiporter activity | 3 | 10 | 0.032737 | 0.326763 |
| MF | GO:0097472 | cyclin-dependent protein kinase activity | 3 | 10 | 0.032737 | 0.326763 |
| MF | GO:0051020 | GTPase binding | 22 | 196 | 0.032783 | 0.326763 |
| BP | GO:0060326 | cell chemotaxis | 19 | 159 | 0.032865 | 0.355709 |
| CC | GO:0030135 | coated vesicle | 13 | 103 | 0.032900 | 0.203284 |
| MF | GO:0022836 | gated channel activity | 27 | 252 | 0.033157 | 0.327827 |
| BP | GO:0051493 | regulation of cytoskeleton organization | 32 | 301 | 0.033521 | 0.361620 |
| BP | GO:0034220 | ion transmembrane transport | 39 | 380 | 0.033527 | 0.361620 |
| BP | GO:0001938 | positive regulation of endothelial cell proliferation | 8 | 50 | 0.033689 | 0.362733 |
| CC | GO:0034702 | ion channel complex | 21 | 191 | 0.033742 | 0.206804 |
| MF | GO:0030276 | clathrin binding | 5 | 25 | 0.033746 | 0.330985 |
| BP | GO:0008284 | positive regulation of cell population proliferation | 50 | 507 | 0.033946 | 0.364393 |
| BP | GO:0009267 | cellular response to starvation | 12 | 88 | 0.033959 | 0.364393 |
| BP | GO:0030100 | regulation of endocytosis | 13 | 98 | 0.034208 | 0.366434 |
| MF | GO:0008047 | enzyme activator activity | 27 | 253 | 0.034614 | 0.336798 |
| BP | GO:0046907 | intracellular transport | 76 | 815 | 0.034625 | 0.366457 |
| CC | GO:0012505 | endomembrane system | 184 | 2269 | 0.034962 | 0.212571 |
| BP | GO:0098609 | cell-cell adhesion | 43 | 427 | 0.034971 | 0.366457 |
| BP | GO:0003279 | cardiac septum development | 10 | 69 | 0.035010 | 0.366457 |
| BP | GO:0008037 | cell recognition | 10 | 69 | 0.035010 | 0.366457 |
| BP | GO:0031503 | protein-containing complex localization | 10 | 69 | 0.035010 | 0.366457 |
| BP | GO:0042552 | myelination | 10 | 69 | 0.035010 | 0.366457 |
| BP | GO:0032695 | negative regulation of interleukin-12 production | 3 | 10 | 0.035149 | 0.366457 |
| BP | GO:0055015 | ventricular cardiac muscle cell development | 3 | 10 | 0.035149 | 0.366457 |
| BP | GO:0055057 | neuroblast division | 3 | 10 | 0.035149 | 0.366457 |
| BP | GO:0071450 | cellular response to oxygen radical | 3 | 10 | 0.035149 | 0.366457 |
| BP | GO:0071451 | cellular response to superoxide | 3 | 10 | 0.035149 | 0.366457 |
| BP | GO:0072160 | nephron tubule epithelial cell differentiation | 3 | 10 | 0.035149 | 0.366457 |
| BP | GO:0098869 | cellular oxidant detoxification | 3 | 10 | 0.035149 | 0.366457 |
| BP | GO:1902932 | positive regulation of alcohol biosynthetic process | 3 | 10 | 0.035149 | 0.366457 |
| BP | GO:1903649 | regulation of cytoplasmic transport | 3 | 10 | 0.035149 | 0.366457 |
| BP | GO:1904263 | positive regulation of TORC1 signaling | 3 | 10 | 0.035149 | 0.366457 |
| BP | GO:0007423 | sensory organ development | 38 | 370 | 0.035209 | 0.366466 |
| CC | GO:0031463 | Cul3-RING ubiquitin ligase complex | 5 | 26 | 0.035258 | 0.212669 |
| BP | GO:0002068 | glandular epithelial cell development | 4 | 17 | 0.035649 | 0.369202 |
| BP | GO:0035883 | enteroendocrine cell differentiation | 4 | 17 | 0.035649 | 0.369202 |
| BP | GO:2000406 | positive regulation of T cell migration | 4 | 17 | 0.035649 | 0.369202 |
| BP | GO:0010812 | negative regulation of cell-substrate adhesion | 6 | 33 | 0.035963 | 0.371219 |
| BP | GO:0048016 | inositol phosphate-mediated signaling | 6 | 33 | 0.035963 | 0.371219 |
| CC | GO:0034704 | calcium channel complex | 7 | 44 | 0.035999 | 0.213433 |
| CC | GO:0030131 | clathrin adaptor complex | 4 | 18 | 0.036070 | 0.213433 |
| BP | GO:0016197 | endosomal transport | 15 | 119 | 0.036078 | 0.371578 |
| BP | GO:0002040 | sprouting angiogenesis | 11 | 79 | 0.036117 | 0.371578 |
| BP | GO:1905114 | cell surface receptor signaling pathway involved in cell-cell signaling | 32 | 303 | 0.036277 | 0.372619 |
| CC | GO:0005634 | nucleus | 339 | 4363 | 0.036341 | 0.213433 |
| BP | GO:0044264 | cellular polysaccharide metabolic process | 9 | 60 | 0.036460 | 0.373879 |
| CC | GO:0001726 | ruffle | 12 | 94 | 0.036508 | 0.213433 |
| MF | GO:0030374 | nuclear receptor coactivator activity | 6 | 34 | 0.036539 | 0.352733 |
| BP | GO:0010634 | positive regulation of epithelial cell migration | 13 | 99 | 0.036754 | 0.376282 |
| BP | GO:0034765 | regulation of ion transmembrane transport | 27 | 248 | 0.037271 | 0.378405 |
| BP | GO:0048732 | gland development | 27 | 248 | 0.037271 | 0.378405 |
| BP | GO:0007099 | centriole replication | 5 | 25 | 0.037374 | 0.378405 |
| BP | GO:0021591 | ventricular system development | 5 | 25 | 0.037374 | 0.378405 |
| BP | GO:0030838 | positive regulation of actin filament polymerization | 5 | 25 | 0.037374 | 0.378405 |
| BP | GO:0031122 | cytoplasmic microtubule organization | 5 | 25 | 0.037374 | 0.378405 |
| BP | GO:0043255 | regulation of carbohydrate biosynthetic process | 8 | 51 | 0.037399 | 0.378405 |
| BP | GO:2000573 | positive regulation of DNA biosynthetic process | 7 | 42 | 0.037447 | 0.378405 |
| MF | GO:0005515 | protein binding | 336 | 4207 | 0.038352 | 0.364513 |
| BP | GO:0009792 | embryo development ending in birth or egg hatching | 48 | 488 | 0.038637 | 0.389801 |
| BP | GO:0010675 | regulation of cellular carbohydrate metabolic process | 11 | 80 | 0.039119 | 0.394032 |
| MF | GO:0030331 | nuclear estrogen receptor binding | 5 | 26 | 0.039322 | 0.364513 |
| MF | GO:0042169 | SH2 domain binding | 5 | 26 | 0.039322 | 0.364513 |
| BP | GO:0050731 | positive regulation of peptidyl-tyrosine phosphorylation | 13 | 100 | 0.039433 | 0.396470 |
| BP | GO:0030902 | hindbrain development | 12 | 90 | 0.039488 | 0.396470 |
| CC | GO:0000159 | protein phosphatase type 2A complex | 3 | 11 | 0.039507 | 0.225693 |
| CC | GO:0043240 | Fanconi anaemia nuclear complex | 3 | 11 | 0.039507 | 0.225693 |
| BP | GO:0019932 | second-messenger-mediated signaling | 16 | 131 | 0.040031 | 0.400627 |
| BP | GO:0051216 | cartilage development | 16 | 131 | 0.040031 | 0.400627 |
| CC | GO:0042734 | presynaptic membrane | 7 | 45 | 0.040090 | 0.225693 |
| CC | GO:0043197 | dendritic spine | 7 | 45 | 0.040090 | 0.225693 |
| CC | GO:0044291 | cell-cell contact zone | 7 | 45 | 0.040090 | 0.225693 |
| MF | GO:0046873 | metal ion transmembrane transporter activity | 30 | 291 | 0.040353 | 0.364513 |
| CC | GO:0005737 | cytoplasm | 493 | 6499 | 0.040462 | 0.226113 |
| BP | GO:0040007 | growth | 50 | 513 | 0.040603 | 0.405677 |
| BP | GO:0048646 | anatomical structure formation involved in morphogenesis | 67 | 714 | 0.040665 | 0.405677 |
| BP | GO:0002009 | morphogenesis of an epithelium | 35 | 340 | 0.040791 | 0.406285 |
| BP | GO:0051899 | membrane depolarization | 6 | 34 | 0.040965 | 0.406718 |
| BP | GO:0071230 | cellular response to amino acid stimulus | 6 | 34 | 0.040965 | 0.406718 |
| MF | GO:0000987 | cis-regulatory region sequence-specific DNA binding | 37 | 373 | 0.041247 | 0.364513 |
| BP | GO:0042147 | retrograde transport, endosome to Golgi | 8 | 52 | 0.041373 | 0.409470 |
| BP | GO:0072078 | nephron tubule morphogenesis | 8 | 52 | 0.041373 | 0.409470 |
| CC | GO:0030120 | vesicle coat | 6 | 36 | 0.041418 | 0.229178 |
| BP | GO:0007272 | ensheathment of neurons | 10 | 71 | 0.041537 | 0.409795 |
| BP | GO:0008366 | axon ensheathment | 10 | 71 | 0.041537 | 0.409795 |
| CC | GO:0034705 | potassium channel complex | 8 | 55 | 0.041614 | 0.229178 |
| BP | GO:0034637 | cellular carbohydrate biosynthetic process | 7 | 43 | 0.041909 | 0.412162 |
| BP | GO:0035023 | regulation of Rho protein signal transduction | 7 | 43 | 0.041909 | 0.412162 |
| CC | GO:0008021 | synaptic vesicle | 9 | 65 | 0.041922 | 0.229213 |
| BP | GO:0090596 | sensory organ morphogenesis | 21 | 185 | 0.041990 | 0.412303 |
| BP | GO:0048565 | digestive tract development | 11 | 81 | 0.042291 | 0.414511 |
| BP | GO:0016482 | cytosolic transport | 12 | 91 | 0.042477 | 0.414511 |
| BP | GO:0030534 | adult behavior | 12 | 91 | 0.042477 | 0.414511 |
| BP | GO:0045927 | positive regulation of growth | 16 | 132 | 0.042480 | 0.414511 |
| MF | GO:0015385 | sodium:proton antiporter activity | 3 | 11 | 0.042592 | 0.364513 |
| MF | GO:0052745 | inositol phosphate phosphatase activity | 3 | 11 | 0.042592 | 0.364513 |
| BP | GO:0048745 | smooth muscle tissue development | 4 | 18 | 0.043160 | 0.415374 |
| BP | GO:0051150 | regulation of smooth muscle cell differentiation | 4 | 18 | 0.043160 | 0.415374 |
| BP | GO:1904894 | positive regulation of receptor signaling pathway via STAT | 4 | 18 | 0.043160 | 0.415374 |
| BP | GO:0006366 | transcription by RNA polymerase II | 81 | 885 | 0.043414 | 0.415374 |
| BP | GO:0001755 | neural crest cell migration | 5 | 26 | 0.043478 | 0.415374 |
| BP | GO:0002066 | columnar/cuboidal epithelial cell development | 5 | 26 | 0.043478 | 0.415374 |
| BP | GO:0031018 | endocrine pancreas development | 5 | 26 | 0.043478 | 0.415374 |
| BP | GO:0051703 | biological process involved in intraspecies interaction between organisms | 5 | 26 | 0.043478 | 0.415374 |
| BP | GO:0098900 | regulation of action potential | 5 | 26 | 0.043478 | 0.415374 |
| BP | GO:2000785 | regulation of autophagosome assembly | 5 | 26 | 0.043478 | 0.415374 |
| CC | GO:0043227 | membrane-bounded organelle | 545 | 7235 | 0.044384 | 0.230582 |
| CC | GO:0032587 | ruffle membrane | 7 | 46 | 0.044473 | 0.230582 |
| CC | GO:0005916 | fascia adherens | 2 | 5 | 0.044599 | 0.230582 |
| CC | GO:0034774 | secretory granule lumen | 2 | 5 | 0.044599 | 0.230582 |
| CC | GO:0038037 | G protein-coupled receptor dimeric complex | 2 | 5 | 0.044599 | 0.230582 |
| CC | GO:0097648 | G protein-coupled receptor complex | 2 | 5 | 0.044599 | 0.230582 |
| CC | GO:0098831 | presynaptic active zone cytoplasmic component | 2 | 5 | 0.044599 | 0.230582 |
| CC | GO:1990909 | Wnt signalosome | 2 | 5 | 0.044599 | 0.230582 |
| BP | GO:0031328 | positive regulation of cellular biosynthetic process | 78 | 850 | 0.044632 | 0.415374 |
| BP | GO:0061024 | membrane organization | 36 | 354 | 0.044711 | 0.415374 |
| MF | GO:0000977 | RNA polymerase II transcription regulatory region sequence-specific DNA binding | 43 | 447 | 0.045377 | 0.364513 |
| CC | GO:0000323 | lytic vacuole | 28 | 279 | 0.045450 | 0.231827 |
| CC | GO:0005764 | lysosome | 28 | 279 | 0.045450 | 0.231827 |
| BP | GO:0002444 | myeloid leukocyte mediated immunity | 8 | 53 | 0.045618 | 0.415374 |
| BP | GO:0008645 | hexose transmembrane transport | 8 | 53 | 0.045618 | 0.415374 |
| BP | GO:0015749 | monosaccharide transmembrane transport | 8 | 53 | 0.045618 | 0.415374 |
| BP | GO:0042490 | mechanoreceptor differentiation | 8 | 53 | 0.045618 | 0.415374 |
| BP | GO:1904659 | glucose transmembrane transport | 8 | 53 | 0.045618 | 0.415374 |
| BP | GO:0002327 | immature B cell differentiation | 3 | 11 | 0.045661 | 0.415374 |
| BP | GO:0010818 | T cell chemotaxis | 3 | 11 | 0.045661 | 0.415374 |
| BP | GO:0010831 | positive regulation of myotube differentiation | 3 | 11 | 0.045661 | 0.415374 |
| BP | GO:0014046 | dopamine secretion | 3 | 11 | 0.045661 | 0.415374 |
| BP | GO:0014059 | regulation of dopamine secretion | 3 | 11 | 0.045661 | 0.415374 |
| BP | GO:0014911 | positive regulation of smooth muscle cell migration | 3 | 11 | 0.045661 | 0.415374 |
| BP | GO:0034694 | response to prostaglandin | 3 | 11 | 0.045661 | 0.415374 |
| BP | GO:0035791 | platelet-derived growth factor receptor-beta signaling pathway | 3 | 11 | 0.045661 | 0.415374 |
| BP | GO:0045056 | transcytosis | 3 | 11 | 0.045661 | 0.415374 |
| BP | GO:0046112 | nucleobase biosynthetic process | 3 | 11 | 0.045661 | 0.415374 |
| BP | GO:0050919 | negative chemotaxis | 3 | 11 | 0.045661 | 0.415374 |
| BP | GO:0051895 | negative regulation of focal adhesion assembly | 3 | 11 | 0.045661 | 0.415374 |
| BP | GO:0060413 | atrial septum morphogenesis | 3 | 11 | 0.045661 | 0.415374 |
| BP | GO:0061318 | renal filtration cell differentiation | 3 | 11 | 0.045661 | 0.415374 |
| BP | GO:0072112 | podocyte differentiation | 3 | 11 | 0.045661 | 0.415374 |
| BP | GO:0072311 | glomerular epithelial cell differentiation | 3 | 11 | 0.045661 | 0.415374 |
| BP | GO:0150118 | negative regulation of cell-substrate junction organization | 3 | 11 | 0.045661 | 0.415374 |
| BP | GO:1900273 | positive regulation of long-term synaptic potentiation | 3 | 11 | 0.045661 | 0.415374 |
| BP | GO:1903861 | positive regulation of dendrite extension | 3 | 11 | 0.045661 | 0.415374 |
| BP | GO:2000696 | regulation of epithelial cell differentiation involved in kidney development | 3 | 11 | 0.045661 | 0.415374 |
| BP | GO:2001026 | regulation of endothelial cell chemotaxis | 3 | 11 | 0.045661 | 0.415374 |
| BP | GO:0045785 | positive regulation of cell adhesion | 27 | 253 | 0.045998 | 0.415374 |
| BP | GO:0036211 | protein modification process | 147 | 1701 | 0.046153 | 0.415374 |
| BP | GO:0055017 | cardiac muscle tissue growth | 6 | 35 | 0.046385 | 0.415374 |
| BP | GO:0014032 | neural crest cell development | 7 | 44 | 0.046702 | 0.415374 |
| MF | GO:0000182 | rDNA binding | 2 | 5 | 0.047099 | 0.364513 |
| MF | GO:0004439 | phosphatidylinositol-4,5-bisphosphate 5-phosphatase activity | 2 | 5 | 0.047099 | 0.364513 |
| MF | GO:0004690 | cyclic nucleotide-dependent protein kinase activity | 2 | 5 | 0.047099 | 0.364513 |
| MF | GO:0008273 | calcium, potassium:sodium antiporter activity | 2 | 5 | 0.047099 | 0.364513 |
| MF | GO:0022821 | solute:potassium antiporter activity | 2 | 5 | 0.047099 | 0.364513 |
| MF | GO:0035515 | oxidative RNA demethylase activity | 2 | 5 | 0.047099 | 0.364513 |
| MF | GO:0035612 | AP-2 adaptor complex binding | 2 | 5 | 0.047099 | 0.364513 |
| MF | GO:0098634 | cell-matrix adhesion mediator activity | 2 | 5 | 0.047099 | 0.364513 |
| BP | GO:0051336 | regulation of hydrolase activity | 51 | 530 | 0.047196 | 0.415374 |
| BP | GO:0061448 | connective tissue development | 19 | 166 | 0.047762 | 0.415374 |
| BP | GO:0010243 | response to organonitrogen compound | 40 | 402 | 0.047769 | 0.415374 |
| BP | GO:0000226 | microtubule cytoskeleton organization | 38 | 379 | 0.047903 | 0.415374 |
| MF | GO:0030594 | neurotransmitter receptor activity | 8 | 55 | 0.048114 | 0.364513 |
| BP | GO:0071695 | anatomical structure maturation | 17 | 145 | 0.048798 | 0.415374 |
| CC | GO:0031981 | nuclear lumen | 216 | 2721 | 0.048799 | 0.245766 |
| BP | GO:0007605 | sensory perception of sound | 12 | 93 | 0.048919 | 0.415374 |
| CC | GO:0044309 | neuron spine | 7 | 47 | 0.049153 | 0.245766 |
| CC | GO:0098685 | Schaffer collateral - CA1 synapse | 7 | 47 | 0.049153 | 0.245766 |
| BP | GO:0000491 | small nucleolar ribonucleoprotein complex assembly | 2 | 5 | 0.049541 | 0.415374 |
| BP | GO:0006307 | DNA dealkylation involved in DNA repair | 2 | 5 | 0.049541 | 0.415374 |
| BP | GO:0007440 | foregut morphogenesis | 2 | 5 | 0.049541 | 0.415374 |
| BP | GO:0008063 | Toll signaling pathway | 2 | 5 | 0.049541 | 0.415374 |
| BP | GO:0009642 | response to light intensity | 2 | 5 | 0.049541 | 0.415374 |
| BP | GO:0016198 | axon choice point recognition | 2 | 5 | 0.049541 | 0.415374 |
| BP | GO:0022028 | tangential migration from the subventricular zone to the olfactory bulb | 2 | 5 | 0.049541 | 0.415374 |
| BP | GO:0034058 | endosomal vesicle fusion | 2 | 5 | 0.049541 | 0.415374 |
| BP | GO:0035860 | glial cell-derived neurotrophic factor receptor signaling pathway | 2 | 5 | 0.049541 | 0.415374 |
| BP | GO:0044340 | canonical Wnt signaling pathway involved in regulation of cell proliferation | 2 | 5 | 0.049541 | 0.415374 |
| BP | GO:0048617 | embryonic foregut morphogenesis | 2 | 5 | 0.049541 | 0.415374 |
| BP | GO:0050882 | voluntary musculoskeletal movement | 2 | 5 | 0.049541 | 0.415374 |
| BP | GO:0051901 | positive regulation of mitochondrial depolarization | 2 | 5 | 0.049541 | 0.415374 |
| BP | GO:0060087 | relaxation of vascular associated smooth muscle | 2 | 5 | 0.049541 | 0.415374 |
| BP | GO:0060161 | positive regulation of dopamine receptor signaling pathway | 2 | 5 | 0.049541 | 0.415374 |
| BP | GO:0060440 | trachea formation | 2 | 5 | 0.049541 | 0.415374 |
| BP | GO:0060558 | regulation of calcidiol 1-monooxygenase activity | 2 | 5 | 0.049541 | 0.415374 |
| BP | GO:0070940 | dephosphorylation of RNA polymerase II C-terminal domain | 2 | 5 | 0.049541 | 0.415374 |
| BP | GO:0070989 | oxidative demethylation | 2 | 5 | 0.049541 | 0.415374 |
| BP | GO:0071361 | cellular response to ethanol | 2 | 5 | 0.049541 | 0.415374 |
| BP | GO:0072102 | glomerulus morphogenesis | 2 | 5 | 0.049541 | 0.415374 |
| BP | GO:0090045 | positive regulation of deacetylase activity | 2 | 5 | 0.049541 | 0.415374 |
| BP | GO:0090241 | negative regulation of histone H4 acetylation | 2 | 5 | 0.049541 | 0.415374 |
| BP | GO:0099515 | actin filament-based transport | 2 | 5 | 0.049541 | 0.415374 |
| BP | GO:0150094 | amyloid-beta clearance by cellular catabolic process | 2 | 5 | 0.049541 | 0.415374 |
| BP | GO:1901838 | positive regulation of transcription of nucleolar large rRNA by RNA polymerase I | 2 | 5 | 0.049541 | 0.415374 |
| BP | GO:1902667 | regulation of axon guidance | 2 | 5 | 0.049541 | 0.415374 |
| BP | GO:1903054 | negative regulation of extracellular matrix organization | 2 | 5 | 0.049541 | 0.415374 |
| BP | GO:1903969 | regulation of response to macrophage colony-stimulating factor | 2 | 5 | 0.049541 | 0.415374 |
| BP | GO:1903972 | regulation of cellular response to macrophage colony-stimulating factor stimulus | 2 | 5 | 0.049541 | 0.415374 |
| BP | GO:1904181 | positive regulation of membrane depolarization | 2 | 5 | 0.049541 | 0.415374 |
| BP | GO:1905323 | telomerase holoenzyme complex assembly | 2 | 5 | 0.049541 | 0.415374 |
| BP | GO:1990253 | cellular response to leucine starvation | 2 | 5 | 0.049541 | 0.415374 |
| BP | GO:2000394 | positive regulation of lamellipodium morphogenesis | 2 | 5 | 0.049541 | 0.415374 |
| BP | GO:2000618 | regulation of histone H4-K16 acetylation | 2 | 5 | 0.049541 | 0.415374 |
| BP | GO:2000807 | regulation of synaptic vesicle clustering | 2 | 5 | 0.049541 | 0.415374 |
| BP | GO:0110053 | regulation of actin filament organization | 18 | 156 | 0.049629 | 0.415374 |
| MF | GO:0044877 | protein-containing complex binding | 55 | 595 | 0.049755 | 0.364513 |
| CC | GO:0032839 | dendrite cytoplasm | 3 | 12 | 0.049933 | 0.248032 |
| MF | GO:0019901 | protein kinase binding | 36 | 367 | 0.049961 | 0.364513 |

**Table S23 The gene annotation of the top 1% in XP-CLR analysis of Zhashijia sheep and Valley Tibetan sheep (partial results)**

| **Chr** | **Bin_Start** | **Bin_End** | **Xpclr_norm** | **Chr** | **Gene_Start** | **Gene_End** | **GeneID** |
| --- | --- | --- | --- | --- | --- | --- | --- |
| NC_056054.1 | 255420001 | 255425000 | 40.3967 | NC_056054.1 | 255417105 | 255497514 | gene-CEP63 |
| NC_056054.1 | 255420001 | 255425000 | 40.3967 | NC_056054.1 | 255417105 | 255497514 | gene-CEP63 |
| NC_056070.1 | 42510001 | 42515000 | 37.2811 | NC_056070.1 | 42429909 | 42672024 | gene-PDGFC |
| NC_056055.1 | 106055001 | 106060000 | 37.0701 | NC_056055.1 | 105968386 | 106151898 | gene-GLRA3 |
| NC_056068.1 | 9520001 | 9525000 | 37.0018 | NC_056068.1 | 8449512 | 10112326 | gene-CNTN5 |
| NC_056062.1 | 32220001 | 32225000 | 33.8857 | NC_056062.1 | 32215526 | 32220399 | gene-LOC105609193 |
| NC_056062.1 | 32220001 | 32225000 | 33.8857 | NC_056062.1 | 32142360 | 32427602 | gene-SPIDR |
| NC_056062.1 | 32195001 | 32200000 | 33.7928 | NC_056062.1 | 32142360 | 32427602 | gene-SPIDR |
| NC_056076.1 | 22590001 | 22595000 | 33.0388 | NC_056076.1 | 22491651 | 22797967 | gene-DTNA |
| NC_056060.1 | 74050001 | 74055000 | 32.9198 | NC_056060.1 | 74021041 | 74066080 | gene-SGPP1 |
| NC_056062.1 | 32205001 | 32210000 | 32.0602 | NC_056062.1 | 32142360 | 32427602 | gene-SPIDR |
| NC_056056.1 | 7515001 | 7520000 | 32.0388 | NC_056056.1 | 7496776 | 7524523 | gene-LRRC8A |
| NC_056070.1 | 42505001 | 42510000 | 31.3797 | NC_056070.1 | 42429909 | 42672024 | gene-PDGFC |
| NC_056062.1 | 32215001 | 32220000 | 30.6383 | NC_056062.1 | 32215526 | 32220399 | gene-LOC105609193 |
| NC_056062.1 | 32215001 | 32220000 | 30.6383 | NC_056062.1 | 32142360 | 32427602 | gene-SPIDR |
| NC_056056.1 | 148185001 | 148190000 | 30.1651 | NC_056056.1 | 148087108 | 148276060 | gene-KIF21A |
| NC_056068.1 | 9525001 | 9530000 | 28.9269 | NC_056068.1 | 8449512 | 10112326 | gene-CNTN5 |
| NC_056055.1 | 106065001 | 106070000 | 28.4752 | NC_056055.1 | 105968386 | 106151898 | gene-GLRA3 |
| NC_056054.1 | 255430001 | 255435000 | 28.3991 | NC_056054.1 | 255417105 | 255497514 | gene-CEP63 |
| NC_056054.1 | 255430001 | 255435000 | 28.3991 | NC_056054.1 | 255417105 | 255497514 | gene-CEP63 |
| NC_056075.1 | 34325001 | 34330000 | 28.3863 | NC_056075.1 | 34301218 | 34455397 | gene-AFAP1L2 |
| NC_056055.1 | 185515001 | 185520000 | 27.6025 | NC_056055.1 | 185494575 | 185578841 | gene-RALB |
| NC_056080.1 | 35665001 | 35670000 | 27.4690 | NC_056080.1 | 35310194 | 35818182 | gene-CFAP47 |
| NC_056071.1 | 55855001 | 55860000 | 27.0767 | NC_056071.1 | 55847463 | 56118296 | gene-UNC79 |
| NC_056064.1 | 14870001 | 14875000 | 26.7749 | NC_056064.1 | 14837230 | 14872460 | gene-CCT6B |
| NC_056055.1 | 185520001 | 185525000 | 26.4959 | NC_056055.1 | 185494575 | 185578841 | gene-RALB |
| NC_056054.1 | 42990001 | 42995000 | 26.3239 | NC_056054.1 | 42972532 | 43047850 | gene-IL12RB2 |
| NC_056054.1 | 42990001 | 42995000 | 26.3239 | NC_056054.1 | 42972532 | 43047850 | gene-IL12RB2 |
| NC_056055.1 | 185540001 | 185545000 | 25.7845 | NC_056055.1 | 185494575 | 185578841 | gene-RALB |
| NC_056073.1 | 835001 | 840000 | 25.6637 | NC_056073.1 | 255140 | 1051733 | gene-KHDRBS2 |
| NC_056076.1 | 22620001 | 22625000 | 25.6071 | NC_056076.1 | 22491651 | 22797967 | gene-DTNA |
| NC_056055.1 | 106045001 | 106050000 | 25.5100 | NC_056055.1 | 105968386 | 106151898 | gene-GLRA3 |
| NC_056054.1 | 42995001 | 43000000 | 25.3854 | NC_056054.1 | 42972532 | 43047850 | gene-IL12RB2 |
| NC_056054.1 | 42995001 | 43000000 | 25.3854 | NC_056054.1 | 42972532 | 43047850 | gene-IL12RB2 |
| NC_056054.1 | 255470001 | 255475000 | 25.3745 | NC_056054.1 | 255417105 | 255497514 | gene-CEP63 |
| NC_056054.1 | 255470001 | 255475000 | 25.3745 | NC_056054.1 | 255417105 | 255497514 | gene-CEP63 |
| NC_056071.1 | 55875001 | 55880000 | 25.3314 | NC_056071.1 | 55847463 | 56118296 | gene-UNC79 |
| NC_056055.1 | 185510001 | 185515000 | 25.2163 | NC_056055.1 | 185494575 | 185578841 | gene-RALB |
| NC_056060.1 | 74040001 | 74045000 | 25.0093 | NC_056060.1 | 74021041 | 74066080 | gene-SGPP1 |
| NC_056080.1 | 35655001 | 35660000 | 24.9166 | NC_056080.1 | 35658197 | 35658268 | gene-TRNAW-CCA-235 |
| NC_056080.1 | 35655001 | 35660000 | 24.9166 | NC_056080.1 | 35310194 | 35818182 | gene-CFAP47 |
| NC_056080.1 | 35680001 | 35685000 | 24.6330 | NC_056080.1 | 35310194 | 35818182 | gene-CFAP47 |
| NC_056079.1 | 2595001 | 2600000 | 24.6232 | NC_056079.1 | 2301792 | 4372358 | gene-CSMD1 |
| NC_056071.1 | 55845001 | 55850000 | 24.5083 | NC_056071.1 | 55847463 | 56118296 | gene-UNC79 |
| NC_056055.1 | 185555001 | 185560000 | 24.4761 | NC_056055.1 | 185494575 | 185578841 | gene-RALB |
| NC_056074.1 | 20110001 | 20115000 | 24.4199 | NC_056074.1 | 20051983 | 20149177 | gene-ANO5 |
| NC_056076.1 | 22615001 | 22620000 | 24.3902 | NC_056076.1 | 22491651 | 22797967 | gene-DTNA |
| NC_056064.1 | 14860001 | 14865000 | 24.2598 | NC_056064.1 | 14837230 | 14872460 | gene-CCT6B |
| NC_056080.1 | 35670001 | 35675000 | 24.1444 | NC_056080.1 | 35310194 | 35818182 | gene-CFAP47 |
| NC_056056.1 | 7510001 | 7515000 | 24.0782 | NC_056056.1 | 7496776 | 7524523 | gene-LRRC8A |
| NC_056070.1 | 42500001 | 42505000 | 23.8796 | NC_056070.1 | 42429909 | 42672024 | gene-PDGFC |
| NC_056055.1 | 185560001 | 185565000 | 23.8442 | NC_056055.1 | 185494575 | 185578841 | gene-RALB |
| NC_056055.1 | 185525001 | 185530000 | 23.7594 | NC_056055.1 | 185494575 | 185578841 | gene-RALB |
| NC_056077.1 | 8265001 | 8270000 | 22.9049 | NC_056077.1 | 8264478 | 8318849 | gene-USP7 |
| NC_056073.1 | 40645001 | 40650000 | 22.8443 | NC_056073.1 | 40577347 | 40676362 | gene-DTNBP1 |
| NC_056054.1 | 41720001 | 41725000 | 22.7921 | NC_056054.1 | 41378608 | 42043605 | gene-PDE4B |
| NC_056054.1 | 41720001 | 41725000 | 22.7921 | NC_056054.1 | 41378608 | 42043605 | gene-PDE4B |
| NC_056055.1 | 106080001 | 106085000 | 22.6139 | NC_056055.1 | 105968386 | 106151898 | gene-GLRA3 |
| NC_056074.1 | 20115001 | 20120000 | 22.5712 | NC_056074.1 | 20051983 | 20149177 | gene-ANO5 |
| NC_056054.1 | 43000001 | 43005000 | 22.5342 | NC_056054.1 | 42972532 | 43047850 | gene-IL12RB2 |
| NC_056054.1 | 43000001 | 43005000 | 22.5342 | NC_056054.1 | 42972532 | 43047850 | gene-IL12RB2 |
| NC_056056.1 | 102195001 | 102200000 | 22.5284 | NC_056056.1 | 102181754 | 102271425 | gene-TSGA10 |
| NC_056055.1 | 185575001 | 185580000 | 22.3772 | NC_056055.1 | 185494575 | 185578841 | gene-RALB |
| NC_056061.1 | 57030001 | 57035000 | 22.1795 | NC_056061.1 | 57008754 | 57051204 | gene-MED23 |
| NC_056059.1 | 112285001 | 112290000 | 22.0654 | NC_056059.1 | 112281233 | 112298463 | gene-LOC114115351 |
| NC_056054.1 | 255475001 | 255480000 | 22.0237 | NC_056054.1 | 255417105 | 255497514 | gene-CEP63 |
| NC_056054.1 | 255475001 | 255480000 | 22.0237 | NC_056054.1 | 255417105 | 255497514 | gene-CEP63 |
| NC_056062.1 | 32190001 | 32195000 | 21.9891 | NC_056062.1 | 32190866 | 32193644 | gene-LOC121820334 |
| NC_056062.1 | 32190001 | 32195000 | 21.9891 | NC_056062.1 | 32142360 | 32427602 | gene-SPIDR |
| NC_056059.1 | 60150001 | 60155000 | 21.6959 | NC_056059.1 | 60111604 | 60281254 | gene-RBM47 |
| NC_056079.1 | 2605001 | 2610000 | 21.6574 | NC_056079.1 | 2301792 | 4372358 | gene-CSMD1 |
| NC_056073.1 | 840001 | 845000 | 21.6480 | NC_056073.1 | 255140 | 1051733 | gene-KHDRBS2 |
| NC_056065.1 | 4155001 | 4160000 | 21.5514 | NC_056065.1 | 4109545 | 4183922 | gene-RASSF5 |
| NC_056068.1 | 9530001 | 9535000 | 21.4787 | NC_056068.1 | 8449512 | 10112326 | gene-CNTN5 |
| NC_056055.1 | 185550001 | 185555000 | 21.2454 | NC_056055.1 | 185494575 | 185578841 | gene-RALB |
| NC_056058.1 | 105525001 | 105530000 | 21.1388 | NC_056058.1 | 105314306 | 105770967 | gene-FER |
| NC_056076.1 | 22595001 | 22600000 | 21.1293 | NC_056076.1 | 22491651 | 22797967 | gene-DTNA |
| NC_056074.1 | 20105001 | 20110000 | 21.0984 | NC_056074.1 | 20051983 | 20149177 | gene-ANO5 |
| NC_056056.1 | 102185001 | 102190000 | 21.0735 | NC_056056.1 | 102181754 | 102271425 | gene-TSGA10 |
| NC_056073.1 | 40640001 | 40645000 | 20.6205 | NC_056073.1 | 40577347 | 40676362 | gene-DTNBP1 |
| NC_056058.1 | 5910001 | 5915000 | 20.4199 | NC_056058.1 | 5851824 | 5951495 | gene-CPAMD8 |
| NC_056068.1 | 80770001 | 80775000 | 20.3381 | NC_056068.1 | 80764032 | 80792678 | gene-LOC101108419 |
| NC_056058.1 | 100295001 | 100300000 | 20.3050 | NC_056058.1 | 100159308 | 100302559 | gene-LOC105609312 |
| NC_056059.1 | 56345001 | 56350000 | 20.1985 | NC_056059.1 | 56203465 | 56401728 | gene-ARAP2 |
| NC_056069.1 | 16380001 | 16385000 | 20.1931 | NC_056069.1 | 16294391 | 16759680 | gene-LOC106991659 |
| NC_056075.1 | 34290001 | 34295000 | 20.1723 | NC_056075.1 | 34248267 | 34300333 | gene-VWA2 |
| NC_056079.1 | 3280001 | 3285000 | 20.1310 | NC_056079.1 | 2301792 | 4372358 | gene-CSMD1 |
| NC_056066.1 | 66145001 | 66150000 | 20.1224 | NC_056066.1 | 66129495 | 66179624 | gene-RPN2 |
| NC_056058.1 | 6060001 | 6065000 | 20.0071 | NC_056058.1 | 6015791 | 6100901 | gene-NWD1 |
| NC_056069.1 | 16375001 | 16380000 | 19.9612 | NC_056069.1 | 16294391 | 16759680 | gene-LOC106991659 |
| NC_056076.1 | 22515001 | 22520000 | 19.8604 | NC_056076.1 | 22491651 | 22797967 | gene-DTNA |
| NC_056054.1 | 251210001 | 251215000 | 19.8066 | NC_056054.1 | 251160455 | 251258285 | gene-CEP70 |
| NC_056054.1 | 251210001 | 251215000 | 19.8066 | NC_056054.1 | 251160455 | 251258285 | gene-CEP70 |
| NC_056079.1 | 2615001 | 2620000 | 19.7831 | NC_056079.1 | 2301792 | 4372358 | gene-CSMD1 |
| NC_056056.1 | 7500001 | 7505000 | 19.7501 | NC_056056.1 | 7496776 | 7524523 | gene-LRRC8A |
| NC_056071.1 | 55850001 | 55855000 | 19.7155 | NC_056071.1 | 55847463 | 56118296 | gene-UNC79 |
| NC_056073.1 | 36285001 | 36290000 | 19.5533 | NC_056073.1 | 36099534 | 36706718 | gene-CDKAL1 |
| NC_056071.1 | 55870001 | 55875000 | 19.5493 | NC_056071.1 | 55847463 | 56118296 | gene-UNC79 |
| NC_056054.1 | 255460001 | 255465000 | 19.5059 | NC_056054.1 | 255417105 | 255497514 | gene-CEP63 |
| NC_056054.1 | 255460001 | 255465000 | 19.5059 | NC_056054.1 | 255417105 | 255497514 | gene-CEP63 |
| NC_056058.1 | 6070001 | 6075000 | 19.4763 | NC_056058.1 | 6015791 | 6100901 | gene-NWD1 |
| NC_056055.1 | 185570001 | 185575000 | 19.4401 | NC_056055.1 | 185494575 | 185578841 | gene-RALB |
| NC_056072.1 | 21975001 | 21980000 | 19.3895 | NC_056072.1 | 21889742 | 21988671 | gene-SUMF1 |
| NC_056073.1 | 830001 | 835000 | 19.3453 | NC_056073.1 | 255140 | 1051733 | gene-KHDRBS2 |
| NC_056054.1 | 255480001 | 255485000 | 19.2882 | NC_056054.1 | 255417105 | 255497514 | gene-CEP63 |
| NC_056054.1 | 255480001 | 255485000 | 19.2882 | NC_056054.1 | 255417105 | 255497514 | gene-CEP63 |
| NC_056070.1 | 42025001 | 42030000 | 19.1320 | NC_056070.1 | 41966872 | 42173672 | gene-GRIA2 |
| NC_056055.1 | 106100001 | 106105000 | 19.1301 | NC_056055.1 | 105968386 | 106151898 | gene-GLRA3 |
| NC_056055.1 | 248160001 | 248165000 | 19.1157 | NC_056055.1 | 247946881 | 248220389 | gene-IGSF21 |
| NC_056058.1 | 5905001 | 5910000 | 19.0208 | NC_056058.1 | 5851824 | 5951495 | gene-CPAMD8 |
| NC_056072.1 | 39310001 | 39315000 | 18.8712 | NC_056072.1 | 39155807 | 39930537 | gene-PTPRG |
| NC_056057.1 | 20125001 | 20130000 | 18.8176 | NC_056057.1 | 19934983 | 20129951 | gene-PHF14 |
| NC_056063.1 | 2120001 | 2125000 | 18.7008 | NC_056063.1 | 1894291 | 2124851 | gene-TDRD3 |
| NC_056054.1 | 20550001 | 20555000 | 18.5943 | NC_056054.1 | 20410748 | 20610364 | gene-MAST2 |
| NC_056054.1 | 20550001 | 20555000 | 18.5943 | NC_056054.1 | 20410748 | 20610364 | gene-MAST2 |
| NC_056055.1 | 224720001 | 224725000 | 18.5703 | NC_056055.1 | 224588986 | 224744099 | gene-SGPP2 |
| NC_056055.1 | 185505001 | 185510000 | 18.5634 | NC_056055.1 | 185494575 | 185578841 | gene-RALB |
| NC_056064.1 | 43575001 | 43580000 | 18.5014 | NC_056064.1 | 43564740 | 43581362 | gene-PYY |
| NC_056080.1 | 35685001 | 35690000 | 18.3450 | NC_056080.1 | 35310194 | 35818182 | gene-CFAP47 |
| NC_056080.1 | 35690001 | 35695000 | 18.3117 | NC_056080.1 | 35310194 | 35818182 | gene-CFAP47 |
| NC_056064.1 | 61555001 | 61560000 | 18.2836 | NC_056064.1 | 61472298 | 61785295 | gene-CEP112 |
| NC_056062.1 | 32290001 | 32295000 | 18.2742 | NC_056062.1 | 32142360 | 32427602 | gene-SPIDR |
| NC_056080.1 | 63485001 | 63490000 | 18.2430 | NC_056080.1 | 63398916 | 63648746 | gene-TEX11 |
| NC_056080.1 | 35660001 | 35665000 | 18.1726 | NC_056080.1 | 35310194 | 35818182 | gene-CFAP47 |
| NC_056058.1 | 6055001 | 6060000 | 18.1572 | NC_056058.1 | 6015791 | 6100901 | gene-NWD1 |
| NC_056077.1 | 8310001 | 8315000 | 18.1152 | NC_056077.1 | 8264478 | 8318849 | gene-USP7 |
| NC_056056.1 | 111050001 | 111055000 | 18.0753 | NC_056056.1 | 110997361 | 111234603 | gene-KCNC2 |
| NC_056054.1 | 31545001 | 31550000 | 17.9901 | NC_056054.1 | 31534318 | 31579387 | gene-C8B |
| NC_056054.1 | 31545001 | 31550000 | 17.9901 | NC_056054.1 | 31534318 | 31579387 | gene-C8B |
| NC_056056.1 | 213700001 | 213705000 | 17.9650 | NC_056056.1 | 213613677 | 213885548 | gene-ERC1 |
| NC_056057.1 | 20120001 | 20125000 | 17.9223 | NC_056057.1 | 19934983 | 20129951 | gene-PHF14 |
| NC_056070.1 | 42035001 | 42040000 | 17.8367 | NC_056070.1 | 41966872 | 42173672 | gene-GRIA2 |
| NC_056056.1 | 102200001 | 102205000 | 17.8251 | NC_056056.1 | 102181754 | 102271425 | gene-TSGA10 |
| NC_056075.1 | 34330001 | 34335000 | 17.8230 | NC_056075.1 | 34301218 | 34455397 | gene-AFAP1L2 |
| NC_056066.1 | 52390001 | 52395000 | 17.7596 | NC_056066.1 | 52393632 | 52402253 | gene-SNRPB |
| NC_056055.1 | 124985001 | 124990000 | 17.6613 | NC_056055.1 | 124682478 | 125033650 | gene-ZNF804A |
| NC_056055.1 | 106090001 | 106095000 | 17.5798 | NC_056055.1 | 105968386 | 106151898 | gene-GLRA3 |
| NC_056078.1 | 44545001 | 44550000 | 17.4772 | NC_056078.1 | 44542689 | 44549502 | gene-LOC121817989 |
| NC_056057.1 | 108460001 | 108465000 | 17.4743 | NC_056057.1 | 108461642 | 108462571 | gene-LOC101117664 |
| NC_056062.1 | 32295001 | 32300000 | 17.4492 | NC_056062.1 | 32142360 | 32427602 | gene-SPIDR |
| NC_056061.1 | 10795001 | 10800000 | 17.4316 | NC_056061.1 | 10751422 | 10925749 | gene-SNAP91 |
| NC_056054.1 | 255450001 | 255455000 | 17.4141 | NC_056054.1 | 255417105 | 255497514 | gene-CEP63 |
| NC_056054.1 | 255450001 | 255455000 | 17.4141 | NC_056054.1 | 255417105 | 255497514 | gene-CEP63 |
| NC_056055.1 | 97205001 | 97210000 | 17.3621 | NC_056055.1 | 96720020 | 98236892 | gene-LINGO2 |
| NC_056066.1 | 66105001 | 66110000 | 17.3497 | NC_056066.1 | 66071333 | 66130020 | gene-MROH8 |
| NC_056055.1 | 97245001 | 97250000 | 17.3354 | NC_056055.1 | 96720020 | 98236892 | gene-LINGO2 |
| NC_056060.1 | 74035001 | 74040000 | 17.3129 | NC_056060.1 | 74021041 | 74066080 | gene-SGPP1 |
| NC_056075.1 | 34295001 | 34300000 | 17.3007 | NC_056075.1 | 34248267 | 34300333 | gene-VWA2 |
| NC_056054.1 | 80335001 | 80340000 | 17.2953 | NC_056054.1 | 80145966 | 80371232 | gene-COL11A1 |
| NC_056054.1 | 80335001 | 80340000 | 17.2953 | NC_056054.1 | 80145966 | 80371232 | gene-COL11A1 |
| NC_056078.1 | 23265001 | 23270000 | 17.2605 | NC_056078.1 | 21570751 | 23431511 | gene-CTNNA3 |
| NC_056056.1 | 7495001 | 7500000 | 17.1514 | NC_056056.1 | 7496776 | 7524523 | gene-LRRC8A |
| NC_056054.1 | 43025001 | 43030000 | 17.0491 | NC_056054.1 | 42972532 | 43047850 | gene-IL12RB2 |
| NC_056054.1 | 43025001 | 43030000 | 17.0491 | NC_056054.1 | 42972532 | 43047850 | gene-IL12RB2 |
| NC_056064.1 | 43600001 | 43605000 | 17.0485 | NC_056064.1 | 43604205 | 43609893 | gene-NAGS |
| NC_056064.1 | 48895001 | 48900000 | 17.0060 | NC_056064.1 | 48896125 | 48896206 | gene-TRNAS-AGA-13 |
| NC_056064.1 | 48895001 | 48900000 | 17.0060 | NC_056064.1 | 48876863 | 48895373 | gene-NOL11 |
| NC_056055.1 | 233690001 | 233695000 | 16.9047 | NC_056055.1 | 233611612 | 233969343 | gene-DIS3L2 |
| NC_056058.1 | 100280001 | 100285000 | 16.8989 | NC_056058.1 | 100159308 | 100302559 | gene-LOC105609312 |
| NC_056055.1 | 60705001 | 60710000 | 16.8538 | NC_056055.1 | 60409880 | 60917448 | gene-PCSK5 |
| NC_056059.1 | 42010001 | 42015000 | 16.8496 | NC_056059.1 | 40863346 | 42176341 | gene-KCNIP4 |
| NC_056058.1 | 5895001 | 5900000 | 16.8439 | NC_056058.1 | 5851824 | 5951495 | gene-CPAMD8 |
| NC_056054.1 | 64970001 | 64975000 | 16.8238 | NC_056054.1 | 64970488 | 64970560 | gene-TRNAE-CUC |
| NC_056054.1 | 64970001 | 64975000 | 16.8238 | NC_056054.1 | 64970488 | 64970560 | gene-TRNAE-CUC |
| NC_056055.1 | 85055001 | 85060000 | 16.7871 | NC_056055.1 | 84866968 | 85352850 | gene-BNC2 |
| NC_056076.1 | 31010001 | 31015000 | 16.7416 | NC_056076.1 | 31006065 | 31047214 | gene-PSMA8 |
| NC_056076.1 | 31015001 | 31020000 | 16.7416 | NC_056076.1 | 31006065 | 31047214 | gene-PSMA8 |
| NC_056080.1 | 63480001 | 63485000 | 16.6945 | NC_056080.1 | 63398916 | 63648746 | gene-TEX11 |
| NC_056055.1 | 137760001 | 137765000 | 16.6382 | NC_056055.1 | 137718805 | 137792368 | gene-METAP1D |
| NC_056072.1 | 51155001 | 51160000 | 16.6161 | NC_056072.1 | 51134151 | 51199846 | gene-PRKAR2A |
| NC_056070.1 | 11675001 | 11680000 | 16.5735 | NC_056070.1 | 11419230 | 11691776 | gene-TTC29 |
| NC_056054.1 | 2570001 | 2575000 | 16.5445 | NC_056054.1 | 2523962 | 2576804 | gene-TWIST2 |
| NC_056054.1 | 2570001 | 2575000 | 16.5445 | NC_056054.1 | 2523962 | 2576804 | gene-TWIST2 |
| NC_056055.1 | 248170001 | 248175000 | 16.4882 | NC_056055.1 | 247946881 | 248220389 | gene-IGSF21 |
| NC_056061.1 | 40240001 | 40245000 | 16.4579 | NC_056061.1 | 40215116 | 40287472 | gene-GPR63 |
| NC_056068.1 | 79215001 | 79220000 | 16.4267 | NC_056068.1 | 79207797 | 79220375 | gene-LRRC55 |
| NC_056056.1 | 7520001 | 7525000 | 16.3201 | NC_056056.1 | 7496776 | 7524523 | gene-LRRC8A |
| NC_056056.1 | 7520001 | 7525000 | 16.3201 | NC_056056.1 | 7523230 | 7564553 | gene-LOC114108697 |
| NC_056075.1 | 4520001 | 4525000 | 16.2227 | NC_056075.1 | 4266126 | 5354887 | gene-PCDH15 |
| NC_056064.1 | 50455001 | 50460000 | 16.2090 | NC_056064.1 | 50453280 | 50455598 | gene-MYADML2 |
| NC_056064.1 | 50455001 | 50460000 | 16.2090 | NC_056064.1 | 50458160 | 50462833 | gene-PYCR1 |
| NC_056071.1 | 55900001 | 55905000 | 16.2058 | NC_056071.1 | 55847463 | 56118296 | gene-UNC79 |
| NC_056079.1 | 40400001 | 40405000 | 16.1950 | NC_056079.1 | 40093676 | 40404887 | gene-LOC121818046 |
| NC_056055.1 | 248155001 | 248160000 | 16.1642 | NC_056055.1 | 247946881 | 248220389 | gene-IGSF21 |
| NC_056068.1 | 79210001 | 79215000 | 16.1351 | NC_056068.1 | 79207797 | 79220375 | gene-LRRC55 |
| NC_056066.1 | 7585001 | 7590000 | 16.1135 | NC_056066.1 | 7380310 | 9704465 | gene-MACROD2 |
| NC_056065.1 | 76285001 | 76290000 | 16.0961 | NC_056065.1 | 76272823 | 76298296 | gene-LHX9 |
| NC_056080.1 | 35600001 | 35605000 | 16.0924 | NC_056080.1 | 35310194 | 35818182 | gene-CFAP47 |
| NC_056070.1 | 63800001 | 63805000 | 16.0846 | NC_056070.1 | 63793175 | 63840664 | gene-UBE3B |
| NC_056054.1 | 31540001 | 31545000 | 16.0780 | NC_056054.1 | 31534318 | 31579387 | gene-C8B |
| NC_056054.1 | 31540001 | 31545000 | 16.0780 | NC_056054.1 | 31534318 | 31579387 | gene-C8B |
| NC_056054.1 | 96925001 | 96930000 | 16.0621 | NC_056054.1 | 96910817 | 96940722 | gene-HAO2 |
| NC_056054.1 | 96925001 | 96930000 | 16.0621 | NC_056054.1 | 96910817 | 96940722 | gene-HAO2 |
| NC_056064.1 | 14835001 | 14840000 | 16.0222 | NC_056064.1 | 14835767 | 14837200 | gene-ZNF830 |
| NC_056064.1 | 14835001 | 14840000 | 16.0222 | NC_056064.1 | 14837230 | 14872460 | gene-CCT6B |
| NC_056054.1 | 31515001 | 31520000 | 16.0057 | NC_056054.1 | 31451090 | 31533275 | gene-C8A |
| NC_056054.1 | 31515001 | 31520000 | 16.0057 | NC_056054.1 | 31451090 | 31533275 | gene-C8A |
| NC_056054.1 | 43010001 | 43015000 | 15.9841 | NC_056054.1 | 42972532 | 43047850 | gene-IL12RB2 |
| NC_056054.1 | 43010001 | 43015000 | 15.9841 | NC_056054.1 | 42972532 | 43047850 | gene-IL12RB2 |
| NC_056073.1 | 19160001 | 19165000 | 15.9703 | NC_056073.1 | 19139291 | 19305418 | gene-CLIC5 |

**Table S24** The key genes associated with altitude adaptation in selected regions

| **Gene ID** | **Gene name** | **Function** |
| --- | --- | --- |
| gene-IL12RB2 | interleukin-12 receptor subunit beta-2 precursor | Regulates immune function (Morris et al., 2018). |
| gene-CEP63 | centrosomal protein of 63 kDa isoform X1 | Regulates cell cycle and cell division (Peng et al., 2017). |
| gene-GLRA3 | glycine receptor subunit alpha-3 isoform X1 | Regulates neurotransmitters (Lin and Xie,, 2019). |
| gene-RALB | ras-related protein Ral-B isoform X1 | Regulates cell proliferation, differentiation and migration (Minato., 2013). |
| gene-LRRC8A | volume-regulated anion channel subunit LRRC8A | Regulates cell volume, cell PH, and ion balance (Chen et al., 2023). |
| gene-FER | tyrosine-protein kinase Fer isoform X1 | Regulates cell adhesion, migration, and invasion (Hinz et al., 2017). |
| gene-SGPP1 | sphingosine-1-phosphate phosphatase 1 | Regulates cell proliferation, apoptosis, and migration (He et al., 2020). |
| gene-SPIDR | DNA repair-scaffolding protein isoform X1 | DNA repair (Tropitzsch et al., 2019). |
| gene-CNTN5 | contactin-5 | Plays an important role in neuronal development (Eve et al., 2022). |
| gene-PDGFC | platelet-derived growth factor C isoform X1 | It is an important mediator of angiogenesis (Gilbertson et al., 2001). |
| gene-UNC79 | protein unc-79 homolog isoform X1 | Adjusts the circadian rhythm (Lear et al., 2013). |
| gene-AFAPIL2 | actin filament-associated protein 1-like 2 isoform X1 | Regulates cell proliferation, migration, and cytoskeleton reorganization (Sun et al., 2023). |
| gene-DTNA | dystrobrevin alpha isoform X1 | Regulates muscle development (Malakootian et al., 2022). |
| gene-CEAP47 | cilia- and flagella-associated protein 47 | Plays a role in the formation and functional play of cilia and flagella, participates in cell motility, signaling, perception (Liu et al. 2021, Li et al., 2022). |

**Table S25 The KEGG enrichment analysis of genes in selective sweep regions of Zhashijia sheep and Valley Tibetan sheep (*P* < 0.05)**

| **PathwayID** | **Pathway** | **List_number** | **Total_number** | ***P*_value** | **FDR** |
| --- | --- | --- | --- | --- | --- |
| ko00230 | Purine metabolism | 16 | 131 | 0.000368 | 0.077254 |
| ko04512 | ECM-receptor interaction | 12 | 90 | 0.000901 | 0.094628 |
| ko04730 | Long-term depression | 9 | 59 | 0.001502 | 0.105123 |
| ko04540 | Gap junction | 11 | 89 | 0.002710 | 0.119960 |
| ko04810 | Regulation of actin cytoskeleton | 20 | 218 | 0.002856 | 0.119960 |
| ko04919 | Thyroid hormone signaling pathway | 13 | 123 | 0.004681 | 0.163838 |
| ko04972 | Pancreatic secretion | 11 | 103 | 0.008293 | 0.169869 |
| ko00600 | Sphingolipid metabolism | 7 | 51 | 0.008923 | 0.169869 |
| ko04360 | Axon guidance | 16 | 180 | 0.009748 | 0.169869 |
| ko00510 | N-Glycan biosynthesis | 7 | 52 | 0.009910 | 0.169869 |
| ko04072 | Phospholipase D signaling pathway | 14 | 150 | 0.010106 | 0.169869 |
| ko04750 | Inflammatory mediator regulation of TRP channels | 11 | 106 | 0.010214 | 0.169869 |
| ko04270 | Vascular smooth muscle contraction | 13 | 136 | 0.010719 | 0.169869 |
| ko04611 | Platelet activation | 12 | 123 | 0.011971 | 0.169869 |
| ko04724 | Glutamatergic synapse | 11 | 109 | 0.012460 | 0.169869 |
| ko04391 | Hippo signaling pathway - fly | 8 | 68 | 0.013286 | 0.169869 |
| ko04510 | Focal adhesion | 17 | 203 | 0.013751 | 0.169869 |
| ko04520 | Adherens junction | 8 | 73 | 0.019781 | 0.230784 |
| ko04071 | Sphingolipid signaling pathway | 11 | 120 | 0.023999 | 0.265254 |
| ko04935 | Growth hormone synthesis, secretion and action | 11 | 121 | 0.025341 | 0.266079 |
| ko04960 | Aldosterone-regulated sodium reabsorption | 5 | 38 | 0.030405 | 0.280206 |
| ko04725 | Cholinergic synapse | 10 | 109 | 0.030425 | 0.280206 |
| ko04713 | Circadian entrainment | 9 | 94 | 0.030689 | 0.280206 |
| ko00592 | alpha-Linolenic acid metabolism | 4 | 27 | 0.034951 | 0.294959 |
| ko04070 | Phosphatidylinositol signaling system | 9 | 97 | 0.036558 | 0.294959 |
| ko00310 | Lysine degradation | 7 | 68 | 0.038257 | 0.294959 |
| ko04015 | Rap1 signaling pathway | 16 | 212 | 0.038850 | 0.294959 |
| ko04392 | Hippo signaling pathway - multiple species | 4 | 28 | 0.039328 | 0.294959 |
| ko04723 | Retrograde endocannabinoid signaling | 12 | 147 | 0.041521 | 0.300667 |
| ko04745 | Phototransduction - fly | 4 | 29 | 0.044003 | 0.308020 |
| ko04924 | Renin secretion | 7 | 71 | 0.046686 | 0.316260 |

**Table S26** The GO enrichment analysis of genes in selective sweep regions of Zhashijia sheep and Valley Tibetan sheep (*P* < 0.05)

| **Category** | **GO.ID** | **Term** | **List** | **Total** | ***P*_value** | **FDR** |
| --- | --- | --- | --- | --- | --- | --- |
| CC | GO:0097060 | synaptic membrane | 25 | 163 | 0.000004 | 0.002966 |
| MF | GO:0004112 | cyclic-nucleotide phosphodiesterase activity | 8 | 22 | 0.000020 | 0.007451 |
| MF | GO:0004114 | 3',5'-cyclic-nucleotide phosphodiesterase activity | 8 | 22 | 0.000020 | 0.007451 |
| MF | GO:0043167 | ion binding | 277 | 3897 | 0.000021 | 0.007451 |
| CC | GO:0045211 | postsynaptic membrane | 19 | 120 | 0.000039 | 0.013046 |
| MF | GO:0043169 | cation binding | 184 | 2467 | 0.000088 | 0.023203 |
| MF | GO:0046872 | metal ion binding | 179 | 2416 | 0.000165 | 0.034978 |
| BP | GO:0099590 | neurotransmitter receptor internalization | 4 | 7 | 0.000359 | 0.717335 |
| CC | GO:0016528 | sarcoplasm | 8 | 35 | 0.000590 | 0.105391 |
| CC | GO:0008305 | integrin complex | 7 | 28 | 0.000731 | 0.105391 |
| MF | GO:0008066 | glutamate receptor activity | 6 | 20 | 0.000733 | 0.128965 |
| CC | GO:0033017 | sarcoplasmic reticulum membrane | 5 | 15 | 0.001046 | 0.105391 |
| MF | GO:0004725 | protein tyrosine phosphatase activity | 13 | 84 | 0.001077 | 0.128965 |
| CC | GO:0034702 | ion channel complex | 22 | 191 | 0.001114 | 0.105391 |
| MF | GO:0030594 | neurotransmitter receptor activity | 10 | 55 | 0.001136 | 0.128965 |
| MF | GO:0004115 | 3',5'-cyclic-AMP phosphodiesterase activity | 4 | 9 | 0.001137 | 0.128965 |
| CC | GO:0016529 | sarcoplasmic reticulum | 7 | 30 | 0.001139 | 0.105391 |
| MF | GO:0004970 | ionotropic glutamate receptor activity | 5 | 15 | 0.001218 | 0.128965 |
| BP | GO:0009719 | response to endogenous stimulus | 63 | 730 | 0.001220 | 0.717335 |
| CC | GO:0098982 | GABA-ergic synapse | 8 | 39 | 0.001264 | 0.105391 |
| CC | GO:0098794 | postsynapse | 29 | 285 | 0.001478 | 0.108173 |
| MF | GO:0022836 | gated channel activity | 27 | 252 | 0.001609 | 0.154880 |
| CC | GO:0098636 | protein complex involved in cell adhesion | 7 | 32 | 0.001707 | 0.108173 |
| MF | GO:0035515 | oxidative RNA demethylase activity | 3 | 5 | 0.001803 | 0.159115 |
| BP | GO:0006198 | cAMP catabolic process | 3 | 5 | 0.001851 | 0.717335 |
| CC | GO:0005930 | axoneme | 11 | 71 | 0.001946 | 0.108173 |
| CC | GO:0097014 | ciliary plasm | 11 | 71 | 0.001946 | 0.108173 |
| BP | GO:0065007 | biological regulation | 429 | 6716 | 0.002161 | 0.717335 |
| MF | GO:0008569 | minus-end-directed microtubule motor activity | 5 | 17 | 0.002277 | 0.183755 |
| MF | GO:0030169 | low-density lipoprotein particle binding | 4 | 11 | 0.002712 | 0.183755 |
| CC | GO:0034704 | calcium channel complex | 8 | 44 | 0.002841 | 0.131806 |
| CC | GO:0032838 | plasma membrane bounded cell projection cytoplasm | 14 | 108 | 0.002911 | 0.131806 |
| CC | GO:0071944 | cell periphery | 210 | 3162 | 0.002964 | 0.131806 |
| MF | GO:0022824 | transmitter-gated ion channel activity | 8 | 43 | 0.003008 | 0.183755 |
| MF | GO:0022835 | transmitter-gated channel activity | 8 | 43 | 0.003008 | 0.183755 |
| BP | GO:0023052 | signaling | 222 | 3239 | 0.003088 | 0.717335 |
| BP | GO:0071495 | cellular response to endogenous stimulus | 57 | 673 | 0.003145 | 0.717335 |
| MF | GO:0003774 | cytoskeletal motor activity | 9 | 53 | 0.003236 | 0.183755 |
| CC | GO:0099055 | integral component of postsynaptic membrane | 8 | 45 | 0.003288 | 0.137080 |
| MF | GO:0046556 | alpha-L-arabinofuranosidase activity | 2 | 2 | 0.003391 | 0.183755 |
| MF | GO:0086007 | voltage-gated calcium channel activity involved in cardiac muscle cell action potential | 2 | 2 | 0.003391 | 0.183755 |
| BP | GO:0003050 | regulation of systemic arterial blood pressure by atrial natriuretic peptide | 2 | 2 | 0.003453 | 0.717335 |
| BP | GO:0007624 | ultradian rhythm | 2 | 2 | 0.003453 | 0.717335 |
| BP | GO:0019566 | arabinose metabolic process | 2 | 2 | 0.003453 | 0.717335 |
| BP | GO:0035552 | oxidative single-stranded DNA demethylation | 2 | 2 | 0.003453 | 0.717335 |
| BP | GO:0046373 | L-arabinose metabolic process | 2 | 2 | 0.003453 | 0.717335 |
| BP | GO:0061152 | trachea submucosa development | 2 | 2 | 0.003453 | 0.717335 |
| BP | GO:0061153 | trachea gland development | 2 | 2 | 0.003453 | 0.717335 |
| BP | GO:1904016 | response to Thyroglobulin triiodothyronine | 2 | 2 | 0.003453 | 0.717335 |
| BP | GO:1904017 | cellular response to Thyroglobulin triiodothyronine | 2 | 2 | 0.003453 | 0.717335 |
| BP | GO:1904533 | regulation of telomeric loop disassembly | 2 | 2 | 0.003453 | 0.717335 |
| MF | GO:0042578 | phosphoric ester hydrolase activity | 27 | 266 | 0.003470 | 0.183755 |
| BP | GO:0009214 | cyclic nucleotide catabolic process | 3 | 6 | 0.003540 | 0.717335 |
| MF | GO:0005096 | GTPase activator activity | 16 | 131 | 0.003895 | 0.196437 |
| CC | GO:0098839 | postsynaptic density membrane | 6 | 28 | 0.004059 | 0.150816 |
| BP | GO:0007154 | cell communication | 223 | 3274 | 0.004160 | 0.717335 |
| CC | GO:0099061 | integral component of postsynaptic density membrane | 5 | 20 | 0.004272 | 0.150816 |
| CC | GO:0098936 | intrinsic component of postsynaptic membrane | 8 | 47 | 0.004345 | 0.150816 |
| CC | GO:0098590 | plasma membrane region | 50 | 610 | 0.004727 | 0.150816 |
| CC | GO:0005886 | plasma membrane | 194 | 2925 | 0.004847 | 0.150816 |
| MF | GO:0045296 | cadherin binding | 5 | 20 | 0.004933 | 0.237436 |
| BP | GO:0045471 | response to ethanol | 5 | 20 | 0.005123 | 0.717335 |
| BP | GO:2000279 | negative regulation of DNA biosynthetic process | 5 | 20 | 0.005123 | 0.717335 |
| CC | GO:0099568 | cytoplasmic region | 15 | 127 | 0.005151 | 0.150816 |
| CC | GO:0043235 | receptor complex | 21 | 203 | 0.005214 | 0.150816 |
| CC | GO:0042383 | sarcolemma | 9 | 59 | 0.005427 | 0.150816 |
| MF | GO:0005545 | 1-phosphatidylinositol binding | 3 | 7 | 0.005777 | 0.265997 |
| CC | GO:0098978 | glutamatergic synapse | 20 | 192 | 0.005852 | 0.152471 |
| BP | GO:0000291 | nuclear-transcribed mRNA catabolic process, exonucleolytic | 3 | 7 | 0.005925 | 0.717335 |
| BP | GO:0048149 | behavioral response to ethanol | 3 | 7 | 0.005925 | 0.717335 |
| CC | GO:0042995 | cell projection | 80 | 1077 | 0.005943 | 0.152471 |
| BP | GO:0048008 | platelet-derived growth factor receptor signaling pathway | 7 | 38 | 0.006011 | 0.717335 |
| MF | GO:0003777 | microtubule motor activity | 5 | 21 | 0.006169 | 0.272196 |
| BP | GO:0050896 | response to stimulus | 304 | 4646 | 0.006298 | 0.717335 |
| CC | GO:0099146 | intrinsic component of postsynaptic density membrane | 5 | 22 | 0.006609 | 0.163267 |
| BP | GO:0003279 | cardiac septum development | 10 | 69 | 0.006747 | 0.717335 |
| BP | GO:0030510 | regulation of BMP signaling pathway | 10 | 69 | 0.006747 | 0.717335 |
| BP | GO:0042058 | regulation of epidermal growth factor receptor signaling pathway | 7 | 39 | 0.006963 | 0.717335 |
| CC | GO:0120025 | plasma membrane bounded cell projection | 77 | 1037 | 0.006981 | 0.166303 |
| BP | GO:0034655 | nucleobase-containing compound catabolic process | 22 | 213 | 0.007108 | 0.717335 |
| CC | GO:0030054 | cell junction | 78 | 1056 | 0.007536 | 0.173331 |
| CC | GO:0031010 | ISWI-type complex | 3 | 8 | 0.008054 | 0.179065 |
| MF | GO:0043168 | anion binding | 128 | 1804 | 0.008242 | 0.323624 |
| BP | GO:0006897 | endocytosis | 24 | 242 | 0.008320 | 0.717335 |
| CC | GO:0099699 | integral component of synaptic membrane | 9 | 63 | 0.008375 | 0.180202 |
| MF | GO:0015276 | ligand-gated ion channel activity | 13 | 106 | 0.008503 | 0.323624 |
| MF | GO:0022834 | ligand-gated channel activity | 13 | 106 | 0.008503 | 0.323624 |
| BP | GO:0098742 | cell-cell adhesion via plasma-membrane adhesion molecules | 14 | 117 | 0.008607 | 0.717335 |
| BP | GO:0061448 | connective tissue development | 18 | 166 | 0.008752 | 0.717335 |
| CC | GO:0005687 | U4 snRNP | 2 | 3 | 0.009152 | 0.186212 |
| MF | GO:0004721 | phosphoprotein phosphatase activity | 14 | 119 | 0.009251 | 0.323624 |
| MF | GO:0098631 | cell adhesion mediator activity | 4 | 15 | 0.009310 | 0.323624 |
| CC | GO:0099060 | integral component of postsynaptic specialization membrane | 6 | 33 | 0.009397 | 0.186212 |
| BP | GO:0001822 | kidney development | 19 | 180 | 0.009506 | 0.717335 |
| CC | GO:0099634 | postsynaptic specialization membrane | 7 | 43 | 0.009561 | 0.186212 |
| BP | GO:0098927 | vesicle-mediated transport between endosomal compartments | 5 | 23 | 0.009609 | 0.717335 |
| CC | GO:0031226 | intrinsic component of plasma membrane | 47 | 589 | 0.009771 | 0.186212 |
| MF | GO:0005219 | ryanodine-sensitive calcium-release channel activity | 2 | 3 | 0.009779 | 0.323624 |
| MF | GO:0043734 | DNA-N1-methyladenine dioxygenase activity | 2 | 3 | 0.009779 | 0.323624 |
| MF | GO:0098639 | collagen binding involved in cell-matrix adhesion | 2 | 3 | 0.009779 | 0.323624 |
| BP | GO:0097305 | response to alcohol | 9 | 62 | 0.009833 | 0.717335 |
| BP | GO:0002674 | negative regulation of acute inflammatory response | 2 | 3 | 0.009953 | 0.717335 |
| BP | GO:0021943 | formation of radial glial scaffolds | 2 | 3 | 0.009953 | 0.717335 |
| BP | GO:0034776 | response to histamine | 2 | 3 | 0.009953 | 0.717335 |
| BP | GO:0035511 | oxidative DNA demethylation | 2 | 3 | 0.009953 | 0.717335 |
| BP | GO:0035553 | oxidative single-stranded RNA demethylation | 2 | 3 | 0.009953 | 0.717335 |
| BP | GO:0035813 | regulation of renal sodium excretion | 2 | 3 | 0.009953 | 0.717335 |
| BP | GO:0044467 | glial cell-derived neurotrophic factor production | 2 | 3 | 0.009953 | 0.717335 |
| BP | GO:0051582 | positive regulation of neurotransmitter uptake | 2 | 3 | 0.009953 | 0.717335 |
| BP | GO:0071349 | cellular response to interleukin-12 | 2 | 3 | 0.009953 | 0.717335 |
| BP | GO:0071871 | response to epinephrine | 2 | 3 | 0.009953 | 0.717335 |
| BP | GO:0071872 | cellular response to epinephrine stimulus | 2 | 3 | 0.009953 | 0.717335 |
| BP | GO:1900166 | regulation of glial cell-derived neurotrophic factor production | 2 | 3 | 0.009953 | 0.717335 |
| BP | GO:1900168 | positive regulation of glial cell-derived neurotrophic factor production | 2 | 3 | 0.009953 | 0.717335 |
| BP | GO:1902731 | negative regulation of chondrocyte proliferation | 2 | 3 | 0.009953 | 0.717335 |
| BP | GO:1902990 | mitotic telomere maintenance via semi-conservative replication | 2 | 3 | 0.009953 | 0.717335 |
| BP | GO:1903850 | regulation of cristae formation | 2 | 3 | 0.009953 | 0.717335 |
| BP | GO:1904430 | negative regulation of t-circle formation | 2 | 3 | 0.009953 | 0.717335 |
| BP | GO:2000588 | positive regulation of platelet-derived growth factor receptor-beta signaling pathway | 2 | 3 | 0.009953 | 0.717335 |
| BP | GO:2001171 | positive regulation of ATP biosynthetic process | 2 | 3 | 0.009953 | 0.717335 |
| BP | GO:0048856 | anatomical structure development | 221 | 3300 | 0.009977 | 0.717335 |
| MF | GO:0005488 | binding | 513 | 8352 | 0.010238 | 0.328291 |
| CC | GO:1990351 | transporter complex | 23 | 243 | 0.010268 | 0.186701 |
| CC | GO:0005887 | integral component of plasma membrane | 44 | 546 | 0.010357 | 0.186701 |
| BP | GO:1901292 | nucleoside phosphate catabolic process | 7 | 42 | 0.010492 | 0.717335 |
| BP | GO:0071772 | response to BMP | 13 | 108 | 0.010603 | 0.717335 |
| BP | GO:0071773 | cellular response to BMP stimulus | 13 | 108 | 0.010603 | 0.717335 |
| MF | GO:0051539 | 4 iron, 4 sulfur cluster binding | 6 | 33 | 0.010995 | 0.328291 |
| MF | GO:0005230 | extracellular ligand-gated ion channel activity | 9 | 64 | 0.011404 | 0.328291 |
| BP | GO:0035137 | hindlimb morphogenesis | 5 | 24 | 0.011563 | 0.717335 |
| MF | GO:0005216 | ion channel activity | 30 | 333 | 0.011597 | 0.328291 |
| MF | GO:0071813 | lipoprotein particle binding | 4 | 16 | 0.011851 | 0.328291 |
| MF | GO:0071814 | protein-lipid complex binding | 4 | 16 | 0.011851 | 0.328291 |
| BP | GO:0007167 | enzyme-linked receptor protein signaling pathway | 45 | 543 | 0.011972 | 0.717335 |
| BP | GO:0050789 | regulation of biological process | 398 | 6300 | 0.012190 | 0.717335 |
| BP | GO:0046058 | cAMP metabolic process | 4 | 16 | 0.012219 | 0.717335 |
| BP | GO:0050850 | positive regulation of calcium-mediated signaling | 4 | 16 | 0.012219 | 0.717335 |
| CC | GO:0043197 | dendritic spine | 7 | 45 | 0.012222 | 0.208819 |
| CC | GO:0005905 | clathrin-coated pit | 6 | 35 | 0.012523 | 0.208819 |
| CC | GO:0098948 | intrinsic component of postsynaptic specialization membrane | 6 | 35 | 0.012523 | 0.208819 |
| MF | GO:0015278 | calcium-release channel activity | 3 | 9 | 0.012700 | 0.328291 |
| MF | GO:0036122 | BMP binding | 3 | 9 | 0.012700 | 0.328291 |
| MF | GO:0030374 | nuclear receptor coactivator activity | 6 | 34 | 0.012710 | 0.328291 |
| BP | GO:0035973 | aggrephagy | 3 | 9 | 0.013015 | 0.717335 |
| BP | GO:0070886 | positive regulation of calcineurin-NFAT signaling cascade | 3 | 9 | 0.013015 | 0.717335 |
| BP | GO:0106058 | positive regulation of calcineurin-mediated signaling | 3 | 9 | 0.013015 | 0.717335 |
| MF | GO:0032559 | adenyl ribonucleotide binding | 90 | 1232 | 0.013120 | 0.330807 |
| CC | GO:0045202 | synapse | 50 | 645 | 0.013162 | 0.212273 |
| BP | GO:0071407 | cellular response to organic cyclic compound | 24 | 252 | 0.013322 | 0.717335 |
| CC | GO:0005813 | centrosome | 34 | 406 | 0.013367 | 0.212273 |
| BP | GO:1901184 | regulation of ERBB signaling pathway | 7 | 44 | 0.013471 | 0.717335 |
| BP | GO:0051716 | cellular response to stimulus | 250 | 3803 | 0.013839 | 0.717335 |
| BP | GO:0072001 | renal system development | 19 | 187 | 0.013933 | 0.717335 |
| MF | GO:0030554 | adenyl nucleotide binding | 90 | 1236 | 0.014208 | 0.349906 |
| CC | GO:0034707 | chloride channel complex | 6 | 36 | 0.014331 | 0.213788 |
| CC | GO:1902495 | transmembrane transporter complex | 22 | 237 | 0.014653 | 0.213788 |
| CC | GO:0099240 | intrinsic component of synaptic membrane | 9 | 69 | 0.014893 | 0.213788 |
| MF | GO:0008237 | metallopeptidase activity | 17 | 164 | 0.014954 | 0.359923 |
| BP | GO:0001505 | regulation of neurotransmitter levels | 13 | 113 | 0.015151 | 0.717335 |
| BP | GO:0030509 | BMP signaling pathway | 12 | 101 | 0.015165 | 0.717335 |
| CC | GO:0044309 | neuron spine | 7 | 47 | 0.015379 | 0.213788 |
| CC | GO:0014069 | postsynaptic density | 13 | 118 | 0.015385 | 0.213788 |
| CC | GO:0032279 | asymmetric synapse | 13 | 118 | 0.015385 | 0.213788 |
| MF | GO:0030331 | nuclear estrogen receptor binding | 5 | 26 | 0.015682 | 0.369060 |
| BP | GO:0030048 | actin filament-based movement | 8 | 56 | 0.015950 | 0.717335 |
| CC | GO:0005856 | cytoskeleton | 95 | 1364 | 0.015952 | 0.217149 |
| BP | GO:0006898 | receptor-mediated endocytosis | 13 | 114 | 0.016216 | 0.717335 |
| BP | GO:0009116 | nucleoside metabolic process | 5 | 26 | 0.016247 | 0.717335 |
| MF | GO:0030552 | cAMP binding | 3 | 10 | 0.017368 | 0.375728 |
| MF | GO:0005524 | ATP binding | 87 | 1200 | 0.017533 | 0.375728 |
| MF | GO:0004222 | metalloendopeptidase activity | 11 | 92 | 0.017691 | 0.375728 |
| BP | GO:0014850 | response to muscle activity | 3 | 10 | 0.017791 | 0.717335 |
| BP | GO:1903649 | regulation of cytoplasmic transport | 3 | 10 | 0.017791 | 0.717335 |
| BP | GO:1904752 | regulation of vascular associated smooth muscle cell migration | 3 | 10 | 0.017791 | 0.717335 |
| MF | GO:0008081 | phosphoric diester hydrolase activity | 9 | 69 | 0.018165 | 0.375728 |
| MF | GO:0030551 | cyclic nucleotide binding | 4 | 18 | 0.018166 | 0.375728 |
| BP | GO:0007165 | signal transduction | 196 | 2936 | 0.018701 | 0.717335 |
| BP | GO:0035116 | embryonic hindlimb morphogenesis | 4 | 18 | 0.018716 | 0.717335 |
| CC | GO:0099572 | postsynaptic specialization | 14 | 134 | 0.018721 | 0.249732 |
| MF | GO:0008656 | cysteine-type endopeptidase activator activity involved in apoptotic process | 2 | 4 | 0.018804 | 0.375728 |
| MF | GO:0030284 | nuclear estrogen receptor activity | 2 | 4 | 0.018804 | 0.375728 |
| MF | GO:0070815 | peptidyl-lysine 5-dioxygenase activity | 2 | 4 | 0.018804 | 0.375728 |
| BP | GO:0071559 | response to transforming growth factor beta | 16 | 154 | 0.018877 | 0.717335 |
| BP | GO:0009261 | ribonucleotide catabolic process | 5 | 27 | 0.019001 | 0.717335 |
| BP | GO:0031503 | protein-containing complex localization | 9 | 69 | 0.019129 | 0.717335 |
| BP | GO:0031532 | actin cytoskeleton reorganization | 9 | 69 | 0.019129 | 0.717335 |
| BP | GO:0010641 | positive regulation of platelet-derived growth factor receptor signaling pathway | 2 | 4 | 0.019131 | 0.717335 |
| BP | GO:0021684 | cerebellar granular layer formation | 2 | 4 | 0.019131 | 0.717335 |
| BP | GO:0021707 | cerebellar granule cell differentiation | 2 | 4 | 0.019131 | 0.717335 |
| BP | GO:0032201 | telomere maintenance via semi-conservative replication | 2 | 4 | 0.019131 | 0.717335 |
| BP | GO:0035513 | oxidative RNA demethylation | 2 | 4 | 0.019131 | 0.717335 |
| BP | GO:0042136 | neurotransmitter biosynthetic process | 2 | 4 | 0.019131 | 0.717335 |
| BP | GO:0045906 | negative regulation of vasoconstriction | 2 | 4 | 0.019131 | 0.717335 |
| BP | GO:0070341 | fat cell proliferation | 2 | 4 | 0.019131 | 0.717335 |
| BP | GO:0070344 | regulation of fat cell proliferation | 2 | 4 | 0.019131 | 0.717335 |
| BP | GO:0070487 | monocyte aggregation | 2 | 4 | 0.019131 | 0.717335 |
| BP | GO:0075044 | positive regulation by symbiont of host autophagy | 2 | 4 | 0.019131 | 0.717335 |
| BP | GO:0075071 | modulation by symbiont of host autophagy | 2 | 4 | 0.019131 | 0.717335 |
| BP | GO:1901896 | positive regulation of ATPase-coupled calcium transmembrane transporter activity | 2 | 4 | 0.019131 | 0.717335 |
| BP | GO:1903651 | positive regulation of cytoplasmic transport | 2 | 4 | 0.019131 | 0.717335 |
| BP | GO:1904429 | regulation of t-circle formation | 2 | 4 | 0.019131 | 0.717335 |
| BP | GO:1904753 | negative regulation of vascular associated smooth muscle cell migration | 2 | 4 | 0.019131 | 0.717335 |
| BP | GO:0048736 | appendage development | 14 | 129 | 0.019228 | 0.717335 |
| BP | GO:0060173 | limb development | 14 | 129 | 0.019228 | 0.717335 |
| BP | GO:0070848 | response to growth factor | 33 | 386 | 0.019303 | 0.717335 |
| BP | GO:0120162 | positive regulation of cold-induced thermogenesis | 8 | 58 | 0.019436 | 0.717335 |
| BP | GO:0007155 | cell adhesion | 57 | 740 | 0.020617 | 0.717335 |
| BP | GO:0046700 | heterocycle catabolic process | 22 | 235 | 0.020669 | 0.717335 |
| CC | GO:0030286 | dynein complex | 6 | 39 | 0.020829 | 0.271148 |
| CC | GO:0035869 | ciliary transition zone | 7 | 50 | 0.021139 | 0.271148 |
| MF | GO:0005509 | calcium ion binding | 40 | 494 | 0.021363 | 0.380851 |
| BP | GO:0001655 | urogenital system development | 20 | 209 | 0.021518 | 0.717335 |
| MF | GO:0005253 | anion channel activity | 9 | 71 | 0.021554 | 0.380851 |
| MF | GO:0009975 | cyclase activity | 4 | 19 | 0.021975 | 0.380851 |
| BP | GO:0014909 | smooth muscle cell migration | 5 | 28 | 0.022045 | 0.717335 |
| BP | GO:0044270 | cellular nitrogen compound catabolic process | 22 | 237 | 0.022530 | 0.717335 |
| BP | GO:0035640 | exploration behavior | 4 | 19 | 0.022631 | 0.717335 |
| BP | GO:0007156 | homophilic cell adhesion via plasma membrane adhesion molecules | 9 | 71 | 0.022678 | 0.717335 |
| MF | GO:0099095 | ligand-gated anion channel activity | 3 | 11 | 0.022864 | 0.380851 |
| CC | GO:0005929 | cilium | 33 | 408 | 0.023105 | 0.290772 |
| BP | GO:0030705 | cytoskeleton-dependent intracellular transport | 11 | 95 | 0.023293 | 0.717335 |
| BP | GO:0042133 | neurotransmitter metabolic process | 3 | 11 | 0.023412 | 0.717335 |
| BP | GO:0050765 | negative regulation of phagocytosis | 3 | 11 | 0.023412 | 0.717335 |
| BP | GO:0051590 | positive regulation of neurotransmitter transport | 3 | 11 | 0.023412 | 0.717335 |
| BP | GO:1904738 | vascular associated smooth muscle cell migration | 3 | 11 | 0.023412 | 0.717335 |
| BP | GO:0061035 | regulation of cartilage development | 7 | 49 | 0.023483 | 0.717335 |
| CC | GO:0005874 | microtubule | 19 | 206 | 0.023576 | 0.291209 |
| MF | GO:0015085 | calcium ion transmembrane transporter activity | 10 | 84 | 0.023742 | 0.380851 |
| BP | GO:0050794 | regulation of cellular process | 369 | 5858 | 0.024105 | 0.717335 |
| BP | GO:0032502 | developmental process | 231 | 3533 | 0.024366 | 0.717335 |
| MF | GO:0015103 | inorganic anion transmembrane transporter activity | 12 | 109 | 0.024630 | 0.380851 |
| CC | GO:0005891 | voltage-gated calcium channel complex | 5 | 30 | 0.024698 | 0.299517 |
| BP | GO:0072523 | purine-containing compound catabolic process | 5 | 29 | 0.025388 | 0.717335 |
| BP | GO:0060021 | roof of mouth development | 8 | 61 | 0.025634 | 0.717335 |
| BP | GO:0010033 | response to organic substance | 104 | 1478 | 0.026146 | 0.717335 |
| BP | GO:0001764 | neuron migration | 12 | 109 | 0.026149 | 0.717335 |
| CC | GO:0031083 | BLOC-1 complex | 3 | 12 | 0.026731 | 0.318383 |
| MF | GO:0017076 | purine nucleotide binding | 106 | 1524 | 0.027047 | 0.380851 |
| BP | GO:0046530 | photoreceptor cell differentiation | 6 | 40 | 0.028103 | 0.717335 |
| CC | GO:0005579 | membrane attack complex | 2 | 5 | 0.028281 | 0.321204 |
| CC | GO:1990454 | L-type voltage-gated calcium channel complex | 2 | 5 | 0.028281 | 0.321204 |
| CC | GO:0030027 | lamellipodium | 12 | 115 | 0.028412 | 0.321204 |
| BP | GO:1904375 | regulation of protein localization to cell periphery | 9 | 74 | 0.028850 | 0.717335 |
| BP | GO:0071560 | cellular response to transforming growth factor beta stimulus | 15 | 149 | 0.028941 | 0.717335 |
| BP | GO:0046434 | organophosphate catabolic process | 10 | 86 | 0.028967 | 0.717335 |
| BP | GO:0042461 | photoreceptor cell development | 5 | 30 | 0.029040 | 0.717335 |
| MF | GO:0004622 | lysophospholipase activity | 3 | 12 | 0.029191 | 0.380851 |
| BP | GO:0035107 | appendage morphogenesis | 12 | 111 | 0.029626 | 0.717335 |
| BP | GO:0035108 | limb morphogenesis | 12 | 111 | 0.029626 | 0.717335 |
| BP | GO:0099003 | vesicle-mediated transport in synapse | 12 | 111 | 0.029626 | 0.717335 |
| BP | GO:0001778 | plasma membrane repair | 3 | 12 | 0.029879 | 0.717335 |
| BP | GO:0016082 | synaptic vesicle priming | 3 | 12 | 0.029879 | 0.717335 |
| BP | GO:0030852 | regulation of granulocyte differentiation | 3 | 12 | 0.029879 | 0.717335 |
| BP | GO:0071360 | cellular response to exogenous dsRNA | 3 | 12 | 0.029879 | 0.717335 |
| BP | GO:1904862 | inhibitory synapse assembly | 3 | 12 | 0.029879 | 0.717335 |
| BP | GO:0007018 | microtubule-based movement | 22 | 244 | 0.030077 | 0.717335 |
| MF | GO:0003854 | 3-beta-hydroxy-delta5-steroid dehydrogenase activity | 2 | 5 | 0.030138 | 0.380851 |
| MF | GO:0005384 | manganese ion transmembrane transporter activity | 2 | 5 | 0.030138 | 0.380851 |
| MF | GO:0016505 | peptidase activator activity involved in apoptotic process | 2 | 5 | 0.030138 | 0.380851 |
| MF | GO:0022851 | GABA-gated chloride ion channel activity | 2 | 5 | 0.030138 | 0.380851 |
| MF | GO:0042392 | sphingosine-1-phosphate phosphatase activity | 2 | 5 | 0.030138 | 0.380851 |
| MF | GO:0098634 | cell-matrix adhesion mediator activity | 2 | 5 | 0.030138 | 0.380851 |
| BP | GO:0016079 | synaptic vesicle exocytosis | 8 | 63 | 0.030459 | 0.717335 |
| BP | GO:2000278 | regulation of DNA biosynthetic process | 8 | 63 | 0.030459 | 0.717335 |
| BP | GO:0006307 | DNA dealkylation involved in DNA repair | 2 | 5 | 0.030652 | 0.717335 |
| BP | GO:0007216 | G protein-coupled glutamate receptor signaling pathway | 2 | 5 | 0.030652 | 0.717335 |
| BP | GO:0007271 | synaptic transmission, cholinergic | 2 | 5 | 0.030652 | 0.717335 |
| BP | GO:0010637 | negative regulation of mitochondrial fusion | 2 | 5 | 0.030652 | 0.717335 |
| BP | GO:0030810 | positive regulation of nucleotide biosynthetic process | 2 | 5 | 0.030652 | 0.717335 |
| BP | GO:0035812 | renal sodium excretion | 2 | 5 | 0.030652 | 0.717335 |
| BP | GO:0070671 | response to interleukin-12 | 2 | 5 | 0.030652 | 0.717335 |
| BP | GO:0070989 | oxidative demethylation | 2 | 5 | 0.030652 | 0.717335 |
| BP | GO:0090435 | protein localization to nuclear envelope | 2 | 5 | 0.030652 | 0.717335 |
| BP | GO:0098884 | postsynaptic neurotransmitter receptor internalization | 2 | 5 | 0.030652 | 0.717335 |
| BP | GO:0140239 | postsynaptic endocytosis | 2 | 5 | 0.030652 | 0.717335 |
| BP | GO:1900373 | positive regulation of purine nucleotide biosynthetic process | 2 | 5 | 0.030652 | 0.717335 |
| BP | GO:1902947 | regulation of tau-protein kinase activity | 2 | 5 | 0.030652 | 0.717335 |
| BP | GO:1902965 | regulation of protein localization to early endosome | 2 | 5 | 0.030652 | 0.717335 |
| BP | GO:1902966 | positive regulation of protein localization to early endosome | 2 | 5 | 0.030652 | 0.717335 |
| BP | GO:1990504 | dense core granule exocytosis | 2 | 5 | 0.030652 | 0.717335 |
| CC | GO:0098797 | plasma membrane protein complex | 27 | 328 | 0.030846 | 0.342905 |
| MF | GO:0005246 | calcium channel regulator activity | 4 | 21 | 0.030958 | 0.380851 |
| BP | GO:0022029 | telencephalon cell migration | 6 | 41 | 0.031344 | 0.717335 |
| MF | GO:0015267 | channel activity | 30 | 361 | 0.031419 | 0.380851 |
| MF | GO:0022803 | passive transmembrane transporter activity | 30 | 361 | 0.031419 | 0.380851 |
| BP | GO:0045022 | early endosome to late endosome transport | 4 | 21 | 0.031857 | 0.717335 |
| BP | GO:0019439 | aromatic compound catabolic process | 22 | 246 | 0.032550 | 0.717335 |
| CC | GO:0043005 | neuron projection | 38 | 496 | 0.032980 | 0.360623 |
| BP | GO:0099072 | regulation of postsynaptic membrane neurotransmitter receptor levels | 5 | 31 | 0.033007 | 0.717335 |
| BP | GO:0071363 | cellular response to growth factor stimulus | 31 | 374 | 0.033164 | 0.717335 |
| MF | GO:0016706 | 2-oxoglutarate-dependent dioxygenase activity | 6 | 42 | 0.033540 | 0.380851 |
| BP | GO:0007229 | integrin-mediated signaling pathway | 9 | 76 | 0.033565 | 0.717335 |
| BP | GO:0099111 | microtubule-based transport | 11 | 101 | 0.034668 | 0.717335 |
| BP | GO:0032922 | circadian regulation of gene expression | 6 | 42 | 0.034820 | 0.717335 |
| BP | GO:1902017 | regulation of cilium assembly | 6 | 42 | 0.034820 | 0.717335 |
| MF | GO:0032553 | ribonucleotide binding | 105 | 1525 | 0.035398 | 0.380851 |
| BP | GO:0060041 | retina development in camera-type eye | 10 | 89 | 0.035622 | 0.717335 |
| BP | GO:0007368 | determination of left/right symmetry | 8 | 65 | 0.035874 | 0.717335 |
| MF | GO:0043015 | gamma-tubulin binding | 4 | 22 | 0.036147 | 0.380851 |
| CC | GO:0005875 | microtubule associated complex | 8 | 68 | 0.036559 | 0.393302 |
| BP | GO:0009187 | cyclic nucleotide metabolic process | 4 | 22 | 0.037182 | 0.717335 |
| BP | GO:0061462 | protein localization to lysosome | 4 | 22 | 0.037182 | 0.717335 |
| BP | GO:0006910 | phagocytosis, recognition | 3 | 13 | 0.037185 | 0.717335 |
| BP | GO:0046339 | diacylglycerol metabolic process | 3 | 13 | 0.037185 | 0.717335 |
| BP | GO:0098801 | regulation of renal system process | 3 | 13 | 0.037185 | 0.717335 |
| MF | GO:0016791 | phosphatase activity | 18 | 196 | 0.037240 | 0.380851 |
| BP | GO:1901019 | regulation of calcium ion transmembrane transporter activity | 5 | 32 | 0.037295 | 0.717335 |
| BP | GO:1901657 | glycosyl compound metabolic process | 5 | 32 | 0.037295 | 0.717335 |
| CC | GO:0060076 | excitatory synapse | 4 | 23 | 0.037618 | 0.398273 |
| BP | GO:0030902 | hindbrain development | 10 | 90 | 0.038058 | 0.717335 |
| MF | GO:0032555 | purine ribonucleotide binding | 104 | 1514 | 0.038234 | 0.380851 |
| BP | GO:1990778 | protein localization to cell periphery | 18 | 195 | 0.038356 | 0.717335 |
| BP | GO:0021885 | forebrain cell migration | 6 | 43 | 0.038534 | 0.717335 |
| CC | GO:0098984 | neuron to neuron synapse | 13 | 134 | 0.038873 | 0.399744 |
| BP | GO:0007160 | cell-matrix adhesion | 13 | 129 | 0.039859 | 0.717335 |
| BP | GO:0030030 | cell projection organization | 62 | 846 | 0.039870 | 0.717335 |
| BP | GO:0016482 | cytosolic transport | 10 | 91 | 0.040608 | 0.717335 |
| BP | GO:0030534 | adult behavior | 10 | 91 | 0.040608 | 0.717335 |
| MF | GO:0003712 | transcription coregulator activity | 22 | 254 | 0.040609 | 0.380851 |
| CC | GO:0034451 | centriolar satellite | 9 | 82 | 0.040627 | 0.399744 |
| CC | GO:0036064 | ciliary basal body | 11 | 108 | 0.040631 | 0.399744 |
| CC | GO:0016589 | NURF complex | 2 | 6 | 0.040856 | 0.399744 |
| CC | GO:0060077 | inhibitory synapse | 2 | 6 | 0.040856 | 0.399744 |
| BP | GO:0086003 | cardiac muscle cell contraction | 5 | 33 | 0.041909 | 0.717335 |
| BP | GO:0071310 | cellular response to organic substance | 85 | 1207 | 0.042009 | 0.717335 |
| BP | GO:1902115 | regulation of organelle assembly | 12 | 117 | 0.042042 | 0.717335 |
| BP | GO:0009154 | purine ribonucleotide catabolic process | 4 | 23 | 0.042986 | 0.717335 |
| BP | GO:0014910 | regulation of smooth muscle cell migration | 4 | 23 | 0.042986 | 0.717335 |
| BP | GO:0042073 | intraciliary transport | 4 | 23 | 0.042986 | 0.717335 |
| BP | GO:0061036 | positive regulation of cartilage development | 4 | 23 | 0.042986 | 0.717335 |
| BP | GO:0086002 | cardiac muscle cell action potential involved in contraction | 4 | 23 | 0.042986 | 0.717335 |
| BP | GO:0001501 | skeletal system development | 28 | 339 | 0.042988 | 0.717335 |
| BP | GO:1901699 | cellular response to nitrogen compound | 25 | 296 | 0.043118 | 0.717335 |
| BP | GO:0051302 | regulation of cell division | 10 | 92 | 0.043272 | 0.717335 |
| MF | GO:0004169 | dolichyl-phosphate-mannose-protein mannosyltransferase activity | 2 | 6 | 0.043482 | 0.380851 |
| MF | GO:0005225 | volume-sensitive anion channel activity | 2 | 6 | 0.043482 | 0.380851 |
| MF | GO:0016885 | ligase activity, forming carbon-carbon bonds | 2 | 6 | 0.043482 | 0.380851 |
| MF | GO:0017162 | aryl hydrocarbon receptor binding | 2 | 6 | 0.043482 | 0.380851 |
| MF | GO:0050291 | sphingosine N-acyltransferase activity | 2 | 6 | 0.043482 | 0.380851 |
| MF | GO:0098821 | BMP receptor activity | 2 | 6 | 0.043482 | 0.380851 |
| CC | GO:0009897 | external side of plasma membrane | 14 | 150 | 0.043494 | 0.399744 |
| BP | GO:0009163 | nucleoside biosynthetic process | 2 | 6 | 0.044208 | 0.717335 |
| BP | GO:0016075 | rRNA catabolic process | 2 | 6 | 0.044208 | 0.717335 |
| BP | GO:0021683 | cerebellar granular layer morphogenesis | 2 | 6 | 0.044208 | 0.717335 |
| BP | GO:0021826 | substrate-independent telencephalic tangential migration | 2 | 6 | 0.044208 | 0.717335 |
| BP | GO:0021830 | interneuron migration from the subpallium to the cortex | 2 | 6 | 0.044208 | 0.717335 |
| BP | GO:0021843 | substrate-independent telencephalic tangential interneuron migration | 2 | 6 | 0.044208 | 0.717335 |
| BP | GO:0021932 | hindbrain radial glia guided cell migration | 2 | 6 | 0.044208 | 0.717335 |
| BP | GO:0022605 | mammalian oogenesis stage | 2 | 6 | 0.044208 | 0.717335 |
| BP | GO:0022616 | DNA strand elongation | 2 | 6 | 0.044208 | 0.717335 |
| BP | GO:0030854 | positive regulation of granulocyte differentiation | 2 | 6 | 0.044208 | 0.717335 |
| BP | GO:0031953 | negative regulation of protein autophosphorylation | 2 | 6 | 0.044208 | 0.717335 |
| BP | GO:0033292 | T-tubule organization | 2 | 6 | 0.044208 | 0.717335 |
| BP | GO:0034404 | nucleobase-containing small molecule biosynthetic process | 2 | 6 | 0.044208 | 0.717335 |
| BP | GO:0042451 | purine nucleoside biosynthetic process | 2 | 6 | 0.044208 | 0.717335 |
| BP | GO:0042455 | ribonucleoside biosynthetic process | 2 | 6 | 0.044208 | 0.717335 |
| BP | GO:0043928 | exonucleolytic catabolism of deadenylated mRNA | 2 | 6 | 0.044208 | 0.717335 |
| BP | GO:0044062 | regulation of excretion | 2 | 6 | 0.044208 | 0.717335 |
| BP | GO:0046129 | purine ribonucleoside biosynthetic process | 2 | 6 | 0.044208 | 0.717335 |
| BP | GO:0046834 | lipid phosphorylation | 2 | 6 | 0.044208 | 0.717335 |
| BP | GO:0060770 | negative regulation of epithelial cell proliferation involved in prostate gland development | 2 | 6 | 0.044208 | 0.717335 |
| BP | GO:0140059 | dendrite arborization | 2 | 6 | 0.044208 | 0.717335 |
| BP | GO:1901894 | regulation of ATPase-coupled calcium transmembrane transporter activity | 2 | 6 | 0.044208 | 0.717335 |
| BP | GO:1902946 | protein localization to early endosome | 2 | 6 | 0.044208 | 0.717335 |
| BP | GO:1903441 | protein localization to ciliary membrane | 2 | 6 | 0.044208 | 0.717335 |
| BP | GO:2000586 | regulation of platelet-derived growth factor receptor-beta signaling pathway | 2 | 6 | 0.044208 | 0.717335 |
| BP | GO:0051216 | cartilage development | 13 | 131 | 0.044258 | 0.717335 |
| MF | GO:0004672 | protein kinase activity | 39 | 506 | 0.044270 | 0.380851 |
| MF | GO:0001965 | G-protein alpha-subunit binding | 3 | 14 | 0.044303 | 0.380851 |
| MF | GO:0099604 | ligand-gated calcium channel activity | 3 | 14 | 0.044303 | 0.380851 |
| CC | GO:0005622 | intracellular anatomical structure | 523 | 8919 | 0.044475 | 0.399744 |
| BP | GO:0009653 | anatomical structure morphogenesis | 111 | 1624 | 0.044954 | 0.717335 |
| CC | GO:0034703 | cation channel complex | 13 | 137 | 0.045177 | 0.399744 |
| MF | GO:0035639 | purine ribonucleoside triphosphate binding | 101 | 1477 | 0.045218 | 0.380851 |
| BP | GO:0009125 | nucleoside monophosphate catabolic process | 3 | 14 | 0.045314 | 0.717335 |
| BP | GO:0010640 | regulation of platelet-derived growth factor receptor signaling pathway | 3 | 14 | 0.045314 | 0.717335 |
| BP | GO:0034661 | ncRNA catabolic process | 3 | 14 | 0.045314 | 0.717335 |
| BP | GO:1900078 | positive regulation of cellular response to insulin stimulus | 3 | 14 | 0.045314 | 0.717335 |
| BP | GO:1902883 | negative regulation of response to oxidative stress | 3 | 14 | 0.045314 | 0.717335 |
| BP | GO:1901361 | organic cyclic compound catabolic process | 22 | 255 | 0.045597 | 0.717335 |
| BP | GO:0032501 | multicellular organismal process | 254 | 3972 | 0.045999 | 0.717335 |
| BP | GO:0070252 | actin-mediated cell contraction | 6 | 45 | 0.046695 | 0.717335 |
| BP | GO:0003205 | cardiac chamber development | 11 | 106 | 0.046762 | 0.717335 |
| BP | GO:0090287 | regulation of cellular response to growth factor stimulus | 17 | 186 | 0.046786 | 0.717335 |
| BP | GO:0043279 | response to alkaloid | 5 | 34 | 0.046852 | 0.717335 |
| CC | GO:0031901 | early endosome membrane | 7 | 59 | 0.046957 | 0.399744 |
| MF | GO:0032451 | demethylase activity | 4 | 24 | 0.047937 | 0.380851 |
| CC | GO:0098552 | side of membrane | 20 | 238 | 0.047998 | 0.399744 |
| CC | GO:0098802 | plasma membrane signaling receptor complex | 11 | 111 | 0.048014 | 0.399744 |
| CC | GO:0005815 | microtubule organizing center | 37 | 496 | 0.048832 | 0.399744 |
| BP | GO:0051937 | catecholamine transport | 4 | 24 | 0.049271 | 0.717335 |
| BP | GO:0060612 | adipose tissue development | 4 | 24 | 0.049271 | 0.717335 |
| BP | GO:0072583 | clathrin-dependent endocytosis | 4 | 24 | 0.049271 | 0.717335 |
| MF | GO:0050839 | cell adhesion molecule binding | 12 | 121 | 0.049322 | 0.380851 |
| MF | GO:0003713 | transcription coactivator activity | 14 | 148 | 0.049893 | 0.380851 |

**Table S27** The common genes in unique SNPs and the selective sweep region of Tibetan sheep relative to Argali

| **Chr** | **GeneID** | **Description** |
| --- | --- | --- |
| NC_056057.1 | gene-CALCR | calcitonin receptor |
| NC_056077.1 | gene-TRAP1 | heat shock protein 75 kDa, mitochondrial isoform X1 |
| NC_056057.1 | gene-KMT2C | histone-lysine N-methyltransferase 2C isoform X1 |
| NC_056054.1 | gene-RPAP2 | putative RNA polymerase II subunit B1 CTD phosphatase RPAP2 isoform X1 |
| NC_056056.1 | gene-TOR4A | torsin-4A |
| NC_056061.1 | gene-PDSS2 | all trans-polyprenyl-diphosphate synthase PDSS2 isoform X1 |
| NC_056055.1 | gene-SP5 | transcription factor Sp5 isoform X1 |
| NC_056054.1 | gene-ATP13A3 | polyamine-transporting ATPase 13A3 isoform X1 |
| NC_056057.1 | gene-LHFPL3 | LHFPL tetraspan subfamily member 3 protein isoform X1 |
| NC_056061.1 | gene-TRNAS-GGA-142 | - |
| NC_056057.1 | gene-CAPZA2 | F-actin-capping protein subunit alpha-2 |
| NC_056059.1 | gene-LOC105615523 | - |
| NC_056054.1 | gene-IFT80 | intraflagellar transport protein 80 homolog isoform X1 |
| NC_056054.1 | gene-LOC101110467 | transmembrane protein 45A isoform X1 |
| NC_056055.1 | gene-KDM4C | lysine-specific demethylase 4C isoform X1 |
| NC_056060.1 | gene-CERT1 | ceramide transfer protein isoform X3 |
| NC_056056.1 | gene-SCAF11 | protein SCAF11 isoform X1 |
| NC_056056.1 | gene-LEMD3 | inner nuclear membrane protein Man1 |
| NC_056062.1 | gene-LOC121820322 | - |
| NC_056055.1 | gene-MARCHF4 | E3 ubiquitin-protein ligase MARCHF4 |
| NC_056069.1 | gene-CDH10 | cadherin-10 isoform X1 |
| NC_056054.1 | gene-PTGER3 | prostaglandin E2 receptor EP3 subtype isoform X1 |
| NC_056055.1 | gene-PRUNE2 | protein prune homolog 2 isoform X1 |
| NC_056056.1 | gene-EXOC6B | exocyst complex component 6B isoform X1 |
| NC_056078.1 | gene-LYST | lysosomal-trafficking regulator isoform X1 |
| NC_056055.1 | gene-ADAMTSL1 | ADAMTS-like protein 1 isoform X1 |
| NC_056079.1 | gene-ZNF385D | zinc finger protein 385D isoform X1 |
| NC_056054.1 | gene-LOC101103547 | guanylate-binding protein 6-like |
| NC_056077.1 | gene-MYH11 | myosin-11 isoform X1 |
| NC_056056.1 | gene-MERTK | tyrosine-protein kinase Mer isoform X1 |
| NC_056077.1 | gene-LOC121817803 | - |
| NC_056056.1 | gene-FANCL | E3 ubiquitin-protein ligase FANCL isoform X1 |
| NC_056060.1 | gene-TOMM20L | TOMM20-like protein 1 |
| NC_056055.1 | gene-TMEM268 | transmembrane protein 268 |
| NC_056057.1 | gene-MAGI2 | membrane-associated guanylate kinase, WW and PDZ domain-containing protein 2 |
| NC_056056.1 | gene-TOGARAM2 | TOG array regulator of axonemal microtubules protein 2 |
| NC_056057.1 | gene-SNX13 | sorting nexin-13 |
| NC_056057.1 | gene-HDAC9 | histone deacetylase 9 isoform X1 |
| NC_056056.1 | gene-TCF7L1 | transcription factor 7-like 1 |
| NC_056054.1 | gene-CADM2 | cell adhesion molecule 2 isoform X1 |
| NC_056071.1 | gene-TRAF3 | TNF receptor-associated factor 3 isoform X1 |
| NC_056054.1 | gene-KCNH8 | potassium voltage-gated channel subfamily H member 8 |
| NC_056057.1 | gene-MGAM | maltase-glucoamylase, intestinal |
| NC_056055.1 | gene-PMS1 | PMS1 protein homolog 1 isoform X1 |
| NC_056058.1 | gene-MED16 | mediator of RNA polymerase II transcription subunit 16 |
| NC_056054.1 | gene-PARP9 | protein mono-ADP-ribosyltransferase PARP9 isoform X1 |
| NC_056075.1 | gene-SORCS3 | VPS10 domain-containing receptor SorCS3 |
| NC_056054.1 | gene-DNAJC13 | dnaJ homolog subfamily C member 13 isoform X1 |
| NC_056055.1 | gene-PCSK5 | proprotein convertase subtilisin/kexin type 5 isoform X1 |
| NC_056054.1 | gene-LRRIQ3 | leucine-rich repeat and IQ domain-containing protein 3 isoform X1 |
| NC_056054.1 | gene-ATF6 | cyclic AMP-dependent transcription factor ATF-6 alpha isoform X1 |
| NC_056058.1 | gene-LOC101110412 | olfactory receptor-like protein OLF4 |
| NC_056056.1 | gene-B3GNT2 | N-acetyllactosaminide beta-1,3-N-acetylglucosaminyltransferase 2 |
| NC_056057.1 | gene-LOC105607520 | - |
| NC_056057.1 | gene-TCAF1 | TRPM8 channel-associated factor 1 isoform X1 |
| NC_056054.1 | gene-DDR2 | discoidin domain-containing receptor 2 isoform X1 |
| NC_056054.1 | gene-NLGN1 | neuroligin-1 |
| NC_056058.1 | gene-LMNB2 | lamin-B2 |
| NC_056070.1 | gene-ABCB9 | ABC-type oligopeptide transporter ABCB9 isoform X1 |
| NC_056054.1 | gene-CD247 | T-cell surface glycoprotein CD3 zeta chain precursor |
| NC_056077.1 | gene-TELO2 | telomere length regulation protein TEL2 homolog isoform X1 |
| NC_056054.1 | gene-SLC44A5 | choline transporter-like protein 5 |
| NC_056066.1 | gene-LOC114108777 | cytochrome c |
| NC_056055.1 | gene-GALNT13 | polypeptide N-acetylgalactosaminyltransferase 13 isoform X1 |
| NC_056067.1 | gene-LOC101109733 | zinc finger protein 347-like |
| NC_056055.1 | gene-FSIP2 | fibrous sheath-interacting protein 2 |
| NC_056078.1 | gene-NRG3 | pro-neuregulin-3, membrane-bound isoform isoform X1 |
| NC_056055.1 | gene-MYO1B | unconventional myosin-Ib isoform X1 |
| NC_056076.1 | gene-CCDC178 | coiled-coil domain-containing protein 178 |
| NC_056055.1 | gene-ATP6V1B2 | V-type proton ATPase subunit B, brain isoform isoform X1 |
| NC_056056.1 | gene-CAMKMT | calmodulin-lysine N-methyltransferase |
| NC_056056.1 | gene-PRDM4 | PR domain zinc finger protein 4 isoform X1 |
| NC_056056.1 | gene-CRADD | death domain-containing protein CRADD |
| NC_056054.1 | gene-RCAN1 | calcipressin-1 isoform X1 |
| NC_056059.1 | gene-LOC114115281 | LOW QUALITY PROTEIN: myotubularin-related protein 2-like |
| NC_056055.1 | gene-AOPEP | aminopeptidase O isoform X1 |
| NC_056079.1 | gene-LOC121818076 | - |
| NC_056058.1 | gene-CRTC1 | CREB-regulated transcription coactivator 1 isoform X1 |
| NC_056078.1 | gene-JMJD1C | probable JmjC domain-containing histone demethylation protein 2C isoform X1 |
| NC_056056.1 | gene-SHISAL1 | protein shisa-like-1 isoform X1 |
| NC_056076.1 | gene-ME2 | NAD-dependent malic enzyme, mitochondrial isoform X1 |
| NC_056057.1 | gene-IQUB | IQ and ubiquitin-like domain-containing protein isoform X1 |
| NC_056060.1 | gene-SLC24A1 | sodium/potassium/calcium exchanger 1 |
| NC_056055.1 | gene-SAP30 | histone deacetylase complex subunit SAP30 |
| NC_056076.1 | gene-PRELID3A | PRELI domain containing protein 3A isoform X4 |
| NC_056055.1 | gene-LINGO2 | leucine-rich repeat and immunoglobulin-like domain-containing nogo receptor-interacting protein 2 |
| NC_056058.1 | gene-TICAM1 | TIR domain-containing adapter molecule 1 |
| NC_056056.1 | gene-MYBPC1 | myosin-binding protein C, slow-type isoform X1 |
| NC_056055.1 | gene-GLI2 | zinc finger protein GLI2 isoform X1 |
| NC_056054.1 | gene-OAZ3 | ornithine decarboxylase antizyme 3 isoform 1 |
| NC_056054.1 | gene-TDRKH | tudor and KH domain-containing protein isoform X1 |
| NC_056054.1 | gene-USP40 | ubiquitin carboxyl-terminal hydrolase 40 isoform X1 |
| NC_056054.1 | gene-DGKD | diacylglycerol kinase delta isoform X1 |
| NC_056057.1 | gene-VPS41 | vacuolar protein sorting-associated protein 41 homolog isoform X1 |
| NC_056062.1 | gene-LMBRD1 | lysosomal cobalamin transport escort protein LMBD1 |
| NC_056058.1 | gene-ACSL6 | long-chain-fatty-acid--CoA ligase 6 isoform X6 |
| NC_056073.1 | gene-TJAP1 | tight junction-associated protein 1 isoform X1 |
| NC_056055.1 | gene-FANCC | Fanconi anemia group C protein isoform X1 |
| NC_056054.1 | gene-ETV3L | ETS translocation variant 3-like protein |
| NC_056054.1 | gene-SLC9A9 | sodium/hydrogen exchanger 9 isoform X2 |
| NC_056054.1 | gene-CHAF1B | chromatin assembly factor 1 subunit B |
| NC_056055.1 | gene-PALLD | palladin isoform X1 |
| NC_056055.1 | gene-VPS13A | vacuolar protein sorting-associated protein 13A isoform X1 |
| NC_056056.1 | gene-PHF21B | PHD finger protein 21B isoform X1 |
| NC_056061.1 | gene-NKAIN2 | sodium/potassium-transporting ATPase subunit beta-1-interacting protein 2 isoform X1 |
| NC_056054.1 | gene-TM2D1 | TM2 domain-containing protein 1 isoform X1 |
| NC_056058.1 | gene-HDGFL2 | hepatoma-derived growth factor-related protein 2 isoform X1 |
| NC_056054.1 | gene-MRPL9 | 39S ribosomal protein L9, mitochondrial isoform X1 |
| NC_056059.1 | gene-NAF1 | H/ACA ribonucleoprotein complex non-core subunit NAF1 isoform X1 |
| NC_056057.1 | gene-PRKAG2 | 5'-AMP-activated protein kinase subunit gamma-2 isoform X1 |
| NC_056066.1 | gene-HNF4A | hepatocyte nuclear factor 4-alpha isoform X3 |
| NC_056054.1 | gene-ROBO2 | roundabout homolog 2 isoform X1 |
| NC_056069.1 | gene-TENT4A | terminal nucleotidyltransferase 4A isoform X1 |
| NC_056056.1 | gene-PRDM12 | PR domain zinc finger protein 12 |
| NC_056065.1 | gene-MARK1 | serine/threonine-protein kinase MARK1 isoform X1 |
| NC_056056.1 | gene-CYTH4 | cytohesin-4 isoform X1 |
| NC_056064.1 | gene-SDK2 | protein sidekick-2 isoform X1 |
| NC_056056.1 | gene-LRRK2 | leucine-rich repeat serine/threonine-protein kinase 2 isoform X1 |
| NC_056055.1 | gene-TTN | titin isoform X1 |
| NC_056054.1 | gene-TTC4 | tetratricopeptide repeat protein 4 |
| NC_056063.1 | gene-STARD13 | stAR-related lipid transfer protein 13 isoform X1 |
| NC_056054.1 | gene-ZFYVE9 | zinc finger FYVE domain-containing protein 9 isoform X1 |
| NC_056067.1 | gene-TRNAG-GCC-38 | - |
| NC_056056.1 | gene-LTBP1 | latent-transforming growth factor beta-binding protein 1 isoform X1 |
| NC_056056.1 | gene-LOC106991031 | uncharacterized protein LOC106991031 |
| NC_056055.1 | gene-COL4A4 | collagen alpha-4(IV) chain isoform X1 |
| NC_056054.1 | gene-TXNDC12 | thioredoxin domain-containing protein 12 isoform X1 |
| NC_056054.1 | gene-LOC114118862 | dual specificity protein phosphatase 8-like |
| NC_056054.1 | gene-GSK3B | glycogen synthase kinase-3 beta isoform X1 |
| NC_056058.1 | gene-PTPRS | receptor-type tyrosine-protein phosphatase S isoform X1 |
| NC_056068.1 | gene-LOC101102928 | LOW QUALITY PROTEIN: hemoglobin subunit beta-like |
| NC_056054.1 | gene-LOC101120030 | serine/threonine-protein kinase TNNI3K isoform X1 |
| NC_056070.1 | gene-TMEM132D | transmembrane protein 132D |
| NC_056054.1 | gene-EPHX4 | epoxide hydrolase 4 isoform X1 |
| NC_056056.1 | gene-CRACDL | CRACD-like protein isoform X1 |
| NC_056055.1 | gene-MSRA | mitochondrial peptide methionine sulfoxide reductase isoform X1 |
| NC_056054.1 | gene-KPNA4 | importin subunit alpha-3 |
| NC_056054.1 | gene-LOC114111379 | - |
| NC_056058.1 | gene-CHAF1A | chromatin assembly factor 1 subunit A isoform X1 |
| NC_056059.1 | gene-ATP10D | phospholipid-transporting ATPase VD isoform X1 |
| NC_056058.1 | gene-MEIKIN | meiosis-specific kinetochore protein |
| NC_056054.1 | gene-SELENOF | selenoprotein F isoform X1 |
| NC_056055.1 | gene-XPO7 | exportin-7 isoform X1 |
| NC_056077.1 | gene-LOC101114079 | phospholipid-transporting ATPase ABCA3-like |
| NC_056056.1 | gene-PIK3C2G | phosphatidylinositol 4-phosphate 3-kinase C2 domain-containing subunit gamma isoform X1 |
| NC_056066.1 | gene-CCDC7 | coiled-coil domain-containing protein 7 isoform X1 |
| NC_056056.1 | gene-DYSF | dysferlin isoform X1 |
| NC_056074.1 | gene-TNNI2 | troponin I, fast skeletal muscle |
| NC_056054.1 | gene-ELAVL4 | ELAV-like protein 4 isoform X9 |
| NC_056058.1 | gene-TNFAIP8 | tumor necrosis factor alpha-induced protein 8 isoform X1 |
| NC_056055.1 | gene-DIS3L2 | DIS3-like exonuclease 2 isoform X1 |
| NC_056056.1 | gene-LMO3 | LIM domain only protein 3 isoform X1 |
| NC_056058.1 | gene-PRDX2 | peroxiredoxin-2 |
| NC_056074.1 | gene-LSP1 | lymphocyte-specific protein 1 isoform X1 |
| NC_056055.1 | gene-PTPN4 | tyrosine-protein phosphatase non-receptor type 4 isoform X1 |
| NC_056061.1 | gene-LAMA2 | laminin subunit alpha-2 isoform X1 |
| NC_056054.1 | gene-ABCG1 | ATP-binding cassette sub-family G member 1 isoform X1 |
| NC_056057.1 | gene-PTPRN2 | receptor-type tyrosine-protein phosphatase N2 isoform X1 |
| NC_056054.1 | gene-LOC105609232 | uncharacterized protein LOC105609232 |
| NC_056055.1 | gene-ANXA10 | annexin A10 |
| NC_056055.1 | gene-NFIB | nuclear factor 1 B-type |
| NC_056054.1 | gene-CCDC80 | coiled-coil domain-containing protein 80 |
| NC_056067.1 | gene-SIPA1L3 | signal-induced proliferation-associated 1-like protein 3 isoform X1 |
| NC_056057.1 | gene-CPED1 | cadherin-like and PC-esterase domain-containing protein 1 isoform X1 |
| NC_056073.1 | gene-TRERF1 | transcriptional-regulating factor 1 isoform X1 |
| NC_056054.1 | gene-JAK1 | tyrosine-protein kinase JAK1 |
| NC_056056.1 | gene-NCOA1 | nuclear receptor coactivator 1 isoform X1 |
| NC_056058.1 | gene-SMARCA4 | transcription activator BRG1 isoform X1 |
| NC_056064.1 | gene-MYH10 | myosin-10 isoform X1 |
| NC_056055.1 | gene-AQP7 | aquaporin-7 isoform X1 |
| NC_056060.1 | gene-BUB1B | mitotic checkpoint serine/threonine-protein kinase BUB1 beta isoform X1 |
| NC_056054.1 | gene-NCAM2 | neural cell adhesion molecule 2 isoform X1 |
| NC_056068.1 | gene-TRAF6 | TNF receptor-associated factor 6 isoform X1 |
| NC_056055.1 | gene-LOC101102548 | aldehyde oxidase 2 isoform X1 |
| NC_056056.1 | gene-LRRIQ1 | leucine-rich repeat and IQ domain-containing protein 1 isoform X1 |
| NC_056057.1 | gene-PDE1C | calcium/calmodulin-dependent 3',5'-cyclic nucleotide phosphodiesterase 1C isoform X9 |
| NC_056056.1 | gene-IFT27 | intraflagellar transport protein 27 homolog isoform X1 |
| NC_056055.1 | gene-NTRK2 | BDNF/NT-3 growth factors receptor isoform X1 |
| NC_056071.1 | gene-SCAMP5 | secretory carrier-associated membrane protein 5 isoform X1 |
| NC_056055.1 | gene-LOC101109147 | centromere protein P |
| NC_056055.1 | gene-NOL8 | nucleolar protein 8 |
| NC_056058.1 | gene-KLHL26 | kelch-like protein 26 isoform X1 |
| NC_056071.1 | gene-SNURF | SNRPN upstream reading frame protein |
| NC_056057.1 | gene-RELN | reelin precursor |
| NC_056055.1 | gene-KCNJ3 | G protein-activated inward rectifier potassium channel 1 isoform X1 |
| NC_056054.1 | gene-ATG3 | ubiquitin-like-conjugating enzyme ATG3 |
| NC_056061.1 | gene-KPNA5 | importin subunit alpha-6 |
| NC_056057.1 | gene-COPG2 | coatomer subunit gamma-2 isoform X1 |
| NC_056056.1 | gene-AEBP2 | zinc finger protein AEBP2 isoform X1 |
| NC_056055.1 | gene-LOC101118510 | clusterin-like isoform X1 |
| NC_056054.1 | gene-MYLK | myosin light chain kinase, smooth muscle isoform X2 |
| NC_056054.1 | gene-OSBPL9 | oxysterol-binding protein-related protein 9 isoform X1 |
| NC_056074.1 | gene-MYRF | myelin regulatory factor isoform X1 |
| NC_056057.1 | gene-ADCYAP1R1 | pituitary adenylate cyclase-activating polypeptide type I receptor isoform X1 |
| NC_056067.1 | gene-ERICH4 | glutamate-rich protein 4 |
| NC_056054.1 | gene-THAP4 | peroxynitrite isomerase THAP4 isoform X1 |
| NC_056056.1 | gene-BPIFC | BPI fold-containing family C protein |
| NC_056054.1 | gene-RUNX1 | runt-related transcription factor 1 isoform X1 |
| NC_056066.1 | gene-MACROD2 | ADP-ribose glycohydrolase MACROD2 isoform X1 |
| NC_056054.1 | gene-SMC4 | structural maintenance of chromosomes protein 4 |
| NC_056063.1 | gene-TRNAR-CCU-23 | - |
| NC_056055.1 | gene-BRINP1 | BMP/retinoic acid-inducible neural-specific protein 1 |
| NC_056076.1 | gene-PPP4R1 | serine/threonine-protein phosphatase 4 regulatory subunit 1 isoform X1 |
| NC_056056.1 | gene-SOS1 | son of sevenless homolog 1 isoform X1 |
| NC_056054.1 | gene-GUCA2A | guanylin precursor |
| NC_056054.1 | gene-SNX27 | sorting nexin-27 isoform X1 |
| NC_056056.1 | gene-PTPRB | receptor-type tyrosine-protein phosphatase beta isoform X1 |
| NC_056054.1 | gene-SCLY | selenocysteine lyase isoform X1 |
| NC_056059.1 | gene-LOC101121519 | transmembrane protease serine 11F |
| NC_056055.1 | gene-HERC2 | E3 ubiquitin-protein ligase HERC2 isoform X1 |
| NC_056056.1 | gene-BAIAP2L2 | brain-specific angiogenesis inhibitor 1-associated protein 2-like protein 2 |
| NC_056056.1 | gene-PLA2G6 | 85/88 kDa calcium-independent phospholipase A2 isoform X1 |
| NC_056058.1 | gene-PRTN3 | myeloblastin |
| NC_056054.1 | gene-IFI44 | interferon-induced protein 44 isoform X1 |
| NC_056054.1 | gene-RNPC3 | RNA-binding region-containing protein 3 isoform X1 |
| NC_056056.1 | gene-NTS | neurotensin/neuromedin N |
| NC_056066.1 | gene-SLCO4A1 | solute carrier organic anion transporter family member 4A1 |
| NC_056054.1 | gene-PRKACB | cAMP-dependent protein kinase catalytic subunit beta isoform X1 |
| NC_056055.1 | gene-CLASP1 | CLIP-associating protein 1 isoform X1 |
| NC_056056.1 | gene-SLC9A2 | sodium/hydrogen exchanger 2 |
| NC_056080.1 | gene-PRKX | cAMP-dependent protein kinase catalytic subunit PRKX isoform X1 |
| NC_056056.1 | gene-PPP6R2 | serine/threonine-protein phosphatase 6 regulatory subunit 2 isoform X1 |
| NC_056056.1 | gene-SBF1 | myotubularin-related protein 5 isoform X1 |
| NC_056054.1 | gene-EPHB3 | ephrin type-B receptor 3 |
| NC_056054.1 | gene-PBX1 | pre-B-cell leukemia transcription factor 1 isoform X1 |
| NC_056056.1 | gene-CDKL4 | cyclin-dependent kinase-like 4 |
| NC_056054.1 | gene-PIK3CA | phosphatidylinositol 4,5-bisphosphate 3-kinase catalytic subunit alpha isoform |
| NC_056057.1 | gene-FAM126A | hyccin isoform X1 |
| NC_056055.1 | gene-HACD4 | very-long-chain (3R)-3-hydroxyacyl-CoA dehydratase 4 |
| NC_056055.1 | gene-GPR55 | G-protein coupled receptor 55 |
| NC_056054.1 | gene-DGKG | diacylglycerol kinase gamma isoform X1 |
| NC_056056.1 | gene-LOC101119202 | WD repeat-containing and planar cell polarity effector protein fritz homolog isoform X1 |
| NC_056059.1 | gene-CTBP1 | C-terminal-binding protein 1 isoform X1 |
| NC_056055.1 | gene-INSL6 | insulin-like peptide INSL6 |
| NC_056072.1 | gene-NEK10 | serine/threonine-protein kinase Nek10 isoform X5 |
| NC_056054.1 | gene-PDXK | pyridoxal kinase isoform X1 |
| NC_056068.1 | gene-RELT | tumor necrosis factor receptor superfamily member 19L isoform X1 |
| NC_056058.1 | gene-GNA15 | guanine nucleotide-binding protein subunit alpha-15 isoform X1 |
| NC_056066.1 | gene-PARD6B | partitioning defective 6 homolog beta |
| NC_056054.1 | gene-MME | neprilysin isoform X1 |
| NC_056063.1 | gene-PCDH9 | protocadherin-9 isoform X1 |
| NC_056054.1 | gene-GBE1 | 1,4-alpha-glucan-branching enzyme |
| NC_056071.1 | gene-LOC101114216 | - |
| NC_056055.1 | gene-ICOS | inducible T-cell costimulator isoform X1 |
| NC_056078.1 | gene-LRMDA | leucine-rich melanocyte differentiation-associated protein |
| NC_056054.1 | gene-KCNAB1 | voltage-gated potassium channel subunit beta-1 isoform X1 |
| NC_056056.1 | gene-TMEM250 | transmembrane protein 250 |
| NC_056055.1 | gene-EPB41L5 | band 4.1-like protein 5 isoform X1 |
| NC_056056.1 | gene-WNK1 | serine/threonine-protein kinase WNK1 isoform X1 |
| NC_056055.1 | gene-MYT1L | myelin transcription factor 1-like protein isoform X1 |
| NC_056074.1 | gene-LOC101117288 | glycerophosphodiester phosphodiesterase domain-containing protein 4-like |
| NC_056056.1 | gene-LRP1 | prolow-density lipoprotein receptor-related protein 1 isoform X1 |
| NC_056072.1 | gene-CNTN4 | contactin-4 isoform X1 |
| NC_056058.1 | gene-ADGRE1 | adhesion G protein-coupled receptor E1 isoform X1 |
| NC_056057.1 | gene-IKZF1 | DNA-binding protein Ikaros isoform X1 |
| NC_056056.1 | gene-ITPR2 | inositol 1,4,5-trisphosphate receptor type 2 |
| NC_056067.1 | gene-ZNF536 | zinc finger protein 536 isoform X1 |
| NC_056056.1 | gene-EML4 | echinoderm microtubule-associated protein-like 4 isoform X1 |
| NC_056054.1 | gene-NSUN4 | 5-methylcytosine rRNA methyltransferase NSUN4 isoform X1 |
| NC_056054.1 | gene-DOCK7 | dedicator of cytokinesis protein 7 isoform X1 |
| NC_056054.1 | gene-EPS15 | epidermal growth factor receptor substrate 15 isoform X1 |
| NC_056055.1 | gene-PALM2AKAP2 | A-kinase anchor protein 2 isoform X1 |
| NC_056064.1 | gene-FBXO39 | F-box only protein 39 |
| NC_056054.1 | gene-ACAD11 | acyl-CoA dehydrogenase family member 11 |
| NC_056076.1 | gene-MAPK4 | mitogen-activated protein kinase 4 |
| NC_056057.1 | gene-LOC101111795 | cationic trypsin |
| NC_056056.1 | gene-BID | BH3-interacting domain death agonist |
| NC_056055.1 | gene-DDOST | dolichyl-diphosphooligosaccharide--protein glycosyltransferase 48 kDa subunit |
| NC_056056.1 | gene-PTGES3 | prostaglandin E synthase 3 isoform X1 |
| NC_056058.1 | gene-RAB11B | ras-related protein Rab-11B |
| NC_056056.1 | gene-IL1R2 | interleukin-1 receptor type 2 isoform X1 |
| NC_056056.1 | gene-KCNC2 | potassium voltage-gated channel subfamily C member 2 isoform X1 |
| NC_056069.1 | gene-CPLANE1 | ciliogenesis and planar polarity effector 1 isoform X1 |
| NC_056056.1 | gene-TTC38 | tetratricopeptide repeat protein 38 isoform X1 |
| NC_056058.1 | gene-FGF1 | fibroblast growth factor 1 isoform X1 |
| NC_056058.1 | gene-UBXN6 | UBX domain-containing protein 6 isoform X1 |
| NC_056054.1 | gene-DRD3 | D(3) dopamine receptor |
| NC_056054.1 | gene-MAP6D1 | MAP6 domain-containing protein 1 isoform X1 |
| NC_056061.1 | gene-EYA4 | eyes absent homolog 4 isoform X1 |
| NC_056054.1 | gene-KIF1A | kinesin-like protein KIF1A isoform X1 |
| NC_056069.1 | gene-ADCY2 | adenylate cyclase type 2 |
| NC_056057.1 | gene-SUGCT | succinate--hydroxymethylglutarate CoA-transferase isoform X1 |
| NC_056063.1 | gene-SPATA13 | spermatogenesis-associated protein 13 isoform X1 |
| NC_056058.1 | gene-NOTCH3 | neurogenic locus notch homolog protein 3 isoform X1 |
| NC_056071.1 | gene-TECPR2 | tectonin beta-propeller repeat-containing protein 2 isoform X1 |
| NC_056054.1 | gene-SOD1 | superoxide dismutase [Cu-Zn] |
| NC_056071.1 | gene-KLHL25 | kelch-like protein 25 isoform X1 |
| NC_056054.1 | gene-UBE2U | ubiquitin-conjugating enzyme E2 U isoform X1 |
| NC_056079.1 | gene-PLEKHA2 | pleckstrin homology domain-containing family A member 2 isoform X1 |
| NC_056055.1 | gene-SESN2 | sestrin-2 isoform X1 |
| NC_056063.1 | gene-PIBF1 | progesterone-induced-blocking factor 1 isoform X1 |
| NC_056056.1 | gene-OTOGL | otogelin-like protein |
| NC_056066.1 | gene-GPR158 | probable G-protein coupled receptor 158 isoform X1 |
| NC_056054.1 | gene-MRAS | ras-related protein M-Ras |
| NC_056054.1 | gene-SH2D2A | SH2 domain-containing protein 2A isoform X1 |
| NC_056055.1 | gene-RALB | ras-related protein Ral-B isoform X1 |
| NC_056077.1 | gene-SYT17 | synaptotagmin-17 |
| NC_056055.1 | gene-VWA5B1 | von Willebrand factor A domain-containing protein 5B1 |
| NC_056076.1 | gene-NFATC1 | nuclear factor of activated T-cells, cytoplasmic 1 isoform X1 |
| NC_056054.1 | gene-POU2F1 | POU domain, class 2, transcription factor 1 isoform X1 |
| NC_056062.1 | gene-VPS13B | vacuolar protein sorting-associated protein 13B isoform X1 |
| NC_056055.1 | gene-GFRA2 | GDNF family receptor alpha-2 |
| NC_056079.1 | gene-LOC121818053 | - |
| NC_056055.1 | gene-ARHGAP15 | rho GTPase-activating protein 15 isoform X1 |
| NC_056057.1 | gene-CTTNBP2 | cortactin-binding protein 2 |
| NC_056058.1 | gene-FNIP1 | folliculin-interacting protein 1 isoform X1 |
| NC_056056.1 | gene-RFX4 | transcription factor RFX4 isoform X1 |
| NC_056054.1 | gene-TMPRSS2 | transmembrane protease serine 2 |
| NC_056054.1 | gene-PRKCI | protein kinase C iota type isoform X1 |
| NC_056073.1 | gene-C20H6orf226 | uncharacterized protein C6orf226 homolog |
| NC_056057.1 | gene-DNAH11 | dynein axonemal heavy chain 11 isoform X2 |
| NC_056065.1 | gene-RPS6KC1 | ribosomal protein S6 kinase delta-1 isoform X1 |
| NC_056056.1 | gene-TTC27 | tetratricopeptide repeat protein 27 isoform X1 |
| NC_056058.1 | gene-LOC121819670 | teneurin-2-like |
| NC_056056.1 | gene-LOC106991011 | polycystic kidney disease and receptor for egg jelly-related protein-like |
| NC_056058.1 | gene-SAFB2 | scaffold attachment factor B2 isoform X1 |
| NC_056055.1 | gene-DENND4C | DENN domain-containing protein 4C isoform X1 |
| NC_056054.1 | gene-LRRC40 | leucine-rich repeat-containing protein 40 isoform X1 |
| NC_056054.1 | gene-GRIK3 | glutamate receptor ionotropic, kainate 3 isoform X1 |
| NC_056067.1 | gene-DHODH | dihydroorotate dehydrogenase (quinone), mitochondrial isoform X1 |
| NC_056055.1 | gene-CNTNAP5 | contactin-associated protein-like 5 |
| NC_056067.1 | gene-FTO | alpha-ketoglutarate-dependent dioxygenase FTO |
| NC_056056.1 | gene-PRKCE | protein kinase C epsilon type isoform X1 |
| NC_056070.1 | gene-PXN | paxillin isoform X1 |
| NC_056058.1 | gene-TUBB4A | tubulin beta-4A chain |
| NC_056056.1 | gene-ANKS1B | ankyrin repeat and sterile alpha motif domain-containing protein 1B isoform X1 |
| NC_056055.1 | gene-DNAH7 | dynein axonemal heavy chain 7 isoform X1 |
| NC_056063.1 | gene-CENPJ | centromere protein J isoform X1 |
| NC_056055.1 | gene-ELAVL2 | ELAV-like protein 2 isoform X1 |
| NC_056077.1 | gene-DNASE1 | deoxyribonuclease-1 isoform X1 |
| NC_056056.1 | gene-FBXO41 | F-box only protein 41 |
| NC_056075.1 | gene-LOC114110374 | - |
| NC_056054.1 | gene-GFI1 | zinc finger protein Gfi-1 isoform X1 |
| NC_056065.1 | gene-ADIPOR1 | adiponectin receptor protein 1 |
| NC_056054.1 | gene-SEC22B | vesicle-trafficking protein SEC22b |
| NC_056057.1 | gene-CDK6 | cyclin-dependent kinase 6 isoform X1 |
| NC_056057.1 | gene-VSTM2A | V-set and transmembrane domain-containing protein 2A isoform X1 |
| NC_056054.1 | gene-PIK3CB | phosphatidylinositol 4,5-bisphosphate 3-kinase catalytic subunit beta isoform |
| NC_056074.1 | gene-SLC22A8 | solute carrier family 22 member 8 |
| NC_056055.1 | gene-GLRA3 | glycine receptor subunit alpha-3 isoform X1 |
| NC_056077.1 | gene-PDPK1 | 3-phosphoinositide-dependent protein kinase 1 isoform X1 |
| NC_056056.1 | gene-CERK | ceramide kinase isoform X1 |
| NC_056067.1 | gene-KCTD19 | BTB/POZ domain-containing protein KCTD19 |
| NC_056054.1 | gene-RIIAD1 | RIIa domain-containing protein 1 isoform X1 |
| NC_056054.1 | gene-TBC1D5 | TBC1 domain family member 5 isoform X4 |
| NC_056058.1 | gene-DOT1L | histone-lysine N-methyltransferase, H3 lysine-79 specific isoform X1 |
| NC_056077.1 | gene-IFT140 | intraflagellar transport protein 140 homolog isoform X1 |
| NC_056056.1 | gene-CTNNA2 | catenin alpha-2 isoform X1 |
| NC_056054.1 | gene-PDE4B | cAMP-specific 3',5'-cyclic phosphodiesterase 4B isoform X1 |
| NC_056058.1 | gene-KIF3A | kinesin-like protein KIF3A isoform X1 |
| NC_056059.1 | gene-CLNK | cytokine-dependent hematopoietic cell linker |
| NC_056055.1 | gene-PID1 | PTB-containing, cubilin and LRP1-interacting protein isoform X1 |
| NC_056064.1 | gene-DRC3 | dynein regulatory complex subunit 3 |
| NC_056056.1 | gene-CACNA1C | voltage-dependent L-type calcium channel subunit alpha-1C isoform X8 |
| NC_056059.1 | gene-EPHA5 | ephrin type-A receptor 5 isoform X1 |
| NC_056054.1 | gene-CFAP44 | cilia- and flagella-associated protein 44 isoform X1 |
| NC_056058.1 | gene-CSNK1G3 | casein kinase I isoform X1 |
| NC_056054.1 | gene-MELTF | melanotransferrin isoform X1 |
| NC_056060.1 | gene-SNX1 | sorting nexin-1 isoform X1 |
| NC_056054.1 | gene-NECTIN3 | nectin-3 isoform X1 |
| NC_056071.1 | gene-PPP2R5C | serine/threonine-protein phosphatase 2A 56 kDa regulatory subunit gamma isoform isoform X1 |
| NC_056055.1 | gene-KIF24 | kinesin-like protein KIF24 isoform X1 |
| NC_056058.1 | gene-LOC114114891 | - |
| NC_056056.1 | gene-CDPF1 | cysteine-rich DPF motif domain-containing protein 1 isoform X1 |
| NC_056054.1 | gene-POU1F1 | pituitary-specific positive transcription factor 1 isoform X1 |
| NC_056055.1 | gene-ADAM28 | disintegrin and metalloproteinase domain-containing protein 28 isoform X1 |
| NC_056056.1 | gene-TMTC1 | protein O-mannosyl-transferase TMTC1 isoform X1 |
| NC_056065.1 | gene-UBE4B | ubiquitin conjugation factor E4 B isoform X1 |
| NC_056056.1 | gene-KCNT1 | potassium channel subfamily T member 1 isoform X1 |
| NC_056055.1 | gene-TTLL4 | tubulin polyglutamylase TTLL4 isoform X1 |
| NC_056056.1 | gene-ZNF384 | zinc finger protein 384 isoform X1 |
| NC_056060.1 | gene-IGDCC3 | immunoglobulin superfamily DCC subclass member 3 isoform X1 |
| NC_056055.1 | gene-MFF | mitochondrial fission factor isoform X1 |
| NC_056065.1 | gene-TMEM9 | proton-transporting V-type ATPase complex assembly regulator TMEM9 |
| NC_056058.1 | gene-ELL | RNA polymerase II elongation factor ELL isoform X1 |
| NC_056055.1 | gene-BMP1 | bone morphogenetic protein 1 isoform X1 |
| NC_056056.1 | gene-LOC101123029 | C-type lectin domain family 7 member A-like isoform X1 |
| NC_056064.1 | gene-CACNA1G | voltage-dependent T-type calcium channel subunit alpha-1G isoform X21 |
| NC_056057.1 | gene-CREB3L2 | cyclic AMP-responsive element-binding protein 3-like protein 2 |
| NC_056079.1 | gene-LOC105605237 | A disintegrin and metallopeptidase domain 3-like |
| NC_056054.1 | gene-LOC101104028 | limbic system-associated membrane protein isoform X1 |
| NC_056060.1 | gene-MAPK1IP1L | MAPK-interacting and spindle-stabilizing protein-like |
| NC_056055.1 | gene-PTPN18 | tyrosine-protein phosphatase non-receptor type 18 isoform X1 |
| NC_056078.1 | gene-SYNPO2L | synaptopodin 2-like protein isoform X1 |
| NC_056054.1 | gene-LEKR1 | leucine-, glutamate- and lysine-rich protein 1 |
| NC_056061.1 | gene-SMOC2 | SPARC-related modular calcium-binding protein 2 isoform X1 |
| NC_056055.1 | gene-ASTN2 | astrotactin-2 isoform X1 |
| NC_056057.1 | gene-CNOT4 | CCR4-NOT transcription complex subunit 4 isoform X1 |
| NC_056056.1 | gene-SEPTIN10 | septin-10 isoform X1 |
| NC_056054.1 | gene-ZNF654 | zinc finger protein 654 isoform X1 |
| NC_056065.1 | gene-GNB1 | guanine nucleotide-binding protein G(I)/G(S)/G(T) subunit beta-1 |
| NC_056057.1 | gene-VPS50 | syndetin |
| NC_056066.1 | gene-NEBL | nebulette isoform X1 |
| NC_056054.1 | gene-EVI5 | ecotropic viral integration site 5 protein homolog isoform X1 |
| NC_056065.1 | gene-KMO | LOW QUALITY PROTEIN: kynurenine 3-monooxygenase |
| NC_056054.1 | gene-LURAP1 | leucine rich adaptor protein 1 |
| NC_056055.1 | gene-BMPR2 | bone morphogenetic protein receptor type-2 precursor |
| NC_056056.1 | gene-RAC2 | ras-related C3 botulinum toxin substrate 2 |
| NC_056080.1 | gene-CHRDL1 | chordin-like protein 1 isoform X1 |
| NC_056058.1 | gene-MLLT1 | protein ENL isoform X1 |
| NC_056056.1 | gene-LRP6 | low-density lipoprotein receptor-related protein 6 isoform X1 |
| NC_056055.1 | gene-DPP4 | dipeptidyl peptidase 4 isoform X1 |
| NC_056055.1 | gene-LOC114112980 | - |
| NC_056055.1 | gene-GDA | guanine deaminase |
| NC_056054.1 | gene-COL6A2 | collagen alpha-2(VI) chain isoform X1 |
| NC_056056.1 | gene-LOC101122683 | LOW QUALITY PROTEIN: ovostatin homolog 2-like |
| NC_056060.1 | gene-GLCE | D-glucuronyl C5-epimerase isoform X1 |
| NC_056056.1 | gene-RASSF8 | ras association domain-containing protein 8 |
| NC_056057.1 | gene-LRGUK | leucine-rich repeat and guanylate kinase domain-containing protein isoform X1 |
| NC_056054.1 | gene-EPHA3 | ephrin type-A receptor 3 isoform X1 |
| NC_056054.1 | gene-LOC114113187 | histone H4 |
| NC_056056.1 | gene-TCF20 | transcription factor 20 isoform X1 |
| NC_056072.1 | gene-PRICKLE2 | prickle-like protein 2 isoform X1 |
| NC_056054.1 | gene-LOC101123247 | phosphatidylinositol 3-kinase regulatory subunit gamma isoform X1 |
| NC_056059.1 | gene-LOC121819875 | - |
| NC_056056.1 | gene-LHCGR | lutropin-choriogonadotropic hormone receptor isoform X1 |
| NC_056058.1 | gene-XRCC4 | DNA repair protein XRCC4 isoform X1 |
| NC_056055.1 | gene-METTL21A | protein N-lysine methyltransferase METTL21A isoform X1 |
| NC_056071.1 | gene-APBA2 | amyloid-beta A4 precursor protein-binding family A member 2 isoform X1 |
| NC_056055.1 | gene-NEB | nebulin isoform X27 |
| NC_056070.1 | gene-CORO1C | coronin-1C |
| NC_056056.1 | gene-LAPTM4A | lysosomal-associated transmembrane protein 4A |
| NC_056056.1 | gene-ADIPOR2 | adiponectin receptor protein 2 |
| NC_056058.1 | gene-CPAMD8 | C3 and PZP-like alpha-2-macroglobulin domain-containing protein 8 |
| NC_056056.1 | gene-SPTBN1 | spectrin beta chain, non-erythrocytic 1 isoform X1 |
| NC_056054.1 | gene-LOC101122496 | multidrug resistance-associated protein 1-like |
| NC_056072.1 | gene-ERC2 | ERC protein 2 isoform X1 |
| NC_056054.1 | gene-USP33 | ubiquitin carboxyl-terminal hydrolase 33 isoform X1 |
| NC_056075.1 | gene-MINPP1 | multiple inositol polyphosphate phosphatase 1 isoform X1 |

**Table S28 The KEGG enrichment pathways of common genes in unique SNPs and the selective sweep region of Tibetan sheep relative to Argali (*P* < 0.05)**

| **PathwayID** | **Pathway** | ***P*_value (KEGG richment of unique SNPs)** | ***P*_value (KEGG richment of selective elimination)** |
| --- | --- | --- | --- |
| ko00562 | Inositol phosphate metabolism | 0.000375 | 0.020689 |
| ko04724 | Glutamatergic synapse | 0.001345 | 0.004415 |
| ko04973 | Carbohydrate digestion and absorption | 0.003228 | 0.000496 |
| ko04725 | Cholinergic synapse | 0.005666 | 0.000005 |
| ko04072 | Phospholipase D signaling pathway | 0.009711 | 0.000093 |
| ko04611 | Platelet activation | 0.014543 | 0.000930 |
| ko04070 | Phosphatidylinositol signaling system | 0.018779 | 0.022748 |
| ko04915 | Estrogen signaling pathway | 0.033398 | 0.000058 |
| ko04911 | Insulin secretion | 0.043571 | 0.000428 |

**Table S29 The GO enrichment pathways of common genes in unique SNPs and the selective sweep region of Tibetan sheep relative to Argali (*P* < 0.05)**

| **Category** | **GO.ID** | **Term** | ***P*_value**  **(GO richment of unique SNPs)** | ***P*_value**  **(GO richment of selective elimination)** |
| --- | --- | --- | --- | --- |
| MF | GO:0032559 | adenyl ribonucleotide binding | 2.24E-18 | 2.32E-07 |
| MF | GO:0005524 | ATP binding | 2.63E-18 | 0.000001 |
| MF | GO:0030554 | adenyl nucleotide binding | 8.31E-18 | 0.000000 |
| MF | GO:0043167 | ion binding | 4.98E-13 | 0.000235 |
| MF | GO:0003774 | motor activity | 9.23E-11 | 0.014860 |
| MF | GO:0043168 | anion binding | 4.62E-09 | 0.000220 |
| MF | GO:0097367 | carbohydrate derivative binding | 5.09E-09 | 0.000048 |
| CC | GO:0005856 | cytoskeleton | 7.7E-09 | 0.000377 |
| MF | GO:0000166 | nucleotide binding | 2.36E-08 | 0.000130 |
| MF | GO:1901265 | nucleoside phosphate binding | 2.36E-08 | 0.000130 |
| CC | GO:0071944 | cell periphery | 3.88E-08 | 0.000009 |
| MF | GO:0032555 | purine ribonucleotide binding | 4.20E-08 | 0.000032 |
| MF | GO:0035639 | purine ribonucleoside triphosphate binding | 4.57E-08 | 0.000069 |
| CC | GO:0005886 | plasma membrane | 7.41E-08 | 0.000015 |
| MF | GO:0032553 | ribonucleotide binding | 8.94E-08 | 0.000031 |
| MF | GO:0017076 | purine nucleotide binding | 1.92E-07 | 0.000020 |
| MF | GO:0005085 | guanyl-nucleotide exchange factor activity | 0.000001 | 0.004365 |
| MF | GO:0004714 | transmembrane receptor protein tyrosine kinase activity | 0.000001 | 0.000000 |
| MF | GO:0003777 | microtubule motor activity | 0.000002 | 0.016498 |
| MF | GO:0051020 | GTPase binding | 0.000004 | 0.032783 |
| MF | GO:0036094 | small molecule binding | 0.000005 | 0.000126 |
| CC | GO:0120025 | plasma membrane bounded cell projection | 0.000006 | 0.000000 |
| MF | GO:0019199 | transmembrane receptor protein kinase activity | 0.000006 | 0.000000 |
| CC | GO:0042995 | cell projection | 0.000009 | 0.000000 |
| MF | GO:0004713 | protein tyrosine kinase activity | 0.000034 | 0.000000 |
| MF | GO:0008092 | cytoskeletal protein binding | 0.000060 | 0.002190 |
| MF | GO:0016301 | kinase activity | 0.000119 | 0.000000 |
| BP | GO:0007156 | homophilic cell adhesion via plasma membrane adhesion molecules | 0.000129 | 0.006855 |
| BP | GO:0046777 | protein autophosphorylation | 0.000130 | 0.009626 |
| MF | GO:0016773 | phosphotransferase activity, alcohol group as acceptor | 0.000158 | 0.000000 |
| CC | GO:0015630 | microtubule cytoskeleton | 0.000251 | 0.014897 |
| BP | GO:0007010 | cytoskeleton organization | 0.000291 | 0.006013 |
| CC | GO:0044304 | main axon | 0.000599 | 0.015440 |
| MF | GO:0016772 | transferase activity, transferring phosphorus-containing groups | 0.000612 | 0.000000 |
| MF | GO:0003779 | actin binding | 0.000634 | 0.000861 |
| CC | GO:0099080 | supramolecular complex | 0.000653 | 0.028203 |
| CC | GO:0098590 | plasma membrane region | 0.000747 | 0.000032 |
| BP | GO:0007155 | cell adhesion | 0.000752 | 0.003944 |
| CC | GO:0070161 | anchoring junction | 0.000827 | 0.030543 |
| CC | GO:0030055 | cell-substrate junction | 0.000908 | 0.020305 |
| MF | GO:0046873 | metal ion transmembrane transporter activity | 0.000996 | 0.040353 |
| MF | GO:0004672 | protein kinase activity | 0.001102 | 0.000000 |
| CC | GO:0005925 | focal adhesion | 0.002154 | 0.014052 |
| BP | GO:0043087 | regulation of GTPase activity | 0.002175 | 0.018221 |
| CC | GO:0099568 | cytoplasmic region | 0.002485 | 0.000707 |
| MF | GO:0022836 | gated channel activity | 0.002540 | 0.033157 |
| BP | GO:0044782 | cilium organization | 0.002541 | 0.000053 |
| CC | GO:0005887 | integral component of plasma membrane | 0.002789 | 0.000606 |
| BP | GO:0120031 | plasma membrane bounded cell projection assembly | 0.002804 | 0.000044 |
| BP | GO:0030031 | cell projection assembly | 0.003098 | 0.000026 |
| BP | GO:0007017 | microtubule-based process | 0.003261 | 0.028173 |
| BP | GO:0060271 | cilium assembly | 0.003511 | 0.000030 |
| MF | GO:0008569 | ATP-dependent microtubule motor activity, minus-end-directed | 0.003587 | 0.006419 |
| CC | GO:0031226 | intrinsic component of plasma membrane | 0.004374 | 0.000188 |
| BP | GO:0051056 | regulation of small GTPase mediated signal transduction | 0.005559 | 0.014622 |
| CC | GO:0005938 | cell cortex | 0.005760 | 0.019678 |
| CC | GO:0030425 | dendrite | 0.005823 | 0.001301 |
| CC | GO:0097447 | dendritic tree | 0.005823 | 0.001301 |
| CC | GO:0005929 | cilium | 0.005833 | 0.002617 |
| BP | GO:0098742 | cell-cell adhesion via plasma-membrane adhesion molecules | 0.005918 | 0.031639 |
| CC | GO:0043005 | neuron projection | 0.006188 | 0.000474 |
| CC | GO:0014069 | postsynaptic density | 0.006352 | 0.000264 |
| CC | GO:0032279 | asymmetric synapse | 0.006352 | 0.000264 |
| MF | GO:0015297 | antiporter activity | 0.008341 | 0.023394 |
| MF | GO:0140096 | catalytic activity, acting on a protein | 0.009648 | 0.000020 |
| MF | GO:0005096 | GTPase activator activity | 0.010837 | 0.032360 |
| MF | GO:0019899 | enzyme binding | 0.011076 | 0.005851 |
| MF | GO:0042169 | SH2 domain binding | 0.011155 | 0.039322 |
| MF | GO:0016740 | transferase activity | 0.011578 | 0.000465 |
| CC | GO:0005930 | axoneme | 0.013181 | 0.001470 |
| CC | GO:0031256 | leading edge membrane | 0.013181 | 0.000128 |
| CC | GO:0097014 | ciliary plasm | 0.013181 | 0.001470 |
| MF | GO:0031267 | small GTPase binding | 0.014880 | 0.012089 |
| CC | GO:0015629 | actin cytoskeleton | 0.015818 | 0.002059 |
| BP | GO:0120036 | plasma membrane bounded cell projection organization | 0.017714 | 0.000000 |
| CC | GO:0031253 | cell projection membrane | 0.017775 | 0.002011 |
| BP | GO:0072673 | lamellipodium morphogenesis | 0.017973 | 0.010044 |
| BP | GO:1905515 | non-motile cilium assembly | 0.018009 | 0.002168 |
| BP | GO:0050764 | regulation of phagocytosis | 0.019508 | 0.024077 |
| MF | GO:0030374 | nuclear receptor transcription coactivator activity | 0.020121 | 0.036539 |
| CC | GO:0098984 | neuron to neuron synapse | 0.020160 | 0.000562 |
| CC | GO:0030054 | cell junction | 0.021656 | 0.000731 |
| BP | GO:0007626 | locomotory behavior | 0.023140 | 0.000574 |
| BP | GO:0061318 | renal filtration cell differentiation | 0.025129 | 0.045661 |
| BP | GO:0072112 | glomerular visceral epithelial cell differentiation | 0.025129 | 0.045661 |
| BP | GO:0072311 | glomerular epithelial cell differentiation | 0.025129 | 0.045661 |
| BP | GO:0007264 | small GTPase mediated signal transduction | 0.028092 | 0.007191 |
| MF | GO:0030594 | neurotransmitter receptor activity | 0.028606 | 0.048114 |
| BP | GO:0016242 | negative regulation of macroautophagy | 0.029697 | 0.011737 |
| BP | GO:0000226 | microtubule cytoskeleton organization | 0.030031 | 0.047903 |
| MF | GO:0015298 | solute:cation antiporter activity | 0.031742 | 0.010327 |
| BP | GO:0030030 | cell projection organization | 0.033847 | 0.000000 |
| MF | GO:0030695 | GTPase regulator activity | 0.034580 | 0.000648 |
| MF | GO:0022843 | voltage-gated cation channel activity | 0.034611 | 0.026206 |
| MF | GO:0042578 | phosphoric ester hydrolase activity | 0.035106 | 0.002335 |
| CC | GO:0030119 | AP-type membrane coat adaptor complex | 0.037824 | 0.018158 |
| BP | GO:0030029 | actin filament-based process | 0.038956 | 0.005599 |
| CC | GO:0031252 | cell leading edge | 0.039074 | 0.000199 |
| BP | GO:0071897 | DNA biosynthetic process | 0.039501 | 0.027332 |
| CC | GO:0044309 | neuron spine | 0.043122 | 0.049153 |
| BP | GO:0030036 | actin cytoskeleton organization | 0.043599 | 0.005823 |
| BP | GO:0048384 | retinoic acid receptor signaling pathway | 0.045856 | 0.023072 |
| BP | GO:0007099 | centriole replication | 0.046931 | 0.037374 |
| BP | GO:0038084 | vascular endothelial growth factor signaling pathway | 0.048114 | 0.014927 |
| CC | GO:0098794 | postsynapse | 0.049304 | 0.000157 |
| BP | GO:0098609 | cell-cell adhesion | 0.049420 | 0.034971 |
| CC | GO:0099572 | postsynaptic specialization | 0.049927 | 0.001401 |

**Table S30** The results of transcriptome in heart and lung tissues for Zhashijia sheep and Valley Tibetan sheep

| **Sample** | **Total_clean_reads（M)** | **Total_clean_bases (G)** | **Q20 (%)** | **Q30 (%)** | **Clean_reads_ratio (%)** |
| --- | --- | --- | --- | --- | --- |
| HJ1 | 44.45 | 6.67 | 98.24 | 93.82 | 97.82 |
| HJ2 | 44.60 | 6.69 | 98.31 | 94.02 | 98.17 |
| HJ3 | 44.27 | 6.64 | 98.20 | 93.73 | 97.44 |
| HJ4 | 43.87 | 6.58 | 98.28 | 94.06 | 96.55 |
| HS1 | 44.12 | 6.62 | 98.25 | 93.92 | 97.10 |
| HS2 | 44.16 | 6.62 | 98.29 | 94.10 | 97.18 |
| HS3 | 44.35 | 6.65 | 98.20 | 93.72 | 97.60 |
| HS4 | 44.34 | 6.65 | 98.35 | 94.24 | 97.59 |
| LJ1 | 44.30 | 6.64 | 98.25 | 93.94 | 97.49 |
| LJ2 | 44.37 | 6.66 | 98.07 | 93.34 | 97.65 |
| LJ3 | 44.42 | 6.66 | 98.26 | 93.98 | 97.77 |
| LJ4 | 44.43 | 6.66 | 98.23 | 93.81 | 97.77 |
| LS1 | 44.15 | 6.62 | 98.25 | 93.97 | 97.16 |
| LS2 | 44.12 | 6.62 | 98.24 | 93.91 | 97.10 |
| LS3 | 44.01 | 6.60 | 98.22 | 93.92 | 96.86 |
| LS4 | 44.32 | 6.65 | 98.23 | 93.87 | 97.55 |
| Average | 44.27 | 6.64 | 98.24 | 93.90 | 97.43 |

HJ: The heart of Zhashijia sheep. HS: The heart of Valley Tibetan sheep. LJ: The lung of Zhashijia sheep. LS: The lung of Valley Tibetan sheep

**Table S31** The KEGG enrichment of differentially expressed genes in heart transcriptome for Zhashijia sheep and Valley Tibetan sheep (*P* < 0.05)

| **PathwayID** | **Pathway** | **List_number** | **Total_number** | ***P*_value** | ***Q*_value** |
| --- | --- | --- | --- | --- | --- |
| ko4512 | ECM-receptor interaction | 18 | 187 | 5.22E-13 | 1.43E-10 |
| ko4974 | Protein digestion and absorption | 18 | 187 | 3.30E-10 | 4.54E-08 |
| ko4510 | Focal adhesion | 21 | 187 | 2.55E-09 | 2.34E-07 |
| ko5205 | Proteoglycans in cancer | 16 | 187 | 0.000011 | 0.000784 |
| ko4933 | AGE-RAGE signaling pathway in diabetic complications | 11 | 187 | 0.000015 | 0.000825 |
| ko4151 | PI3K-Akt signaling pathway | 21 | 187 | 0.000061 | 0.002796 |
| ko5146 | Amoebiasis | 10 | 187 | 0.000107 | 0.003834 |
| ko4926 | Relaxin signaling pathway | 11 | 187 | 0.000112 | 0.003834 |
| ko5165 | Human papillomavirus infection | 18 | 187 | 0.000649 | 0.019824 |
| ko5418 | Fluid shear stress and atherosclerosis | 10 | 187 | 0.001166 | 0.032076 |
| ko0071 | Fatty acid degradation | 5 | 187 | 0.003095 | 0.077381 |
| ko0514 | Other types of O-glycan biosynthesis | 5 | 187 | 0.003733 | 0.085559 |
| ko5144 | Malaria | 5 | 187 | 0.005734 | 0.121290 |
| ko0100 | Steroid biosynthesis | 3 | 187 | 0.011561 | 0.209348 |
| ko5415 | Diabetic cardiomyopathy | 10 | 187 | 0.013663 | 0.209348 |
| ko4912 | GnRH signaling pathway | 6 | 187 | 0.015003 | 0.209348 |
| ko5410 | Hypertrophic cardiomyopathy | 6 | 187 | 0.015003 | 0.209348 |
| ko4361 | Axon regeneration | 6 | 187 | 0.015756 | 0.209348 |
| ko4935 | Growth hormone synthesis, secretion and action | 7 | 187 | 0.016396 | 0.209348 |
| ko4261 | Adrenergic signaling in cardiomyocytes | 8 | 187 | 0.016665 | 0.209348 |
| ko4611 | Platelet activation | 7 | 187 | 0.017793 | 0.209348 |
| ko5222 | Small cell lung cancer | 6 | 187 | 0.018173 | 0.209348 |
| ko4514 | Cell adhesion molecules | 8 | 187 | 0.018559 | 0.209348 |
| ko1522 | Endocrine resistance | 6 | 187 | 0.019032 | 0.209348 |
| ko5414 | Dilated cardiomyopathy | 6 | 187 | 0.019032 | 0.209348 |
| Ko0380 | Tryptophan metabolism | 4 | 187 | 0.020155 | 0.213182 |
| ko4979 | Cholesterol metabolism | 4 | 187 | 0.021584 | 0.219835 |
| ko5200 | Pathways in cancer | 19 | 187 | 0.026855 | 0.249844 |
| ko4750 | Inflammatory mediator regulation of TRP channels | 6 | 187 | 0.028035 | 0.249844 |
| ko4010 | MAPK signaling pathway | 12 | 187 | 0.028911 | 0.249844 |
| ko0062 | Fatty acid elongation | 3 | 187 | 0.029265 | 0.249844 |
| ko4710 | Circadian rhythm | 3 | 187 | 0.029265 | 0.249844 |
| ko4270 | Vascular smooth muscle contraction | 7 | 187 | 0.029981 | 0.249844 |
| ko5171 | Coronavirus disease - COVID-19 | 13 | 187 | 0.033921 | 0.274357 |
| ko4020 | Calcium signaling pathway | 10 | 187 | 0.035193 | 0.276513 |
| ko5145 | Toxoplasmosis | 6 | 187 | 0.038086 | 0.290935 |
| ko4670 | Leukocyte transendothelial migration | 6 | 187 | 0.039485 | 0.293469 |
| ko0512 | Mucin type O-glycan biosynthesis | 3 | 187 | 0.042977 | 0.303997 |
| ko0300 | Lysine biosynthesis | 1 | 187 | 0.043112 | 0.303997 |
| ko4960 | Aldosterone-regulated sodium reabsorption | 3 | 187 | 0.049215 | 0.338351 |

**Table S32** The GO enrichment of differentially expressed genes in heart transcriptome for Zhashijia sheep and Valley Tibetan sheep (*P* < 0.05)

| **Category** | **GO.ID** | **Term** | **List** | **Total** | ***P*_value** | ***Q*_value** |
| --- | --- | --- | --- | --- | --- | --- |
| CC | GO:0062023 | collagen-containing extracellular matrix | 20 | 371 | 1.32E-17 | 3.95E-15 |
| CC | GO:0031012 | extracellular matrix | 24 | 371 | 5.90E-16 | 8.84E-14 |
| CC | GO:0005581 | collagen trimer | 16 | 371 | 9.05E-13 | 9.05E-11 |
| CC | GO:0005604 | basement membrane | 15 | 371 | 2.14E-11 | 1.61E-09 |
| CC | GO:0005576 | extracellular region | 62 | 371 | 5.08E-11 | 3.05E-09 |
| CC | GO:0005615 | extracellular space | 39 | 371 | 2.18E-07 | 0.000011 |
| CC | GO:0005588 | collagen type V trimer | 3 | 371 | 0.000014 | 0.000591 |
| CC | GO:0005584 | collagen type I trimer | 2 | 371 | 0.000577 | 0.019222 |
| CC | GO:0038143 | ERBB3:ERBB2 complex | 2 | 371 | 0.000577 | 0.019222 |
| CC | GO:0001527 | microfibril | 3 | 371 | 0.000706 | 0.021177 |
| CC | GO:0005911 | cell-cell junction | 10 | 371 | 0.001756 | 0.047899 |
| CC | GO:0005614 | interstitial matrix | 3 | 371 | 0.001971 | 0.049276 |
| CC | GO:0045121 | membrane raft | 9 | 371 | 0.002738 | 0.063183 |
| CC | GO:0030426 | growth cone | 5 | 371 | 0.005241 | 0.112303 |
| CC | GO:0005587 | collagen type IV trimer | 2 | 371 | 0.008113 | 0.162259 |
| CC | GO:0009897 | external side of plasma membrane | 9 | 371 | 0.010411 | 0.171755 |
| CC | GO:0030425 | dendrite | 9 | 371 | 0.011291 | 0.171755 |
| CC | GO:0030424 | axon | 9 | 371 | 0.013734 | 0.171755 |
| CC | GO:0042995 | cell projection | 11 | 371 | 0.018747 | 0.171755 |
| CC | GO:0005586 | collagen type III trimer | 1 | 371 | 0.024046 | 0.171755 |
| CC | GO:0005607 | laminin-2 complex | 1 | 371 | 0.024046 | 0.171755 |
| CC | GO:0005668 | RNA polymerase transcription factor SL1 complex | 1 | 371 | 0.024046 | 0.171755 |
| CC | GO:0008287 | protein serine/threonine phosphatase complex | 1 | 371 | 0.024046 | 0.171755 |
| CC | GO:0016507 | mitochondrial fatty acid beta-oxidation multienzyme complex | 1 | 371 | 0.024046 | 0.171755 |
| CC | GO:0032798 | Swi5-Sfr1 complex | 1 | 371 | 0.024046 | 0.171755 |
| CC | GO:0034668 | integrin alpha4-beta1 complex | 1 | 371 | 0.024046 | 0.171755 |
| CC | GO:0034681 | integrin alpha11-beta1 complex | 1 | 371 | 0.024046 | 0.171755 |
| CC | GO:0043257 | laminin-8 complex | 1 | 371 | 0.024046 | 0.171755 |
| CC | GO:0060987 | lipid tube | 1 | 371 | 0.024046 | 0.171755 |
| CC | GO:0071540 | eukaryotic translation initiation factor 3 complex, eIF3e | 1 | 371 | 0.024046 | 0.171755 |
[truncated: 132,612 more chars]
